# Mechanistic analyses in kidney transplant recipients prospectively randomized to two steroid free regimen—Low dose Tacrolimus with Everolimus versus standard dose Tacrolimus with Mycophenolate Mofetil

**DOI:** 10.1371/journal.pone.0216300

**Published:** 2019-05-28

**Authors:** Opas Traitanon, James M. Mathew, Aneesha Shetty, Sai Vineela Bontha, Daniel G. Maluf, Yvonne El Kassis, Sook H. Park, Jing Han, M. Javeed Ansari, Joseph R. Leventhal, Valeria Mas, Lorenzo Gallon

**Affiliations:** 1 Department of Medicine-Nephrology, Northwestern University, Chicago, IL, United States of America; 2 Department of Medicine-Nephrology, Thammasart University Hospital, Pathumthani, Thailand; 3 Department of Surgery, Northwestern University, Chicago, IL, United States of America; 4 Comprehensive Transplant Center, Northwestern University, Chicago, IL, United States of America; 5 Department of Microbiology-Immunology, Northwestern University, Chicago, IL, United States of America; 6 Methodist University Transplant Institute; University of Tennessee Health Science Center; Memphis, TN, United States of America; Icahn School of Medicine at Mount Sinai, UNITED STATES

## Abstract

Calcineurin inhibitors (CNI), the cornerstone of immunosuppression after transplantation are implicated in nephrotoxicity and allograft dysfunction. We hypothesized that combined low doses of CNI and Everolimus (EVR) may result in better graft outcomes and greater tolerogenic milieu. Forty adult renal transplant recipients were prospectively randomized to (steroid free) low dose Tacrolimus (TAC) and EVR or standard dose TAC and Mycophenolate (MMF) after Alemtuzumab induction. Baseline characteristics were statistically similar. EVR levels were maintained at 3–8 ng/ml. TAC levels were 4.5±1.9 and 6.4±1.5 ng/ml in the TAC+EVR and TAC+MMF group respectively. Follow up was 14±4 and 17±5 months respectively and included protocol kidney biopsies at 3 and 12 months post-transplantation. Rejection-rate was lower in the TAC+EVR group. However patient and overall graft survival, eGFR and incidence of adverse events were similar. TAC+EVR induced expansion of CD4^+^CD25^hi^Foxp3^+^ regulatory T cells as early as 3 months and expansion of IFN-γ^+^CD4^+^CD25^hi^Foxp3^+^ regulatory T cells at 12 months post-transplant. Gene expression profile showed a trend toward decreased inflammation, angiogenesis and connective tissue growth in the TAC+EVR Group. Thus, greater tolerogenic mechanisms were found to be operating in patients with low dose TAC+EVR and this might be responsible for the lower rejection-rate than in patients on standard dose TAC+MMF. However, further studies with longer follow up and evaluating impact on T regulatory cells are warranted.

## Introduction

The advent of calcineurin inhibitor (CNI) based immunosuppression (IS) changed the face of kidney transplantation (KT), dramatically improving short term graft and patient outcomes. However, long term CNI exposure has been associated with poorer graft function, increased risk of cardiovascular events and glucose intolerance [1–3]. Histological features of chronic CNI nephrotoxicity include irreversible and progressive tubular atrophy, interstitial fibrosis, and focal hyalinosis of small renal arteries and arterioles [4]. Additionally, CNIs block IL2 production leading to a negative impact on regulatory T cell (Treg) generation (an important subpopulation of T helper cells that has been associated with positive immunomodulation and donor specific hypo responsiveness). Attempts at complete avoidance of CNIs have been associated with increased cellular rejection [5] while alternative regimens like combination of a full dose CNI with an mTOR inhibitor has been shown to be synergistically nephrotoxic [6].

Various strategies to minimize CNI exposure and consequently improve graft outcomes have been studied [7]. The A2309 study comparing reduced dose Cyclosporine (CsA) + Everolimus (EVR) with standard dose CsA + Mycophenolate Mofetil (MMF) is one such study, which showed equivalent graft outcomes between the 2 groups and earned Everolimus FDA approval for use in KT [8]. However, previous trials have shown superior graft survival with tacrolimus (TAC) when compared with CsA [9–11] and TAC based regimen is now the standard of care in most institutions.

**Fig 1:**
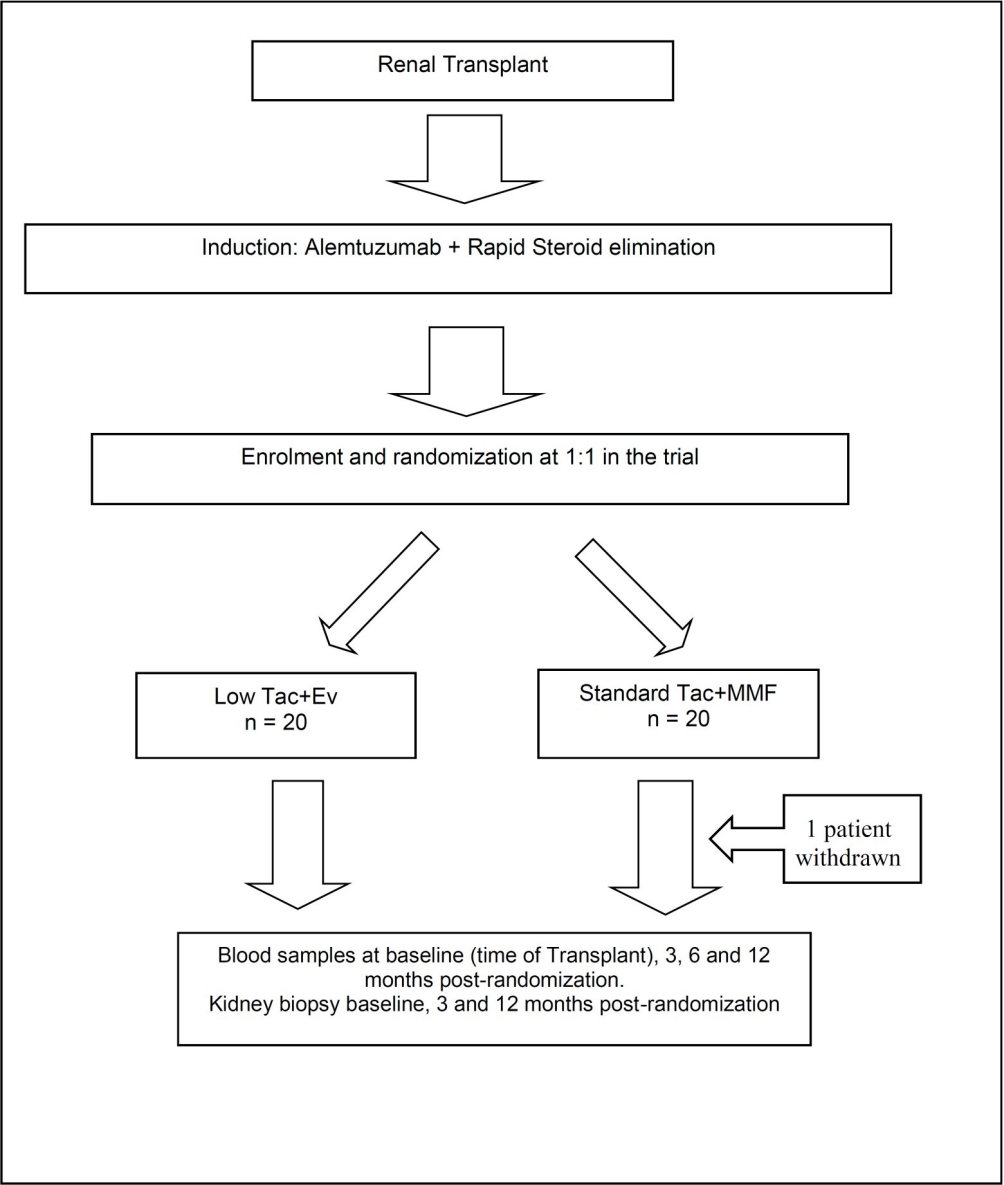
Consort diagram of enrollment. Please see S3 File, Consort Checklist for additional information.

Herein, we evaluated the combination of low dose TAC+EVR when compared to standard dose TAC+MMF in patients who received T-cell depleting induction therapy followed by steroid free immunosuppression (Fig 1). A detailed longitudinal intragraft gene expression and peripheral blood T cell subset analysis has been done for patients groups receiving TAC+MMF versus those receiving low dose TAC+EVR. We hypothesized that the positive effect of Everolimus on expansion of Tregs combined with low exposure of TAC is sufficient to control allo-reactive T cells translating into better renal allograft outcomes. We observed a greater tolerogenic melieu operating in patients with low dose TAC+EVR and this might be responsible for the lower rejection-rate than in patients on standard dose TAC+MMF.

## Materials and methods

We conducted a single-center prospective randomized controlled pilot trial (NCT01653847) to study the immune mechanisms operating in adult non-sensitized living donor KT recipients receiving low dose TAC+EVR vs. standard dose TAC+MMF immunosuppressive regimen (Please see S2 File for the Clinical Trial Protocol). Recipients between ages 18–70 were recruited from February 1, 2013 to May 29, 2014 through a Northwestern University Institutional Review Board (IRB) approved protocol after obtaining written informed consent. The randomization was made by a non-study personnel using the online “sealed envelope” randomization service (https://www.sealedenvelope.com/simple-randomiser/v1/). All procedures followed were in accordance with the ethical standards of the responsible committee on human experimentation (institutional and national) and with the Helsinki Declaration of 1975, as revised in 2008. Informed consent was obtained for all subjects. No organs/tissues were procured from prisoners and the organs were procured by Gift of Hope (https://www.giftofhope.org/) and the transplants were performed at the Comprehensive Transplant Center at Northwestern University. Patients with dual organ transplants or a panel reactive antibody (PRA) > 20% were excluded. Additional exclusion criteria included pregnancy, severe hyperlipidemia, history of FSGS and cytopenias. The clinical protocol and patient follow-up was completed on February 7, 2018.

### Objectives

Forty patients were randomized 1:1 at the time of transplant to IS with low dose TAC and EVR (n = 20) or standard dose TAC and MMF (n = 20) (**Fig 1**). We had calculated our sample power for the two interrelated primary endpoints based on our clinical experience to obtain biopsy, cell sub-poulations and genomic data in a small pilot study. Using a two-tailed α of 0.05, the anticipated effect sizes corresponding to the specific aim between the two treatment arms were calculated using a minimum of 80% statistical power and assumed 10% attrition at 12 months. All patients, except a recipient >65 years old, received Alemtuzumab induction as it is standard of care (SOC) at our center. Maintenance IS was steroid free unless indicated by the following medical conditions: acute renal allograft rejection, renal diseases that might require the use of steroids and other systemic diseases such as RA, SLE, or asthma. Everolimus levels were maintained between 3–8 ng/ml. In the standard IS group TAC levels were maintained between 8–10 ng/ml up to 2 months, 6–8 ng/ml from 2–6 months and 4–8 ng/ml after 6 months post-transplant. TAC levels in the low dose group were maintained between 4–7 ng/ml up to 2 months, 3–5 ng/ml from 3–6 months and 2–5 ng/ml after 6 months post-transplant, reflecting a 50% lower dose than the standard IS.

### Outcomes

The primary endpoints of the study were: 1) to evaluate the impact of the two maintenance immunosuppressive regimens on subpopulation of T cells including regulatory T cells and other T cell subpopulations at different time points post renal transplant, 2) to determine the impact of the two maintenance immunosuppressive regimens on renal allograft function at 12 months post randomization (post-transplant). Secondary endpoints of the study included: 1) to evaluate the impact of the two maintenance immunosuppressive regimens on allograft immunohistopathology and gene expression profiles at 3 and 12 months post-transplant in renal allograft biopsies, 2) to evaluate the impact of the two maintenance immunosuppressive regimens on acute rejection, graft loss and death at 12 months post-transplant.

### Follow up

Patients were followed for 2 years post- transplant. Baseline and follow up clinical data was obtained using the transplant center Electronic Medical Record. In addition to standard of care clinic visits and blood tests, follow up also included protocol kidney biopsies (done at 3 and 12 months post-transplant) and analyses of T cell populations in peripheral blood samples at baseline, 3 and 12 months post-transplant. All allograft biopsies were independently read by our renal pathologist using the Banff classification for renal allograft pathology [12, 13]. Rejection episodes were determined based on pathological evaluation, presence of DSA and clinical judgement. Borderline change was not considered as a Rejection episode for the purposes of this study.

### Mechanistic data

#### Sample collection

Blood samples were obtained from all renal transplant recipients prior to randomization for characterization of the cell subpopulations by flow cytometry. Additional blood samples were collected at 3, 6 and 12 months post-randomization. Peripheral blood mononuclear cells (PBMC) were obtained by Ficoll-Hypaque gradient centrifugation and were stored in liquid nitrogen. Serum was also collected and stored at -80 C. Kidney biopsy samples were collected with standard-of-care samples at the time of transplant, 3 months post-transplant and 12 months post-transplant.

#### Phenotypic characterization of regulatory T cell subsets

T cell subpopulations from peripheral blood samples were identified at randomization (baseline, pre-transplant), at 3 months and at 12 months post-transplant by multicolor flow cytometry and were analyzed as described previously[14, 15]. For T cell subtype analysis, the PBMC were stained with CD4-Alexa-PE 700 (Invitrogen, Carlsbad, CA), CD8-APC-Cy7 (BD Biosciences, San Jose, CA), CD28-FITC (eBioscience, San Diego, CA), CD45RA-PE-Cy7 (BD Biosciences, San Jose, CA), CD45RO-PerCP-Cy5.5 (BioLegend, San Diego, CA)

For intracellular cytokine and transcriptional factor staining, the cryopreserved PBMC were thawed and stimulated with 20ng/ml PMA and 500 ng/ml ionomycin for 6 hours in presence of GolgiStop (BD Biosciences) during the last 4 hours of incubation to prevent cytokine secretion. The stimulated PBMC were then labeled with CD4-v500 (BD Biosciences, San Jose, CA), CD25-Alexa-700 (Biolegend, San Diego, CA), and CD45RO-PerCP Cy5.5 (Biolegend, San Diego, CA). After incubation and washing, the cells were fixed and permeabilized and then incubated with FOXP3-PE (eBioscience, San Diego, CA), RORγt-APC (eBioscience, San Diego, CA), IFN-γ-FITC (BD Biosciences, San Jose, CA), IL-17-PE-Cy7 (BD Biosciences, San Jose, CA). We analyzed the percentage of cells by LSRFortessa flow cytometer (BD Biosciences, San Jose, CA). Regulatory T cells were identified as CD4^+^CD25^hi^FoxP3^+^ cells. A total of 50,000 events were recorded per sample and the data were analyzed by FlowJo software v.10 (Tree Star, Inc. Ashland, OR).

#### RNA isolation from kidney biopsy samples

Graft biopsy samples at 3 and 12 months post-randomization were collected in RNAlater reagent (Ambion Inc., Austin, TX) and stored at -80°C until use. Total RNA was isolated using Trizol (Life Technologies, Carlsbad, CA) following the guidelines and recommendations in the Affymetrix GeneChip Expression Analysis Manual (Affymetrix, Santa Clara, CA). RNA quality control was evaluated following previous established parameters for microarray hybridization.[16]

#### Gene expression microarray hybridization and analysis

Total RNA were reverse transcribed and used for *in vitro* transcription to generate labeled cDNA using Affymetrix 3' IVT Express Kit (Santa Clara, CA, USA) following manufacturer protocol and recommendations. Affymetrix HG-U133A v2.0 GeneChip microarrays for gene expression (n = 31) were hybridized and scanned on an Affymetrix GeneChip Scanner 3000 G7. Quality control and normalization were performed as reported previously.[17] Probe sets raw intensities were stored in electronic files (.DAT and .CEL formats) by the GeneChip Operating Software (GCOS). Statistical analyses were performed over all probesets (n = 22,277) on each GeneChip microarray including control probesets to discard significant differences. A two-sample *t-*test was fit for TAC *vs*. EVR comparison in the R programming environment.[18] A *p*-value <0.05 was considered significant for differentially expressed genes. Differential gene expression was illustrated using fold-changes.

#### Interaction networks, functional analysis, and upstream regulators

Gene ontology analyses were performed using Ingenuity Pathways Analysis (IPA; www.ingenuity.com). Spreadsheet lists containing probeset IDs, Gene IDs, and fold-changes were uploaded to IPA. For these analyses, *p-*values <0.05 were considered significant. Molecular pathway activity was interpreted using activation z-score (z) generated by IPA. Briefly, z-score estimates the behavior and relationship among several scores to the calculated mean. Zero z-score value indicates similar statistical behavior while positive or negative values indicate shifted trend to activation or inhibition, respectively.

#### Statistical methods

All analyses were conducted on the intent-to treat population. We employed this approach in order to maintain the integrity of randomization, and hence reduce confounding due to non-random loss to follow up. Additionally, intent to treat analysis aligns more with real world practice where the effect of ordering the treatment drug is analyzed as opposed to only the effect of actual drug use. Quantitative data was expressed as mean with standard deviation or median with interquartile range. Qualitative data was expressed in percent frequency. Continuous variables were compared using paired Students T-test while categorical variables were compared using the χ^2^ or Fischer exact test. Survival curves were plotted using the Kaplan Meier- method and the log-rank test used to compare groups. All significance tests were 2 tailed and a p value <0.05 was considered significant. Statistical analyses were conducted using JMP v. 11.0 (SAS Institute Inc, NC).

## Results

Baseline demographic and clinical characteristics of the 40 study patients are shown in **Table 1**. The mean age at transplant was 48.3 ± 16 and 48.4 ± 13 years in the TAC+EVR and TAC+MMF group respectively (p = 0.42). Both groups had a higher proportion of males, while the TAC+MMF group had a higher proportion of Caucasian patients. About a third of the patients in each group had pre-emptive transplants i.e. they had never been on dialysis. There was a higher proportion of patients who were diabetic in the TAC+MMF group when compared to the TAC+EVR group. All patients in the TAC+MMF group and all except one patient in the TAC+EVR group received Alemtuzumab induction. Other relevant baseline characteristics including BMI and HLA match were statistically similar.

**Table 1:** Baseline characteristics.

	TAC+EVR (%)	TAC+MMF (%)
N	20	20
Age	48.3 (16)	48.4(13)
Gender (% male)	13 (65)	16 (80)
Race (% Caucasian)	9 (45)	13 (65)
Pre-emptive transplant	6 (30)	7 (35)
Cause of ESRD		
Hypertension	6 (30)	5 (25)
Polycystic kidney disease	3 (15)	1 (5)
Lupus nephritis	1 (5)	1 (5)
Other/Unknown	5 (25)	5 (25)
Diabetics	5 (25)	10 (50)
Coronary artery disease	2 (10)	2 (10)
BMI	24.9 (5)	28.9(6)
PRA- Class I (Mean ± SD)	1.4 ± 3.1	3.3 ± 10.6
PRA- Class II (Mean ± SD)	2.2 ± 8.3	3.8 ± 3.1
Crossmatch	Negative	Negative
HLA match	2 (1)	2.2(1)
Alemtuzumab Induction	19 (95)	20 (100)

### Clinical results

**Table 2**shows some of the results. Mean follow up was 14±4 and 17±5 months in the TAC+EVR and TAC+MMF group respectively (p = 0.02). The cumulative mean TAC levels were 4.5 ± 1.9 and 6.4 ± 1.5 ng/ml (p = 0.03) in the TAC+EVR and TAC+MMF group respectively. Graft and patient survival were at 100% in both groups. However none of the patients in the TAC+EVR group had rejection during study follow up, compared with 4 rejection episodes documented in the TAC+MMF group. All rejection episodes, including three antibody-mediated rejection (AMR) and one combined AMR with acute cellular rejection (ACR), were treated with a variable combination of corticosteroids, plasmapheresis, IVIG, Rituximab, Bortezomib and an escalation of immunosuppression. Development of denovo DSA without overt rejection was seen in 1 patient in each group. Similarly, development of proteinuria was seen in 2 patients in each group. A longitudinal anlalysis revealed that rejection rate was lower in the TAC+EVR group compared to the TAC+MMF group (**Fig 2**). However eGFR remained similar between the two groups at 3,6,12 and 18 months post-transplant (**Fig 3**). Incidence of adverse events, including opportunistic infections, hyperlipidemia and neutropenia was similar between the two groups (**Table 2**).

**Fig 2:**
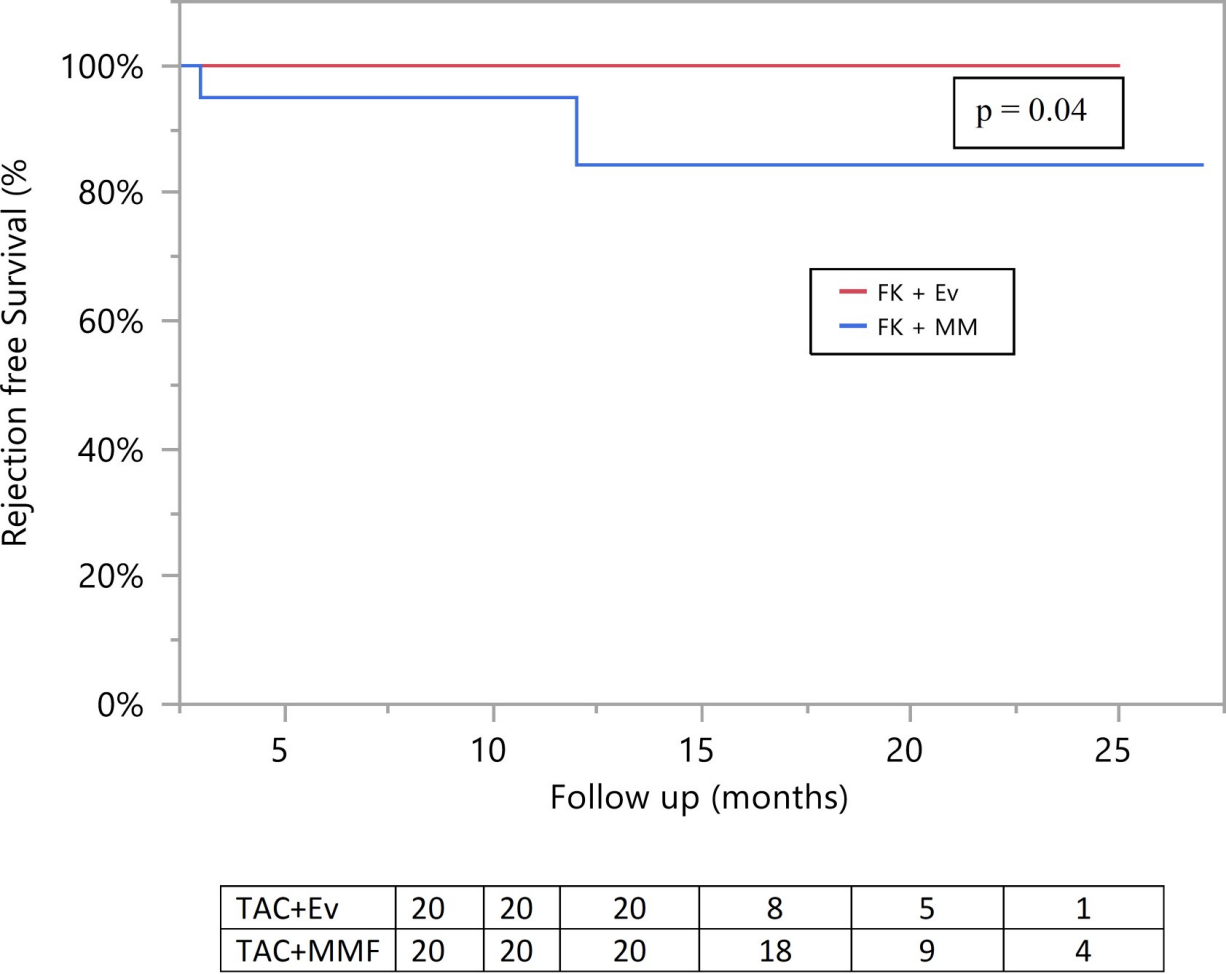
Comparison of biopsy proven rejection-rates between study groups.

**Fig 3:**
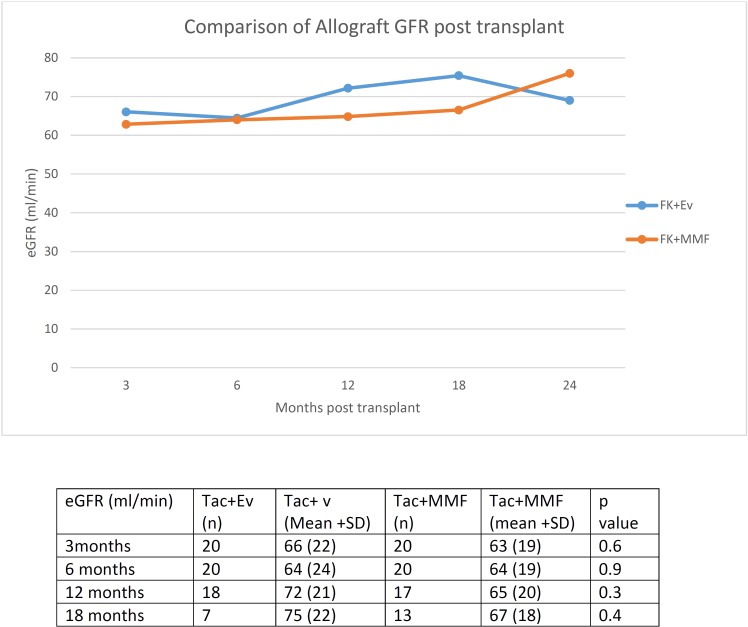
Comparison of post-transplant estimated GFR between study groups.

**Table 2:** Clinical results.

Results	TAC+EVR	TAC+MMF	p value
	n = 20	n = 20	
Mean Follow up (months)*	14 ± 4	17 ± 5	0.02*
Lost to follow up	0	1	
Graft Survival (percent)	100	100	1.00
Tacrolimus level	4.5 ± 1.9	6.4 ± 1.5	0.03
Rejection episodes	0	4	0.03*
Development of Denovo DSA without overt rejection	1	1	1.00
Proteinuria > 1g/day	2	2	1.00
Adverse Events			
Hypertriglyceridemia	3	1	0.33
BK nephropathy	0	1	1.00
Neutropenia	0	1	1.00
Other Infections**	5	3	0.44

* Results expressed as mean ± standard deviation. Please see Table A in S4 File for complete information.

** Other infections included bacteremia, clostridium difficile colitis, abdominal abscess and herpes zoster

### Histopathological results

**Table 3**depicts a comparison of allograft histopathology between the 2 groups seen on 1 year protocol renal allograft biopsies. The presence of features suggestive of CNI nephrotoxicity, including interstitial fibrosis and tubular atrophy (IFTA), isometric vacuolization and arteriolar hyalinosis was similar between the two groups.

**Table 3:** Results– 12 month histopathology data.

Pathology	TAC+EVR (n = 19)*	TAC+MMF (n = 16)*
**Glomerusclerosis:-Global (GS) and Segmental (SS)**	GS: 8 (42%)	GS: 5 (33%)
	SS: 5 (26%)	SS: 4 (27%)
**Acute glomerulitis (g)**	g1: 0 (0%)	g1: 2 (13%)
	g2: 0 (0%)	g2: 0 (0%)
	g3: 0 (0%)	g3: 0 (0%)
**Tubulitis NOT in IFTA (>t)**	t1: 1 (5%)	t1: 2 (13%)
	t2: 2 (11%)	t2: 1 (6%)
	t3: 0 (0%)	t3: 0 (0%)
**Acute Tubular Necrosis (ATN)/Isometric Tubular vacuolization**	Mild: 14 (74%)	Mild: 9 (56%)
	Moderate: 5 (26%)	Moderate: 1 (6%)
	Severe: 0 (0%)	Severe: 0 (0%)
	*Isometric*: *1 (5%)*	
**Interstitial Inflammation (i)**	i1: 1 (5%)	i1: 2 (12%)
	i2: 0 (0%)	i2: 0 (0%)
	i3: 0 (0%)	i3: 0 (0%)
**Arteriolar Hyalinosis (ah)**	ah1: 1 (5%)	ah1: 1 (6%)
	ah2: 0 (0%)	ah2: 0 (0%)
	ah3: 0 (0%)	ah3: 0 (0%)
**Arteriosclerosis (cv)**	cv1: 5 (26%)	cv1: 4 (25%)
	cv2: 1 (5%)	cv2: 0 (0%)
	cv3: 0 (0%)	cv3: 0 (0%)
**Peritubular capillaritis (PTC)**	ptc1: 0 (0%)	ptc1: 3 19%)
	ptc2: 0 (0%)	ptc:2: 2 (13%)
	ptc3: 0 (0%)	ptc:3: 0 (0%)
Interstitial fibrosis and Tubular atrophy (IFTA)**	Mild: 12 (63%)	Mild: 11 (70%)
	Moderate: 1 (5%)	Moderate: 1 (6%)
	Severe: 0 (0%)	Severe: 0 (0%)
**Inflammation associated with IFTA**	Minimal: 4 (21%)	Minimal: 1 (6%)
	Mild: 5 (26%)	Mild: 4 (25%)
	Moderate: 3 (16%)	Moderate: 3(20%)
	Severe: 0 (0%)	Severe: 0 (0%)
**Tubulitis of intact tubules within/interface (t)**	t1: 4 (21%)	t1: 3 (20%)
	t2: 2 (11%)	t2: 1 (6%)
	t3: 0 (0%)	t3: 0 (0%)
**Transplant glomerulopathy (cg)**	cg1: 0 (0%)	cg1:0 (0%)
	cg2: 0 (0%)	cg2:0 (0%)
	cg3: 0(0%)	cg3: 0 (0%)
**C4d staining on immunofluorescence**	C4d1: 0 (0%)	C4d1: 0 (0%)
	C4d2: 0 (0%)	C4d2: 0 (0%)
	C4d3: 0 (0%)	C4d3: 4(25%)
**OTHER FINDINGS/DIAGNOSIS**	Borderline change:2 (12%)	Borderline change: 2/15 (13%)
	Subcapsular injury: 5 (30%)	Subcapsular injury: 2/15 (13%)
		Polyoma Virus: 1/15 (7%)
		AMR: 4/15 (27%)

* All values expressed as n (%). There was no significant difference between the 2 study groups. All pathological definitions are per Banff criteria. Please see **Table B** in S4 Data file for complete information.

** IFTA0 and IFTA1 combined in one group.

### Mechanistic data

#### TAC+EVR leads to expansion of regulatory T cells without affecting other T cell subpopulations

**Fig 4:**
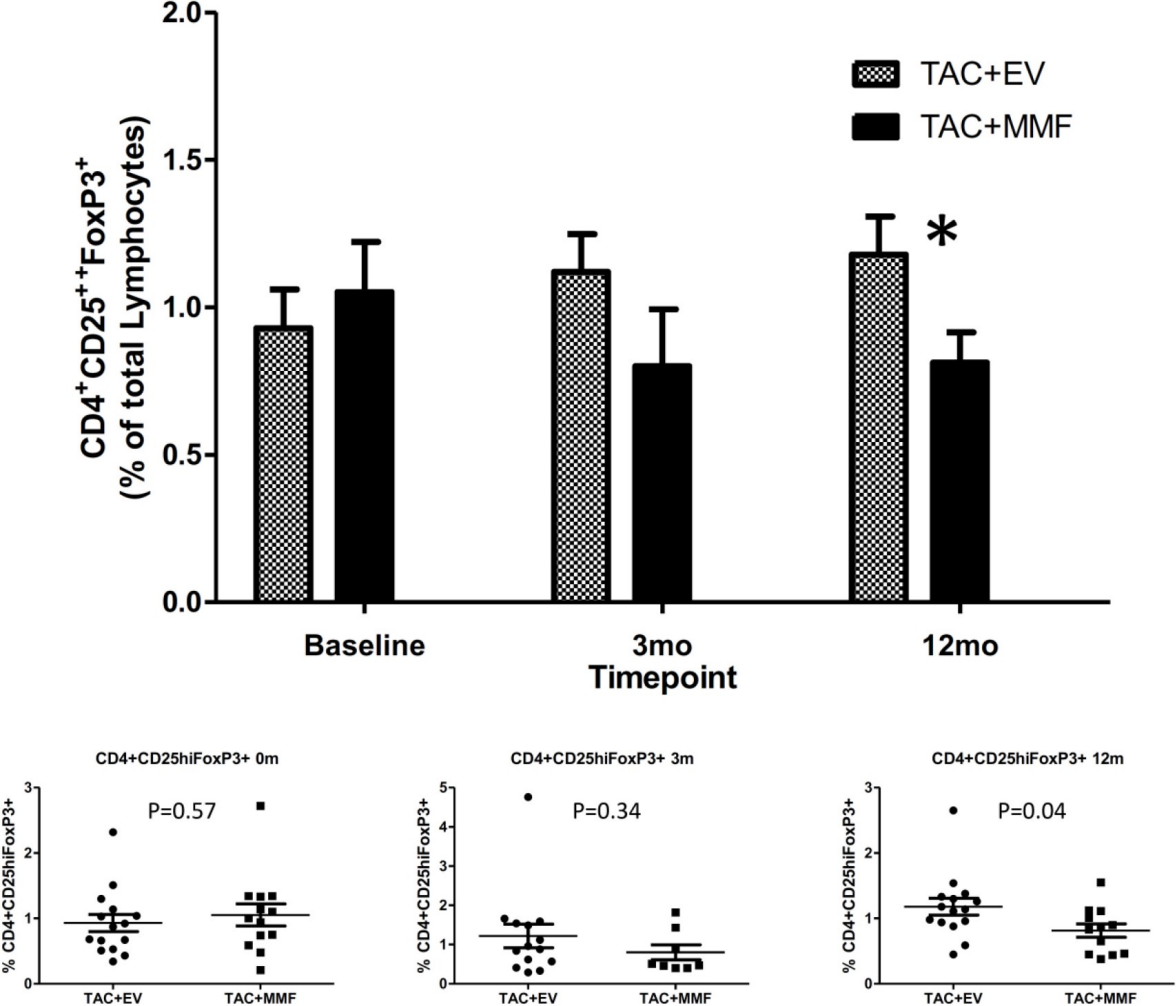
Percentage of CD4^+^CD25^hi^FOXP3^+^ regulatory T cells in the peripheral blood between study groups. Flow cytometric analyses were performed as described in Materials and methods. The number of subjects analyzed at—Baseline: TAC+EVR N = 15,TAC+MMF N = 13; 3 mo: TAC+EVR N = 14,TAC+MMF N = 9; 12 mo: TAC+EVR N = 15,TAC+MMF N = 12. Please see **Table C** in S5 File for complete information.

Frequency of CD4 T cells, CD8 T cells, CD4^+^CD25^-^ T cells, naïve CD4 T cells (CD4^+^CD45RA^+^), memory CD4 T cells (CD4^+^CD45RO^+^), naïve CD8 T cells (CD8^+^CD45RA^+^), memory CD8 T cells (CD8^+^CD45RO^+^), and regulatory T cells (CD4^+^CD25^hi^Foxp3^+^) in peripheral blood were analyzed by flow cytometry. Changes in T cells subpopulations and regulatory T cells were compared over time from baseline (pre-transplant) to 3 months and 12 months post-transplant within each group and between TAC+EVR and TAC+MMF groups. The frequencies of CD4 T cells, CD8 T cells, CD4^+^CD25^-^ T cells, naïve CD4 and CD8, and memory CD4 and CD8 T cells were similar between the two groups [19] However, we observed an increase in frequencies of CD4^+^CD25^hi^Foxp3^+^ regulatory T cells in the TAC+EVR group compared to the TAC+MMF group starting from 3 months post-transplant and the frequencies of CD4^+^CD25^hi^Foxp3^+^ regulatory T cells were significantly higher in the TAC+EVR group at 12 months post-transplant (**Fig 4**).

#### Co-expression of IFN-Gamma (IFN-γ) in CD4^+^CD25^hi^Foxp3^+^T cells of recipients receiving TAC+EVR

**Fig 5:**
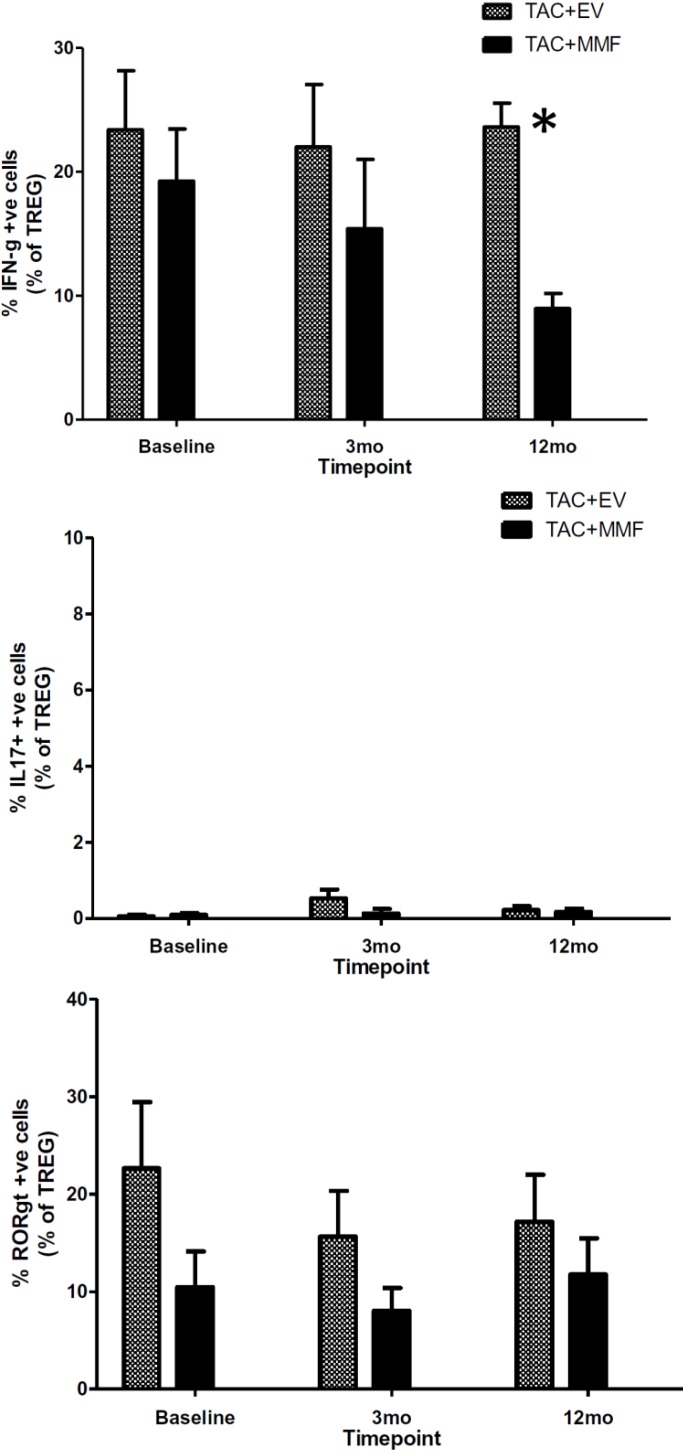
Intracellular cytokine and transcription factor staining for interferon-γ, interlekin-17 (IL-17) and RAR-related orphan receptor gamma 2 (RORγt) in CD4^+^CD25^hi^FOXP3^+^ regulatory T cells between study groups. The number of subjects analyzed at—Baseline: TAC+EVR N = 15,TAC+MMF N = 13; 3 mo: TAC+EVR N = 14,TAC+MMF N = 9; 12 mo: TAC+EVR N = 15,TAC+MMF N = 12. Please Tables D, E and F in S5 File for complete information.

We used intracellular cytokine and transcriptional factor staining to evaluate the CD4^+^CD25^hi^Foxp3^+^ T cells from both TAC+EVR and TAC+MMF group. Intracellular cytokine and transcriptional factor staining for Interferon Gamma (IFN-γ), Interleukin 17 (IL-17) and RAR-related orphan receptor gamma 2 (RORγt) showed no difference at baseline between the two groups. Interestingly, we observed a significant decline in the TAC+MMF group with stable maintenance in TAC+EVR group of percentage IFN-γ positive CD4^+^CD25^hi^Foxp3^+^ T cells at 12 months post-transplant (**Fig 5**). The frequencies of IL-17 positive CD4^+^CD25^hi^Foxp3^+^ T cells and RORγt positive T cells were similar between the two groups (Fig 5).

#### Gene expression results

**Fig 6:**
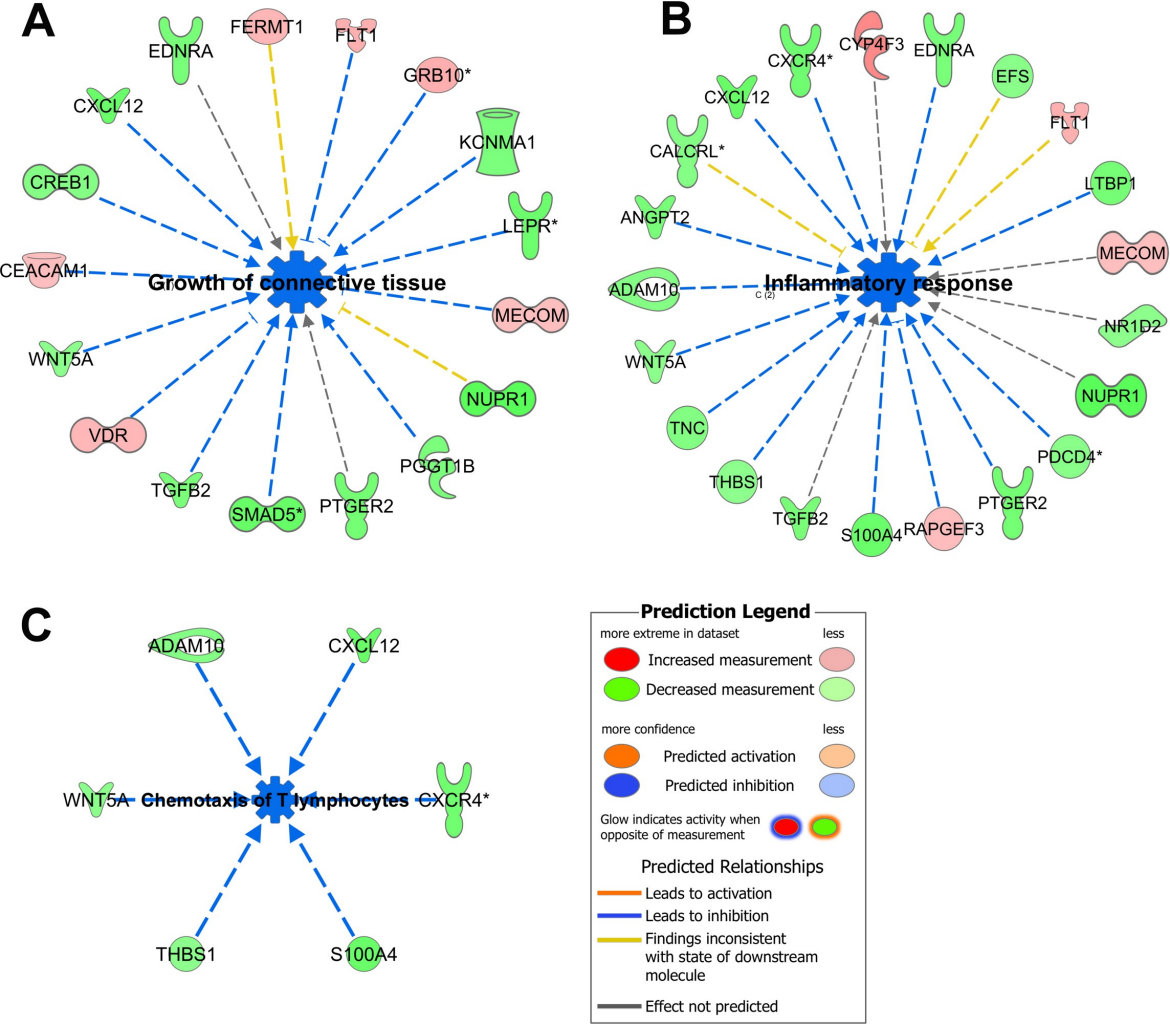
Inhibition of major functions. Functions like growth of connective tissue (**A**), inflammatory response **(B**), and chemotaxis of T lymphocytes (**C**) were predicted to be inhibited (blue) and the genes involved were mostly downregulated (green). The legend with the color code for the prediction is shown at the bottom right. Please see Table G in S5 File for complete information.

A total of 22 patients with longitudinal biopsies at 3- and 12 months post-randomization were available. Of the RNA samples isolated from 44 biopsies, 31 samples (3 mo TAC+EVR n = 7; 12 mo TAC+EVR n = 7; 3 mo TAC+MMF n = 9; 12 mo TAC+MMF n = 8) passed qualility control (QC) conducted at various stages through RNA isolation to gene expression array process and thus were used for the final analysis. Twenty eight of the 31 samples represented paired two-time point biopsy RNA samples from 14 patients (TAC+EVR n = 6; TAC+MMF n = 8) and were used for longitudinal analysis of gene expression. At 3 months post-transplant, there were no differences in the gene expression profiles between TAC+EVR and TAC+MMF groups. However, at 12 months post-transplant a total of 183 probesets were differentially expressed (60% were down regulated and 40% were upregulated in TAC+EVR group) between groups (Fold change cutoff 1.5; p-value cutoff 0.05). Pathway analysis (ingenuity.com) showed that the differentially expressed genes in TAC+EVR group were involved in inhibition of functions like inflammatory response (p-value 5.45E-05; z-score -2.488), growth of connective tissue (p-value 2.68E-04; z-score -2.187), and chemotaxis of lymphatic system cells (p-value 1.29E-04; z-score -2.543), more specifically chemotaxis of T lymphocytes (p = 1.54E-04; z-score -2.366) (**Fig 6**).

**Fig 7:**
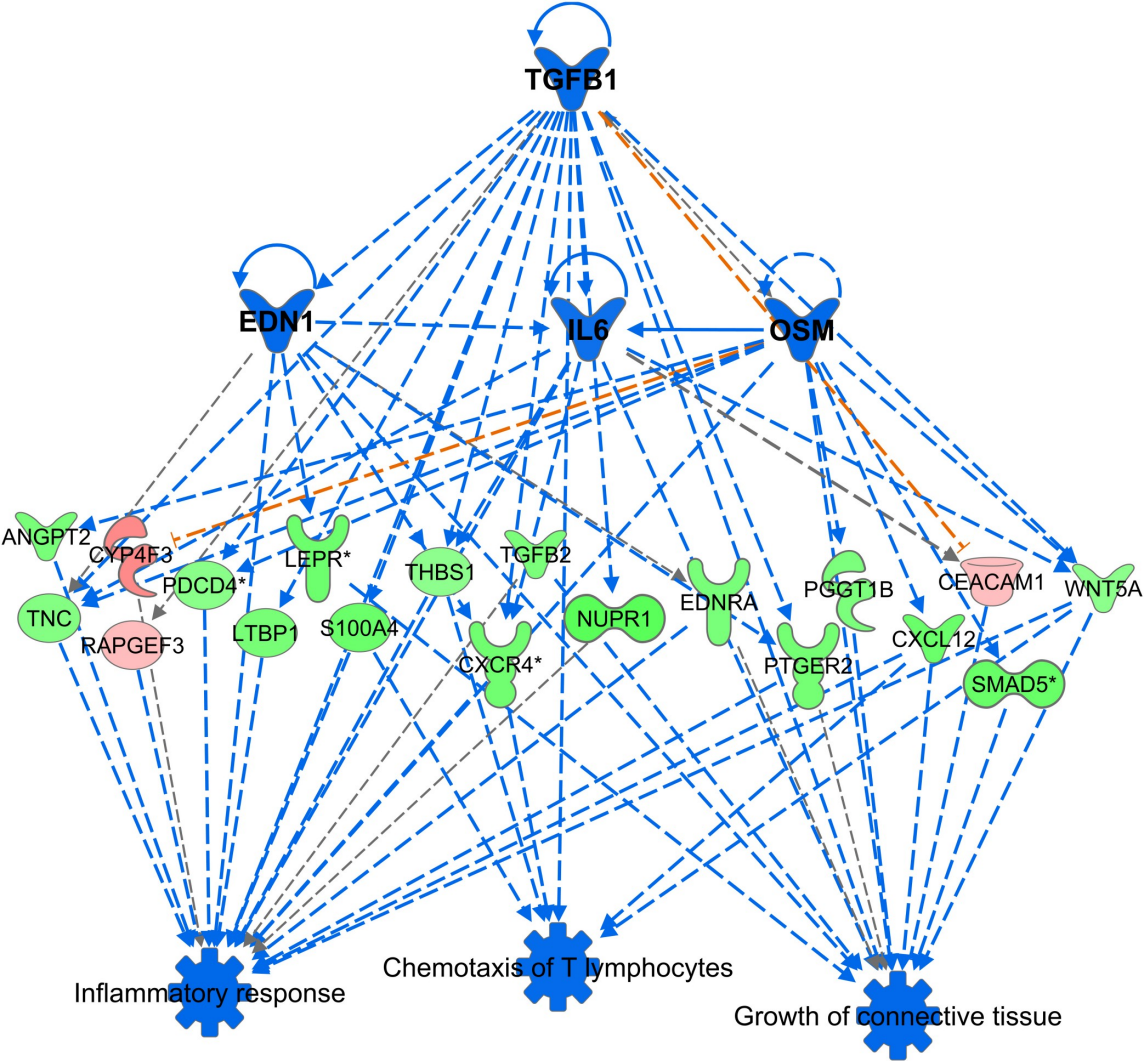
Predicted upstream regulators. Based on the differentially expressed genes, certain functions like inflammatory response, chemotaxis of T lymphocytes, and growth of connective tissue were predicted to be inhibited (blue). IPA analysis predicted that the inhibition of upstream regulators like the cytokine TGFB1, EDN1, IL-6 and OSM would lead to decrease in the functions listed in the third tier of the figure via their effect on the mediator genes that were also seen to be differentially expressed between the two groups. Please see Table H in S5 File for complete information.

The upstream regulator that was most significantly predicted to be inhibited was *TGFB1* (p-value 2.02E-08; z-score -2.325); there were others like *IL6* (p-value 3.69E-02; *z*-score -1.919), *OSM* (p-value 4.65E-04; *z*-score -2.855) and *EDN1* (p-value 1.66E-06; *z*-score -2.595) (**Fig 7**). These findings supported a trend of decreased inflammatory response, angiogenesis, and decreased connective tissue growth at 12 months post-transplant in the TAC+EVR group.

**Fig 8:**
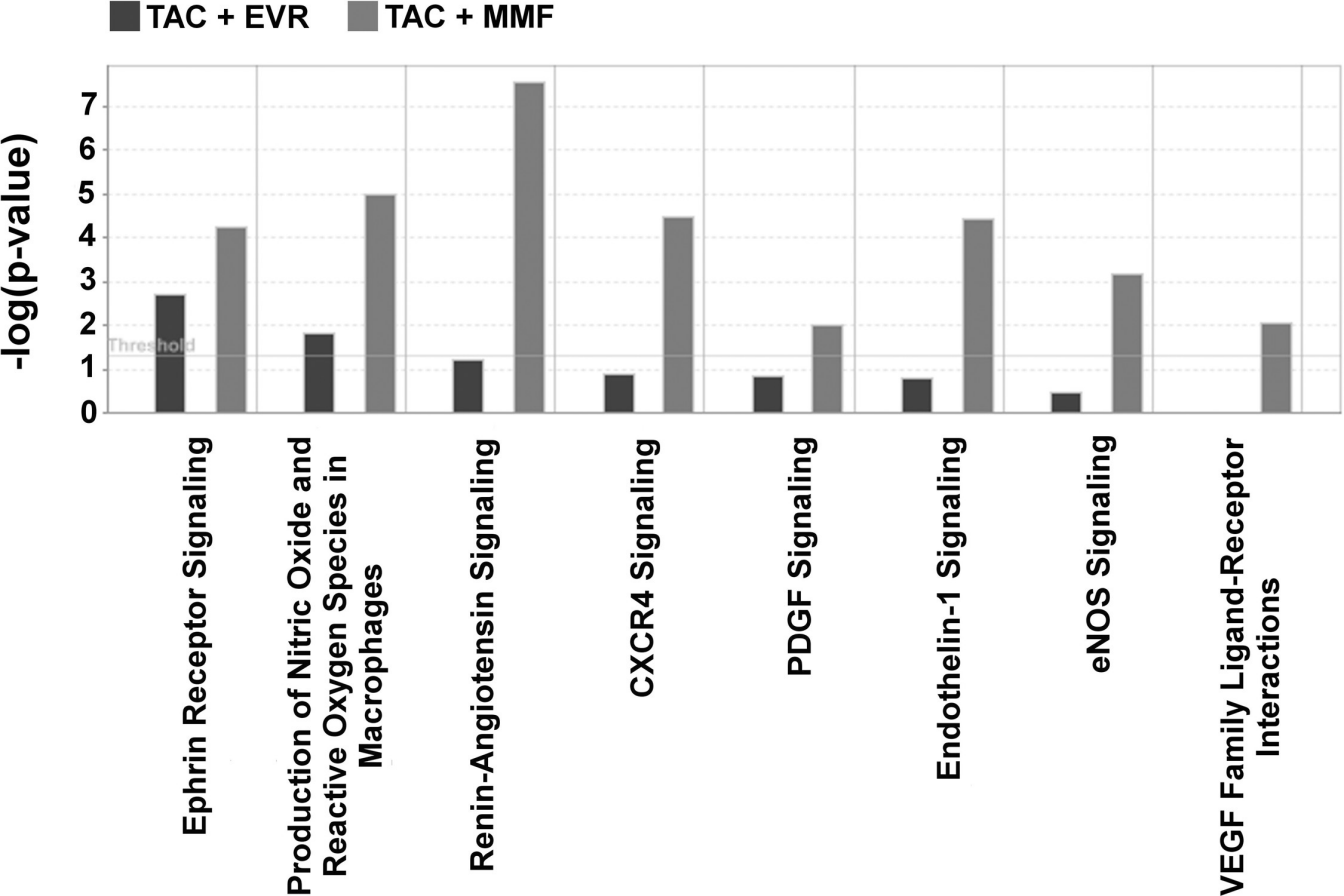
Analysis of time dependent changes in gene expression pathways in TAC+EVR and TAC+MMF groups. Based on comparison analysis of differentially expressed genes at 12 month in comparison to 3 months post-transplantation on the two studied groups, it was observed that pathways related to cell proliferation, chemotaxis and inflammation were much pronounced in TAC+MMF (grey) in comparison to TAC+EVR (black). Please see Tables J and K in S5 File for complete information.

A longitudinal analysis of gene expression profile changes in biopsies from TAC+EVR and TAC+MMF groups were also performed. Thus, when compared between 3 months and 12 months post-transplant, TAC+EVR and TAC+MMF groups differentially expressed 189 and 542 probe sets, respectively. This also showed that canonical pathways related to cell proliferation and migration/chemotaxis like renin-angiotensin signaling (**Figure A** in S1 File), ephrin receptor signaling (**Figure B** in S1 File), endothelin-1 signaling, PDGF signaling, among others, were more activated in TAC+MMF in comparison to TAC+EVR (**Fig 8**).

Using IPA, T cell related diseases and functions were compared for sequential changes in biopsies from TAC+EVR and TAC+MMF groups. It was observed that though there were common functions between the two groups, functions like chemotaxis of T cells, T cell development, T cell migration were more activated in TAC+MMF. Each group had unique set of genes related to T cell functions implying that these groups started to diverge in terms of their gene expression pattern and thereby T cell functionality (**Figure C** in S1 File).

## Discussion

Immunosuppressive protocols in kidney transplantation often utilize a combination of drugs that significantly decrease the rate of acute cellular rejection. Despite advances in surgical and medical care of kidney transplant recipients, long-term graft survival has not yet drastically improved. This is mainly due to the side effects associated with CNI therapy, such as nephrotoxicity as well as increased malignancy and cardiovascular diseases. Consequently, many strategies have been developed to minimize nephrotoxicity while maintaining efficacy of immunosuppression and improve long-term outcomes. Thus, mammalian target of rapamycin (mTOR) inhibitors such as everolimus in combination with low-dose CNI being recognized as an attractive option for providing acceptable rejection rates and better allograft function. De novo treatment with mTOR inhibitors in combination with mycophenolate has been shown to result in higher acute rejection rates [6]. Similarly, early conversion from a CNI to an mTOR inhibitor therapy in selected patients provided only equal or slightly better GFR with some increase in acute rejection rate [7].

From our previous report, conversion from TAC to Sirolimus at 12 months post-transplantation is not associated with increased rates of acute rejection and graft loss. Nevertheless, despite CNI elimination, renal allograft function is equally maintained in both groups [20]. We also demonstrated that Sirolimus conversion led to an increase in CD4^+^CD25^hi^Foxp3^+^ regulatory T cells (Treg) overtime after conversion. Despite the expansion of Treg, we found some evidences of chronic immune alterations after sirolimus conversion [21]. There was an increased indirect alloreactive T-cell frequencies measured by IFN-γ ELISPOT. At the molecular level, there was an activation of the antigen presentation, IL-12 signaling, oxidative stress, macrophage-derived production pathways, and increased inflammatory and immune responses. However, data from an *in vitro* study indicated that combination of an mTOR inhibitor with low dose CNI promotes the differentiation and expansion of donor-specific Tregs without secondary reprogramming to IFN-γFOXP3 and IL-17FOXP3 Treg subsets [22]. Furthermore, mTOR inhibitor was shown to have potent B cell inhibitory effects in-vitro [23] which might help control the humoral immune response to the allograft.

In contrast to our previous report that demonstrated an unfavorable chronic immune alteration in Sirolimus-treated recipients, we found more encouraging results with Everolimus. Therefore, in this study, we used a combination of low dose TAC+EVR aiming to achieve the immunomodulatory effects of both the mTOR inhibitor and the Calcineurin inhibitor. The study included only living donors so that the complicating effects of cold ischemia time could be avoided. The one-year results are quite promising with lower rejection-rate in the TAC+EVR group. An independent review of biopsies showed that there was a trend towards increased pathological features of immunological activity in the TAC+MMF group compared to the TAC+EVR group, but these features didn’t reach statistical significance when analyzed individually. However, a longer follow up is needed to see if this translates into better long term graft function. As expected, we found an expansion of CD4^+^CD25^hi^Foxp3^+^ regulatory T cell subset in the TAC+EVR group compared to the TAC+MMF group as early as 3 months post-transplant and the expansion continued to persist at 12 months post-transplant. Interestingly, we found a significantly higher percentage of IFN-γ positive CD4^+^CD25^hi^Foxp3^+^ T cells in the TAC+EVR group compared to the TAC+MMF group at 12 months post-transplant. It is increasing being recognized that IFN-gamma plays a major role in both induction and functioning of regulatory T cells [24]. Sawitzki et al. reported that IFN-γ production was important for the regulatory functions of mouse alloantigen-reactive regulatory T cells in vivo [25]. In human, it was observed that kidney transplant recipients with good graft function and a serum creatinine of ≤ 1.8 mg/dl for more than 100 days (mean 5 years) had more CD4^+^CD25^+^Foxp3^+^IFNγ^+^ T cells in the peripheral blood than recipients with impaired graft function and a serum creatinine of ≥ 2.0 mg/dl [26]. These reports suggest that CD4^+^CD25^+^Foxp3^+^IFNγ^+^ T cells may be involved in the prevention of acute rejection early in mice and the maintenance of good graft function long-term in the human.

Although Everolimus has a similar structure to Sirolimus, the additional hydroxyethyl group at the C(40) of the Everolimus molecule results in different pharmacokinetic and pharmacodynamics properties such as tissue and subcellular distribution, different affinities to active drug transporters and drug-metabolizing enzymes as well as differences in drug-target protein interactions including a much higher potency in terms of interacting with the mTOR complex 2 than Sirolimus [27]. Previous reports in animals comparing both mTOR inhibitors showed that Sirolimus might enhance calcineurin inhibitors nephrotoxicity while Everolimus has less negative effects [28]. Ex vivo study also showed that Everolimus had a more favorable effect on vascular endothelial function than Sirolimus [29] and Everolimus was more effective than Sirolimus in reducing the inflammatory cytokine IL-8 and VEGF release as well as increasing the release of the anti-inflammatory cytokine IL-1RA [30]. Similarly, in the *in vitro* Treg-MLR assay, EVL was found to be more potent and consistent immunoregulatory agent and less dependent on concentrations than Sirolimus [31]. In this study, the comparison of gene expression profile in kidney tissue between TAC+EVR and TAC+MMF also supported the previous data with the findings of a decreased inflammatory response, angiogenesis, and a decreased connective tissue growth at 12 months post-transplant in the TAC+EVR group.

Recently, a large multi-center prospective study of EVR with reduced CNI exposure demonstrated non-inferiority compared to MMF with standard-exposure of CNI [32]. In contrast to our study, all subjects were on triple immunosuppression including steroids and majority received IL-2R blocker induction. Further, no mechanistic studies were performed. The limitations of our study include a small sample size and only 12 months of follow-up. Despite the short-term follow up, however, we were able to demonstrate promising results in rejection rate and the mechanistic data and gene expression profile favoring the combination of Everolimus with low dose Tacrolimus over the conventional regimen. Longer-term data are needed to confirm these encouraging results. Also for the future, use of single cell mRNA sequencing technique, when it is better developed and validated, may provide more pathological and physiological information affecting the graft.

## Supporting information

S1 File Containing Figure A, Figure B and Figure C. (DOCX)

S2 File Clinical trial protocol. (PDF)

S3 File Consort checklist. (DOCX)

S4 File Table A (clinical data) and Table B (histopathological data). (PDF)

S5 File Tables C, D, E, F, G, H, J and K for Figs 4–8. (PDF)

## Abbreviations

ACR: Acute cellular rejection
AMR: Antibody-mediated rejection
BMI: Body mass index
CNI: Calcineurin inhibitors
CsA: Cyclosporine-A
DSA: Donor-specific antibodies
ELISPOT: Enzyme-linked ImmunoSpot
EVR: Everolimus
FSGS: Focal segmental glomerulosclerosis
HLA: human leukocyte antigen
IFN-γ: Interferon Gamma
IL-17: Interleukin 17
IFTA: interstitial fibrosis and tubular atrophy
IRB: Institutional Review Board
IS: Immunosuppression, immunosuppressive drugs
KT: kidney transplantation
MMF: Mycophenolate
PBMC: Peripheral blood mononuclear cells
PMA: Phorbol 12-myristate 13-acetate
RA: Rheumatoid Arthritis
SLE: Systemic lupus erythematosus
TAC: Tacrolimus
Tregs: CD4^+^CD25^hi^Foxp3^+^ regulatory T cells
