# Supplementary material for: Mechanistic analyses in kidney transplant recipients prospectively randomized to two steroid free regimen—Low dose Tacrolimus with Everolimus versus standard dose Tacrolimus with Mycophenolate Mofetil
Source: PLoS One. 2019 May 28;14(5):e0216300. doi: 10.1371/journal.pone.0216300 (PMC6538151; doi:10.1371/journal.pone.0216300)
Supplement: S1 File — (DOCX) [file pone.0216300.s001.docx]

**Supplementary Figures**


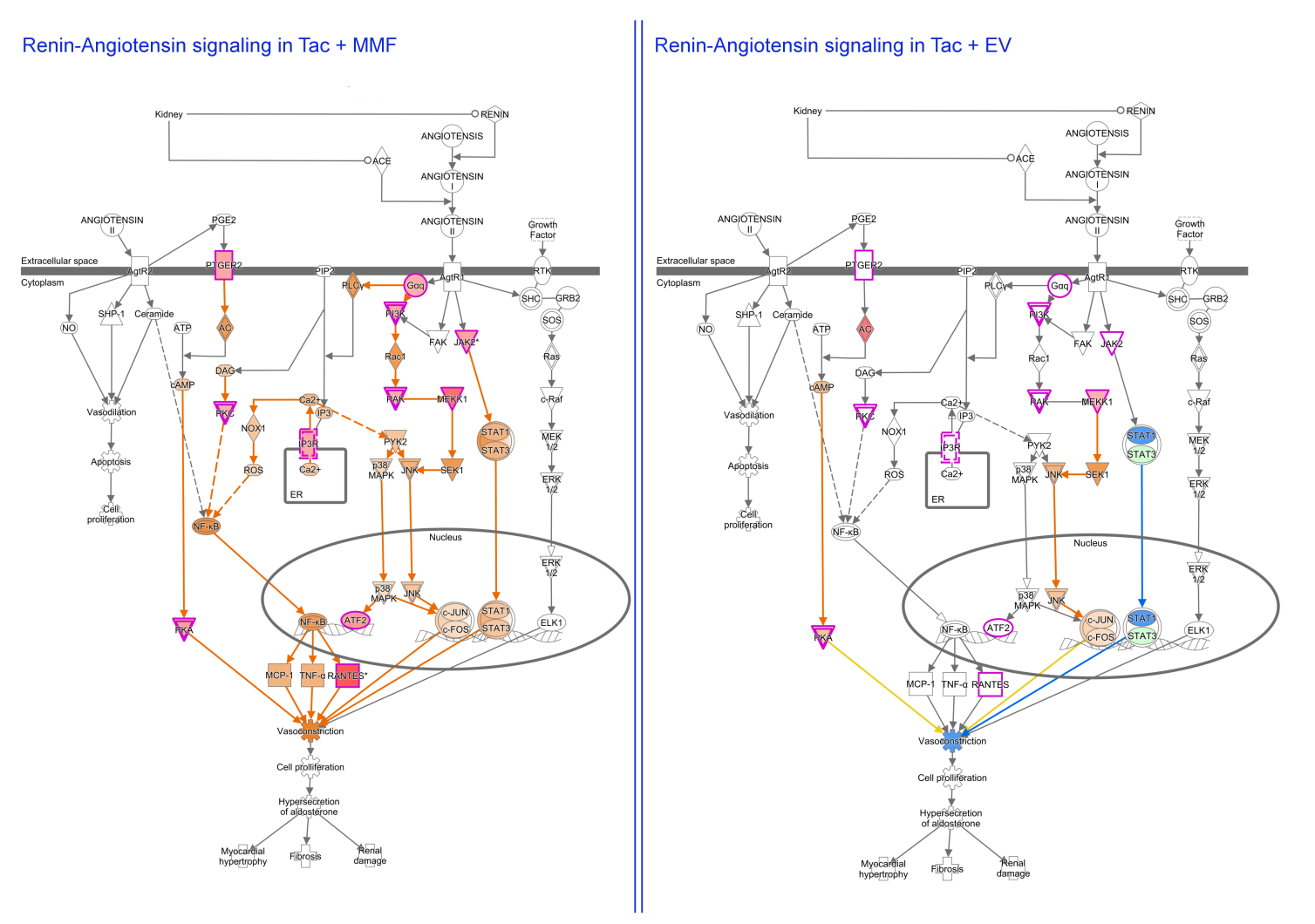


***Figure A*: Comparison of Renin-Angiotensin signaling in TAC+MMF and TAC+EVR groups.** Renin-angiotensin signaling is more activated with time (12 months in comparison to 3 months) in TAC+MMF in comparison to TAC+EVR. More number of genes are differentially expressed and upregulated (filled in red) and thus functions like vasoconstriction are predicted to be activated (filled in orange) in TAC+MMF in comparison to TAC+EVR where the functions are predicted to be inhibited (filled in blue).


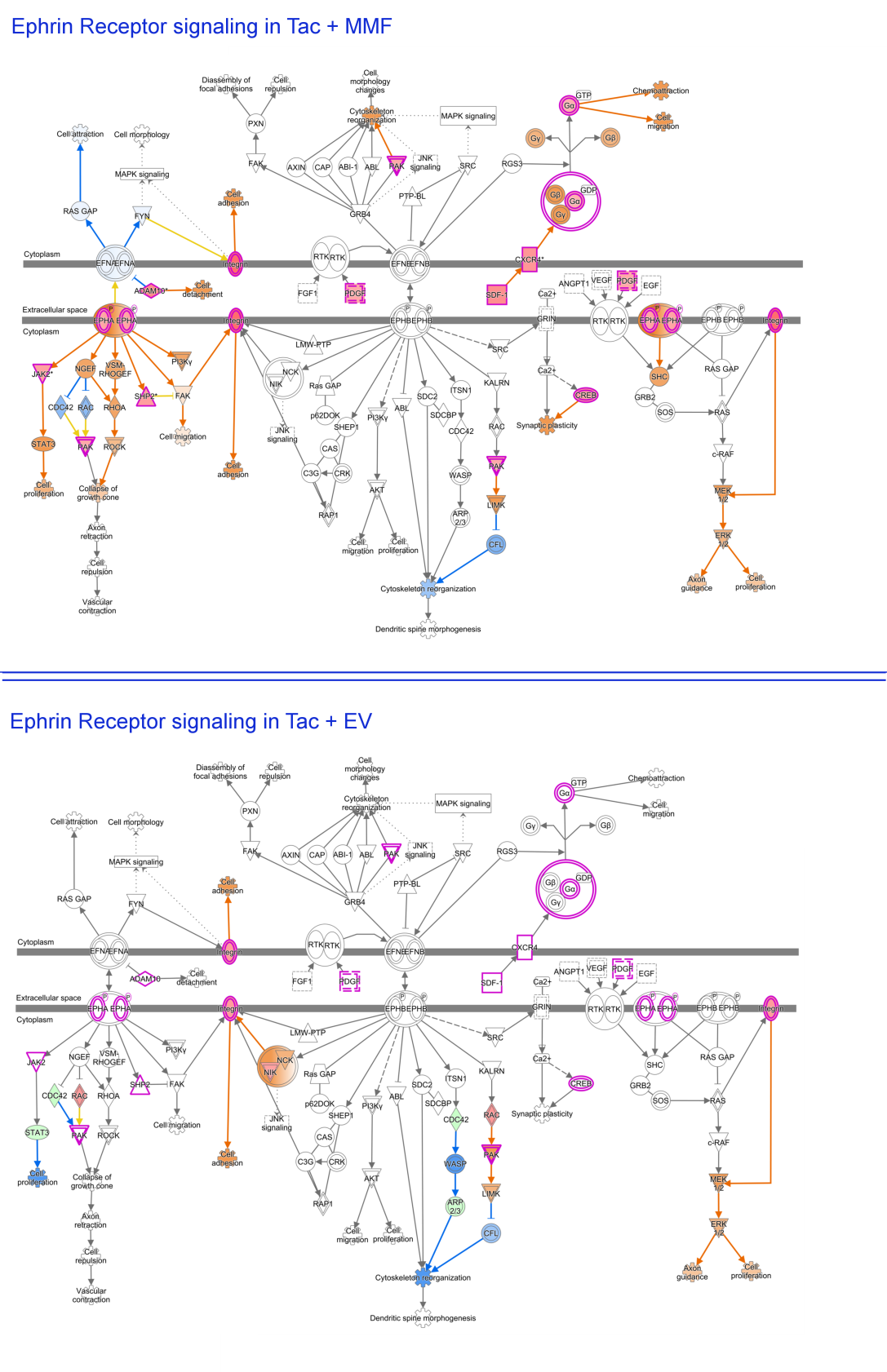


***Figure B*: Comparison of Ephrin Receptor signaling in TAC+MMF and TAC+EVR groups.** Ephrin Receptor signaling is more activated with time (12 months in comparison to 3 months) in TAC+MMF in comparison to TAC+EVR. More number of genes are differentially expressed and upregulated (filled in red) and thus functions like cell proliferation chemo attraction, cell migration and collapse of growth cone are predicted to be activated (filled in orange) in TAC+MMF in comparison to TAC+EVR where the functions are predicted to be inhibited (filled in blue) or not affected (no color).


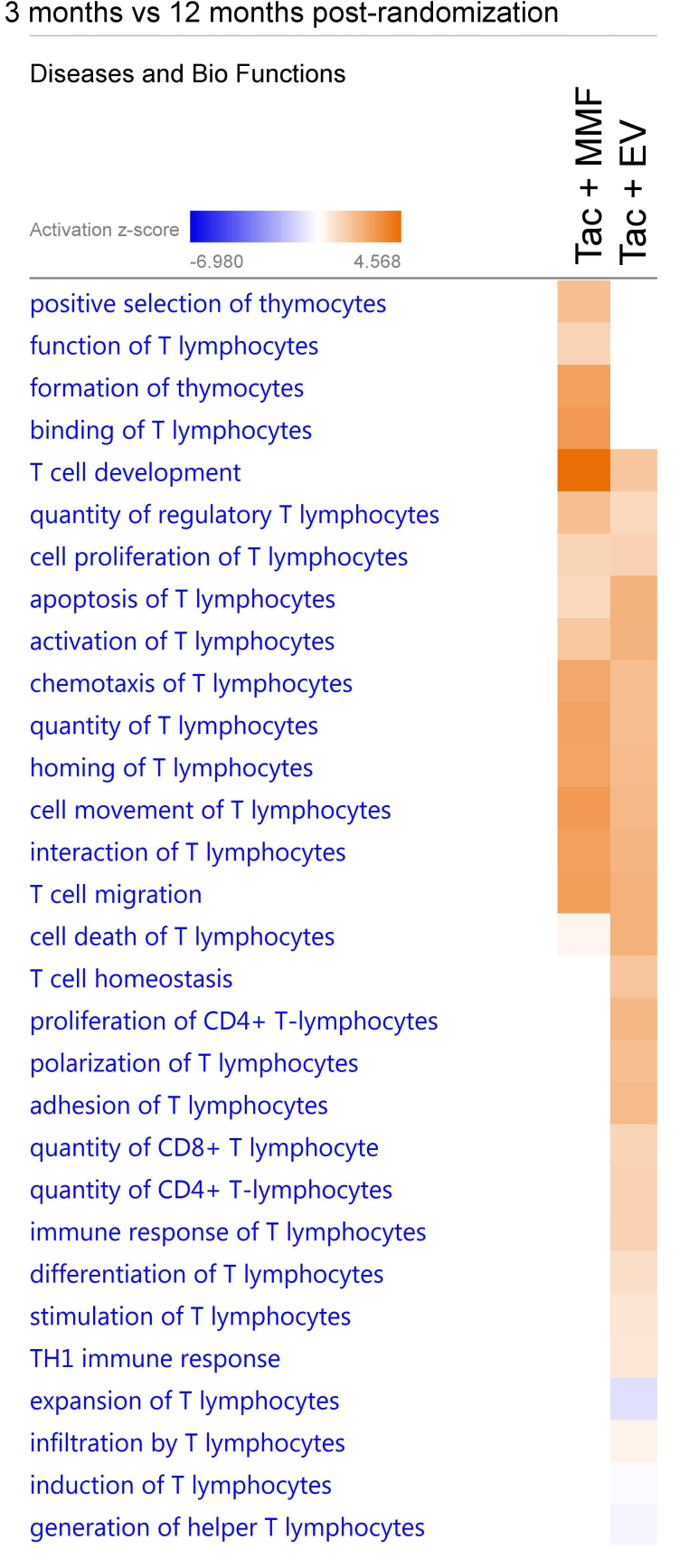


***Figure C:* Comparison analysis of longitudinal changes (3- Vs 12 months) in T cell related functions in TAC+MMF and TAC+EVR groups.** Heatmap showing changes in T cell related functions with time in TAC+MMF group and TAC+EVR group.
