# Supplementary material for: Mechanistic analyses in kidney transplant recipients prospectively randomized to two steroid free regimen—Low dose Tacrolimus with Everolimus versus standard dose Tacrolimus with Mycophenolate Mofetil
Source: PLoS One. 2019 May 28;14(5):e0216300. doi: 10.1371/journal.pone.0216300 (PMC6538151; doi:10.1371/journal.pone.0216300)
Supplement: S2 File — (PDF) [file pone.0216300.s002.pdf]

Investigator's Name: Lorenzo Gallon MD.
Associated Professor of Medicine
UNOS Medical Director
Institution: Northwestern University Feinberg School of Medicine
Address: 676 North St. Clair Suite 1900,
Chicago, IL 60611
Tel: 312-695-4457
Email: l-gallon@northwestern.edu
Version: 11-10-2016
ePRISM ID: IIRP-1275

Title of study

Impact of two prednisone-free maintenance immunosuppressive regimens with reduced dose FK506+Everolimus vs. standard dose FK506+MMF on subpopulation of T and B cells, renal allograft function and gene expression profiles in renal allograft biopsies at 12, 24 and 36 months post-transplant. Prospective single center study in recipients of renal transplant allograft.

Study purpose and rationale

Immunosuppressive therapy with the calcineurin inhibitors (CNI) cyclosporine (CsA) and tacrolimus (Tac), has radically changed the field of organ transplantation. Ironically, although extensively and effectively used for kidney and other solid organ transplants, CsA and Tac cause significant adverse renal side effects: acute and chronic renal dysfunction, hemolytic-uremic syndrome, hypertension, electrolyte disturbances and tubular acidosis. Chronic nephrotoxicity from CNI has been implicated as a principal cause of post-transplant renal dysfunction. It is characterized by an irreversible and progressive tubular atrophy, interstitial fibrosis, and focal hyalinosis of small renal arteries and arterioles. Furthermore, by blocking IL2 production, this class of medications is also associated with a negative impact on generation of regulatory T cells, or (Tregs). (Tregs are an important subpopulation of helper T cells that has been associated with positive immunomodulation and donor specific hypo-responsiveness).

In renal transplant recipients, complete avoidance of calcineurin inhibitors from the time of renal transplant surgery has been associated with increased incidence of acute cellular rejection. The combination of mTOR inhibitors with full dose CNI has been shown to be synergistically nephrotoxic, and has been associated with poor graft outcome. We and other investigators have tested conversion from CNI to mTOR inhibitors with promising results. We have shown that in a prednisone-free immunosuppression regimen, conversion from tacrolimus to mTor inhibitors is safe, and not associated with an increased risk of acute rejection. More importantly, we have shown that it is associated with a persistent increase of regulatory T cells (Data presented at ATC 09 and 2010).

Recently, the A2309 study led to FDA-approval of Everolimus. The A2309 was a study designed to combine reduced dose CsA+Everolimus. Interestingly, the reduced exposure to CsA was not associated with an increased rate of ACR; renal allograft function was well maintained when compared to that of the control group. This study

study leads us to question the mechanism(s) behind the efficacy of a low dose CNI with an mTOR inhibitor in preventing acute allograft rejection.

The present proposal is designed to investigate the mechanisms of the synergistic effect(s) of low dose CNI and mTOR inhibitors (Everolimus) in controlling alloreactive T and B cells while expanding Tregs.

Our hypothesis, based on published data and our laboratory results (see preliminary data-Supportive documents), is that mTOR inhibitors allow expansion of Tregs and that low exposure to CNI is sufficient to control alloreactive T cells. Decreased exposure to CNI and concomitant increase of Tregs will then translate to improved renal allograft function and histology.

Objectives

The overall aim of the present study is to prospectively investigate the impact of two maintenance immunosuppressive regimens (both prednisone-free) on subpopulations of T and B cells and on renal allograft function at 12, 24 and 36 months post-randomization. One regimen combines low CNI exposure (tacrolimus) with concentration controlled Everolimus, and the second combines standard dose CNI (Tacrolimus) with MMF.

The primary endpoints of the study are to: 1) evaluate the impact of the two maintenance immunonusuppressive regimens on T cells subpopulations, including regulatory T cells and B cell subpopulations, at different time points post renal transplant, and2) determine the impact of the two regimens on renal allograft function at 12, 24 and 36 months post-randomization (post-transplant). Secondary endpoints of the study are to: 1) evaluate the impact of the two regimens on allograft immunohistopathology gene expression profiles and graft infiltrating cells (memory T cell ($CD4^+$ or $CD8^+$ $CD45RO^+$), T_{REG} ($CD4^+FoxP3^+$), memory B cell ($CD19^+CD27^+$)) at 12 and 24 months in renal allograft biopsies, and 2) evaluate the impact of the regimens on acute rejection, graft loss and death at 12, 24 and 36 months post-transplant.

Population

The study will be conducted at Northwestern University Feinberg School of Medicine/Northwestern Memorial Hospital, and subjects will be recruited during visits to the Comprehensive Transplant Center. We plan to enroll and randomize recipients and their respective donors until 40 recipients have started the study drug to which they were randomized. As of January 2014, 3 recipients randomized to the experimental arm and 1 randomized to the control arm were withdrawn prior to dosing due to surgical complications. Replacement pairs will be enrolled, bringing the prospective enrollment to a minimum of 44 donor/recipient pairs (at least 88 total subjects).

Inclusion Criteria:

1) Adults between 18 and 70 years of age
2) Either gender and of any ethnic background
3) Single-organ recipients (kidney only)
4) Able to understand the protocol and provide informed consent.
5) Recipient of living donor kidney transplants
6) PRA<20%

Exclusion criteria:
1) End Stage Renal Disease (ESRD) secondary to primary FSGS (focal segmental glomerulonephritis).
2) Inability to fully understand the purpose of the study and the inability to sign the informed consent
3) A significant or active infection
4) Pregnant or nursing females
5) History of severe hyperlipidemia not controlled with statins, subjects with Cholesterol > 400mg/dl
6) Platelet count <100,000mm3, WBC < 2,000mm3 (or clinical practice)
7) Any surgical, medical or psychiatric condition, other than the current transplant, which in the opinion of the investigator precludes enrollment into this trial

Number of centers & subjects
Northwestern University Feinberg School of Medicine /Northwestern Memorial Hospital.

Investigational and reference therapy
Everolimus. Dose: 0.75mg PO BID (concentration controlled).

Study duration
We expect the total duration for this study not to exceed 2 years from the date of the last subject'senrollment.

Study design
Renal Transplant Recipients:
All recipients will receive the same induction immunotherapy at time of transplant. : Campath (30mg IV x1) will be administered at the time of surgery. Methylprednisolone (500 mg IV) will be administered on the day of transplant and on days 1 (250 mg IV), and 2 (125 mg IV) post-transplant. No further steroids will be given post-transplant unless indicated by the following medical conditions: acute renal allograft rejection, renal diseases necessitating the use of steroids, and other systemic diseases such as rheumatoid arthritis (RA), systemic lupus erythematosus (SLE), and asthma.

Maintenance immunosuprression is administered according to the 1:1 randomization at time of enrollment: 20 subjects will begin **standard dose tacrolimus and MMF (control arm)**

Tacrolimus will be initiated according to our practice. The dose will be adjusted from day 3 on to achieve a target whole blood trough concentration of 8 ng/mL to 10 ng/mL. From month 2 until Month 6, the target tacrolimus trough level will be reduced to 6 ng/mL to 8 ng/mL. After month 6, the target level will be reduced to 4 ng/mL to 8 ng/mL.

MMF dose will be initiated as 1 g b.i.d. (2 g/day). Adjustments should be made for adverse events including but not limited to gastrointestinal intolerance and a decrease in WBC. MMF trough or AUC shall not be used to adjust dosing.

Twenty (20) subjects will begin low dose tacrolimus with concentration controlled everolimus (treatment arm)
Tacrolimus will be initiated according to our practice. In this treatment arm, the Tacrolimus dose will be adjusted from day 3 on, to a target whole blood trough concentration of 4 ng/mL to 7 ng/mL. From month 2 until Month 6, the target Tacrolimus trough level will be 3 ng/mL to 6 ng/mL. After month 6, the Tacrolimus dose should be adjusted in order to achieve a target trough level of 2 ng/mL to 5 ng/mL.

From day 5 on, the starting dose of Everolimus (0.75 mg bid) will be increased if the trough level is < 3 ng/mL, or reduced if the trough level is > 8 ng/mL.

All drugs will be administered in an open label fashion.

The following transplant recipient data will be collected at the time of randomization:.
Demographics: Age at transplantation, sex and race.
Causes of end-stage renal disease
Transplant related information: donor age, histocompatibility and cross match data, viral serology, history of acute rejection and delayed graft function, use of ACEI and/or ARB, level of renal allograft function - estimated GFR (e-GFR(12) using MDRD formula, proteinuria.

Blood samples will be obtained prior to randomization from all renal transplant recipients for baseline T and B cell functional activity and characterization of the cell subpopulations by flow cytometry.

-All recipients will be routinely followed at our Transplant center with post-transplant labs (SMA7, CBC, and relevant immunosuppression trough levels).

Additional blood will be collected and analyzed for the study at 3, 6, 12, 24 and 36 months post-randomization. If the final blood sample cannot be drawn at the 36 month timepoint, it may be drawn up to 50 months post-transplant. These samples will be used to investigate the impact of the two maintenance immunonusuppressive regimens on subpopulation of T cells In comparison with baseline levels. Specifically,

we will look at regulatory T cells (Treg: Foxp3+CD4+ CD25bright+) that could have been generated via the two maintenance immunonusuppressive regimens, and correlate the generation of with their function.

RNA from peripheral blood will be also collected and stored for post-hoc analyses of T and B-cell immunoresponse gene expression via gene arrays.

Kidney biopsy samples will be collected with standard-of-care samples at time of transplant (back table), 3 months, 12 months and at 24 months post-transplant. These will help evaluateand compare the impacts of the two maintenance immunonusuppressive regimens on allograft immunohistopathology. Gene expression profiles will also be analyzed. These samples will be processed in the principal investigator's research laboratory.

Both groups of subjects will be followed through 36-months post-randomization. In addition to monitoring renal allograft function, we will evaluate the incidence of acute rejection, subject and graft survival, incidence of hypertension (HTN), malignancies, opportunistic infections and post-transplant diabetes mellitus (DM).

Donors:
Blood samples from donor subjects will also be obtained at either the time of randomization or post-surgery during standard-of-care follow-up appointments These donor leukocytes will be used as stimulator cells in studies of functional activity of recipient T-cells. These samples will be processed in the principal investigator's research laboratory.

Safety:

Safety Endpoints: The primary safety endpoints will be assessed on the AEs and SAEs that are observed throughout the trial.
For the purpose of this clinical trial, the National Cancer Institute Common Terminology Criteria for Adverse Events v3.0 (CTCAE), dated December 12, 2003, will be used to grade all adverse events.

Recording of Adverse Events

At each contact with the subject, the investigator/member of investigator's team will seek information on adverse events by specific questioning and, as appropriate, by examination. Information on all adverse events will be recorded in the source document, and also in the appropriate adverse event module of the case report form (CRF). All clearly related signs, symptoms, and abnormal diagnostic procedures results should recorded in the source document, though should be grouped under one diagnosis.

All adverse events occurring during the study period will be recorded. The clinical course of each event should be followed until resolution, stabilization, or until it has been determined that the study treatment or participation is not the cause. Serious adverse events that are still ongoing at the end of the study period must be followed up to determine the final outcome. Any serious adverse event that occurs after the study period and is considered to be possibly related to the study treatment or study participation will be recorded and reported immediately.

Stopping Rules

Subjects developing post-transplant infections (i.e., UTI, CMV, HSV, EBV, HCV, HBV, HIV,PCP), that in the opinion of the investigator are detrimental to the subject and his/her participation in this research trial, will be stopped from participating in this study..

Patient Authorization: This study is to be conducted according to US and international standards of Good Clinical Practice (FDA Title 21 part 312 and International Conference on Harmonization guidelines), applicable government regulations and Institutional research policies and procedures.

This protocol and any amendments will be submitted to a properly constituted independent Institutional Review Board (IRB), (Northwestern University Institutional Review Board), in agreement with local legal prescriptions, for formal approval of the study conduct. The decision of the IRB concerning the conduct of the study will be made in writing to the investigator and a copy of this decision will be provided to the investigator/sponsor before commencement of this study. The investigator will place a list of IRB members and their affiliate in the regulatory binder for the clinical trial.

All subjects for this study will be provided with a printed consent form describing this study and providing sufficient information for subjects to make an informed decision

about their participation in this study. This consent form will be submitted with the protocol for review and approval by the IRB for the study. The formal consent of a subject, using the IRB-approved consent form, will be obtained before the subject is submitted to any study procedures. This consent form will be signed by the subject and the investigator-designated research professional obtaining the consent. The subject will be given a signed copy of the informed consent for their records.

Statistical analysis:

The primary endpoints of the study are graft function and subpopulations of T cells, including regulatory T cells and B cell subpopulations at 3, 6, 12, 24 and 36 months post kidney transplant. The secondary endpoints of the study include 1) incidence of acute rejection, graft loss, and death at 12,24 and 36 months post kidney transplant; 2) allograft immunohistopathology and gene expression profiles at 12 and 24 months post kidney transplant.

We will run univariate and bivariate analyses for all variables prior to our main analyses. We will evaluate continuous variables using t-tests or F-tests and categorical variables using χ^2. We will examine data distributions and test all variables for linear relationships or non-linear relationships.

Descriptive statistics (means, standard deviation, frequency) and correlations (both Pearson and Spearman) among variables will be calculated for preliminary data assessment.
Necessary transformation and imputations will be carried out based on the raw data distribution.

As missing data are inevitable in a longitudinal study, we will determine whether missing data are MCAR (data are missing completely at random), MAR (data are missing at random), or NMAR (not missing at random). If missing data are MCAR or MAR, it is likely that the standard multivariate computations using PROC MI (multiple imputations) will not result in biased standard error estimates. However, if missing data are NMAR, we will use the "pattern mixture" approach to compute a weighted average of the parameters that are associated with the missing data to estimate what the data would have been.

For the primary objective, we hypothesize that subjects receiving low dose CNI with mTOR inhibitor (Everolimus) will exhibit significantly greater graft function and higher numbers of regulatory T cells as compared to the standard dose CNI+MMF group. We will utilize both repeated-measures ANOVA and random mixed effect models to compare the trajectory change in graft function and proportion of regulatory T cells over 12, 24 and 36 months between the two experimental groups. The method of estimation will be maximum likelihood (ML).

A variety of covariance structures (first order regressive, compound symmetry, Toeplitz, variance components, unstructured) will be carefully examined and compared using the best fitting statistics, such as Akaike's Information (AIC) and Bayesian Information Criteria (BIC). These statistics are likelihood functions and can be compared across models. If, in spite of randomization, distributions of subjects' baseline characteristics are found to be different between the two experimental groups, these variables will be entered as covariates in the statistical analysis.

For the secondary endpoints, we will use logistic regression, Kaplan-Meier method, and Cox proportional hazards models to analyze time-to-event outcomes (acute rejection, graft failure, and death). For allograft immunohistopathology and gene expression profile outcomes, we will first develop a comprehensive immune profile of each participant by longitudinal analysis of frequency and function of T and B cell subsets. We will examine how these correlate with immunohistopathology and alloantibody production using Pearson or Spearman correlations. We will compare correlation coefficients between the two experimental groups using Z -tests to determine the distinct effects of each treatment arm. We will also compare the trajectory changes in cell populations and antibody production over 12, 24 and 36 months using both repeated measures ANOVA and random mixed effect models. All tests will be two-sided, and an error rate of $\alpha < 0.05$ will be considered statistically significant. All data analyses will be performed using SAS 9.2 statistical software (SAS Inc., Cary, NC).

Sample size and power

We calculated our sample power for the two primary endpoints based on our clinical experience and small pilot data. Using a two-tailed α of 0.05, the anticipated effect sizes corresponding to each specific aim between the two treatment arms were calculated using a minimum of 80% statistical power and assumed 10% attrition at 12 months.

For the sample size of n= 20 in each group, the proposed study will have a power of 0.82 and 0.99 to detect effect size of 1.0 and 2.0 for graft function and % regulatory T cell change, respectively, between the two treatment arms. Because this is a pilot study, we do not consider the statistical power of secondary outcomes; It is anticipated that a much larger sample size will be needed in order to achieve statistical power for the secondary endpoints in this proposed study.

Evaluation schedule:

Supportive documents and preliminary data:

Conversion from CNI to Mtor-based immunosuppression after kidney transplant changes the frequency and phenotype of Tregs

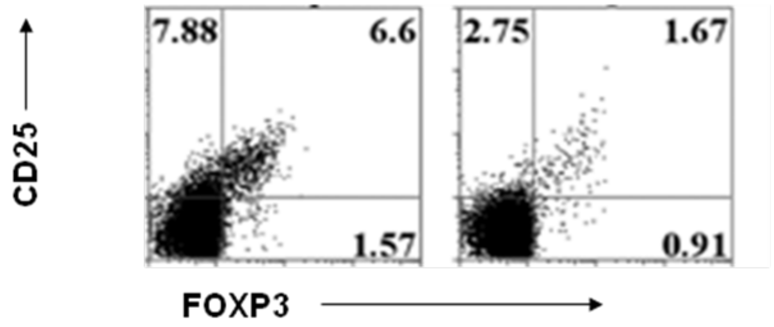

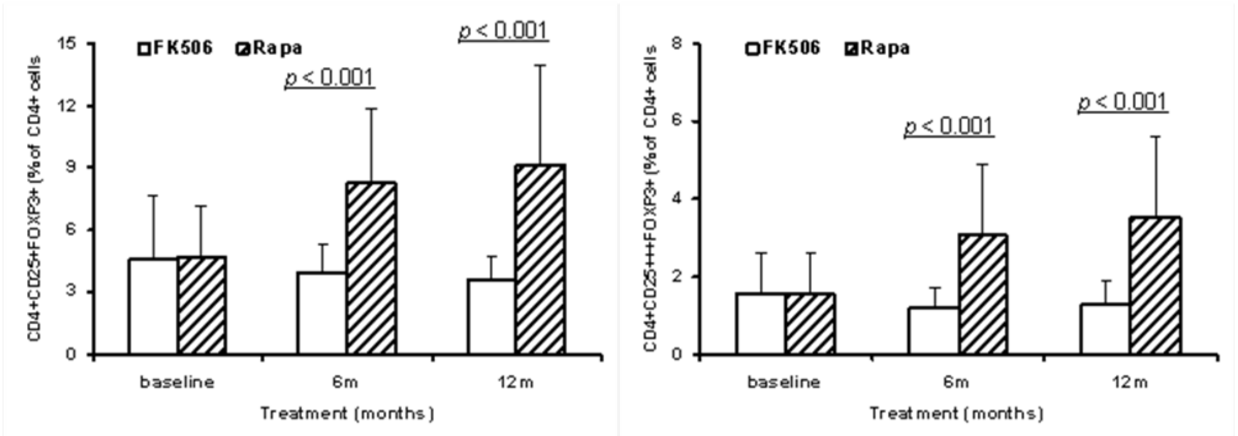

Abstract ATC 2010:

Lorenzo Gallon, Yuming Yu, Nader Najafian, Xuemei Huang, Giovanna La Monica, James Mathew, Josh Miller, Joseph Leventhal, Luting Xu Medicine/Comprehensive Transplant Center, Northwestern University, Chicago; Medicine, Harvard University

Subpopulation of T helper cells (Th1, Th17, and Treg) can play a major role in promoting rejection or can facilitate regulatory immune-mechanisms that can help the development of a pro-tolerant state towards the transplant. Little is known about the impact of immunosuppressive (IS) drugs on allospecific T cell subpopulations. Methods: A two-step culture system was used. Alloreactve memory CD4+ T cells were first generated in MLR culture with responding CD4+CD45RA+ nave T cells and allogeneic CD14+ monocytes. Memory CD4+ T cells, from the primary MLR, were enriched and re-stimulated by co-culturing with autologous CD14+ monocytes in the presence of antigenic stimulation of anti-CD3 to increase the frequency of Th1,Th17 and Treg cells. Two different IS agents, Tacrolimus (Tac) and Sirolimus (SRL) were added to the second culture alone or in combination at different concentrations. The effects on the generation of Th1, Th17, Treg cells from alloreactive memory T cells (CD4+CD45RO+) were then determined by intracellular cytokine staining. Results: After secondary stimulation, there was up to 10-fold increase, compared to primary stimulation, in the % of Th1, Th17 and Treg cells. Tac at low doses, significantly blocked the productions of IFN-γ (Th1) and IL-17 (Th17), while SRL even at high concentration (10ng/ml or 20ng/ml) had minimal effect on IFN-γ and IL-17 production (see top panels of figure). FOXP3 expression (Treg) was markedly increased in SRL compared to Tac (see bottom panel). When Tac and SRL were used in combination and at different concentrations, we found that Tac at 2-5 ng/ml with SRL at 2.5-10ng/ml achieved the maximal effect in inhibiting the production of IFN-γ and IL-17 while maintaining a high level of FOXP3 expression.

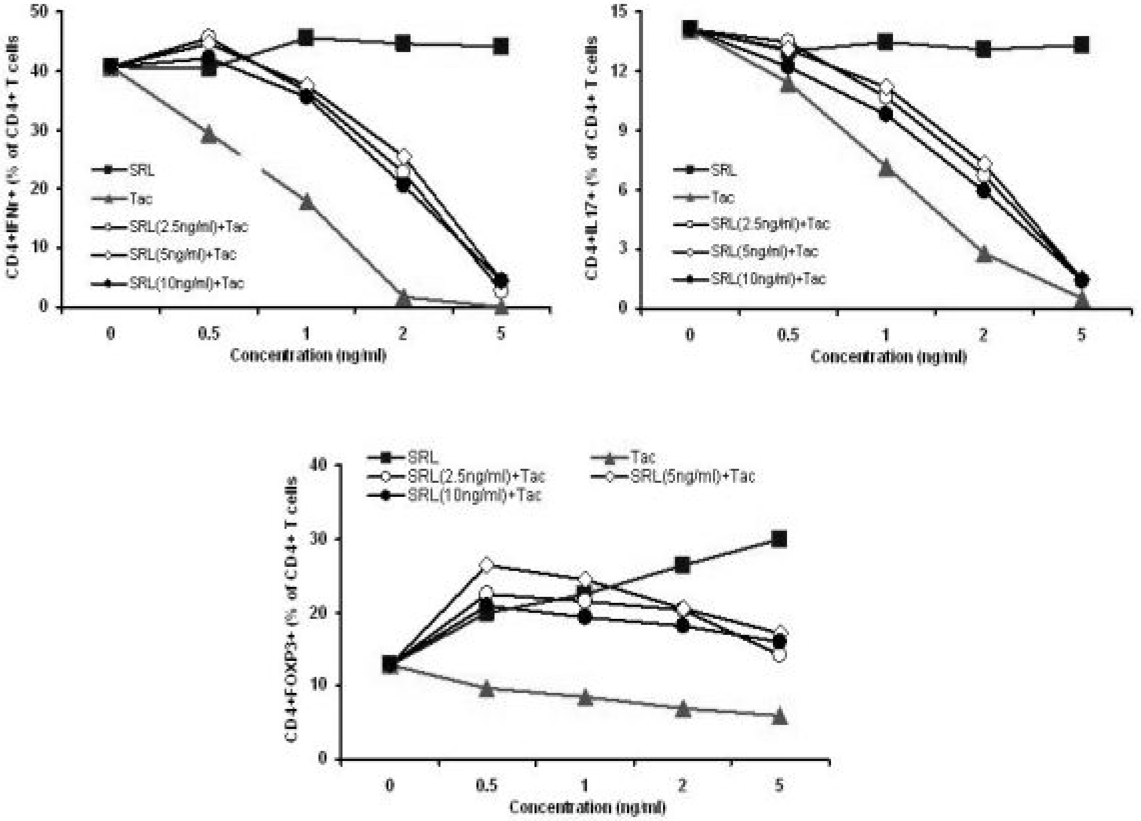

Conclusions: Tac and SRL have differential effects on subpopulation of T helper cells. These findings can help to guide the clinical use of IS drugs to promote Treg expansion while controlling Th1 and Th17 responses.
Keywords: Immunosuppression; Sirolimus (SLR); Calcineurin; T cell activation
Oral Session: Late-Breaking Basic Abstract Session (10:45 AM-12:15 PM)

Selected references to support this application:

1. Bennett WM. Insights into chronic cyclosporine nephrotoxicity. Int J Clin Pharmacol Ther 1996, 34:515.
2. Bennett WM, DeMattos A, Meyer MM, Andoh T, Barry JM. Chronic cyclosporine nephropathy: the Achilles' heel of immunosuppressive therapy. Kidney Int 1996, 50:1089.
3. Myers BD: Cyclosporine nephrotoxicity. Kidney Int 1986, 30:964.
4. Puschett JB, Greenberg A, Holley J, McCauley J. The spectrum of cyclosporine nephrotoxicity. Am J Nephrol 1990, 10: 296.
5. Myers BD, Ross J, Newton L, Luetscher J, Perlroth M. Cyclosporine-associated chronic nephropathy. N Engl J Med 1984, 311:699.
6. Goldstein DJ, Zuech N, Sehgal V, Weinberg AD, Drusin R, Cohen D. Cyclosporine-associated end-stage nephropathy after cardiac transplantation: incidence and progression. Transplantation 1997, 63:664.
7. Hornberger J, Best J, Geppert J, McClellan M. Risks and costs of end-stage renal disease after heart transplantation. Transplantation 1998, 66:1763.

8. Ojo AO, Held PJ, Port FK, et al. Chronic renal failure after transplantation of a nonrenal organ. N Engl J Med 2003, 349:931.
9. Burdmann EA, Andoh TF, Yu L, Bennett WM. Cyclosporine nephrotoxicity. Semin Nephrol 2003, 23:465.
10. Nankivell B., Alexander S. Rejection of the kidney allograft. New England Journal of Medicine 363:1451-1462 (2010).
11. Kim IK, Bedi DS, Denecke C, Ge X, Tullius SG. Impact of innate and adaptive immunity on rejection and tolerance. Transplantation 86:889-94 (2008)
12. Meier-Kriesche HU, Shold JD, Srinivas TR, Kaplan B. Lack of improvement in renal allograft survival despite a marked decrease in acute rejection rates over the most recent era. Am J Transplant 4:378-83 (2004)
13 Nankivell B, Borrows R, Fung C, et al. The natural history of chronic allograft nephropathy. N Engl J Med 349:2326:2333 (2003).
14 Pontrelli P, Rossini M, Infante B, et al. Rapamycin inhibits PAI-1 expression and reduces interstitial fibrosis and glomerulosclerosis in chronic allograft nephropathy. Transplantation 85: 125-134 (2008).
15 Lebranchu Y, Thierry A, Toupance O, Westeel P, Etienne I, Thervet E, Moulin B, Frouget T, Le Meur Y, Glotz D, Heng A, Onno C, Buchler M, Girardot-Seguin S, Hurault de Ligny B. Efficacy on renal function of early conversion from cyclosporine to sirolimus 3 months after renal transplantation: concept study. Am J Transplant 9:1115:1123 (2009)
16 Servais A, Meas-Yedid V, Toupance O, Lebranchu Y, Thierry A, Moulin B, Etienne I, Presne C, Hurault de Ligny B, Le Pogamp P, Le Meur Y, Glotz D, Hayem C, Olivo Marin J, Thervet E. Interstitial fibrosis quantification in renal transplant recipients randomized to continue cyclosporine or convert to sirolimus. Am J Transplant 9: 2552:2560 (2009)
17 Chhabra D, Grafals M, Cabral B, Leventhal J, Parker M, Gallon L. Late conversion of tacrolimus to sirolimus in a prednisone-free immunosuppression regimen in renal transplant patients. Clin Transplant 24 (2):199:206. 2010.
18. Levitsky J, Gallon L, Miller J, Tambur A, Leventhal J, Flaa C, Huang X, Sarraj B, Wang E, Mathew JM. Allospecific regulatory effects of sirolimus and tacrolimus in the human mixed lymphocyte reaction. Transplantation 27;91(2): 199-206, 2011
19. Battaglia, M., *et al.* Rapamycin and interleukin-10 treatment induces T regulatory type 1 cells that mediate antigen-specific transplantation tolerance. *Diabetes* 55, 40-49 (2006).
20. Battaglia, M., Stabilini, A. & Roncarolo, M.G. Rapamycin selectively expands CD4+CD25+FoxP3+regulatory T cells. *Blood* 105, 4743-4748 (2005).
21. Nikolaeva, N., Bemelman, F.J., Yong, S.L., van Lier, R.A. & ten Berge, I.J. Rapamycin does not induce anergy but inhibits expansion and differentiation of alloreactive human T cells. *Transplantation* 81,445-454 (2006).
22. Turnquist, H.R., *et al.* Rapamycin-conditioned dendritic cells are poor stimulators of allogeneic CD4+ T cells, but enrich for antigen-specific Foxp3+ T regulatory cells and promote organ transplant tolerance. *J Immunol* 178, 7018-7031 (2007).

23. Valmori, D., *et al.* Rapamycin-mediated enrichment of T cells with regulatory activity in stimulated CD4+ T cell cultures is not due to the selective expansion of naturally occurring regulatory T cells but to the induction of regulatory functions in conventional CD4+ T cells. *J Immunol* 177, 944-949 (2006).
24. Baan, C.C., *et al.* Differential effect of calcineurin inhibitors, anti-CD25 antibodies and rapamycin on the induction of FOXP3 in human T cells. *Transplantation* 80, 110-117 (2005).
25. Gao, W., *et al.* Contrasting effects of cyclosporine and rapamycin in de novo generation of alloantigen specific regulatory T cells. *Am J Transplant* 7, 1722-1732 (2007).
26. Bestard O, Cruzado J, Mestre M, Caldes A, Bas J, Carrera M, Torras J, Rama I, Moreso F, Seron D, Grinyo J. Achieving donor-specific hyporesponsiveness is associated with FoxP3+ regulatory T cell recruitment in human renal allograft infiltrates. Journal of Immunology 179 4901:4909 (2007)
27. Alvarez C, Opelz G, Garcia L, Susal C. Expression of regulatory T-cell-related molecule genes and clinical outcomes in kidney transplant recipients. Transplantation 87:857-863 (2009)
28. Noris M, Casiraghi F, Todeschini M, Cravedi P, Cugini D, Monteferrante G, Aiello S, Cassis L, Gotti E, Gaspari F, Cattaneo D, Perico N, Remuzzi G. Regulatory T cells and T cells depletion: role of immunosuppressive drugs. JASN 18: 1007-1018 (2007)
29. Ruggenenti P, Perico N, Gotti E, et al. Sirolimus versus cyclosporine therapy increases circulating regulatory T cells, but does not protect renal transplant patients given alemtuzumab induction from chronic allograft injury. Transplantation, 84: 956-964 (2007)
30. Wang Y, Camirand G, Lin Y, Froicu M, Deng S, Shlomchik W, Lakkis F, Rothstein D. Regulatory T cells require mammalian target of rapamycin signaling to maintain both homeostasis and alloantigen-driven proliferation in lymphocyte-replete mice. Journal of Immunology 186:000-000 (2011)
31. Valujskikh A. The challenge of inhibiting alloreactive T cell memory. Am J Transplantation 6: 647-651 (2006)
32. Valujskikh A, Baldwin III W, Fairchild R. Recent progress and new perspectives in studying T cell responses to allografts. Am J Transplantation 10: 1117-1125 (2010)
33. Pearl J, Parris J, Hale D, Hoffmann S, Bernstein W, McCoy K, Swanson S, Mannon R, Roederer M, Kirk A. Immunocompetent T cells with a memory-like phenotype are the dominant cell type following antibody-mediated T-cell depletion. Am J Transplantation 5:465-474 (2005)
34. Araki K, Turner A, Shaffer V, Gangappa S, Keller S, Bachmann M, Larsen C, Ahmed R. mTOR regulates memory CD8 T-cell differentiation. Nature 460:108-112 (2009)
35. Ferrer I, Wagener M, Robertson J, Turner A, Araki K, Ahmed R, Kirk A, Larsen C, Ford M. Cutting edge: Rapamycin augments pathogen-specific but not graft-reactive CB8+ T cell responses. The Journal of Immunology 185:2004-2008, (2010)
