# Supplementary material for: Mechanistic analyses in kidney transplant recipients prospectively randomized to two steroid free regimen—Low dose Tacrolimus with Everolimus versus standard dose Tacrolimus with Mycophenolate Mofetil
Source: PLoS One. 2019 May 28;14(5):e0216300. doi: 10.1371/journal.pone.0216300 (PMC6538151; doi:10.1371/journal.pone.0216300)
Supplement: S4 File — (PDF) [file pone.0216300.s004.pdf]

Table A: Clinical Data in Low Dose TAC + EVR Vs. TAC + MMF

Subject					Pre-transplant																Baseline Labs								
Subject ID	Study Drug	Age at Tx	Gender	Race	Pre-emptive Tx				Years on RRT prior to Tx	Cause of ESRD	DM	HTN	CAD	CHF (EF<50%)	BMI @ tX	PRA - I	PRA-II	HLA match	induction	DGF	WBC	HGB	SCr	Chol	TG	HDL	LDL	cmv	eGFR
E-01-R	Tac + EVR	33	0	0					0.46	1	1	1	0	0	38.96						11.5	10.6	7.04						8
E-02-R	Tac + EVR	69	0	2	0				0.27	3	0	1	0	0	25.53	0	0	3	0	0	6.9	11.2	5.42					-	11
E-03-R	Tac + EVR	39	0	2	0				3	0	0	1	0	0	22.69	0	0	3	0	0	9.9	11.6	0					-	6
E-11-R	Tac + EVR	24	0	1	0				6.37	0	0	1	0	0	24.93	0	0	1	0		6.5	6.4	20.34						3
E-15-R	Tac + EVR	63	0	0	0				1.86	4	0	1	0	0	26.5	0	0	4	0		7.7	13.6	3.64					-	17
E-18-R	Tac + EVR	21	0	2	0				1.16	5	0	0	0	0	27	0	0	3	0		6.5	10.5	5.81						10
E-22-R	Tac + EVR	67	0	0	1				N/A	0	1	1	0	0	27.9	0	0	1	0		7.5	10.6	4.77						17
E-23-R	Tac + EVR	31	0	2	0				1.62	0	0	1	0	0	27.49	0	0	3	0		4	9.6	6.84						10
E-26-R	Tac + EVR	46	1	0	0				0.98	4	0	1	0	0	20.32	0	0	1	0		7.8	10.5	4.57						10
E-27-R	Tac + EVR	49	0	2	0				0.55	1	1	1	0	0	27.47	0	0	4	0		7.8	11	6.41						9
E-29-R	Tac + EVR	42	0	3	0				0.43	1	1	1	0	0	29.76	0	0	3	0		10.8	10.6	6.86						9
E-30-R	Tac + EVR	36	1	2	1				N/A	2	0	1	0	0	18.87	0	0	1	0		8.5	10.7	7.09						7
E-31-R	Tac + EVR	30	1	2	0				4.03	3	0	1	0	0	20.84	11	37	0	0		8.7	10.5	17.7						
E-33-R	Tac + EVR	65	1	0	1				N/A	2	0	1	0	0	23.09	0	0	0	0		3.7	9	7.12						
E-34-R	Tac + EVR	67	1	0	0				3.36	0	0	1	0	0	26.95	4	4	0	0		10.2	11.6	5.31						
E-37-R	Tac + EVR	43	0	0	1				N/A	0	0	1	0	0	22.25	4	0	2	0		2.6	12.2	1.12						
E-39-R	Tac + EVR	53	0	0	0				0.22	1	1	1	0	0	36.88	0	0	3	0		12.8	9.3	5.5						11
E-40-R	Tac + EVR	62	0	2	0				0.81	1	1	1	1	0	30.67	0	0	1	0		6.4	11.6	5.03						12
E-42-R	Tac + EVR	35	0	2	0				0.97	4	0	1	0	0	18.22	0	0	3	0		10.7	11.5	9.55						6
E-43-R	Tac + EVR	59	1	0	1				N/A	5	0	0	0	0	17.47	8	0	2	0		6.9	9.1	5						9
**Mean**		**47**	**0**	**1**	**0**				**1.74**	**2**	**0**	**1**	**0**	**0**	**25.69**	**1**	**2**	**2**	**0**	**0**	**7.87**	**10.6**	**6.756**						**10**
SD		16	0	1	0				1.74	2	0	0	0	0	5.659	3	8	1	0	0	2.64	1.47	4.706						4

E-04-R	Tac + MMF	58	0	2	0				0.53	1	1	1	0	0	34.06	3	0	3	0	0	4.5	10.9	8.29					-	7
E-05-R	Tac + MMF	22	0	0	1				N/A	4	0	1	0	0	19.5	4	0	0	0		8.5	11	6.73					-	16
E-06-R	Tac + MMF	60	0	0	1				N/A	0	0	1	0	0	23.65	25	4	3	0		4.9	10.8	6.27					-	9
E-07-R	Tac + MMF	51	0	0	0				0.5	3	0	1	0	0	30.05	0	0	1	0		18.8	10.3	6.83					-	9
E-09-R	Tac + MMF	52	0	0	0				0.36	2	0	1	0	0	23.53	5	0	1	0		15.5	10	5.74	183	68	97	72	-	9
E-10-R	Tac + MMF	39	0	1	0				0.78	1	1	1	1	0	32.52	0	48	0	0		7.3	9.9	3.26					-	26
E-13-R	Tac + MMF	64	1	0	0				2.03	4	0	1	0	1	21.4	0	0	3	0		7.4	7.3	6.85						6
E-14-R	Tac + MMF	57	0	2	0				0.67	1	1	1	0	0	30.96	0	0	4	0		4.9	11.5	5.28					-	11
E-16-R	Tac + MMF	30	0	2	1	1	1	1	N/A	0	0	1	0	0	26.92	0	6	0	0		6.5	11.2	3.25					-	23
E-17-R	Tac + MMF	59	0	0	0				0.13	1	1	1	0	0	34.5	0	0	3	0		7.8	12.5	6.7						9
E-19-R	Tac + MMF	62	0	0	0				4.16	4	1	1	0	0	26.26	0	0	3	0		3.2	10.9	6.39						9
E-20-R	Tac + MMF	48	0	1	0				2.94	1	1	1	0	0	34.77	0	0	3	0		5.9	12.6	11.2						5
E-21-R	Tac + MMF	53	1	2	1				N/A	1	1	1	0	0	42.1	4	0	1	0		7.5	9.7	7.34						6
E-24-R	Tac + MMF	45	0	0	0				1.63	0	0	1	0	0	23.38	0	7	3	0		7	12.2	12.12						
E-25-R	Tac + MMF	52	1	0	1				N/A	4	0	1	0	0	30.23	7	0	3	0		3.3	11.9	3.46						14
E-28-R	Tac + MMF	24	1	0	0				0.7	4	0	0	0	0	18.43	0	11	2	0		6.6	12.8	6.23						8
E-32-R	Tac + MMF	63	0	0	1				N/A	1	1	1	0	0	27.67	14	0	3	0		5.1	9	4.02						
E-38-R	Tac + MMF	67	1	0	1				N/A	2	0	1	1	0	24.22	4	0	2	1		6	9.9	3.29						14
E-41-R	Tac + MMF	31	0	2	0				5.24	0	0	1	0	0	25.65	0	0	2	0		7.9	11.6	13.1						5
E-44-R	Tac + MMF	65	0	0	1				N/A	0	1	1	1	0	34.84	0	0	4	0		10.3	11.1	3.99						15
**Mean**		**50**	**0**	**1**	**0**	**1**	**1**	**1**	**1.64**	**2**	**0**	**1**	**0**	**0**	**28.23**	**3**	**4**	**2**	**0**	**0**	**7.45**	**10.9**	**6.517**	**183**	**68**	**97**	**72**		**11**
SD		14	0	1	1				1.66	2	1	0	0	0	6.067	6	11	1	0		3.78	1.34	2.872						6

Table A: Clinical Data in Low Dose TAC + EVR Vs. TAC + MMF

Subject		3 Months Lab																			
Subject ID	Study Drug	WBC3	HGB3	Creat3	Chol3	TG3	HDL3	LDL3	FK3	MPA3	EVL3	Ucr3	Upro3	pcr	pcr Code3	bkbl3	bku3	cmv3	eGFR3	DSA3	0=class I, 1 = class II
E-01-R	Tac + EVR	4.3	11.1	1.31															>60		
E-02-R	Tac + EVR	5.7	10.4	1.13					6.4			43.4	4	0.09	0	0	0	-	>60		
E-03-R	Tac + EVR	3.8	16.4	1.19					8.7			112.4	36	0.32	0	0	-	-	>60	0	
E-11-R	Tac + EVR	3.5	13	1.38					3.5		3.3				-	-	-	-	83		
E-15-R	Tac + EVR	3.3	11.6	1.19					5		4	49	<4	<1	0	0	0	-	>60	-	
E-18-R	Tac + EVR	3.8	13	1.36					3.2		3.6	168.2	21	0.12	0	0	0		>60	0	
E-22-R	Tac + EVR	3.2	13	1.19					8.5		5.8	42.4	4.5	0.11	0	0	0		>60		
E-23-R	Tac + EVR	3.6	13.6	0.93					5.6		4.8	82.2	7	0.09	0		0		>60		
E-26-R	Tac + EVR	4	13.7	0.85					4.9		4.5	145.1	14	0.1	0	0	0		>60		
E-27-R	Tac + EVR	4.4	14.9	0.97					7.2		4.2	168	6.19	0.04	0				81		
E-29-R	Tac + EVR	5.4	12.2	1.55					3		3.1	173.9	46	0.26	0		0		50		
E-30-R	Tac + EVR	4.4	13.5	0.83					6.4		7.1	101.8	59	0.58	0	0	0		>60		
E-31-R	Tac + EVR	3.3	11.7	0.85	194	70	58	122	6.9		5.2	188.3	33	0	0	0	5E+08		>60		
E-33-R	Tac + EVR	2.2	12.3	0.72					7.6		1.9	35.1	<4	<1	0				>60		
E-34-R	Tac + EVR	4.1	10.9	1.3					<2		3.7	108.6	51	0.47	0	0	2E+07		41		
E-37-R	Tac + EVR	2.6	12.2	1.12					6.6		5.4	136	11	0.08	0	0	0				
E-39-R	Tac + EVR	6	10.6	1.09					5		3.7					0	0		>60		
E-40-R	Tac + EVR	2.2	10.9	1.89					3.5		5.1	198.8	325	1.63	1		0	0	46		
E-42-R	Tac + EVR	5.3	11.6	1.41					3.2		6.6	18.5	<4		0				57		
E-43-R	Tac + EVR	5.8	5.4	14	1.6				5.4		5.1	67.7	63	0.93	0	0	0				
**Mean**		**4**	**12.1**	**1.81**	**98**	**70**	**58**	**122**	**5.6**		**4.5**	**108.2**	**48.6**	**0.34**	**0**	**0**	**4E+07**	**0**	**59.7**	**0**	
SD		1.1	2.18	2.88	136				1.8		1.3	59.19	82.3	0.45	0	0	1E+08		18.1	0	
E-04-R	Tac + MMI	3.6	12.3	1.1					5.1			-	-	-	-	-	-	-	>60	-	
E-05-R	Tac + MMI	1	12.2	1.02					16			137.5	29	0.21	0	0	-	-	>60	1	1
E-06-R	Tac + MMI	2.5	12.1	1.68					4.8	13.4		44.2	<4	<1	0	0	-	-	47		
E-07-R	Tac + MMI	2.5	12.1	1.68					10			91	350	3.85	2	<250	-	-	>60		
E-09-R	Tac + MMI	7.5	12	1.05					6.8			87.5	20	0.23	0	0	-	-	>60	0	
E-10-R	Tac + MMI	1.8	11.6	1.77								106.4	39	0.37	0	<250	6E+08	-	52		
E-13-R	Tac + MMI	6.6	13.2	0.74					8			42.5	5	0.12	0	0	3E+07	-	86	0	
E-14-R	Tac + MMI	1.1	10.7	1.55					7.8			148	59	0.4	0	0	0	-	41	-	
E-16-R	Tac + MMI	2.3	13.3	1.33					8.1			112.2	14	0.12	0	0	-	-	>60	-	
E-17-R	Tac + MMI	6.1	10.6	0.94					7.5			157.8	66	0.42		0			>60	0	
E-19-R	Tac + MMI	3.7	8.8	1.8	119		47	59	9.9			172.4	29	0.17	0	0	0		38		
E-20-R	Tac + MMI	2.4	13.5	1.84					11	1.6		291.7	41	0.14		0			54		
E-21-R	Tac + MMI	4.2	12.8	1.17					15			58	<4	<1	0	11750	1E+09				
E-24-R	Tac + MMI	3.7	13.6	1.56								202.8	197	0.97	0	0	0				
E-25-R	Tac + MMI	4.4	12.5	0.92	231		48	146	4										57	0	
E-28-R	Tac + MMI	2.7	14.6	0.75					4.7			126.7	11	0.09	0	0	7E+07		>60	0	
E-32-R	Tac + MMI	6.2	10.7	1.36					14			34.9	<4	<1	0	0	0			0	
E-38-R	Tac + MMI	7.3	10.2	1.73					6.5		5.3				0	0			38		
E-41-R	Tac + MMI	5.9	12.3	2.05					5.7									0	38		
E-44-R	Tac + MMI	5.8	9.9	1.14					10			76.9	14	0.18	0	0	0				
**Mean**		**4.1**	**12**	**1.36**	**175**		**48**	**103**	**8.6**	**7.5**	**5.3**	**118.2**	**67.2**	**0.56**	**0**	**783**	**2E+08**	**0**	**50.1**	**0**	**1**
SD		2.1	1.44	0.4	79		1	62	3.6	8.34		67.54	98.4	1.02	1	3034	4E+08		15.4	0	

Table A: Clinical Data in Low Dose TAC + EVR Vs. TAC + MMF

Subject		6 Months Lab																		
Subject ID	Study Drug	WBC6	HGB6	Creat6	Chol6	TG	HDL	LDL	FK	MPA	EVL	Ucr	Upro	pcr	pcr Code	bkbl	bku	cmv	eGFR	DSA
E-01-R	Tac + EVR	6.6	11.3	1.84					7.2	3.9									47	
E-02-R	Tac + EVR	4.9	11.6	1.37					7.9	-	4.2								52	
E-03-R	Tac + EVR	5.1	16.9	1.07					3.7		2.7			-					87	
E-11-R	Tac + EVR	2.4	13.8	1.4					2.6		3.4	-	-	-	-	0	0	-	81	
E-15-R	Tac + EVR	3.3	13.2	1.31					5.4	5.1		208	12	0.06	0	0	484885	0	58	
E-18-R	Tac + EVR	4.6	14.6	2.76					3.3		2.9	72	8	0.11	0	0	0		37	
E-22-R	Tac + EVR	5.9	14.5	1	128	102	46	62	5.4	4.7										
E-23-R	Tac + EVR	4.3	13.5	0.98					2.8	1.8									>60	
E-26-R	Tac + EVR	3.3	13.4	0.72					5	<1								0	>60	
E-27-R	Tac + EVR	6.97	16.2	0.98					4.3							0	761000		81	
E-29-R	Tac + EVR	6.6	13	1.65					5	4.2						0	0		51	
E-30-R	Tac + EVR	3.6	13.9	0.8					5.9	5			0						95	
E-31-R	Tac + EVR	4	12.5	0.77					8.1		2.5	127	0	0	0	0	130241		>60	
E-33-R	Tac + EVR	6.4	12.2	0.79	195	154	55	115	4	3.9		53	8	0.15	0	0	0		79	
E-34-R	Tac + EVR	4.3	13.7	1.69					4.4		3.5	222.9	83	0.37	0	0	0		31	
E-37-R	Tac + EVR	2.9	13.3	1.17													0		>60	
E-39-R	Tac + EVR	7.3	11.3	1.22					5.1	4.7						0	0		62	
E-40-R	Tac + EVR	4.8	13.6	1.76	171	442	32		10.7		10.2	160	203	1.27	1	0	0		41	
E-42-R	Tac + EVR	5	11	2.2					<2										31	
E-43-R	Tac + EVR	8.7	14.5	2.33					5.7		3.6								21	
**Mean**		**5.05**	**13.4**	**1.39**	**165**	**233**	**44**	**89**	**5.36**	**4.2**	**4.13**	**140.5**	**45**	**0.33**	**0**	**0**	**125102**	**0**	**57**	
SD		1.65	1.54	0.57	34	183	12	37	2.05	1.1	2.51	69.65	76	0.48	0	0	256718	0	23	
E-04-R	Tac + MM	3	12.7	1.05					3.9			165.3	20	0.12	0	0	0	-	-	
E-05-R	Tac + MM	7	13.2	9.3					3.6			137	97	0.71	0	0	-	0	116	
E-06-R	Tac + MM	3.7	13.2	1.63					7.5	15		197	17	0.09	0	-	2E+08	-	43	
E-07-R	Tac + MM	6.6	12	1.21					5.4	1.1		134	101	0.75	0	-	-	-	69	
E-09-R	Tac + MM	4.5	13.6	1.17	230	92	69	143	4.8			177	40	0.23	0	0	-	-	71	
E-10-R	Tac + MM	3.2	12.6	1.4					3.6	1.7		77	0	0	0	##	2E+06	-	72	
E-13-R	Tac + MM	7	12.9	0.73					6.9										>60	
E-14-R	Tac + MM	5.4	13.8	1.74					6.1			148	59	0.4	0	0	0	-	41	
E-16-R	Tac + MM	4.2	13.7	1.31					3.1			-	-	-	-	-	53168		<60	
E-17-R	Tac + MM	3.3	13.6	1					6			101.7	30	0.29	0		1E+08		>60	
E-19-R	Tac + MM	3	11.5	1.58															46	
E-20-R	Tac + MM	2.6	14.8	1.66					9.3	1.1		203.5	109	0.54	0	0			44	
E-21-R	Tac + MM	4.9	13	1					5										58	
E-24-R	Tac + MM	5.5	13.8	1.49					4.4	4.8		173.8	27	0.16	0				51	
E-25-R	Tac + MM	4.5	12.9	1.04					4.3	3.5		61	0	0	0				62	
E-28-R	Tac + MM	8.8	16.5	0.9	148	56	85	55	5.7	1.6		102.9	7	0.07	0	##			>60	
E-32-R	Tac + MM	5.4	12.5	1.27	179	180	55	88	3	4.2		108	8	0.07	0	0	<500		60	
E-38-R	Tac + MM	9.5	11.3	1.6						on CSA										0
E-41-R	Tac + MM	5.6	14	1.7					7.4			91.4	17	0.19	0				47	
E-44-R	Tac + MM	7.2	12.8	1.26					6.3										59	
**Mean**		**5.25**	**13.2**	**1.7**	**186**	**109**	**70**	**95**	**5.35**	**4.2**		**134.1**	**38**	**0.26**	**0**	**##**	**6E+07**	**0**	**60**	**0**
SD		1.95	1.14	1.81	41	64	15	44	1.72	4.7		45.15	38	0.25	0	##	1E+08		19	

Table A: Clinical Data in Low Dose TAC + EVR Vs. TAC + MMF

Subject		3 Months Lab																			
Subject ID	Study Drug	WBC	HGB	Creat	Chol	TG	HDL	LDL	FK	MPA	EVL	Ucr	Upro	pcr	pcr Code	bkbl	bku	cmv	eGFR	DSA	0=class I, 1 = class II, 2= both classes
E-01-R	Tac + EVR																				
E-02-R	Tac + EVR	5.2	12.7	1.08	179		38	75	4.6		3.2	66.6	5	0.08	0	0	0		>60		
E-03-R	Tac + EVR	5.5	23.9	1.08	247		42	174	5.7		5.8	116	373	3.22	2	0	0		>60	0	
E-11-R	Tac + EVR	2.5	15.1	1.22	178		49	120	2.4		4.4	120.8	14	0.12	0	<50	<50	-	>60	0	
E-15-R	Tac + EVR	5.7	13.8	1.32	185		34	119	3.8		3.9	199.4	12	0.06	0	0	0	-	55		
E-18-R	Tac + EVR	5.2	16.7	1.5	198		30	116	3.2		3.1	327.1	31	0.09	0	0	0		59		
E-22-R	Tac + EVR	8.3	14.6	1					6.5		5.3	84.8	9	0.11	0		0		>60		
E-23-R	Tac + EVR	7.5	14.5	1.02					3.1		3.3		<1.5			0			97		
E-26-R	Tac + EVR	3.3	13.4	0.72					2.8		2.6					0	0		94		
E-27-R	Tac + EVR	6.1	18.8	1.08	199		53	127	10.7		6.2	132.7	11	0.08	0	0	0		>60		
E-29-R	Tac + EVR	5.8	14.1	1.5	98		31	109	4.9		4.3	103.3	19	0.18	0	0	0	51			
E-30-R	Tac + EVR	5.7	13.5	0.73	188		45	67	5.3		6	182	10	0.05	0	0	57168		>60		
E-31-R	Tac + EVR	4.6	13	0.74					4.3		4.2	139.6	24	0.17	0	0	0		>60	1	2
E-33-R	Tac + EVR	3.5	14.3	0.78					3.9		4.4	23	6	0.26	0			0	80		
E-34-R	Tac + EVR																				
E-37-R	Tac + EVR	3.9	15	1.05	244		53	172	2.8		5.5	144.5	14	0.1	0	0	0		>60	0	
E-39-R	Tac + EVR	6.9	10.2	1.09	122	472	21	7	4.4		6.1								70		
E-40-R	Tac + EVR	3.5	14.1	1.27	84		25	26	5.3		9.2	124.6	119	0.96	0	0	0		57		
E-42-R	Tac + EVR	4.3	12.8	2	236		30					115.1	28	0.24	0	0	0		41	0	
E-43-R	Tac + EVR	7.2	11.5	2.4	174	111	81	71	3.3		7.8	81	47	0.58	0	0	0			1	0
**Mean**		**5.3**	**14.6**	**1.2**	**179**	**292**	**41**	**99**	**4.53**		**5**	**130.7**	**48**	**0.42**	**0**	**0**	**4083**	**26**	**69**	**0**	**1**
SD		1.6	2.98	0.44	52	255	16	51	1.96		1.7	69.61	94	0.81	1	0	15279	36	20	1	1.41421

E-04-R	Tac + MM	3.4	12.6	1.05	154		39	90	4.6			132.1	21	0.16	0	0	0		>60	0	
E-05-R	Tac + MM	5.3	13.8	1.12	111		27	77	7.5			120.5	76	0.63	0	0	0		>60	1	0
E-06-R	Tac + MM	4.6	12.6	1.75	139		46	81	6.2			86.4	4	0.05	0	0	0	-	43	1	2
E-07-R	Tac + MM	6	13.8	1.16	146		39	81	7.1			111.3	11	0.1	0	0	0	-	>60	0	
E-09-R	Tac + MM	5.7	14	1.16					6.9	3		66	7	0.11	0	<50	-	-	>60		
E-10-R	Tac + MM	3.7	12.9	1.42	136		42	77	4.9			70.8	28	0.4	0	0	2807	-	>60	1	1
E-13-R	Tac + MM	7.1	13.1	0.73	157		49	80	8.6			99.3	9	0.09	0	<50	2E+06	-	>60		
E-14-R	Tac + MM	3.9	12.2	1.69	165		22	-	6.1			192.7	19	0.1	0	-	-	-	41		
E-16-R	Tac + MM	6.3	14.8	1.4	121		31	47	6.7			124.4	16	0.13	0	0	948	-	59	1	0
E-17-R	Tac + MM	4.2	14.1	0.8	124	85	40	67	6.1	2									>60		
E-19-R	Tac + MM	3.3	13.6	1.67	131		47	62	5.9			244.3	19	0.08	0	5024	2E+07		42		
E-20-R	Tac + MM	2.8	16.2	1.69					8.1			186.9	144	0.77	0		0		53		
E-21-R	Tac + MM	4.9	13.1	1.22	169	33	104	40	3	4		142.3	17	0.12	0	0	1889		55	0	
E-24-R	Tac + MMF																				
E-25-R	Tac + MM	5.2	14.6	1.11	212		45	138	7.9			117.8	17	0.14	0	0	0		52	0	
E-28-R	Tac + MM	4.9	16.1	0.8	136		66	60	6.4			82.7	10	0.12	0	0	0		>60		
E-32-R	Tac + MM	56	12.3	1.13	152		43	79	4.6	3		196	14	0.07	0	0	0		>60	0	
E-38-R	Tac + MMF																				
E-41-R	Tac + MM	4.1	14.5	1.92	189		35	101	8.8			131.1	31	0.24	0	0	0		41		
E-44-R	Tac + MMF																				
**Mean**		**7.7**	**13.8**	**1.28**	**149**	**59**	**45**	**77**	**6.44**	**3.0**		**131.5**	**28**	**0.21**	**0**	**419**	**1E+06**		**48**	**0**	**0.75**
SD		12	1.19	0.36	27	37	19	24	1.55	1		50.34	35	0.21	0		5E+06		7.3	1	0.95743

Table A: Clinical Data in Low Dose TAC + EVR Vs. TAC + MMF

Subject		18 Months Lab																			
Subject ID	Study Drug	WBC	HGB	Creat	Chol	TG	HDL	LDL	FK	MPA	EVL	Ucr	Upro	pcr	pcr Code	bkbl	bku	cmv	eGFR	DSA	0=class I, 1 = class II, 2= both classes
E-01-R	Tac + EVR																				
E-02-R	Tac + EVR	6	13.2	1.16					7.9		5.6	-	-	-	-	-	-	-	63		
E-03-R	Tac + EVR													-							
E-11-R	Tac + EVR	3.4	15.3	1.27					4.2		6.6	-	-	-	-	0	0	-	90		
E-15-R	Tac + EVR	3.6	14.3	1.34					4.8		4.5								56		
E-18-R	Tac + EVR	6.2	15.1	1.39					8.8		3.6	157	12	0.08	0	0			71		
E-22-R	Tac + EVR	7.3	14.9	1	154	95	54	81	7.1										>60		
E-23-R	Tac + EVR	5.4	15.7	0.84	221	419	39	N/a	4.7		3.2	0	0	0	0				116		
E-26-R	Tac + EVR																				
E-27-R	Tac + EVR				215		58	134													
E-29-R	Tac + EVR	7	14.3	1.57	191	150	38	123	4.3		4.3	100	18	0.18	0				54		
E-30-R	Tac + EVR											18		0							
E-31-R	Tac + EVR																				
E-33-R	Tac + EVR																				
E-34-R	Tac + EVR																				
E-37-R	Tac + EVR																				
E-39-R	Tac + EVR																				
E-40-R	Tac + EVR																				
E-42-R	Tac + EVR																				
E-43-R	Tac + EVR																				
**Mean**		**5.6**	**14.7**	**1.22**	**195**	**221**	**47**	**113**	**5.97**		**4.6**	**68.75**	**10**	**0.065**	**0**	**0**	**0**	**##**	**75**		
SD		1.5	0.83	0.25	30	173	10	28	1.91		1.3	73.18	9.2	0.0854	0	0		##	24		

E-04-R	Tac + MMF	4.9	12.1	1.09					4.8					-					>60		
E-05-R	Tac + MMF																				
E-06-R	Tac + MMF	5.8	13	1.57	139		46	81	6.2			86.4	4	<1	0	0	51	-		1	2
E-07-R	Tac + MMF	6.9	13.4	1.05					6.3	1.1		130	12	0.09	0	-	-	-	81		
E-09-R	Tac + MMF	4.9	14.5	1.16	237	115	45	169	6.1	8.7		215	37	0.17	0	0	-	-	71		
E-10-R	Tac + MMF	2.6	13.7	1.19					6	1.8		59	109	1.85	0	-	-	-	76		
E-13-R	Tac + MMF																				
E-14-R	Tac + MMF	3.4	12.2	1.67					3.1										45		
E-16-R	Tac + MMF	5.1	14.1	1.3	144	217	40	61	6.6			164.8	23	0.14		-			>60	0	
E-17-R	Tac + MMF																				
E-19-R	Tac + MMF	4.5	13.1	1.41					4.9							0	493	0	51		
E-20-R	Tac + MMF	2.3	16.7	1.98	303		39	N/A	7.9	4.2		105	58	0.55	0	0		0	44		
E-21-R	Tac + MMF	4.8	12.7	1.08					3.5										58		
E-24-R	Tac + MMF																				
E-25-R	Tac + MMF	6.3	13.8	0.95					10.4										68		
E-28-R	Tac + MMF	5	15.6	0.7	125	68	63	48	6.3							0	0		>60		
E-32-R	Tac + MMF	7.8	13.1	1.07					8.3	4		51	<4	<1	0				73		
E-38-R	Tac + MMF																				
E-41-R	Tac + MMF																				
E-44-R	Tac + MMF																				
**Mean**		**4.9**	**13.7**	**1.25**	**190**	**133**	**47**	**90**	**6.18**	**4**		**115.9**	**41**	**0.56**	**0**	**0**	**181**	**0**	**63**	**1**	**2**
SD		1.6	1.31	0.34	77	76	10	55	1.95	3		58.95	39	0.7439	0	0	271	0	14	1	

Table A: Clinical Data in Low Dose TAC + EVR Vs. TAC + MMF

Subject		24 Months Lab																			
Subject ID	Study Drug	WBC	HGB	Creat	Chol	TG	HDL	LDL	FK	MPA	EVL	Ucr	Upro	pcr	pcr Code	bkbl	bku	cmv	eGFR	DSA	0=class I, 1 = class II, 2= both classes
E-01-R	Tac + EVR																				
E-02-R	Tac + EVR	5.4	13.7	1.13	162		41	77	6.2		4.3	94.3	6	0.06	0	0	0		>60		
E-03-R	Tac + EVR																			0	
E-11-R	Tac + EVR	3.9	14.8	1.16	199	74	53	131	4.6		6.9	28.5	4	0.14					>60		
E-15-R	Tac + EVR	5.6	11.8	1.21					2.4		3.1	169	9	0.05	0				63		
E-18-R	Tac + EVR	6.1	16.2	1.47																	
E-22-R	Tac + EVR																				
E-23-R	Tac + EVR																				
E-26-R	Tac + EVR																				
E-27-R	Tac + EVR																				
E-29-R	Tac + EVR																				
E-30-R	Tac + EVR																				
E-31-R	Tac + EVR																				
E-33-R	Tac + EVR																				
E-34-R	Tac + EVR																				
E-37-R	Tac + EVR																				
E-39-R	Tac + EVR																				
E-40-R	Tac + EVR																				
E-42-R	Tac + EVR																				
E-43-R	Tac + EVR																				
**Mean**		**5.3**	**14.1**	**1.24**	**181**	**74**	**47**	**104**	**4.4**		**4.8**	**97.27**	**6**	**0.08333**	**0**	**0**	**0**		**63**	**0**	
SD		0.9	1.86	0.16	26		8	38	1.9		1.9	70.3	3	0.04933	0						

E-04-R	Tac + MMF	4.4	13.5	1.09	184		45	101	5.5			99.8	20	0.2	0	165	300652		>60	0	
E-05-R	Tac + MMF	7.2	13.2	1.09	148		25	111	9.1			161.4	95	0.59	0	0	0	-	>60	1	0
E-06-R	Tac + MMF	5.9	13.1	1.45	154		55	88				69.7	5	0.07	0				49		
E-07-R	Tac + MMF	7.1	13.8	0.95					6	1.2		-	-		-	-	-	-	92		
E-09-R	Tac + MMF																				
E-10-R	Tac + MMF																				
E-13-R	Tac + MMF																				
E-14-R	Tac + MMF																				
E-16-R	Tac + MMF																				
E-17-R	Tac + MMF																				
E-19-R	Tac + MMF																				
E-20-R	Tac + MMF																				
E-21-R	Tac + MMF																				
E-24-R	Tac + MMF																				
E-25-R	Tac + MMF																				
E-28-R	Tac + MMF																				
E-32-R	Tac + MMF																				
E-38-R	Tac + MMF																				
E-41-R	Tac + MMF																				
E-44-R	Tac + MMF																				
**Mean**		**6.2**	**13.4**	**1.15**	**162**		**42**	**100**	**6.9**	**1.2**		**110.3**	**40**	**0.28667**	**0**	**83**	**150326**		**71**	**1**	**0**
SD		1.3	0.32	0.21	19		15	12	2			46.74	48	0.27062	0	117	212593		30	1	

Table A: Clinical Data in Low Dose TAC + EVR Vs. TAC + MMF

Subject		Rejection												
Subject ID	Study Drug	Rejection	Date of 1st Rejection	Type of rejection	Borderline change	Graft failure	Death	BK blood	BK urine	CMV/QNT or CMV DNA/PCR	Proteinuria (PC ratio >1)	Group	FU time	FU time rejection
E-01-R	Tac + EVR	1	4/18/2013	1								0		
E-02-R	Tac + EVR	0			0	0	0	0	0	0	0	1	33	33
E-03-R	Tac + EVR	0			0	0	0	0	0	0	1	1	34	34
E-11-R	Tac + EVR	0			0	0	0	0	0	0	1	1	34	34
E-15-R	Tac + EVR	0			1	0	0	1	1	0	0	1	29	29
E-18-R	Tac + EVR	0			0	0	0	1	1	0	1	1	33	33
E-22-R	Tac + EVR	0			0	0	0	0	0	0	0	1	32	32
E-23-R	Tac + EVR	0			0	0	0	0	1	0	0	1	31	31
E-26-R	Tac + EVR	0			0	0	0	0	0	0	0	1	29	29
E-27-R	Tac + EVR	0			0	0	0	0	1	0	1	1	20	20
E-29-R	Tac + EVR	0			1	0	0	0	0	0	0	1	25	25
E-30-R	Tac + EVR	0			1	0	0	0	1		0	1	27	27
E-31-R	Tac + EVR	1	12/29/2015	1	0	0	0	1	1		1	1	30	25
E-33-R	Tac + EVR	0			0	0	0	0	0	0	0	1	29	29
E-34-R	Tac + EVR	0			0	0	0					1		
E-37-R	Tac + EVR	0			0	0	0	0	0		0	1	24	24
E-39-R	Tac + EVR	0			1	0	0	0	0		1	1	27	27
E-40-R	Tac + EVR	0			0	0	0	0	0	0	1	1	26	26
E-42-R	Tac + EVR	0			1	0	0	0	0	0	0	1	5	5
E-43-R	Tac + EVR	0			0	0	0	0	0	0	0	1	16	16
**Mean**		**0.1**	**41874.5**	**1**	**0.26**	**0**	**0**	**0**	**0.3**	**0**	**0.3889**	**1**	**26.9**	**26.6111**
SD		0.3	696.50018	0	0.45	0	0	0	0.5	0	0.5016	0	7.28	7.24479

E-04-R	Tac + MMF	0			1	0	0	0	1	0	0	0	39	39
E-05-R	Tac + MMF	0			0	0	0	0	0	0	0	0	37	37
E-06-R	Tac + MMF	1	6/10/2014	1	0	0	0	0	1		0	0	23	12
E-07-R	Tac + MMF	0			0	0	0	0	0	0	1	0	35	35
E-09-R	Tac + MMF	0			0	0	0	0	0	0	0	0	35	35
E-10-R	Tac + MMF	1	7/10/2014	1	1	0	0	1	1	0	1	0	33	12
E-13-R	Tac + MMF	0			0	0	0	0	1		1	0	33	33
E-14-R	Tac + MMF	0			1	0	0	0	1	0	0	0	15	15
E-16-R	Tac + MMF	1	8/7/2014	1	1	0	0	0	1	1	0	0	30	12
E-17-R	Tac + MMF	0			0	0	0	0	1	0	1	0	11	11
E-19-R	Tac + MMF	0			1	0	0	1	1	0	0	0	32	32
E-20-R	Tac + MMF	0			0	0	0	0	0	0	1	0	26	26
E-21-R	Tac + MMF	0			0	0	0	0	0	0	0	0	30	30
E-24-R	Tac + MMF	0			0	0	0	0	0		1	0	5	5
E-25-R	Tac + MMF	0			0	0	0	0	1	0	0	0	26	26
E-28-R	Tac + MMF	1	12/2/2014	1	0	0	0	0	1	1	0	0	12	12
E-32-R	Tac + MMF	0			1	0	0	0	1	0	0	0	29	29
E-38-R	Tac + MMF	0			0	0	0	1	1	0	0	1	5	5
E-41-R	Tac + MMF	0			0	0	0	0	0	0	1	0	16	16
E-44-R	Tac + MMF	0			0	0	0	0	0	0	0	0	13	13
**Mean**		**0.2**	**41865.75**	**1**	**0.3**	**0**	**0**	**0**	**0.6**	**0.12**	**0.35**	**0**	**24.3**	**21.75**
SD		0.4	76.587096	0	0.47	0	0	0	0.5	0.33	0.4894	0	10.9	11.4288

Table A: Clinical Data in Low Dose TAC + EVR Vs. TAC + MMF

Subject		
Subject ID	Study Drug	Adverse events / Comments
E-01-R	Tac + EVR	
E-02-R	Tac + EVR	Herpes Zoster (3/2014)
E-03-R	Tac + EVR	Herpes Zoster (6/2013); Erythrocytosis
E-11-R	Tac + EVR	
E-15-R	Tac + EVR	
E-18-R	Tac + EVR	
E-22-R	Tac + EVR	Noncompliance with medication (2/28/14)
E-23-R	Tac + EVR	giardiasis (12/16/2013)
E-26-R	Tac + EVR	
E-27-R	Tac + EVR	no urine creatinine
E-29-R	Tac + EVR	C. diff infection (1/2/2014)
E-30-R	Tac + EVR	UTI (3/20/2014) / switched medication to MPA
E-31-R	Tac + EVR	assuming 2+ is positive for proteinuria
E-33-R	Tac + EVR	
E-34-R	Tac + EVR	withdrawn
E-37-R	Tac + EVR	
E-39-R	Tac + EVR	Hypertension (1/22/2015)
E-40-R	Tac + EVR	ulcerative stomatitis and mucositis, neutropenia (7/15/ 2014)
E-42-R	Tac + EVR	Oral aphthae (7/29/2014) / taken off tacrolimus
E-43-R	Tac + EVR	
**Mean**		
SD		

E-04-R	Tac + MMF	Cholecystitis (7/2014) Only taking tacrolimus, proteinuria only immediately after transplant (prior to measuring immunosuppression medications)
E-05-R	Tac + MMF	Drug induced neutropenia (8/12/2013) / 2+ for protein?
E-06-R	Tac + MMF	
E-07-R	Tac + MMF	
E-09-R	Tac + MMF	
E-10-R	Tac + MMF	BK Viruruia (9/13/2013), cerebrovascular event (8/13/2015)
E-13-R	Tac + MMF	Dyspnea and respiratory abnormalities (8/7/13), UTI (9/5/13, 2/13/14)
E-14-R	Tac + MMF	has not been typed for HLA-DP locus, cannot determine DSA class II
E-16-R	Tac + MMF	cytomegalovirus disease (11/4/2013) / proteinuria only occurred day of transplant
E-17-R	Tac + MMF	UTI (8/28/13, 9/18/13, 10/17/13), Bactremia (10/18/13), Abnormal liver function (9/23/13)
E-19-R	Tac + MMF	BK Nephropathy (11/7/2014) / only proteinuria day after transplant
E-20-R	Tac + MMF	
E-21-R	Tac + MMF	BK Viremia/Viruria (1/14/2014)
E-24-R	Tac + MMF	Hematuria (2/13/2014) / 2+ and 3+
E-25-R	Tac + MMF	UTI (3/12/14, 4/12/2014), acute pyelonephritis (4/13/2014)
E-28-R	Tac + MMF	Neutropenia (2/21/2014
E-32-R	Tac + MMF	
E-38-R	Tac + MMF	Neutropenia (7/10/2014) / no FK since 2014
E-41-R	Tac + MMF	UTI (5/27/2014), pyelonephritis (5/29/2014), neutropenia (9/2/2014) / proteinuria 1 week post transplant
E-44-R	Tac + MMF	Enteritis (Norwalk virus) (4/20/2015)
**Mean**		
SD		

Table B: Histopathological Data in Low Dose TAC + EVR vs. TAC + MMF

	IFTA (in TAC+ EVR Group)							Histopathology (in TAC+ EVR Group)							
**SUBJECT NUMBER**	**IFTA (ci, ct) Degree/%**	**Inflammation assoc with IFTA**	**Composition of infiltrate (LM)**	**Tubulitis of intact tubules within/interface**	**TG (cg)**	**C4d**	**OTHER FINDINGS/DIAGNOSIS**	**GS/Total G/SS**	**Acute glomerulitis (g)**	**Tubulitis NOT in IFTA (t)**	**ATN/ISO Tubular vacuolation**	**Interstitial Inflammation (i)**	**Hyaline arteriosclerosis (ah)**	**Arteriosclerosis (cv)**	**PTC**
**E-02-R**	0%(ci0,ct0)	None	0	0	0(cg0)	Neg(C4d:0)	None	0/9 - 0/9	0(g0)	0(t0)	Moderate /No ISO	0(i0)	0(aho)	0(cv0)	0(ptc:0)
**E-03-R**	10% (Mild; ci1,ct1)	Very mild	Lymphocytes, rare macrophages, many plasma cells	Rare mild tubulitis (1 tubule)	0(cg0)	Neg(C4d:0)	None	1/41 - 0/41	0(g0)	0(t0)	Mild/No ISO	0(i0)	0(ah0)	Focal mild (cv1)	0(ptc:0)
**E-11-R**	20%(Mild; ci1,ct1)	Minimal	lymphocytes	None	0(cg0)	Neg(C4d:0)	Very small subcapsular injury	0/11 - 0/11	0(g0)	0(t0)	Mild/No ISO	0(i0)	0(aho)	0(cv0)	0(ptc:0)
**E-15-R**	5%-10%(Mild; ci1, cti)	Moderate	Many plasma cells, lymphs, few macrophages	Few Mild (t1)	0(cg0)	Neg (C4d:0)	Borderline change	0/17 - 1/17	0(g0)	Moderate (t2)	Moderate /No ISO	Mild(i1)	0(ah0)	Focal mild (cv1)	0(ptc:0)
**E-18-R**	5%-10%(Mild; ci1, ct1)	Focally moderate	Lymphocytes, some macrophages	None	0(cg0)	Neg(C4d:0)	Subcapsular injury	2/48 - 0/48	0(g0)	0(t0)	Mild/No ISO	0(i0)	0(ah0)	0(cv0)	0(ptc:0)
**E-22-R**	0%(ci0, ct0)	None	0	0	0 (cg0)	Neg(C4d:0)	Small subcapsular injury	0/29 - 0/29	0(g0)	0(t0)	Mild/No ISO	0(i0)	0(ah0)	0(cv0)	0(ptc:0)
**E-23-R**	5% - 10%(Mild; ci1,ct1)	Minimal	None	None	0(cg0)	Neg(C4d:0)	None	0/15 - 0/15	0(g0)	0(t0)	Mild/No ISO	0(i0)	0(aho)	0(cv0)	0(ptc:0)
**E-26-R**	0	None	None	None	0(cg0)	Neg(C4d:0)	None	1/35 – 1/35	0(g0)	0(t0)	Mild/No ISO	0(i0)	0(ah0)	0(cv0)	0(ptc:0)
**E-27-R**	0% (ci0,ct0)	None	None	None	0(cg0)	Neg(C4d:0)	None	0/26 – 0/26	0(g0)	0(t0)	Mild/No ISO	0(i0)	0(ah0)	0(cv0)	0(ptc:0)
**E-29-R**	5% 10%(Mild; ci1-ct1)	Focal mild	Lymphocytes, Few macrophages, rare lymphs	Few mild tubulitis (t1). Rare moderate t2 in adjacent area	0(cg0)	Neg (C4d:0)	None	2/60 - 2/60	0(g0)	0(t0)	Mild/No ISO	0(i0 -<10%)	0(ah0)	0(cv0)	0(ptc:0) <10%
**E-30-R**	5%-10% (Mild; ci1-ct1)	Minimal	Lymphocytes	None	0(cg0)	Neg(C4d:0)	None	0/11 - 0/11	0(g0)	0(t0)	Mild/1 focus of Isometric vac	0(i0)	0(aho)	0(cv0)	0(ptc:0)
**E-31-R**	5% (Mild; ci1-ct1)	Mild, very focal	Lymphocytes, rare macro	Mild(t1)-rare	0(cg0)	Neg(C4d:0)	Moderate arterio-sclerosis	1/20 – 1/20	0(g0)	Mild(t1)	Moderate /No ISO	0(i0 - <3%)	0(ah0)	Moderate (cv2)	0(ptc:0) <10%
**E-33-R**	0	None	None	None	0(cg0)	Neg(C4d:0)	None	0/27 – 0/27	0(g0)	0(t0)	Mild/No ISO	0(i0)	0(ah0)	0(cv0)	0(ptc:0)
**E-34-R**	0	None	None	None	0cg0	Neg(C4d:0)	None	0/21 – 0/21	0(g0)	O(t0)	Moderate/NO ISO	0(i0)	0(ah0)	0(cv0)	0(ptc:0)
**E-37-R**	5% (Mild; ci1-ct1)	Minimal	lymphocytes	None	0(cg0)	Neg(C4d:0)	None	1/13 – 0/13	0(g0)	0(t0)	Mild/No !SO	0(i0)	0(ah0)	Mild(cv1)	0(ptc:0)
**E-39-R**	5% (Mild; ci1-ct1)	Mild	Lymphocytes , Many plasma cells	None	0(cg0)	Neg(C4d:0)	Small subcapsular injury	1/15 – 1/15	0(g0)	0(t0)	Mild/No ISO	0(i0)	0(ah0)	Mild(cv1)	0(ptc:0)
**E-40-R**	<5%(Mild)	None	None	None	0(cg0)	Neg(C4d:0)	None	0/15 – 0/15	0(g0)	0(t0)	Mild/No ISO	0(i0)	0(ah0)	Mild(cv1)	0(ptc:0)
**E-42-R**	40%(Moderate; c12,ct2)	Mild - Moderate	Lymphocytes, some macrophages	Mild)t1)	0(cg0)	Neg(C4d:0)	None	3/41 – 0/41	0(cg0)	0(t0)	Mild/No ISO	0(i0)	1(ah1)	0(cv0)	0(ptc:0)
**E-43-R**	10%-20% (Mild; ci1,ct1)	Focally mild	Lymphocytes	Some mild (t1) and moderate (t2) tubulitis	0(cg0)	Neg(C4d:0)	Borderline change; Subcapsular injury	0/31 – 0/31	0(g0)	Focal moderate (t2)	Moderate/No ISO	0(i0)	0(ah0)	0(cv0)	0(ptc:0)
**TOTAL**	19							19							
	None: 5/19 (26%)	None: 7/19 (37%)		t0: 13/19 (68%)	cg0: 19/19 (100%)	cd0: 19/19 (100%)	Borderline change:2/17 (12%)	GS: 8/19 (42%)	g0: 19/19 (100%)	t0: 16/19 (84%)	None: 0/19 (0%)	i0: 18/19 (95%)	aho: 18/19 (95%)	cv0: 13/19 (69%)	9ptc:0: 17/19 (100%)
	Mild: 12/19 (63%)	Minimal: 4/19 (21%)		t1: 4/19 (21%)	cg1: 0/19 (0%)	C4d1: 0/19 (0%)	Subcapsular injury: 5/17 (30%)	FGS: 5/19 (26%)	g1: 0/19 (0%)	t1: 1/19 (5%)	Mild: 14/19 (74%)	i1: 1/19 (5%)	ah1: 1/19 (5%)	cv1: 5/19 (26%)	9ptc1: 0/17 (0%)
	Moderate: 1/19 (5%)	Mild: 5/19 (26%)		t2: 2/19 (11%)	cg2: 0/19 (0%)	C4d2: 0/19 (0%)			g2: 0/19 (0%)	t2: 2/17 (11%)	Moderate: 5/19 (26%)	i2: 0/19 (0%)	ah2: 0/19 (0%)	cv2: 1/19 (5%)	7ptc2: 0/17 (0%)
	Severe: 0/19 (0%)	Moderate: 3/19 (16%)		t3: 0/19 (0%)	cg3: 0/19 (0%)	C4d3: 0/19 (0%)			g3: 0/19 (0%)	t3: 0/19 (0%)	Severe: 0/19 (0%)	i3: 0/19 (0%)	ah3: 0/19 (0%)	cv3: 0/19(0%)	ptc3: 0/19 (0%)
		Severe: 0/19 (0%)									ISO: 1/19 (5%)				

Table B: Histopathological Data in Low Dose TAC + EVR vs. TAC + MMF

	IFTA (in TAC+ MMF Group)							Histopathology (in TAC+ MMF Group)							
**SUBJECT NUMBER**	**IFTA (ci, ct) Degree/%**	**Inflammation assoc with IFTA**	**Composition of infiltrate (LM)**	**Tubulitis of intact tubules within/interface**	**TG (cg)**	**C4d**	**OTHER FINDINGS / DIAGNOSIS**	**GS/Total G/SS**	**Acute glomerulitis (g)**	**Tubulitis NOT in IFTA (t)**	**ATN/ISO Tubular vacuolatiion**	**Interstitial Inflammation (i)**	**Hyaline arteriosclerosis (ah)**	**Arteriosclerosis (cv)**	**PTC**
**E-04-R**	Mild (<5%; ci0,ct0)	mild	Lymphocytes, rare plasma cells, few macrophages	Mild tubulitis (t1) in 2 - 3 tubues	0(cg0)	Neg(C4d:0)	Borderline change (g0,i0,t1, v0)	0/16 – 1/16	0 (g0)	Focal mild (t1)	None	<10% (i0)	0 (ah0)	0 (cv0)	0 (ptc:0)
**E-05-R**	0	None	None	None	0(cg0)	Neg(C4d:0)	---------	0/19 – 0/19	0 (g0)	0(t0)	Mild / No ISO	0 (i0)	0(ah0)	0(cv0)	0(ptc:0)
**E-06-R**	Mild (<5%; ci0ct1)	None	None	None	0(cg0)	Mod-severe (4+) Linear (C4d:3)	Antibody-mediated rejection (Grade 1)	0/35 – 0/35	1 (g1)	0(t0)	Moderate/No ISO	0(i0)	0(ah0)	0(cv0)	focal (ptc:1)
**E-07-R**	0	None	None	None	0(cg0)	Neg(C4d:0)	----------	0/16 – 0/16	0(cg0)	0(t0)	Mild/No ISO	0(i0)	0(ah0)	0(ah0)	0(ptc:0)
**E-09-R**	Mild (5% - 10%; ci1,ct1)	Focal,mild	Some lymphocytes	Rare t1(2)	0(cg0)	Neg(C4d:0)	Subcapsular injury	0/20 – 0/20	0(g0)	0(t0)	None	0(i0)	focal 1(ah1)	0(cv0)	0(ptc:0)
**E-10-R**	Moderate(30% - 40%; ci2, ct2)	Moderate (25% - 50%)	Lymphocytes, rare plasma cell, rare macropahges	Some Mild (t1) and rare moderate (t2)	0(cg0)	2+, diffuse, Linear (C4d3)	Suspicious for antibody-mediated rejection	0/15 – 0/15	1(suspicious g1)	Mild (t1)	None	0(i0)	0(ah0)	0(cv0)	2 (ptc:2) diffuse
**E-13-R**	Mild(<5%; ci0,ct1)	None	None	None	O(cg0)	Neg(C4d:0)	---------	0/36 – 0/36	0(g0)	0(t0)	Mild	0(10)	0(ah0)	Mild(cv1)	0(ptc:0)
**E-14-R**	Mild (10%-20%)	Focal mild	Lymphocyes	Mild tubulitis (1 -2 tubules)	O(cg0)	Neg(C4d:0)	---------	1/14 – 1/14	0(g0)	0(t0)	None	0(i0)	0(ah0)	Focal mild (cv1)	0(ptc:0)
**E-16-R**	Mild(5%-10%; Ci1,ct1)	None	None	None	0(cg0)	4+.diffuse, Linear (C4d:3)	Borderline change (g0, i1, t1, v0) AMR	1/34 – 1/34	0(g0)	Focal mild (t1)	Mild	10%(i1) Some plasma cells	0(ah0)	Focal mild (cv1)	2(ptc:2) diffuse
**E-17-R**	**No record**	--------	--------	--------	--------	-------	--------								
**E-19-R**	Mild(<5%; ci0,ct1)	None	None	None	0(cg0)	Neg(C4d:0)	Polyoma Virus Nerphropathy Class B1	0/19 – 0/19	0(g0)	Mild - moderate (t1 – t2)	Mild/No ISO	10% - 20%	0(ah0)	0(cv0)	focal 1(ptc:1)
**E-21-R**	Mild(<5%; ci0,ct1)	Focally moderate	Mostly lymphs; Few macrophages, Some Plasma cells	None?	0(cg0)	Neg(C4d:0)	--------	1/15 – 1/15	0(g0)	0(t0)	Mild/No ISO	O(i0)	0(ah0)	0(cv0)	ptc:0
**E-24-R**	**No Record**														
**E-25-R**	Mild (<5%)	mild	Lymphocytes, rare macrophages	None	0(cg0)	Neg(C4d:0)	---------	1/18 – 0/18	0(g0)	0(t0)	Mild/No ISO	0(i0)	0(ah0)	0(cv0)	ptc:0
**E-28-R**	Mild(5%-10%; ci1, ct1)	Mild - moderate	Lymphs, few	None	0(cg0)	2+, mild-moderate (C4d:3)	Possible antibody-mediated	1/15 – 1/15	0(g0)?	0(t0)	Mild/No ISO	0(i0) 2 small foci of mild-mononuclear + some monocytoid cells	0(ah0)	0(cv0)	1(ptc:1) (75% of ptc)
**E-32-R**	0	None	None	None	0(cg0)	Neg(C4d:0)	--------	0/15 – 0/15	0(g0)	0(t0)	No ATN /No ISO	0(i0)	0(ah0)	0(cv0)	ptc:0
**E-38-R**	**No Record**														
**E-41-R**	Mild(5%-10%) ci1, ct1	minimal	Few scattered lymphocytes	None	0(cg0)	Negative(C4d:0)	Small Subcapsular injury RBC and RBC casts in tubules	0/11 – 0/11	0(g0)	0(t0)	No ATN/No ISO	0(i0)	0(ah0)	Mild(cv1)	ptc:0
**E-44-R**	0	None	None	None	0(cg0)	Negative(C4d:0)	---------	0/27 – 0/27	0(g0)	0(t0)	Mild/NO ISO	0(i0)	0(ah0)	0(cv0)	ptc:0
**TOTAL**	16							16							
	None: 4/16 (25%)	None: 8/16 (50%)		None: 12/16 (75%)	cg0: 16/16 (100%)	C4d0: 12/16 (75%)	Borderline change: 2/15 (13%)	GS: 5/15 (33%)	g0: 14/16 (94%)	t0: 12/16 (75%)	None: 5/16 (31%)	i0: 14/16 (88%)	ah0: 15/16 (94%)	cv0: 12/16 (75%)	ptc0: 11/16 (69%)
	Mild: 11/16 (69%)	Minimal: 1/16 (6%)		t1: 3/16 (19%)	cg1:0/16 (0%)	C4d1: 0/16 (0%)	Subcapsular injury: 2/15 (13%)	FGS: 4/15 (27%)	g1: 2/16 (13%)	t1: 2/16 (13%)	Mild: 9/16 (56%)	i1: 2/16 (12%)	ah1: 1/16 (6%)	cv1: 4/16 (25%)	ptc1: 3/16 (19%)
	Moderate: 1/16 (6%)	Mild: 4/16 (25%)		t2: 1/16 (6%)	cg2: 0/16 (0%)	C4d2: 0/16 (0%)	Polyoma Virus: 1/15 (7%)		g2: 0/16 (0%)	t2: 1/16 (6%)	Moderate: 1/16 (6%)	i2: 0/15 (0%)	ah2: 0/16 (0%)	cv2: 0/16 (0%)	ptc:2: 2/16 (12%)
	Severe:	Moderate: 3/16 (19%)		t3: 0/15 (0%)	cg3: 0/16 (0%)	C4d3: 4/16 (25%)	AMR: 4/15 (27%)		g3: 0/16 (0%)	t3: 0/15 (0%)	Severe: 0/15 (0%)	i3: 0/15 (0%)	ah3: 0/16 (0%)	cv3: 0/16 (0%)	ptc:3: 0/15 (0%)
		Severe: 0/15 (0%)													
