# Supplementary material for: Mechanistic analyses in kidney transplant recipients prospectively randomized to two steroid free regimen—Low dose Tacrolimus with Everolimus versus standard dose Tacrolimus with Mycophenolate Mofetil
Source: PLoS One. 2019 May 28;14(5):e0216300. doi: 10.1371/journal.pone.0216300 (PMC6538151; doi:10.1371/journal.pone.0216300)
Supplement: S5 File — (PDF) [file pone.0216300.s005.pdf]

Table C showing data presented in Fig 4 on the percentage of $CD4^+CD25^{hi}FOXP3^+$ regulatory T cells in the peripheral blood between study groups

	TAC+EVR			TAC+MMF		
	Baseline	*3mo*	*12mo*	*Baseline*	*3mo*	*12mo*
1	1.51	0.94	2.65	0.74	0.46	0.45
2	0.87	0.61	1.14	1	0.47	1.12
3	0.66	0.71	0.94	1.1	1.82	0.9
4	1.3	1.6	0.96	1.34	0.92	1.11
5	0.67	1.15	0.98	0.75	0.4	0.46
6	0.51	1.54	1.38	0.94	0.51	0.83
7	0.43	1.86	1.11	2.72	0.4	0.65
8	0.34	0.61	1.26	0.59	1.43	1.55
9	0.68	0.5	1.54	1.34		1.01
10	0.53	1.17	0.88	0.21		0.44
11	1.04	1.71	1.3	1.14		0.86
12	0.92	0.54	1.33	0.48		0.38
13	1.14	1.62	0.45	1.33		
14	2.32	1.13	1.18			
15	1.03		0.59			
**Mean**	**0.93**	**1.12**	**1.18**	**1.05**	**0.80**	**0.81**
SE	0.13	0.13	0.13	0.17	0.19	0.10

Table D showing data presented in Fig 5 (top) on the percentage of IFN-γ^+ regulatory T cells in the peripheral blood between study groups (% of $CD4^+CD25^{hi}FOXP3^+$ cells)

	TAC+EVR			TAC+MMF		
	Baseline	*3mo*	*12mo*	*Baseline*	*3mo*	*12mo*
1	50	6.87	19.7	31.3	2.03	17.6
2	18.2	14.3	21	46.5	42	3.33
3	6.21	17.4	46.4	18.7	10.9	5.86
4	10.7	13	22.2	18.5	43.2	6.35
5	30.9	50	31.4	41.3	0	9.26
6	27.4	29.8	23.3	17.9	10.8	7.89
7	15.1	67.1	17.3	12	21.6	14.9
8	21.1	10.4	26.5	6.03	7.09	3.4
9	46.2	25.8	21.4	7.27	0.98	9.96
10	50	21.4	25	37.7		11.4
11	52.9	7.25	21.9	4.15		8.04
12	9.63	38.5	15.9	5.03		9.55
13	4.18	5.35	22.1	3.73		
14	8.29	0.88	23.6			
15	0		16.6			
**Mean**	**23.4**	**22.0**	**23.6**	**19.24**	**15.40**	**8.96**
SE	4.8	5.0	1.9	4.21	5.93	1.23

Table E showing data presented in Fig 5 (middle) on the percentage of IL17⁺ regulatory T cells in the peripheral blood between study groups (% of $CD4^{+}CD25^{hi}FOXP3^{+}$ cells)

	TAC+EVR			TAC+MMF		
	Baseline	*3mo*	*12mo*	*Baseline*	*3mo*	*12mo*
1	0	2.06	0.33	0	0	0
2	0	2.86	1.12	0	1	0
3	0	0	0	0.51	0	0
4	0.51	0.63	0.83	0	0.24	0.32
5	0	0.61	0	0	0	0
6	0	0	0.79	0.51	0	0
7	0	0.26	0	0.21	0	0
8	0	0	0	0	0	1.02
9	0	0	0	0	0	0.41
10	0	0	0	0		0
11	0	0.57	0	0		0
12	0	0	0.29	0		0.32
13	0	0	0	0		
14	0.32	0.44	0			
15	0		0			
**Mean**	**0.06**	**0.53**	**0.22**	**0.09**	**0.14**	**0.17**
SE	0.04	0.23	0.10	0.05	0.12	0.09

Table F showing data presented in Fig 5 (bottom) on the percentage of RORγt⁺ regulatory T cells in the peripheral blood between study groups (% of $CD4^{+}CD25^{hi}FOXP3^{+}$ cells)

	TAC+EVR			TAC+MMF		
	Baseline	*3mo*	*12mo*	*Baseline*	*3mo*	*12mo*
1	50	0.69	0.16	22.3	1.01	0.35
2	6.25	0.57	0.75	2.19	2	41.2
3	3.45	3.72	0.75	2.02	2.56	27.1
4	1.02	0.42	1.11	0.34	12	27.6
5	7.45	19.3	50.5	0.6	0	6.79
6	0	5.32	34	18.9	19.8	7.89
7	0	37.3	23.4	48.3	14.9	6.97
8	0	2.89	29.6	7.91	9.93	6.12
9	4.14	7.28	50.3	7.88	10.1	5.81
10	55.3	40.5	21	6.49		3.96
11	76.5	29.2	33.5	9.73		3.57
12	47.8	52.6	3.47	3.14		3.82
13	36.8	16.9	3.54	6.22		
14	47.5	2.21	3.11			
15	3.26		2.29			
**Mean**	**22.6**	**15.6**	**17.2**	**10.46**	**8.03**	**11.77**
SE	6.8	4.7	4.8	3.65	2.47	3.70

Table G: Gene Functions Data Shown in Figure 6

Figure 6 - Gene Functions

Categories	Diseases or Functions Annotation	p-value	Predicted Activation State	Activation z-score	Molecules	# Molecules
Cellular Movement	migration of cells	9.08E-11	Decreased	-2.004	ABHD6,ADAM10,ADGRL3,ANGPT2,C4A/C4B,CALCRL,CALU,CEACAM1,CHN2,CLIC4,CPNE3,CXCL12,CXCR4,DPT,EDIL3,EDNRA,EFS,FERMT1,FHL1,FLT1,HNRNPL,IQGAP1,ITGB1BP1,JAG1,KCNMA1,LEPR,LYVE1,MAGI1,MCAM,NCOA2,PATJ,PCM1,PDCD4,PDCD6,PDE4DIP,PGGT1B,PROX1,RAPGEF3,S100A4,SEMA6A,SMAD5,SRPX2,STK4,TCF7L2,TG,TGFB2,THBS1,TM4SF1,TNC,VDR,VLDLR,WNT5A	52
Cellular Movement	migration of cancer cells	1.59E-10	Decreased	-2.139	ADAM10,ANGPT2,CEACAM1,CXCL12,CXCR4,FLT1,IQGAP1,MCAM,PROX1,RAPGEF3,TGFB2,THBS1,TM4SF1,TNC,WNT5A	15
Cellular Movement	cell movement of tumor cells	8.28E-10	Decreased	-2.12	ADAM10,ANGPT2,CEACAM1,CXCL12,CXCR4,FLT1,IQGAP1,MCAM,RAPGEF3,TGFB2,THBS1,TM4SF1,TNC,WNT5A	14
Tissue Morphology	quantity of cells	3.97E-09	Decreased	-3.504	ADAM10,ANGPT2,ATRX,C4A/C4B,CALCR,CALCRL,CLIC4,CREB1,CXCL12,CXCR4,DLK1,EDNRA,EIF2S1,ELF1,ENTPD1,FHL5,FLT1,HSF2,ITGB1BP1,JAG1,KIF13A,LEPR,MECOM,MLXIPL,NCOA2,NEFH,NFASC,PDCD6,PLCL2,PTGER2,RAPGEF3,SMAD5,SSBP2,STK4,TANK,TCF7L2,TFRC,TG,TGFB2,THBS1,TNC,UTRN,VDR,VLDLR,WNT5A,ZFX	46
Organ Morphology,Reproductive System Development and Function	quantity of gonad	4.95E-08	Decreased	-2.18	CXCL12,CXCR4,ENTPD1,FHL5,FLT1,HSF2,LEPR,PTGER2,SMAD5,TGFB2,VDR,WNT5A,ZFX	13
Cell-To-Cell Signaling and Interaction,Cellular Assembly and Organization	cell-cell contact	0.00000111	Decreased	-2.692	ADAM10,ADGRL3,ANGPT2,CEACAM1,CREB1,CXCL12,CXCR4,ENTPD1,GABPA,HNF1B,IQGAP1,MBNL1,MBNL2,MCAM,NFASC,RAPGEF3,SRPX2,THBS1,TNC,UTRN,WNT5A	21
Embryonic Development,Organismal Development	development of body trunk	0.0000038	Decreased	-2.006	ADAM10,CALCRL,CREB1,CXCL12,CXCR4,EDNRA,EMP1,FHL1,FILIP1L,FLT1,GABPA,HNF1B,ID4,JAG1,LEPR,LTBP1,MECOM,NCOA2,PDCD4,PROX1,PTGER2,RAPGEF3,SMAD5,STK4,TCF7L2,TGFB2,THBS1,UTRN,VDR,WNT5A,ZFX	31
Organismal Development	development of genitourinary system	0.0000258	Decreased	-2.176	ATRX,CREB1,CXCL12,CXCR4,EDNRA,FHL5,FLT1,GRB10,HNF1B,HSF2,ID4,JAG1,LEPR,MLXIPL,NCOA2,NUPR1,PTGER2,RAPGEF3,SMAD5,STK4,TGFB2,VDR,VLDLR,WNT5A,ZFX	25
Cellular Movement	cell movement of breast cancer cell lines	0.0000428	Decreased	-2.035	ANGPT2,CHN2,CPNE3,CXCL12,CXCR4,IQGAP1,MCAM,PDCD4,S100A4,SENP7,SMAD5,WNT5A	12
Inflammatory Response	inflammatory response	0.0000545	Decreased	-2.488	ADAM10,ANGPT2,CALCRL,CXCL12,CXCR4,CYP4F3,EDNRA,EFS,FLT1,LTBP1,MECOM,NR1D2,NUPR1,PDCD4,PTGER2,RAPGEF3,S100A4,TGFB2,THBS1,TNC,WNT5A	21
Cellular Movement,Immune Cell Trafficking,Lymphoid Tissue Structure and Development	chemotaxis of lymphatic system cells	0.000129	Decreased	-2.543	ADAM10,CXCL12,CXCR4,PROX1,S100A4,THBS1,WNT5A	7

Cell-mediated Immune Response,Cellular Movement,Hematological System Development and Function,Immune Cell Trafficking,Inflammatory Response,Lymphoid Tissue Structure and Development	chemotaxis of T lymphocytes	0.000154	Decreased	-2.366	ADAM10,CXCL12,CXCR4,S100A4,THBS1,WNT5A	6
Gene Expression	transcription	0.000205	Decreased	-2.659	ATRX,C4A/C4B,CPE,CREB1,CXCR4,EIF2S1,ELF1,FERMT1,FHL5,GABPA,HNF1B,HSF2,ID4,IQGAP1,ITGB1BP1,JAG1,LEPR,MDFIC,MECOM,MLXIPL,NANOG,NCOA2,NR1D2,PDCD4,PROX1,PTGER2,RAPGEF3,SMAD5,STK4,TANK,TCF7L2,TG,TGFB2,TNC,VDR,VLDLR,WNT5A,ZNF711	38
Cellular Movement,Hematological System Development and Function,Immune Cell Trafficking,Inflammatory Response	chemotaxis of mononuclear leukocytes	0.000207	Decreased	-2.175	ADAM10,ANGPT2,CXCL12,CXCR4,FLT1,S100A4,THBS1,WNT5A	8
Cell-To-Cell Signaling and Interaction	binding of blood cells	0.000244	Decreased	-2.021	ADAM10,ANGPT2,C4A/C4B,CEACAM1,CXCL12,CXCR4,ENTPD1,FLT1,MCAM,STK4,TFRC,THBS1,TNC	13
Connective Tissue Development and Function,Tissue Development	growth of connective tissue	0.000268	Decreased	-2.187	CEACAM1,CREB1,CXCL12,EDNRA,FERMT1,FLT1,GRB10,KCNMA1,LEPR,MECOM,NUPR1,PGGT1B,PTGER2,SMAD5,TGFB2,VDR,WNT5A	17
Cell-To-Cell Signaling and Interaction	adhesion of blood cells	0.000271	Decreased	-2.607	ADAM10,ANGPT2,C4A/C4B,CEACAM1,CXCL12,CXCR4,ENTPD1,FLT1,MCAM,STK4,THBS1,TNC	12
Cellular Development,Cellular Growth and Proliferation,Nervous System Development and Function,Tissue Development	proliferation of neuronal cells	0.000372	Decreased	-2.193	ADAM10,CREB1,CXCL12,CXCR4,FLT1,ID4,IQGAP1,JAG1,NEFH,S100A4,SEMA6A,THBS1,TNC,VLDLR,WNT5A	15
Gene Expression	expression of RNA	0.00041	Decreased	-2.817	ATRX,CALCR,CPE,CREB1,CXCR4,EIF2S1,ELF1,FERMT1,FHL5,GABPA,HNF1B,HSF2,ID4,IQGAP1,ITGB1BP1,JAG1,LEPR,MAP4K5,MDFIC,MECOM,MLXIPL,NANOG,NCOA2,NR1D2,PDCD4,PROX1,PTGER2,RAPGEF3,SMAD5,STK4,TANK,TCF7L2,TG,TGFB2,TNC,VDR,VLDLR,WNT5A,ZNF711	39
Gene Expression	transcription of RNA	0.000432	Decreased	-2.119	ATRX,CPE,CREB1,CXCR4,EIF2S1,ELF1,FHL5,GABPA,HNF1B,HSF2,ID4,IQGAP1,ITGB1BP1,JAG1,LEPR,MDFIC,MECOM,MLXIPL,NANOG,NCOA2,NR1D2,PDCD4,PROX1,PTGER2,RAPGEF3,SMAD5,STK4,TANK,TCF7L2,TG,VDR,VLDLR,WNT5A,ZNF711	34
Reproductive System Development and Function	fertility	0.000644	Decreased	-2.401	CLIC4,CPE,ENTPD1,FHL5,LEPR,NCOA2,PTGER2,SMAD5,THBS1,VDR,ZFX	11
Cellular Development,Cellular Growth and Proliferation,Nervous System Development and Function,Tissue Development	growth of neurites	0.00073	Decreased	-2.063	ADAM10,CREB1,CXCL12,CXCR4,FLT1,IQGAP1,NEFH,S100A4,SEMA6A,THBS1,TNC,VLDLR,WNT5A	13
Organismal Survival	organismal death	3.36E-09	Increased	2.194	ABCC9,ADAM10,ADAMTS9,ANGPT2,C4A/C4B,CALCR,CALCRL,CEACAM1,CLIC4,CREB1,CXCL12,CXCR4,DLK1,DNAJB4,EDNRA,EIF2S1,FERMT1,FLT1,GABPA,GPX2,HNF1B,HNRNPL,ITGB1BP1,JAG1,KCNMA1,LEPR,LTBP1,MBNL1,MBNL2,MECOM,MLXIPL,NCOA2,NFASC,PDCD4,PDE4DIP,PRKACB,PROZ,PTGER2,S100A4,SLCO2A1,SMAD5,SSBP2,STK4,SYT1,TANK,TCF7L2,TFRC,TGFB2,THBS1,TNC,UTRN,VDR,VLDLR,WNT5A,ZFR,ZFX	56
Cardiovascular Disease,Developmental Disorder,Organismal Injury and Abnormalities	perimembranous ventricular septal defect	8.62E-07	Increased	2.219	CXCL12,CXCR4,EDNRA,LTBP1,TGFB2	5

Cardiovascular Disease,Developmental Disorder	congenital anomaly of cardiovascular system	0.0000203	Increased	2.057	ANGPT2,C4A/C4B,CXCL12,CXCR4,EDNRA,FLT1,JAG1,LTBP1,SMAD5,STK4,TGFB2	11
Cardiovascular Disease,Developmental Disorder,Organismal Injury and Abnormalities	congenital heart disease	0.0000395	Increased	2.412	C4A/C4B,CXCL12,CXCR4,EDNRA,FLT1,JAG1,LTBP1,SMAD5,STK4,TGFB2	10
Cancer,Organismal Injury and Abnormalities,Reproductive System Disease	tumorigenesis of genital organ	1.08E-15			ABCC9,ABHD6,ABO,ADAM10,ADAMTS9,ANGPT2,ATRX,C4A/C4B,CALCR,CALCRL,CALU,CEACAM1,CES2,CHN2,CLIC4,CPE,CPNE3,CREB1,CXCL12,CXCR4,CYP4F2,CYP4F3,DPT,EDIL3,EDNRA,EIF2S1,ELF1,ENPEP,ENTPD1,FAM198B,FERMT1,FGL2,FHL1,FHL5,FILIP1L,FLT1,GPX2,GRB10,HNF1B,HNRNPL,IQGAP1,JAG1,KCNMA1,KIF13A,LEPR,LTBP1,LYVE1,MAGI1,MAP4K5,MAST4,MBNL1,MBNL2,MCAM,ME2,MECOM,MIA2,MUC5AC,NCOA2,NEFH,NFASC,OVGP1,PDCD4,PDE4DIP,PRKACB,PROX1,PTGER2,RAB23,RAPGEF3,RASAL2,RETREG1,RIOK3,S100A4,SEMA4G,SEMA6A,SENP7,SLC13A2,SLC22A18,SLC26A4,SLC39A4,SLCO2A1,SMAD5,SPAG5,SRPX2,SSBP2,STAG2,STK4,TAF13,TES,TFRC,TG,THBS1,THSD7A,TLK1,TM4SF1,TNC,TPCN1,TPM4,TSPAN5,TUBAL3,UBE3B,USH1C,UTRN,VDR,VLDLR,VPS13D,WNT5A,ZFR,ZFX,ZFY,ZNF277,ZNF480,ZNF711	112
Cancer,Organismal Injury and Abnormalities,Reproductive System Disease	genital tumor	1.11E-15			ABCC9,ABHD6,ABO,ADAM10,ADAMTS9,ANGPT2,ATRX,C4A/C4B,CALCR,CALCRL,CALU,CEACAM1,CES2,CHN2,CLIC4,CPE,CPNE3,CREB1,CXCL12,CXCR4,CYP4F2,CYP4F3,DPT,EDIL3,EDNRA,EIF2S1,ELF1,ENPEP,ENTPD1,FAM198B,FERMT1,FGL2,FHL1,FHL5,FILIP1L,FLT1,GPX2,GRB10,HNF1B,HNRNPL,IQGAP1,JAG1,KCNMA1,KIF13A,LEPR,LTBP1,LYVE1,MAGI1,MAP4K5,MAST4,MBNL1,MBNL2,MCAM,ME2,MECOM,MIA2,MUC5AC,NCOA2,NEFH,NFASC,OVGP1,PDCD4,PDE4DIP,PRKACB,PROX1,PTGER2,RAB23,RAPGEF3,RASAL2,RETREG1,RIOK3,S100A4,SEMA4G,SEMA6A,SENP7,SLC13A2,SLC22A18,SLC26A4,SLC39A4,SLCO2A1,SMAD5,SPAG5,SRPX2,SSBP2,STAG2,STK4,TAF13,TES,TFRC,TG,THBS1,THSD7A,TLK1,TM4SF1,TNC,TPCN1,TPM4,TSPAN5,TUBAL3,UBE3B,USH1C,UTRN,VDR,VLDLR,VPS13D,WNT5A,ZFR,ZFX,ZFY,ZNF277,ZNF480,ZNF711	112
Cancer,Organismal Injury and Abnormalities,Reproductive System Disease	genital tract cancer	3.92E-14			ABCC9,ABHD6,ABO,ADAM10,ANGPT2,ATRX,C4A/C4B,CALCR,CALCRL,CALU,CEACAM1,CES2,CHN2,CLIC4,CPE,CPNE3,CREB1,CXCL12,CXCR4,CYP4F2,CYP4F3,DPT,EDIL3,EDNRA,EIF2S1,ELF1,ENPEP,ENTPD1,FAM198B,FERMT1,FGL2,FHL1,FHL5,FILIP1L,FLT1,GPX2,GRB10,HNF1B,HNRNPL,IQGAP1,JAG1,KCNMA1,KIF13A,LEPR,LYVE1,MAGI1,MAP4K5,MAST4,MBNL1,MBNL2,MCAM,ME2,MECOM,MIA2,MUC5AC,NCOA2,NEFH,NFASC,OVGP1,PDCD4,PDE4DIP,PRKACB,PROX1,PTGER2,RASAL2,RETREG1,RIOK3,S100A4,SEMA4G,SEMA6A,SENP7,SLC13A2,SLC22A18,SLC26A4,SLC39A4,SLCO2A1,SMAD5,SPAG5,SRPX2,SSBP2,STAG2,STK4,TAF13,TES,TFRC,TG,THBS1,THSD7A,TLK1,TM4SF1,TNC,TPCN1,TPM4,TSPAN5,TUBAL3,UBE3B,USH1C,UTRN,VDR,VLDLR,VPS13D,WNT5A,ZFR,ZFX,ZFY,ZNF277,ZNF480,ZNF711	108
Cancer,Organismal Injury and Abnormalities	genitourinary tumor	5E-14			ABCC9,ABHD6,ABO,ADAM10,ADAMTS9,ANGPT2,ATRX,C4A/C4B,CALCR,CALCRL,CALU,CEACAM1,CES2,CHN2,CLIC4,CPE,CPNE3,CREB1,CXCL12,CXCR4,CYP4F2,CYP4F3,DPT,EDIL3,EDNRA,EIF2S1,ELF1,ENPEP,ENTPD1,FAM198B,FERMT1,FGL2,FHL1,FHL5,FILIP1L,FLT1,GPX2,GRB10,HNF1B,HNRNPL,IQGAP1,JAG1,KCNMA1,KIF13A,LEPR,LTBP1,LYVE1,MAGI1,MAP4K5,MAST4,MBNL1,MBNL2,MCAM,ME2,MECOM,MIA2,MLXIPL,MUC5AC,NCOA2,NEFH,NFASC,OVGP1,PDCD4,PDE4DIP,PRKACB,PROX1,PTGER2,RAB23,RAPGEF3,RASAL2,RETREG1,RIOK3,S100A4,SEMA4G,SEMA6A,SENP7,SLC13A2,SLC22A18,SLC26A4,SLC39A4,SLCO2A1,SMAD5,SPAG5,SRPX2,SSBP2,STAG2,STK4,SULT1C2,TAF13,TES,TFRC,TG,THBS1,THSD7A,TLK1,TM4SF1,TNC,TPCN1,TPM4,TSPAN5,TUBAL3,UBE3B,USH1C,UTRN,VDR,VLDLR,VPS13D,WNT5A,ZFR,ZFX,ZFY,ZNF277,ZNF480,ZNF711	114

Cancer,Organismal Injury and Abnormalities	pelvic cancer	1.89E-13			ABCC9,ABHD6,ABO,ADAM10,ANGPT2,ATRX,C4A/C4B,CALCR,CALCRL,CALU,CEACAM1,CES2,CHN2,CLIC4,CPE,CPNE3,CREB1,CXCL12,CXCR4,CYP4F2,CYP4F3,DPT,EDIL3,EDNRA,EIF2S1,ELF1,ENPEP,ENTPD1,FAM198B,FERMT1,FGL2,FHL1,FHL5,FILIP1L,FLT1,GPX2,GRB10,HNF1B,HNRNPL,IQGAP1,JAG1,KCNMA1,KIF13A,LEPR,LYVE1,MAGI1,MAP4K5,MAST4,MBNL1,MBNL2,MCAM,ME2,MECOM,MIA2,MUC5AC,NCOA2,NEFH,NFASC,OVGP1,PDCD4,PDE4DIP,PRKACB,PROX1,PTGER2,RASAL2,RETREG1,RIOK3,S100A4,SEMA4G,SEMA6A,SENP7,SLC13A2,SLC22A18,SLC26A4,SLC39A4,SLCO2A1,SMAD5,SPAG5,SRPX2,SSBP2,STAG2,STK4,TAF13,TCF7L2,TES,TFRC,TG,THBS1,THSD7A,TLK1,TM4SF1,TNC,TPCN1,TPM4,TSPAN5,TUBAL3,UBE3B,USH1C,UTRN,VDR,VLDLR,VPS13D,WNT5A,ZFR,ZFX,ZFY,ZNF277,ZNF480,ZNF711	109
Cardiovascular System Development and Function	development of vasculature	6E-13		-0.8	ANGPT2,CALCRL,CEACAM1,CLIC4,CREB1,CXCL12,CXCR4,CYP4F2,DLK1,EDIL3,EDNRA,EMP1,ENPEP,FLT1,HNF1B,ITGB1BP1,JAG1,KCNMA1,LEPR,LTBP1,MCAM,NANOG,PATJ,PDCD6,PROX1,PTGER2,RAPGEF3,S100A4,SEMA6A,SMAD5,SRPX2,STK4,TCF7L2,TGFB2,THBS1,TM4SF1,TNC,VDR,VLDLR,WNT5A	40
Cardiovascular System Development and Function,Organismal Development	angiogenesis	1.15E-12		-0.798	ANGPT2,CALCRL,CEACAM1,CLIC4,CREB1,CXCL12,CXCR4,CYP4F2,EDIL3,EDNRA,EMP1,ENPEP,FLT1,ITGB1BP1,JAG1,KCNMA1,LEPR,LTBP1,MCAM,NANOG,PATJ,PDCD6,PROX1,PTGER2,RAPGEF3,S100A4,SEMA6A,SMAD5,SRPX2,STK4,TCF7L2,TGFB2,THBS1,TM4SF1,TNC,VLDLR,WNT5A	37
Cancer,Organismal Injury and Abnormalities	urogenital cancer	1.36E-12			ABCC9,ABHD6,ABO,ADAM10,ANGPT2,ATRX,C4A/C4B,CALCR,CALCRL,CALU,CEACAM1,CES2,CHN2,CLIC4,CPE,CPNE3,CREB1,CXCL12,CXCR4,CYP4F2,CYP4F3,DPT,EDIL3,EDNRA,EIF2S1,ELF1,ENPEP,ENTPD1,FAM198B,FERMT1,FGL2,FHL1,FHL5,FILIP1L,FLT1,GPX2,GRB10,HNF1B,HNRNPL,IQGAP1,JAG1,KCNMA1,KIF13A,LEPR,LYVE1,MAGI1,MAP4K5,MAST4,MBNL1,MBNL2,MCAM,ME2,MECOM,MIA2,MLXIPL,MUC5AC,NCOA2,NEFH,NFASC,OVGP1,PDCD4,PDE4DIP,PRKACB,PROX1,PTGER2,RASAL2,RETREG1,RIOK3,S100A4,SEMA4G,SEMA6A,SENP7,SLC13A2,SLC22A18,SLC26A4,SLC39A4,SLCO2A1,SMAD5,SPAG5,SRPX2,SSBP2,STAG2,STK4,SULT1C2,TAF13,TES,TFRC,TG,THBS1,THSD7A,TLK1,TM4SF1,TNC,TPCN1,TPM4,TSPAN5,TUBAL3,UBE3B,USH1C,UTRN,VDR,VLDLR,VPS13D,WNT5A,ZFR,ZFX,ZFY,ZNF277,ZNF480,ZNF711	110
Cardiovascular System Development and Function,Organismal Development	vasculogenesis	1.87E-12		-1.079	ANGPT2,CEACAM1,CLIC4,CREB1,CXCL12,CXCR4,CYP4F2,EDIL3,EDNRA,EMP1,FLT1,ITGB1BP1,JAG1,KCNMA1,LEPR,LTBP1,MCAM,NANOG,PATJ,PDCD6,PROX1,PTGER2,S100A4,SEMA6A,SMAD5,SRPX2,STK4,TGFB2,THBS1,TM4SF1,TNC,VLDLR,WNT5A	33
Cellular Movement	cell movement	7.28E-12		-1.304	ABHD6,ADAM10,ADGRL3,ANGPT2,C4A/C4B,CALCRL,CALU,CEACAM1,CHN2,CLIC4,CPNE3,CREB1,CXCL12,CXCR4,DNAJB4,DPT,EDIL3,EDNRA,EFS,ENTPD1,FERMT1,FGL2,FHL1,FLT1,HNRNPL,IQGAP1,ITGB1BP1,JAG1,KCNMA1,LEPR,LYVE1,MAGI1,MCAM,NCOA2,PATJ,PCM1,PDCD4,PDCD6,PDE4DIP,PGGT1B,PROX1,RAPGEF3,S100A4,SEMA6A,SENP7,SMAD5,SRPX2,STK4,TCF7L2,TG,TGFB2,THBS1,TM4SF1,TNC,UTRN,VDR,VLDLR,WNT5A	58
Cancer,Organismal Injury and Abnormalities,Reproductive System Disease	tumorigenesis of reproductive tract	6.14E-11			ABCC9,ABHD6,ADAM10,ADAMTS9,ANGPT2,ATRX,C4A/C4B,CALCRL,CES2,CHN2,CLIC4,CPE,CPNE3,CXCL12,CXCR4,CYP4F2,CYP4F3,DPT,EDNRA,EIF2S1,ELF1,ENPEP,ENTPD1,FAM198B,FGL2,FHL1,FILIP1L,FLT1,GRB10,HNRNPL,IQGAP1,JAG1,KCNMA1,KIF13A,LTBP1,MAP4K5,MAST4,MBNL1,MCAM,MECOM,MUC5AC,NCOA2,NEFH,OVGP1,PDE4DIP,PROX1,PTGER2,RAB23,RAPGEF3,RASAL2,RIOK3,S100A4,SENP7,SLC13A2,SLC22A18,SLC39A4,SLCO2A1,SMAD5,SRPX2,STAG2,TAF13,TES,TFRC,TG,THBS1,TM4SF1,TNC,TPCN1,TPM4,TUBAL3,UBE3B,USH1C,UTRN,VDR,VLDLR,WNT5A,ZFR,ZFX,ZNF277,ZNF480	80
Cancer,Organismal Injury and Abnormalities,Reproductive System Disease	female genital neoplasm	6.43E-11			ABCC9,ABHD6,ADAM10,ADAMTS9,ANGPT2,ATRX,C4A/C4B,CALCRL,CES2,CHN2,CLIC4,CPE,CPNE3,CXCL12,CXCR4,CYP4F2,CYP4F3,DPT,EDNRA,EIF2S1,ELF1,ENPEP,ENTPD1,FAM198B,FGL2,FHL1,FILIP1L,FLT1,GRB10,HNRNPL,IQGAP1,JAG1,KCNMA1,KIF13A,LTBP1,MAP4K5,MAST4,MBNL1,MCAM,MECOM,MUC5AC,NCOA2,NEFH,OVGP1,PDE4DIP,PROX1,PTGER2,RAB23,RAPGEF3,RASAL2,RIOK3,S100A4,SENP7,SLC13A2,SLC22A18,SLC39A4,SLCO2A1,SMAD5,SRPX2,STAG2,TAF13,TES,TFRC,TG,THBS1,TM4SF1,TNC,TPCN1,TPM4,TUBAL3,UBE3B,USH1C,UTRN,VDR,VLDLR,WNT5A,ZFR,ZFX,ZNF277,ZNF480	80

Cellular Movement	cell movement of cancer cells	3.44E-10		-1.716	ADAM10,CEACAM1,CXCL12,CXCR4,FLT1,IQGAP1,MCAM,RAPGEF3,TGFB2,THBS1,TM4SF1,TNC,WNT5A	13
Cancer,Organismal Injury and Abnormalities,Reproductive System Disease	uterine tumor	7.14E-10			ABCC9,ABHD6,ADAM10,ANGPT2,ATRX,CALCRL,CES2,CHN2,CPNE3,CXCL12,CYP4F2,CYP4F3,DPT,EDNRA,EIF2S1,ELF1,ENPEP,ENTPD1,FAM198B,FGL2,FHL1,FILIP1L,FLT1,GRB10,HNRNPL,IQGAP1,JAG1,KCNMA1,KIF13A,LTBP1,MAP4K5,MAST4,MBNL1,MECOM,MUC5AC,NEFH,PDE4DIP,PROX1,RAB23,RAPGEF3,RASAL2,RIOK3,S100A4,SENP7,SLC13A2,SLCO2A1,SMAD5,SRPX2,STAG2,TAF13,TES,TFRC,TG,THBS1,TNC,TPCN1,UBE3B,USH1C,UTRN,VDR,VLDLR,WNT5A,ZFR,ZFX,ZNF277,ZNF480	66
Cancer,Organismal Injury and Abnormalities	benign neoplasia	8.75E-10		1.131	ANGPT2,ATRX,C4A/C4B,CALCRL,CEACAM1,CES2,CXCL12,DPT,EDNRA,ENPEP,FLT1,ID4,IQGAP1,JAG1,LEPR,LTBP1,MCAM,MECOM,PDCD4,PTGER2,RAB23,RAPGEF3,S100A4,SLC39A4,SMAD5,SSBP2,STAG2,TAF13,TG,THBS1,TNC,UTRN,VDR	33
Cancer,Organismal Injury and Abnormalities,Reproductive System Disease	Prostate Cancer and Tumors	8.82E-10			ABCC9,ABO,ADAM10,ATRX,C4A/C4B,CALCR,CALCRL,CALU,CEACAM1,CES2,CPE,CPNE3,CREB1,CXCR4,EDIL3,EDNRA,EIF2S1,ENTPD1,FERMT1,FHL5,FILIP1L,FLT1,GPX2,HNF1B,JAG1,KCNMA1,LEPR,LYVE1,MAGI1,MAP4K5,MAST4,MBNL2,ME2,MECOM,MIA2,MUC5AC,NCOA2,NEFH,NFASC,PDCD4,PDE4DIP,PRKACB,PROX1,RASAL2,RETREG1,RIOK3,SEMA4G,SEMA6A,SENP7,SLC26A4,SMAD5,SPAG5,SSBP2,STAG2,STK4,TAF13,TG,THSD7A,TLK1,TNC,TSPAN5,UBE3B,USH1C,UTRN,VDR,VLDLR,VPS13D,WNT5A,ZFR,ZFY,ZNF277,ZNF711	72
Cancer,Organismal Injury and Abnormalities	genitourinary carcinoma	1.88E-09			ABCC9,ABHD6,ABO,ADAM10,ANGPT2,ATRX,C4A/C4B,CALCR,CALCRL,CALU,CEACAM1,CES2,CHN2,CPE,CPNE3,CREB1,CXCL12,CYP4F2,CYP4F3,EDIL3,EIF2S1,ELF1,ENPEP,ENTPD1,FAM198B,FERMT1,FHL5,FILIP1L,FLT1,GRB10,HNF1B,HNRNPL,IQGAP1,JAG1,KCNMA1,KIF13A,LEPR,LYVE1,MAGI1,MAP4K5,MAST4,MBNL1,MBNL2,MCAM,ME2,MECOM,MIA2,MLXIPL,MUC5AC,NCOA2,NEFH,NFASC,OVGP1,PDCD4,PDE4DIP,PRKACB,PROX1,PTGER2,RASAL2,RETREG1,RIOK3,SEMA4G,SEMA6A,SENP7,SLC13A2,SLC22A18,SLC26A4,SLC39A4,SLCO2A1,SMAD5,SPAG5,SRPX2,SSBP2,STAG2,SULT1C2,TES,TG,THBS1,THSD7A,TLK1,TM4SF1,TNC,TPCN1,TSPAN5,TUBAL3,UBE3B,USH1C,UTRN,VDR,VLDLR,VPS13D,WNT5A,ZFR,ZFX,ZFY,ZNF277,ZNF480,ZNF711	98
Cancer,Organismal Injury and Abnormalities,Reproductive System Disease	prostate cancer	5.11E-09			ABCC9,ABO,ADAM10,ATRX,C4A/C4B,CALCR,CALCRL,CALU,CEACAM1,CES2,CPE,CREB1,CXCR4,EDIL3,EDNRA,EIF2S1,ENTPD1,FERMT1,FHL5,FILIP1L,FLT1,GPX2,HNF1B,KCNMA1,LEPR,LYVE1,MAGI1,MAP4K5,MAST4,MBNL2,ME2,MECOM,MIA2,MUC5AC,NCOA2,NEFH,NFASC,PDCD4,PDE4DIP,PRKACB,PROX1,RASAL2,RETREG1,RIOK3,SEMA4G,SEMA6A,SENP7,SLC26A4,SMAD5,SPAG5,SSBP2,STAG2,STK4,TAF13,TG,THSD7A,TLK1,TNC,TSPAN5,UBE3B,USH1C,UTRN,VDR,VLDLR,VPS13D,WNT5A,ZFR,ZFY,ZNF277,ZNF711	70
Cancer,Organismal Injury and Abnormalities	abdominal neoplasm	5.26E-09		0.611	ABCC9,ABHD6,ABO,ADAM10,ADAMTS9,ALDH6A1,ALLC,ANGPT2,ATRX,BAG2,C4A/C4B,CALCR,CALCRL,CALU,CDC40,CEACAM1,CES2,CHN2,CLIC4,CPE,CPNE3,CREB1,CXCL12,CXCR4,CYP4F2,CYP4F3,DLK1,DNAJB4,DPT,EDIL3,EDNRA,EFS,EIF2S1,ELF1,EMP1,ENOSF1,ENPEP,ENTPD1,FAM198B,FAM216A,FERMT1,FGL2,FHL1,FHL5,FILIP1L,FLT1,GGH,GPX2,GRB10,HNF1B,HNRNPL,ID4,IQGAP1,ITGB1BP1,JAG1,KCNMA1,KIF13A,LEPR,LHPP,LTBP1,LYVE1,MAGI1,MAP4K5,MAST4,MBNL1,MBNL2,MCAM,MDFIC,ME2,MECOM,MIA2,MLXIPL,MUC5AC,NCOA2,NEFH,NFASC,NR1D2,NUPR1,OVGP1,PAMR1,PCM1,PDCD4,PDE4DIP,PGGT1B,PLCL2,PRKACB,PROX1,PROZ,PTGER2,RAB23,RAPGEF3,RASAL2,RETREG1,RIOK3,S100A4,SEMA4G,SEMA6A,SENP7,SLC13A2,SLC22A18,SLC26A4,SLC38A2,SLC39A4,SLCO2A1,SMAD5,SPAG5,SPTLC3,SRPX2,SSBP2,STAG2,STK4,SULT1C2,SYT1,TAF13,TCF7L2,TES,TFRC,TG,TGFB2,THBS1,THSD7A,TLK1,TM4SF1,TMX1,TNC,TPCN1,TPM4,TSPAN5,TUBAL3,UBE3B,USH1C,UTRN,VDR,VLDLR,VPS13D,WNT5A,ZFR,ZFX,ZFY,ZNF277,ZNF480,ZNF711	142

Cancer,Organismal Injury and Abnormalities	cancer of secretory structure	7.03E-09			ABCC9,ABO,ADAM10,AMY1C (includes others),ANGPT2,ATRX,C4A/C4B,CALCR,CALCRL,CALU,CEACAM1,CES2,CPE,CREB1,CXCL12,CXCR4,EDIL3,EDNRA,EIF2S1,ENOSF1,ENTPD1,FERMT1,FHL1,FHL5,FILIP1L,FLT1,GPX2,GRB10,HNF1B,HNRNPL,IQGAP1,KCNMA1,LEPR,LTBP1,LYVE1,MAGI1,MAP4K5,MAST4,MBNL2,MCAM,ME2,MECOM,MIA2,MUC5AC,NCOA2,NEFH,NFASC,PCM1,PDCD4,PDE4DIP,PRKACB,PROX1,RASAL2,RETREG1,RIOK3,SEMA4G,SEMA6A,SENP7,SLC22A18,SLC26A4,SMAD5,SPAG5,SPTLC3,SSBP2,STAG2,STK4,TAF13,TG,TGFB2,THBS1,THSD7A,TLK1,TNC,TSPAN5,UBE3B,USH1C,UTRN,VDR,VLDLR,VPS13D,WNT5A,ZFR,ZFX,ZFY,ZNF277,ZNF480,ZNF711	87
Cancer,Organismal Injury and Abnormalities	abdominal cancer	7.61E-09		0.647	ABCC9,ABHD6,ABO,ADAM10,ADAMTS9,ALDH6A1,ALLC,ANGPT2,ATRX,BAG2,C4A/C4B,CALCR,CALCRL,CALU,CDC40,CEACAM1,CES2,CHN2,CLIC4,CPE,CPNE3,CREB1,CXCL12,CXCR4,CYP4F2,CYP4F3,DLK1,DNAJB4,DPT,EDIL3,EDNRA,EFS,EIF2S1,ELF1,EMP1,ENOSF1,ENPEP,ENTPD1,FAM198B,FAM216A,FERMT1,FGL2,FHL1,FHL5,FILIP1L,FLT1,GGH,GPX2,GRB10,HNF1B,HNRNPL,ID4,IQGAP1,JAG1,KCNMA1,KIF13A,LEPR,LHPP,LTBP1,LYVE1,MAGI1,MAP4K5,MAST4,MBNL1,MBNL2,MCAM,MDFIC,ME2,MECOM,MIA2,MLXIPL,MUC5AC,NCOA2,NEFH,NFASC,NR1D2,NUPR1,OVGP1,PAMR1,PCM1,PDCD4,PDE4DIP,PGGT1B,PLCL2,PRKACB,PROX1,PROZ,PTGER2,RAB23,RAPGEF3,RASAL2,RETREG1,RIOK3,S100A4,SEMA4G,SEMA6A,SENP7,SLC13A2,SLC22A18,SLC26A4,SLC38A2,SLC39A4,SLCO2A1,SMAD5,SPAG5,SPTLC3,SRPX2,SSBP2,STAG2,STK4,SULT1C2,SYT1,TAF13,TCF7L2,TES,TFRC,TG,TGFB2,THBS1,THSD7A,TLK1,TM4SF1,TMX1,TNC,TPCN1,TPM4,TSPAN5,TUBAL3,UBE3B,USH1C,UTRN,VDR,VLDLR,VPS13D,WNT5A,ZFR,ZFX,ZFY,ZNF277,ZNF480,ZNF711	141
Cancer,Organismal Injury and Abnormalities	cancer	9.6E-09		-0.482	ABCC9,ABHD6,ABO,ADAM10,ADAMTS9,ADGRL3,ALDH6A1,ALLC,AMY1C (includes others),ANGPT2,ATRX,BAG2,C4A/C4B,CALCR,CALCRL,CALU,CDC40,CEACAM1,CES2,CHN2,CLIC4,CPE,CPNE3,CREB1,CXCL12,CXCR4,CYP4F2,CYP4F3,DLK1,DNAJB4,DPT,EDIL3,EDNRA,EFS,EIF2S1,ELF1,EMP1,ENOSF1,ENPEP,ENTPD1,FAM198B,FAM216A,FERMT1,FGL2,FHL1,FHL5,FILIP1L,FLT1,GGH,GPX2,GRB10,HIST1H2BK,HNF1B,HNRNPL,HSF2,ID4,IQGAP1,JAG1,KCNMA1,KIF13A,LEPR,LHPP,LTBP1,LYVE1,MAGI1,MAP4K5,MAST4,MBNL1,MBNL2,MCAM,MDFIC,ME2,MECOM,MIA2,MLXIPL,MSMO1,MUC5AC,NANOG,NCOA2,NEFH,NFASC,NR1D2,NUPR1,OVGP1,PAMR1,PCM1,PDCD4,PDCD6,PDE4DIP,PGGT1B,PLCL2,PRKACB,PROX1,PROZ,PTGER2,RAB23,RAPGEF3,RASAL2,RETREG1,RIOK3,RSPH14,S100A4,SEMA4G,SEMA6A,SENP7,SLC13A2,SLC22A18,SLC26A4,SLC38A2,SLC39A4,SLCO2A1,SMAD5,SPAG5,SPTLC3,SRPX2,SSBP2,STAG2,STK4,SULT1C2,SYT1,TAF13,TCF7L2,TES,TFRC,TG,TGFB2,THBS1,THSD7A,TLK1,TM4SF1,TMX1,TNC,TPCN1,TPM4,TSPAN5,TUBAL3,UBE3B,USH1C,UTRN,VDR,VLDLR,VPS13D,WNT5A,ZFR,ZFX,ZFY,ZNF277,ZNF480,ZNF711	149
Cancer,Organismal Injury and Abnormalities,Reproductive System Disease	female genital tract cancer	1.09E-08			ABCC9,ABHD6,ADAM10,ANGPT2,ATRX,C4A/C4B,CES2,CHN2,CLIC4,CPE,CPNE3,CXCL12,CXCR4,CYP4F2,CYP4F3,DPT,EDNRA,EIF2S1,ELF1,ENPEP,ENTPD1,FAM198B,FGL2,FHL1,FILIP1L,FLT1,GRB10,HNRNPL,IQGAP1,JAG1,KCNMA1,KIF13A,MAP4K5,MAST4,MBNL1,MCAM,MECOM,MUC5AC,NCOA2,NEFH,OVGP1,PDE4DIP,PROX1,PTGER2,RASAL2,RIOK3,S100A4,SENP7,SLC13A2,SLC22A18,SLC39A4,SLCO2A1,SRPX2,STAG2,TES,TFRC,TG,THBS1,TM4SF1,TNC,TPCN1,TPM4,TUBAL3,UBE3B,USH1C,UTRN,VDR,VLDLR,WNT5A,ZFR,ZFX,ZNF277,ZNF480	73
Cellular Movement	migration of melanoma cells	1.54E-08		-0.555	ADAM10,CEACAM1,CXCR4,MCAM,RAPGEF3,TNC	6
Organ Morphology,Reproductive System Development and Function,Tissue Morphology	quantity of primordial germ cells	1.54E-08		-1.718	CXCL12,CXCR4,SMAD5,TGFB2,WNT5A,ZFX	6
Cancer,Organismal Injury and Abnormalities,Reproductive System Disease	prostatic carcinoma	2.74E-08			ABCC9,ABO,ADAM10,ATRX,C4A/C4B,CALCR,CALCRL,CALU,CEACAM1,CES2,CPE,CREB1,EDIL3,EIF2S1,ENTPD1,FERMT1,FHL5,FILIP1L,FLT1,HNF1B,KCNMA1,LEPR,LYVE1,MAGI1,MAP4K5,MAST4,MBNL2,ME2,MECOM,MIA2,MUC5AC,NCOA2,NEFH,NFASC,PDCD4,PDE4DIP,PRKACB,PROX1,RASAL2,RETREG1,RIOK3,SEMA4G,SEMA6A,SENP7,SLC26A4,SMAD5,SPAG5,SSBP2,STAG2,TG,THSD7A,TLK1,TNC,UBE3B,USH1C,UTRN,VLDLR,VPS13D,ZFR,ZFY,ZNF277,ZNF711	62

Tissue Development	development of epithelial tissue	3.71E-08		0.622	ANGPT2,ATRX,CEACAM1,CLIC4,CXCL12,CXCR4,CYP4F2,FLT1,HNF1B,ITGB1BP1,JAG1,LEPR,MCAM,NANOG,PDCD6,PROX1,SEMA6A,TGFB2,THBS1,VLDLR,WNT5A	21
Cancer,Organismal Injury and Abnormalities,Reproductive System Disease	uterine cancer	3.84E-08			ABCC9,ABHD6,ADAM10,ANGPT2,ATRX,CES2,CHN2,CPNE3,CXCL12,CYP4F2,CYP4F3,DPT,EDNRA,EIF2S1,ELF1,ENPEP,ENTPD1,FAM198B,FGL2,FHL1,FILIP1L,FLT1,GRB10,HNRNPL,IQGAP1,JAG1,KCNMA1,KIF13A,MAP4K5,MAST4,MBNL1,MECOM,MUC5AC,NEFH,PDE4DIP,PROX1,RASAL2,RIOK3,S100A4,SENP7,SLC13A2,SLCO2A1,SRPX2,STAG2,TES,TFRC,TG,THBS1,TNC,TPCN1,UBE3B,USH1C,UTRN,VDR,VLDLR,WNT5A,ZFR,ZFX,ZNF277,ZNF480	60
Organismal Development	morphology of body cavity	4.52E-08			ABCC9,ADAM10,ADAMTS9,ANGPT2,C4A/C4B,CALCRL,CEACAM1,CREB1,CXCL12,CXCR4,DLK1,EDNRA,EFS,FERMT1,FLT1,GABPA,HNF1B,HNRNPL,IQGAP1,JAG1,LEPR,LTBP1,MBNL1,MBNL2,MECOM,MLXIPL,MUC5AC,NUPR1,PTGER2,RAPGEF3,SLCO2A1,SSBP2,STK4,TCF7L2,TG,TGFB2,THBS1,USH1C,VDR,WNT5A	40
Cancer,Organismal Injury and Abnormalities	growth of malignant tumor	4.92E-08		-0.723	ADAM10,CEACAM1,CREB1,CXCL12,CXCR4,DLK1,ENTPD1,FHL1,JAG1,LEPR,MCAM,PGGT1B,PROX1,TCF7L2,TGFB2,THBS1,TNC,VDR,WNT5A	19
Cancer,Organismal Injury and Abnormalities	solid tumor	5.1E-08		-0.293	ABCC9,ABHD6,ABO,ADAM10,ADAMTS9,ALDH6A1,ALLC,AMY1C (includes others),ANGPT2,ATRX,BAG2,C4A/C4B,CALCR,CALCRL,CALU,CDC40,CEACAM1,CES2,CHN2,CLIC4,CPE,CPNE3,CREB1,CXCL12,CXCR4,CYP4F2,CYP4F3,DLK1,DNAJB4,DPT,EDIL3,EDNRA,EFS,EIF2S1,ELF1,EMP1,ENOSF1,ENPEP,ENTPD1,FAM198B,FAM216A,FERMT1,FGL2,FHL1,FHL5,FILIP1L,FLT1,GGH,GPX2,GRB10,HIST1H2BK,HNF1B,HNRNPL,HSF2,ID4,IQGAP1,ITGB1BP1,JAG1,KCNMA1,KIF13A,LEPR,LHPP,LTBP1,LYVE1,MAGI1,MAP4K5,MAST4,MBNL1,MBNL2,MCAM,MDFIC,ME2,MECOM,MIA2,MLXIPL,MUC5AC,NANOG,NCOA2,NEFH,NFASC,NR1D2,NUPR1,OVGP1,PAMR1,PCM1,PDCD4,PDCD6,PDE4DIP,PGGT1B,PLCL2,PRKACB,PROX1,PROZ,PTGER2,RAB23,RAPGEF3,RASAL2,RETREG1,RIOK3,RSPH14,S100A4,SEMA4G,SEMA6A,SENP7,SLC13A2,SLC22A18,SLC26A4,SLC38A2,SLC39A4,SLCO2A1,SMAD5,SPAG5,SPTLC3,SRPX2,SSBP2,STAG2,STK4,SULT1C2,SYT1,TAF13,TCF7L2,TES,TFRC,TG,TGFB2,THBS1,THSD7A,TLK1,TM4SF1,TMX1,TNC,TPCN1,TPM4,TSPAN5,TUBAL3,UBE3B,USH1C,UTRN,VDR,VLDLR,VPS13D,WNT5A,ZFR,ZFX,ZFY,ZNF277,ZNF480,ZNF711	148
Cardiovascular System Development and Function,Cellular Movement	cell movement of endothelial cells	6.48E-08		-1.4	ADAM10,ANGPT2,CEACAM1,CXCL12,CXCR4,EDIL3,FLT1,ITGB1BP1,MCAM,PDCD6,PROX1,RAPGEF3,S100A4,TG,TGFB2,THBS1,WNT5A	17
Cardiovascular System Development and Function,Tissue Development	development of endothelial tissue	7.31E-08		0.622	ANGPT2,CEACAM1,CLIC4,CXCL12,CXCR4,CYP4F2,FLT1,ITGB1BP1,MCAM,NANOG,PDCD6,PROX1,SEMA6A,TGFB2,THBS1,VLDLR,WNT5A	17
Cancer,Organismal Injury and Abnormalities,Reproductive System Disease	benign neoplasm of female genital organ	7.49E-08			ATRX,C4A/C4B,CALCRL,DPT,ENPEP,IQGAP1,LTBP1,MCAM,MECOM,PTGER2,RAB23,RAPGEF3,SLC39A4,SMAD5,STAG2,TAF13,TNC,UTRN,VDR	19
Cancer,Organismal Injury and Abnormalities	advanced malignant tumor	7.85E-08		-1.897	ADAM10,ANGPT2,C4A/C4B,CREB1,CXCL12,CXCR4,EDNRA,EFS,EMP1,ENPEP,FLT1,JAG1,KCNMA1,MAGI1,MAP4K5,MCAM,MECOM,NANOG,PDCD4,PROX1,PTGER2,S100A4,SLC39A4,TCF7L2,TGFB2,THBS1,TM4SF1,TNC,VDR,WNT5A	30

Cancer,Organismal Injury and Abnormalities	non-melanoma solid tumor	8.77E-08		-0.347	ABCC9,ABHD6,ABO,ADAM10,ADAMTS9,ALDH6A1,ALLC,AMY1C (includes others),ANGPT2,ATRX,BAG2,C4A/C4B,CALCR,CALCRL,CALU,CDC40,CEACAM1,CES2,CHN2,CPE,CPNE3,CREB1,CXCL12,CXCR4,CYP4F2,CYP4F3,DLK1,DNAJB4,DPT,EDIL3,EDNRA,EFS,EIF2S1,ELF1,ENOSF1,ENPEP,ENTPD1,FAM198B,FAM216A,FERMT1,FGL2,FHL1,FHL5,FILIP1L,FLT1,GGH,GPX2,GRB10,HIST1H2BK,HNF1B,HNRNPL,ID4,IQGAP1,JAG1,KCNMA1,KIF13A,LEPR,LHPP,LTBP1,LYVE1,MAGI1,MAP4K5,MAST4,MBNL1,MBNL2,MCAM,MDFIC,ME2,MECOM,MIA2,MLXIPL,MUC5AC,NANOG,NCOA2,NEFH,NFASC,NR1D2,NUPR1,OVGP1,PAMR1,PCM1,PDCD4,PDCD6,PDE4DIP,PGGT1B,PLCL2,PRKACB,PROX1,PROZ,PTGER2,RAB23,RAPGEF3,RASAL2,RETREG1,RIOK3,RSPH14,S100A4,SEMA4G,SEMA6A,SENP7,SLC13A2,SLC22A18,SLC26A4,SLC38A2,SLC39A4,SLCO2A1,SMAD5,SPAG5,SPTLC3,SRPX2,SSBP2,STAG2,SULT1C2,SYT1,TAF13,TCF7L2,TES,TFRC,TG,TGFB2,THBS1,THSD7A,TLK1,TM4SF1,TMX1,TNC,TPCN1,TSPAN5,TUBAL3,UBE3B,USH1C,UTRN,VDR,VLDLR,VPS13D,WNT5A,ZFR,ZFX,ZFY,ZNF277,ZNF480,ZNF711	142
Cancer,Organismal Injury and Abnormalities	malignant solid tumor	8.84E-08		-0.029	ABCC9,ABHD6,ABO,ADAM10,ADAMTS9,ALDH6A1,ALLC,AMY1C (includes others),ANGPT2,ATRX,BAG2,C4A/C4B,CALCR,CALCRL,CALU,CDC40,CEACAM1,CES2,CHN2,CLIC4,CPE,CPNE3,CREB1,CXCL12,CXCR4,CYP4F2,CYP4F3,DLK1,DNAJB4,DPT,EDIL3,EDNRA,EFS,EIF2S1,ELF1,EMP1,ENOSF1,ENPEP,ENTPD1,FAM198B,FAM216A,FERMT1,FGL2,FHL1,FHL5,FILIP1L,FLT1,GGH,GPX2,GRB10,HIST1H2BK,HNF1B,HNRNPL,HSF2,ID4,IQGAP1,JAG1,KCNMA1,KIF13A,LEPR,LHPP,LTBP1,LYVE1,MAGI1,MAP4K5,MAST4,MBNL1,MBNL2,MCAM,MDFIC,ME2,MECOM,MIA2,MLXIPL,MUC5AC,NANOG,NCOA2,NEFH,NFASC,NR1D2,NUPR1,OVGP1,PAMR1,PCM1,PDCD4,PDCD6,PDE4DIP,PGGT1B,PLCL2,PRKACB,PROX1,PROZ,PTGER2,RAB23,RAPGEF3,RASAL2,RETREG1,RIOK3,RSPH14,S100A4,SEMA4G,SEMA6A,SENP7,SLC13A2,SLC22A18,SLC26A4,SLC38A2,SLC39A4,SLCO2A1,SMAD5,SPAG5,SPTLC3,SRPX2,SSBP2,STAG2,STK4,SULT1C2,SYT1,TAF13,TCF7L2,TES,TFRC,TG,TGFB2,THBS1,THSD7A,TLK1,TM4SF1,TMX1,TNC,TPCN1,TPM4,TSPAN5,TUBAL3,UBE3B,USH1C,UTRN,VDR,VLDLR,VPS13D,WNT5A,ZFR,ZFX,ZFY,ZNF277,ZNF480,ZNF711	147
Cancer,Organismal Injury and Abnormalities	growth of tumor	9.12E-08		-0.813	ADAM10,ANGPT2,CEACAM1,CREB1,CXCL12,CXCR4,DLK1,ENTPD1,FHL1,FLT1,IQGAP1,JAG1,LEPR,MCAM,NCOA2,PGGT1B,PROX1,PTGER2,SMAD5,TCF7L2,TG,TGFB2,THBS1,TMX1,TNC,VDR,WNT5A	27
Tissue Development	growth of epithelial tissue	9.92E-08		0.24	ANGPT2,CEACAM1,CXCL12,CXCR4,CYP4F2,EDNRA,FERMT1,FLT1,HNF1B,ITGB1BP1,LEPR,MCAM,NANOG,NCOA2,PDCD6,PROX1,PTGER2,SEMA6A,TCF7L2,TGFB2,THBS1,VDR,VLDLR,WNT5A	24
Cardiovascular System Development and Function,Organ Morphology,Organismal Development	morphology of heart	1.01E-07			ABCC9,ADAM10,ADAMTS9,ANGPT2,CALCRL,CREB1,CXCL12,CXCR4,EDNRA,FLT1,JAG1,LEPR,LTBP1,MBNL1,MBNL2,MECOM,RAPGEF3,SLCO2A1,STK4,TG,TGFB2,THBS1,VDR	23
Cardiovascular System Development and Function	morphology of cardiovascular system	1.08E-07			ABCC9,ADAM10,ADAMTS9,ANGPT2,CALCRL,CLIC4,CREB1,CXCL12,CXCR4,EDNRA,FLT1,ITGB1BP1,JAG1,LEPR,LTBP1,MBNL1,MBNL2,MECOM,RAPGEF3,SLCO2A1,SMAD5,STK4,TFRC,TG,TGFB2,THBS1,VDR	27
Organismal Injury and Abnormalities,Reproductive System Disease	abnormal growth in endometrium	1.12E-07			ABCC9,ABHD6,ANGPT2,ATRX,CES2,CHN2,CPNE3,CXCL12,CYP4F2,CYP4F3,DPT,EDNRA,EIF2S1,ELF1,ENPEP,ENTPD1,FAM198B,FGL2,FHL1,FILIP1L,GRB10,HNRNPL,IQGAP1,JAG1,KCNMA1,KIF13A,MAP4K5,MAST4,MBNL1,MECOM,MUC5AC,NEFH,PDE4DIP,PROX1,PTGER2,RASAL2,RIOK3,S100A4,SENP7,SLC13A2,SRPX2,STAG2,TES,TG,THBS1,TNC,TPCN1,UBE3B,USH1C,UTRN,VDR,VLDLR,ZFR,ZFX,ZNF277,ZNF480	56
Organismal Injury and Abnormalities,Reproductive System Disease	abnormality of endometrium	1.21E-07			ABCC9,ABHD6,ANGPT2,ATRX,CES2,CHN2,CPNE3,CXCL12,CYP4F2,CYP4F3,DPT,EDNRA,EIF2S1,ELF1,ENPEP,ENTPD1,FAM198B,FGL2,FHL1,FILIP1L,GRB10,HNRNPL,IQGAP1,JAG1,KCNMA1,KIF13A,MAP4K5,MAST4,MBNL1,MECOM,MUC5AC,NEFH,PDE4DIP,PROX1,PTGER2,RASAL2,RIOK3,S100A4,SENP7,SLC13A2,SRPX2,STAG2,TES,TG,THBS1,TNC,TPCN1,UBE3B,USH1C,UTRN,VDR,VLDLR,ZFR,ZFX,ZNF277,ZNF480	56
Embryonic Development,Organismal Development	development of head	1.46E-07		-0.673	ADAM10,ADAMTS9,ATRX,CLIC4,CREB1,CXCL12,CXCR4,DPT,EDNRA,HNF1B,ID4,JAG1,LYVE1,MECOM,MUC5AC,NEFH,PROX1,RAPGEF3,S100A4,SEMA6A,SMAD5,TCF7L2,TG,TGFB2,THBS1,TM4SF1,TNC,USH1C,VLDLR,WNT5A	30

Cardiovascular System Development and Function,Cellular Development,Cellular Function and Maintenance,Cellular Growth and Proliferation,Organismal Development,Tissue Development	proliferation of endothelial cells	0.00000019		0.652	ANGPT2,CEACAM1,CXCL12,CYP4F2,FLT1,ITGB1BP1,MCAM,NANOG,PDCD6,PROX1,SEMA6A,TGFB2,THBS1,VLDLR,WNT5A	15
Nervous System Development and Function	morphology of nervous system	2.01E-07			ADAM10,ATRX,CLIC4,CREB1,CXCL12,CXCR4,EDNRA,ENTPD1,HSF2,IQGAP1,JAG1,LEPR,MBNL1,MBNL2,MECOM,NEFH,NFASC,PRKACB,RETREG1,S100A4,SLC26A4,SMAD5,TCF7L2,TGFB2,USH1C,UTRN,VLDLR,WNT5A	28
Cancer,Organismal Injury and Abnormalities,Reproductive System Disease	endometrial cancer	2.01E-07			ABCC9,ABHD6,ANGPT2,ATRX,CES2,CHN2,CPNE3,CXCL12,CYP4F2,CYP4F3,DPT,EDNRA,EIF2S1,ELF1,ENPEP,ENTPD1,FAM198B,FGL2,FHL1,FILIP1L,GRB10,HNRNPL,IQGAP1,JAG1,KCNMA1,KIF13A,MAP4K5,MAST4,MBNL1,MECOM,MUC5AC,NEFH,PDE4DIP,PROX1,RASAL2,RIOK3,S100A4,SENP7,SLC13A2,SRPX2,STAG2,TES,TG,THBS1,TNC,TPCN1,UBE3B,USH1C,UTRN,VDR,VLDLR,ZFR,ZFX,ZNF277,ZNF480	55
Cancer,Organismal Injury and Abnormalities,Skeletal and Muscular Disorders	muscle tumor	2.16E-07			ANGPT2,CALCR,CALCRL,DPT,FERMT1,FLT1,IQGAP1,LTBP1,MUC5AC,NCOA2,OVGP1,PDE4DIP,PTGER2,RAB23,RAPGEF3,SLC22A18,SLC39A4,SMAD5,TAF13,TG,TNC,UTRN,VDR	23
Cardiovascular System Development and Function,Cellular Movement	chemotaxis of endothelial cells	0.00000022		-0.765	ADAM10,ANGPT2,CEACAM1,CXCL12,CXCR4,PROX1,THBS1	7
Cancer,Organismal Injury and Abnormalities,Reproductive System Disease	prostatic adenocarcinoma	2.25E-07			ABCC9,ABO,ADAM10,ATRX,C4A/C4B,CALCR,CALCRL,CALU,CEACAM1,CES2,CPE,CREB1,EDIL3,EIF2S1,ENTPD1,FERMT1,FHL5,FILIP1L,FLT1,KCNMA1,LEPR,LYVE1,MAGI1,MAST4,MBNL2,ME2,MECOM,MIA2,MUC5AC,NEFH,NFASC,PDCD4,PDE4DIP,PRKACB,PROX1,RASAL2,RETREG1,RIOK3,SEMA4G,SEMA6A,SENP7,SLC26A4,SMAD5,SPAG5,SSBP2,STAG2,TG,THSD7A,TLK1,TNC,UBE3B,USH1C,UTRN,VLDLR,VPS13D,ZFY,ZNF277,ZNF711	58
Cancer,Organismal Injury and Abnormalities	connective or soft tissue tumor	2.62E-07		1.121	ANGPT2,ATRX,CALCR,CALCRL,CREB1,CXCL12,DPT,FERMT1,FLT1,IQGAP1,LEPR,LTBP1,MUC5AC,NCOA2,OVGP1,PDE4DIP,PTGER2,RAB23,RAPGEF3,S100A4,SLC22A18,SLC39A4,SMAD5,SSBP2,STAG2,TAF13,TG,THBS1,TNC,UTRN,VDR	31
Cancer,Organismal Injury and Abnormalities	head and neck cancer	2.62E-07			ABCC9,ABO,ADAM10,ADAMTS9,ALLC,AMY1C (includes others),ANGPT2,ATRX,CALCR,CES2,CREB1,CXCL12,CXCR4,DPT,EDIL3,ENPEP,FAM198B,FHL5,FLT1,MAGI1,MAP4K5,MECOM,MIA2,MUC5AC,NANOG,NEFH,NR1D2,OVGP1,PCM1,PDE4DIP,PRKACB,PROX1,S100A4,SEMA6A,SSBP2,STAG2,TAF13,TG,THBS1,THSD7A,TLK1,TM4SF1,TNC,UTRN,WNT5A,ZFX	46
Cardiovascular System Development and Function,Cellular Development,Cellular Function and Maintenance,Cellular Growth and Proliferation,Organismal Development,Tissue Development	endothelial cell development	2.91E-07		0.667	ANGPT2,CEACAM1,CLIC4,CXCL12,CYP4F2,FLT1,ITGB1BP1,MCAM,NANOG,PDCD6,PROX1,SEMA6A,TGFB2,THBS1,VLDLR,WNT5A	16
Cancer,Organismal Injury and Abnormalities	head and neck neoplasia	3.38E-07			ABCC9,ABO,ADAM10,ADAMTS9,ALLC,AMY1C (includes others),ANGPT2,ATRX,CALCR,CES2,CREB1,CXCL12,CXCR4,DPT,EDIL3,ENOSF1,ENPEP,FAM198B,FGL2,FHL5,FILIP1L,FLT1,ID4,JAG1,LEPR,MAGI1,MAP4K5,ME2,MECOM,MIA2,MUC5AC,NANOG,NEFH,NR1D2,OVGP1,PCM1,PDCD6,PDE4DIP,PRKACB,PROX1,RASAL2,S100A4,SEMA6A,SLCO2A1,SMAD5,SPTLC3,SSBP2,STAG2,SULT1C2,TAF13,TG,THBS1,THSD7A,TLK1,TM4SF1,TNC,UTRN,VDR,WNT5A,ZFX,ZNF480	61

Cancer,Hematological Disease,Organismal Injury and Abnormalities	hematological neoplasia	4.19E-07		0.946	ABCC9,ADAM10,ADAMTS9,AMY1C (includes others),ANGPT2,ATRX,BAG2,C4A/C4B,CALCRL,CXCL12,CXCR4,DPT,FERMT1,FILIP1L,FLT1,HIST1H2BK,HNF1B,ID4,IQGAP1,KCNMA1,LTBP1,MAGI1,MAP4K5,MCAM,MECOM,MIA2,MLXIPL,MSMO1,MUC5AC,NCOA2,NFASC,PAMR1,PDCD4,PDE4DIP,PGGT1B,PLCL2,PRKACB,RASAL2,RSPH14,S100A4,SLC13A2,SMAD5,SSBP2,STAG2,TAF13,TCF7L2,TFRC,TGFB2,THBS1,TLK1,TNC,VPS13D,ZNF277,ZNF711	54
Cancer,Organismal Injury and Abnormalities	carcinoma	4.49E-07		-0.339	ABCC9,ABHD6,ABO,ADAM10,ADAMTS9,ALDH6A1,ALLC,AMY1C (includes others),ANGPT2,ATRX,BAG2,C4A/C4B,CALCR,CALCRL,CALU,CDC40,CEACAM1,CES2,CHN2,CPE,CPNE3,CREB1,CXCL12,CXCR4,CYP4F2,CYP4F3,DLK1,DNAJB4,DPT,EDIL3,EDNRA,EFS,EIF2S1,ELF1,ENOSF1,ENPEP,ENTPD1,FAM198B,FAM216A,FERMT1,FGL2,FHL1,FHL5,FILIP1L,FLT1,GGH,GPX2,GRB10,HNF1B,HNRNPL,ID4,IQGAP1,JAG1,KCNMA1,KIF13A,LEPR,LHPP,LTBP1,LYVE1,MAGI1,MAP4K5,MAST4,MBNL1,MBNL2,MCAM,MDFIC,ME2,MECOM,MIA2,MLXIPL,MUC5AC,NCOA2,NEFH,NFASC,NR1D2,NUPR1,OVGP1,PAMR1,PCM1,PDCD4,PDCD6,PDE4DIP,PGGT1B,PLCL2,PRKACB,PROX1,PROZ,PTGER2,RAB23,RAPGEF3,RASAL2,RETREG1,RIOK3,S100A4,SEMA4G,SEMA6A,SENP7,SLC13A2,SLC22A18,SLC26A4,SLC38A2,SLC39A4,SLCO2A1,SMAD5,SPAG5,SPTLC3,SRPX2,SSBP2,STAG2,SULT1C2,SYT1,TAF13,TCF7L2,TES,TFRC,TG,TGFB2,THBS1,THSD7A,TLK1,TM4SF1,TMX1,TNC,TPCN1,TSPAN5,TUBAL3,UBE3B,USH1C,UTRN,VDR,VLDLR,VPS13D,WNT5A,ZFR,ZFX,ZFY,ZNF277,ZNF480,ZNF711	139
Cardiovascular System Development and Function	morphology of vessel	5.27E-07			ANGPT2,CALCRL,CXCL12,CXCR4,EDNRA,FLT1,HNF1B,ITGB1BP1,JAG1,LTBP1,SMAD5,STK4,TGFB2,THBS1,VDR	15
Cancer,Gastrointestinal Disease,Organismal Injury and Abnormalities	digestive organ tumor	5.39E-07		0.353	ABCC9,ABO,ADAM10,ADAMTS9,ALDH6A1,ALLC,ANGPT2,ATRX,BAG2,CALCR,CALCRL,CALU,CDC40,CEACAM1,CES2,CHN2,CPE,CPNE3,CREB1,CXCL12,CXCR4,CYP4F2,DLK1,DNAJB4,DPT,EDIL3,EDNRA,EFS,ELF1,EMP1,ENOSF1,ENPEP,ENTPD1,FAM198B,FAM216A,FERMT1,FGL2,FHL1,FHL5,FILIP1L,FLT1,GGH,GPX2,GRB10,HNRNPL,ID4,IQGAP1,ITGB1BP1,JAG1,KCNMA1,KIF13A,LEPR,LHPP,LTBP1,LYVE1,MAGI1,MAP4K5,MAST4,MBNL1,MBNL2,MCAM,MDFIC,ME2,MECOM,MIA2,MLXIPL,MUC5AC,NCOA2,NEFH,NFASC,NR1D2,NUPR1,OVGP1,PAMR1,PCM1,PDCD4,PDE4DIP,PGGT1B,PLCL2,PRKACB,PROX1,PROZ,RAB23,RAPGEF3,RASAL2,RIOK3,S100A4,SEMA4G,SEMA6A,SENP7,SLC13A2,SLC22A18,SLC26A4,SLC38A2,SLC39A4,SLCO2A1,SPAG5,SPTLC3,SRPX2,SSBP2,STAG2,STK4,SULT1C2,SYT1,TAF13,TCF7L2,TES,TFRC,TG,TGFB2,THBS1,THSD7A,TLK1,TM4SF1,TMX1,TNC,TPCN1,TSPAN5,TUBAL3,UBE3B,USH1C,UTRN,VDR,VLDLR,VPS13D,WNT5A,ZFR,ZFX,ZFY,ZNF277,ZNF480,ZNF711	132
Cancer,Neurological Disease,Organismal Injury and Abnormalities	central nervous system solid tumor	5.78E-07		-1.342	ALLC,ANGPT2,ATRX,CXCL12,CXCR4,DLK1,DPT,FLT1,JAG1,KCNMA1,LHPP,MAP4K5,MECOM,MUC5AC,NANOG,NEFH,PDE4DIP,RSPH14,S100A4,SMAD5,SPTLC3,STAG2,SULT1C2,TAF13,TCF7L2,TGFB2,THBS1,TNC,USH1C,WNT5A,ZFX,ZNF480	32
Cellular Development,Connective Tissue Development and Function,Tissue Development	differentiation of connective tissue cells	6.32E-07		-1.326	ALDH6A1,CALCR,CEACAM1,CREB1,DLK1,EDNRA,ID4,JAG1,MECOM,PLCL2,PTGER2,RAB23,RAPGEF3,SMAD5,STK4,TCF7L2,TFRC,TGFB2,TNC,TPM4,VDR,WNT5A	22
Cardiovascular System Development and Function	morphology of vasculature	6.65E-07			ANGPT2,CALCRL,CLIC4,CREB1,CXCL12,CXCR4,DLK1,EDNRA,FLT1,ITGB1BP1,LTBP1,SMAD5,STK4,TGFB2,THBS1,VDR	16
Cancer,Organismal Injury and Abnormalities	neoplasia of cells	6.94E-07		-1.204	ABCC9,ABO,ADAM10,ADAMTS9,ALLC,ANGPT2,ATRX,CALCR,CEACAM1,CES2,CHN2,CREB1,CXCL12,CXCR4,CYP4F3,DNAJB4,DPT,EDIL3,ENOSF1,ENPEP,FAM198B,FERMT1,FGL2,FHL5,FILIP1L,GPX2,GRB10,JAG1,KCNMA1,LEPR,MAGI1,MAST4,MBNL1,MBNL2,MCAM,ME2,MECOM,MIA2,MUC5AC,NANOG,NEFH,NR1D2,NUPR1,OVGP1,PCM1,PDCD4,PDE4DIP,PROX1,PTGER2,RASAL2,RETREG1,S100A4,SEMA4G,SEMA6A,SLCO2A1,SSBP2,STAG2,TCF7L2,TG,THBS1,THSD7A,TM4SF1,TNC,USH1C,UTRN,WNT5A,ZNF711	67

Digestive System Development and Function,Gastrointestinal Disease,Organ Morphology,Organismal Development,Organismal Injury and Abnormalities	abnormal morphology of colon	8.21E-07			CEACAM1,FERMT1,TCF7L2,USH1C,VDR,WNT5A	6
Cancer,Gastrointestinal Disease,Organismal Injury and Abnormalities	digestive system cancer	8.56E-07		0.503	ABCC9,ABO,ADAM10,ADAMTS9,ALDH6A1,ALLC,ANGPT2,ATRX,BAG2,CALCR,CALCRL,CALU,CDC40,CEACAM1,CES2,CHN2,CPE,CPNE3,CREB1,CXCL12,CXCR4,CYP4F2,DLK1,DNAJB4,DPT,EDIL3,EDNRA,EFS,ELF1,EMP1,ENOSF1,ENPEP,ENTPD1,FAM198B,FAM216A,FERMT1,FHL1,FHL5,FILIP1L,FLT1,GGH,GPX2,GRB10,HNRNPL,ID4,IQGAP1,JAG1,KCNMA1,KIF13A,LEPR,LHPP,LTBP1,LYVE1,MAGI1,MAP4K5,MAST4,MBNL1,MBNL2,MCAM,MDFIC,ME2,MECOM,MIA2,MLXIPL,MUC5AC,NCOA2,NEFH,NFASC,NR1D2,NUPR1,OVGP1,PAMR1,PCM1,PDCD4,PDE4DIP,PGGT1B,PLCL2,PRKACB,PROX1,PROZ,RAB23,RAPGEF3,RASAL2,RIOK3,S100A4,SEMA4G,SEMA6A,SENP7,SLC13A2,SLC22A18,SLC26A4,SLC38A2,SLC39A4,SLCO2A1,SPAG5,SPTLC3,SRPX2,SSBP2,STAG2,STK4,SULT1C2,SYT1,TAF13,TCF7L2,TES,TFRC,TG,TGFB2,THBS1,THSD7A,TLK1,TM4SF1,TMX1,TNC,TPCN1,TSPAN5,TUBAL3,UBE3B,USH1C,UTRN,VDR,VLDLR,VPS13D,WNT5A,ZFR,ZFX,ZFY,ZNF277,ZNF480,ZNF711	130
Cancer,Organismal Injury and Abnormalities	metastasis	9.07E-07		-1.897	ADAM10,ANGPT2,C4A/C4B,CREB1,CXCL12,CXCR4,EDNRA,EFS,EMP1,FLT1,JAG1,MAGI1,MAP4K5,MCAM,NANOG,PDCD4,PROX1,PTGER2,S100A4,TCF7L2,TGFB2,THBS1,TM4SF1,TNC,VDR,WNT5A	26
Cancer,Hematological Disease,Immunological Disease,Organismal Injury and Abnormalities	lymphoproliferative malignancy	0.00000101		1.757	ABCC9,ADAM10,ADAMTS9,AMY1C (includes others),ANGPT2,ATRX,BAG2,C4A/C4B,CALCRL,CXCL12,CXCR4,DPT,FERMT1,FILIP1L,FLT1,HIST1H2BK,HNF1B,ID4,IQGAP1,LTBP1,MAGI1,MAP4K5,MCAM,MECOM,MIA2,MLXIPL,MSMO1,MUC5AC,NCOA2,NFASC,PAMR1,PDCD4,PDE4DIP,PLCL2,RASAL2,RSPH14,S100A4,SLC13A2,SMAD5,SSBP2,STAG2,TAF13,TGFB2,THBS1,TLK1,TNC,VPS13D,ZNF277,ZNF711	49
Cancer,Organismal Injury and Abnormalities,Reproductive System Disease	female genital tract adenocarcinoma	0.00000123			ABCC9,ABHD6,ANGPT2,ATRX,C4A/C4B,CES2,CHN2,CPE,CPNE3,CXCL12,CYP4F2,CYP4F3,DPT,EDNRA,EIF2S1,ELF1,ENPEP,ENTPD1,FAM198B,FGL2,FILIP1L,FLT1,GRB10,HNRNPL,IQGAP1,JAG1,KCNMA1,KIF13A,MAP4K5,MAST4,MBNL1,MCAM,MECOM,MUC5AC,NEFH,PDE4DIP,PROX1,PTGER2,RASAL2,RIOK3,SENP7,SLC13A2,SLC22A18,SLC39A4,SRPX2,STAG2,TES,TG,THBS1,TM4SF1,TNC,TPCN1,TUBAL3,UBE3B,USH1C,UTRN,VDR,VLDLR,ZFR,ZFX,ZNF277,ZNF480	62
Cardiovascular System Development and Function,Embryonic Development,Organ Development,Organismal Development,Tissue Development	cardiogenesis	0.00000123			CALCRL,CREB1,CXCL12,CXCR4,EDNRA,EMP1,FHL1,FILIP1L,JAG1,LTBP1,MECOM,PROX1,RAPGEF3,SMAD5,STK4,TGFB2,THBS1,UTRN,WNT5A	19
Cellular Movement	cell movement of tumor cell lines	0.00000126		-1.777	ADAM10,ANGPT2,CALU,CEACAM1,CHN2,CPNE3,CXCL12,CXCR4,DNAJB4,FLT1,IQGAP1,LEPR,LYVE1,MAGI1,MCAM,NCOA2,PDCD4,RAPGEF3,S100A4,SENP7,SMAD5,SRPX2,TCF7L2,TGFB2,THBS1,TNC,WNT5A	27
Cellular Movement	cell movement of connective tissue cells	0.00000144		-0.861	ANGPT2,CALCRL,CXCL12,CXCR4,DPT,ITGB1BP1,KCNMA1,PGGT1B,RAPGEF3,THBS1,UTRN,WNT5A	12
Cellular Movement	invasion of hybrid cells	0.00000146			CEACAM1,CXCL12,CXCR4	3

Cancer,Organismal Injury and Abnormalities	tumorigenesis of epithelial neoplasm	0.00000148		-0.063	ABCC9,ABHD6,ADAM10,ADAMTS9,ALDH6A1,ALLC,ANGPT2,ATRX,C4A/C4B,CALCR,CALCRL,CEACAM1,CES2,CHN2,CPE,CPNE3,CXCL12,CYP4F2,CYP4F3,DLK1,DPT,EDIL3,EIF2S1,ELF1,ENPEP,FAM198B,FAM216A,FERMT1,FHL1,FILIP1L,FLT1,GPX2,GRB10,HNF1B,HNRNPL,ID4,IQGAP1,JAG1,KCNMA1,KIF13A,LEPR,LTBP1,MAGI1,MAP4K5,MAST4,MBNL1,MBNL2,MCAM,MECOM,MIA2,MLXIPL,MUC5AC,NCOA2,NEFH,NFASC,NR1D2,OVGP1,PCM1,PDE4DIP,PGGT1B,PROX1,PTGER2,RAPGEF3,RASAL2,RIOK3,SEMA4G,SEMA6A,SENP7,SLC13A2,SLC22A18,SLC26A4,SLC38A2,SLC39A4,SPTLC3,SRPX2,SSBP2,STAG2,SULT1C2,SYT1,TAF13,TCF7L2,TES,TFRC,TG,THBS1,THSD7A,TM4SF1,TNC,TPCN1,TSPAN5,TUBAL3,UBE3B,USH1C,UTRN,VDR,VLDLR,VPS13D,WNT5A,ZFR,ZFX,ZFY,ZNF277,ZNF480,ZNF711	104
Cancer,Organismal Injury and Abnormalities	breast or colorectal cancer	0.00000158			ABCC9,ABO,ADAM10,ADAMTS9,ALDH6A1,ALLC,ANGPT2,ATRX,CALCR,CEACAM1,CES2,CPE,CXCL12,CXCR4,CYP4F2,DPT,EDIL3,EFS,ELF1,EMP1,ENPEP,FAM198B,FAM216A,FERMT1,FHL1,FLT1,GGH,GPX2,HNRNPL,JAG1,KCNMA1,KIF13A,LEPR,LTBP1,LYVE1,MAGI1,MAP4K5,MAST4,MBNL1,MCAM,MECOM,MIA2,MLXIPL,MUC5AC,NCOA2,NEFH,NFASC,NR1D2,OVGP1,PCM1,PDCD4,PDCD6,PDE4DIP,PGGT1B,PROX1,RAPGEF3,RASAL2,RIOK3,S100A4,SEMA4G,SEMA6A,SENP7,SLC13A2,SLC22A18,SLC38A2,SLC39A4,SSBP2,STAG2,STK4,SULT1C2,SYT1,TCF7L2,TES,TFRC,TG,TGFB2,THBS1,TM4SF1,TNC,TUBAL3,UTRN,VDR,VLDLR,VPS13D,WNT5A,ZFR,ZFX,ZFY,ZNF480,ZNF711	90
Cell Death and Survival	cell survival	0.00000158		-1.246	ANGPT2,ATRX,CALCR,CDC40,CEACAM1,CREB1,CXCL12,CXCR4,DLK1,EIF2S1,ELF1,EMP1,FLT1,GRB10,HNF1B,HSF2,ID4,IQGAP1,ITGB1BP1,JAG1,LEPR,MCAM,MECOM,NEFH,NUPR1,PDCD4,PRKACB,RAPGEF3,S100A4,SEMA6A,TANK,TGFB2,THBS1,VDR,WNT5A	35
Organismal Injury and Abnormalities,Reproductive System Disease	benign pelvic disease	0.00000168			ANGPT2,CALCRL,CREB1,CXCR4,DPT,FLT1,IQGAP1,LEPR,LTBP1,OVGP1,PRKACB,PTGER2,RAB23,RAPGEF3,SMAD5,TAF13,TGFB2,TNC,UTRN,VDR	20
Cellular Development,Connective Tissue Development and Function,Tissue Development	differentiation of bone cells	0.00000174		-1.689	CALCR,CEACAM1,CREB1,DLK1,EDNRA,ID4,JAG1,PLCL2,PTGER2,RAPGEF3,SMAD5,TFRC,TGFB2,TNC,TPM4,WNT5A	16
Cancer,Organismal Injury and Abnormalities	advanced malignant solid tumor	0.00000208		-0.369	ANGPT2,C4A/C4B,CXCL12,CXCR4,EFS,EMP1,ENPEP,FLT1,JAG1,KCNMA1,MAGI1,MAP4K5,MCAM,MECOM,S100A4,SLC39A4,TGFB2,THBS1,TM4SF1,TNC,VDR,WNT5A	22
Cardiovascular Disease,Cardiovascular System Development and Function	abnormal morphology of cardiovascular system	0.0000022			ADAM10,ADAMTS9,CALCRL,CLIC4,CXCL12,CXCR4,EDNRA,FLT1,JAG1,LTBP1,MBNL1,MBNL2,MECOM,SLCO2A1,SMAD5,STK4,TFRC,TGFB2,THBS1	19
Cancer,Hematological Disease,Organismal Injury and Abnormalities	lymphoid cancer	0.00000225		1.591	ABCC9,ADAMTS9,AMY1C (includes others),ANGPT2,ATRX,BAG2,C4A/C4B,CALCRL,CXCL12,CXCR4,DPT,FERMT1,FILIP1L,FLT1,HIST1H2BK,HNF1B,ID4,IQGAP1,LTBP1,MAGI1,MAP4K5,MCAM,MECOM,MIA2,MLXIPL,MSMO1,MUC5AC,NCOA2,NFASC,PAMR1,PDCD4,PDE4DIP,PLCL2,PRKACB,RASAL2,RSPH14,S100A4,SLC13A2,SMAD5,SSBP2,STAG2,TAF13,TFRC,TGFB2,THBS1,TLK1,TNC,VPS13D,ZNF277,ZNF711	50
Cancer,Organismal Injury and Abnormalities,Skeletal and Muscular Disorders	leiomyomatosis	0.0000024			ANGPT2,CALCRL,DPT,IQGAP1,LTBP1,PTGER2,RAB23,RAPGEF3,SMAD5,TAF13,TNC,UTRN,VDR	13
Cellular Development,Cellular Growth and Proliferation	cell proliferation of tumor cell lines	0.00000242		0.342	ADAM10,ANGPT2,CEACAM1,CES2,CHN2,CREB1,CXCL12,CXCR4,DLK1,DNAJB4,EMP1,ENTPD1,FLT1,HNF1B,IQGAP1,JAG1,KIF13A,MAGI1,MECOM,NANOG,NCOA2,PDCD4,PRKACB,PROX1,PTGER2,S100A4,SMAD5,STAG2,STK4,TCF7L2,TES,TFRC,TGFB2,THBS1,TNC,UTRN,VDR,WNT5A	38

Cancer,Organismal Injury and Abnormalities	tumorigenesis of malignant tumor	0.00000242		-0.493	ABCC9,ABHD6,ADAM10,ADAMTS9,ALDH6A1,ALLC,ANGPT2,ATRX,C4A/C4B,CALCR,CALCRL,CEACAM1,CES2,CHN2,CPE,CPNE3,CXCL12,CXCR4,CYP4F2,CYP4F3,DLK1,EDIL3,EIF2S1,ELF1,ENPEP,FAM198B,FAM216A,FERMT1,FHL1,FILIP1L,FLT1,GPX2,GRB10,HNF1B,HNRNPL,IQGAP1,JAG1,KCNMA1,KIF13A,LEPR,LTBP1,MAGI1,MAP4K5,MAST4,MBNL1,MBNL2,MCAM,MECOM,MIA2,MLXIPL,MUC5AC,NCOA2,NEFH,NFASC,NR1D2,OVGP1,PCM1,PDE4DIP,PGGT1B,PROX1,PTGER2,RAPGEF3,RASAL2,RIOK3,SEMA4G,SEMA6A,SENP7,SLC13A2,SLC22A18,SLC26A4,SLC38A2,SLC39A4,SPTLC3,SRPX2,SSBP2,STAG2,SULT1C2,SYT1,TCF7L2,TES,TFRC,TG,TGFB2,THBS1,THSD7A,TM4SF1,TNC,TPCN1,TSPAN5,TUBAL3,UBE3B,USH1C,UTRN,VDR,VLDLR,VPS13D,WNT5A,ZFR,ZFX,ZFY,ZNF277,ZNF480,ZNF711	103
Cancer,Organismal Injury and Abnormalities	melanoma	0.00000272			ABCC9,ABHD6,ABO,ADAM10,ADAMTS9,ALDH6A1,ALLC,ATRX,C4A/C4B,CALCR,CALCRL,CALU,CEACAM1,CES2,CHN2,CPE,CPNE3,CYP4F2,CYP4F3,DLK1,EDIL3,EDNRA,EFS,ELF1,ENPEP,ENTPD1,FAM198B,FAM216A,FERMT1,FGL2,FHL1,FHL5,FILIP1L,FLT1,HNRNPL,HSF2,IQGAP1,JAG1,KCNMA1,KIF13A,LEPR,LTBP1,MAGI1,MAP4K5,MAST4,MBNL1,MCAM,MECOM,MIA2,MLXIPL,MUC5AC,NANOG,NCOA2,NEFH,NFASC,OVGP1,PAMR1,PDCD6,PDE4DIP,PLCL2,PRKACB,PROX1,PROZ,PTGER2,RAPGEF3,RASAL2,RIOK3,S100A4,SEMA4G,SENP7,SLC13A2,SLC22A18,SLC26A4,SLC39A4,SLCO2A1,SPAG5,SPTLC3,STK4,SULT1C2,SYT1,TAF13,TCF7L2,TES,TFRC,TG,TGFB2,THBS1,THSD7A,TLK1,TM4SF1,TNC,TPCN1,TSPAN5,TUBAL3,USH1C,UTRN,VDR,VLDLR,VPS13D,WNT5A,ZFR,ZFX,ZNF277,ZNF480,ZNF711	105
Cancer,Organismal Injury and Abnormalities	cancer of head	0.00000291			ADAM10,ANGPT2,CXCL12,DPT,EDIL3,ENPEP,FLT1,MAP4K5,NANOG,PRKACB,SSBP2,THBS1,TLK1,TM4SF1,TNC,ZFX	16
Cancer,Organismal Injury and Abnormalities,Skeletal and Muscular Disorders	smooth muscle tumor	0.00000295			ANGPT2,CALCRL,DPT,FLT1,IQGAP1,LTBP1,PTGER2,RAB23,RAPGEF3,SMAD5,TAF13,TNC,UTRN,VDR	14
Cancer,Gastrointestinal Disease,Organismal Injury and Abnormalities	colorectal neoplasia	0.00000303			ADAM10,ADAMTS9,ALDH6A1,ALLC,ANGPT2,ATRX,CALCR,CEACAM1,CES2,CXCL12,CYP4F2,DPT,EDIL3,EFS,ELF1,EMP1,ENPEP,FAM198B,FAM216A,FERMT1,FHL1,FLT1,GGH,GPX2,HNRNPL,JAG1,KCNMA1,KIF13A,LEPR,LTBP1,MAGI1,MAP4K5,MAST4,MBNL1,MCAM,MECOM,MIA2,MLXIPL,MUC5AC,NCOA2,NEFH,NFASC,NR1D2,OVGP1,PCM1,PDCD4,PDE4DIP,PGGT1B,RAPGEF3,RASAL2,RIOK3,S100A4,SEMA4G,SEMA6A,SENP7,SLC13A2,SLC38A2,SLC39A4,SSBP2,STAG2,STK4,SULT1C2,SYT1,TCF7L2,TES,TFRC,TG,TGFB2,THBS1,THSD7A,TM4SF1,TNC,TUBAL3,UTRN,VDR,VPS13D,WNT5A,ZFR,ZFX,ZFY,ZNF480,ZNF711	82
Cancer,Gastrointestinal Disease,Organismal Injury and Abnormalities	colorectal cancer	0.00000318			ADAM10,ADAMTS9,ALDH6A1,ALLC,ANGPT2,ATRX,CALCR,CEACAM1,CES2,CXCL12,CYP4F2,DPT,EDIL3,EFS,ELF1,EMP1,ENPEP,FAM198B,FAM216A,FERMT1,FHL1,FLT1,GGH,GPX2,HNRNPL,JAG1,KCNMA1,KIF13A,LEPR,LTBP1,MAGI1,MAP4K5,MAST4,MBNL1,MCAM,MECOM,MIA2,MLXIPL,MUC5AC,NCOA2,NEFH,NFASC,NR1D2,OVGP1,PCM1,PDCD4,PDE4DIP,PGGT1B,RAPGEF3,RASAL2,RIOK3,S100A4,SEMA4G,SEMA6A,SENP7,SLC13A2,SLC38A2,SLC39A4,SSBP2,STAG2,STK4,SULT1C2,SYT1,TCF7L2,TES,TFRC,TG,TGFB2,THBS1,TM4SF1,TNC,TUBAL3,UTRN,VDR,VPS13D,WNT5A,ZFR,ZFX,ZFY,ZNF480,ZNF711	81
Cellular Function and Maintenance	cellular homeostasis	0.0000032		-1.693	ABCC9,ADAM10,ANGPT2,C4A/C4B,CALCR,CEACAM1,CLIC4,CPE,CREB1,CXCL12,CXCR4,EDNRA,EIF2S1,ELF1,ENTPD1,FLT1,GABPA,HNRNPL,JAG1,KCNMA1,LEPR,LTBP1,MCAM,MECOM,MLXIPL,NUPR1,PLCL2,PTGER2,RAPGEF3,SLC26A4,SLC39A4,TCF7L2,TFRC,TG,TPCN1,VDR,WNT5A	37
Cancer,Hematological Disease,Organismal Injury and Abnormalities	hematologic cancer	0.00000368		1.757	ABCC9,ADAM10,ADAMTS9,AMY1C (includes others),ANGPT2,ATRX,BAG2,C4A/C4B,CALCRL,CXCL12,CXCR4,DPT,FERMT1,FILIP1L,FLT1,HIST1H2BK,HNF1B,ID4,IQGAP1,LTBP1,MAGI1,MAP4K5,MCAM,MECOM,MIA2,MLXIPL,MSMO1,MUC5AC,NCOA2,NFASC,PAMR1,PDCD4,PDE4DIP,PLCL2,PRKACB,RASAL2,RSPH14,S100A4,SLC13A2,SMAD5,SSBP2,STAG2,TAF13,TFRC,TGFB2,THBS1,TLK1,TNC,VPS13D,ZNF277,ZNF711	51
Embryonic Development,Nervous System Development and Function,Organ Development,Organismal Development,Tissue Development	formation of brain	0.00000405		-0.836	ADAM10,ATRX,CREB1,CXCL12,CXCR4,HNF1B,ID4,MECOM,NEFH,PROX1,RAPGEF3,S100A4,SMAD5,TG,THBS1,TNC,VLDLR,WNT5A	18

Cellular Movement	migration of tumor cell lines	0.00000417		-1.64	ADAM10,ANGPT2,CALU,CEACAM1,CHN2,CPNE3,CXCL12,CXCR4,IQGAP1,LEPR,LYVE1,MAGI1,MCAM,NCOA2,PDCD4,RAPGEF3,S100A4,SMAD5,SRPX2,TCF7L2,THBS1,TNC,WNT5A	23
Cancer,Organismal Injury and Abnormalities	adenocarcinoma	0.00000431		0.588	ABCC9,ABHD6,ABO,ADAM10,ADAMTS9,ALDH6A1,ALLC,ANGPT2,ATRX,C4A/C4B,CALCR,CALCRL,CALU,CDC40,CEACAM1,CES2,CHN2,CPE,CPNE3,CREB1,CXCL12,CXCR4,CYP4F2,CYP4F3,DLK1,DNAJB4,DPT,EDIL3,EDNRA,EIF2S1,ELF1,ENOSF1,ENPEP,ENTPD1,FAM198B,FAM216A,FERMT1,FGL2,FHL1,FHL5,FILIP1L,FLT1,GGH,GRB10,HNF1B,HNRNPL,IQGAP1,JAG1,KCNMA1,KIF13A,LEPR,LHPP,LTBP1,LYVE1,MAGI1,MAP4K5,MAST4,MBNL1,MBNL2,MCAM,ME2,MECOM,MIA2,MLXIPL,MUC5AC,NCOA2,NEFH,NFASC,NR1D2,OVGP1,PAMR1,PCM1,PDCD4,PDE4DIP,PGGT1B,PLCL2,PRKACB,PROX1,PTGER2,RAPGEF3,RASAL2,RETREG1,RIOK3,S100A4,SEMA4G,SEMA6A,SENP7,SLC13A2,SLC22A18,SLC26A4,SLC38A2,SLC39A4,SLCO2A1,SMAD5,SPAG5,SPTLC3,SRPX2,SSBP2,STAG2,SULT1C2,SYT1,TCF7L2,TES,TFRC,TG,TGFB2,THBS1,THSD7A,TLK1,TM4SF1,TMX1,TNC,TPCN1,TSPAN5,TUBAL3,UBE3B,USH1C,UTRN,VDR,VLDLR,VPS13D,WNT5A,ZFR,ZFX,ZFY,ZNF277,ZNF480,ZNF711	128
Cancer,Organismal Injury and Abnormalities	solid tumor of head and neck	0.00000442			ABCC9,ABO,ADAM10,ALLC,AMY1C (includes others),ANGPT2,ATRX,CALCR,CES2,CREB1,CXCL12,CXCR4,DPT,EDIL3,ENPEP,FAM198B,FHL5,FLT1,MAGI1,MECOM,MIA2,MUC5AC,NEFH,NR1D2,OVGP1,PCM1,PDE4DIP,PRKACB,PROX1,S100A4,SEMA6A,SSBP2,STAG2,TG,THBS1,THSD7A,TLK1,TM4SF1,TNC,WNT5A	40
Cancer,Organismal Injury and Abnormalities	papillary adenocarcinoma	0.00000464			C4A/C4B,CES2,CREB1,CXCR4,ENPEP,FLT1,MCAM,PCM1,SLC39A4,TG,THBS1,WNT5A	12
Cell Morphology	morphology of cells	0.00000485			ADAM10,ANGPT2,ATRX,CLIC4,CREB1,CXCL12,CXCR4,DLK1,DPT,EDNRA,EMP1,FHL1,FHL5,FLT1,GABPA,HSF2,IQGAP1,KCNMA1,LEPR,MBNL1,MBNL2,MECOM,MUC5AC,NCOA2,NEFH,NFASC,PGGT1B,PTGER2,RAPGEF3,RETREG1,S100A4,SLC26A4,SMAD5,STK4,TCF7L2,TFRC,TG,TGFB2,THBS1,TNC,USH1C,UTRN,VDR,VLDLR,WNT5A	45
Gastrointestinal Disease,Hepatic System Disease,Organismal Injury and Abnormalities	liver lesion	0.000005		0.705	ABCC9,ABHD6,ANGPT2,ATRX,BAG2,C4A/C4B,CALCRL,CALU,CDC40,CEACAM1,CES2,CHN2,CPNE3,CREB1,CXCL12,CXCR4,DLK1,DPT,EDIL3,EDNRA,EFS,ENTPD1,FERMT1,FGL2,FHL1,FLT1,GPX2,GRB10,ID4,IQGAP1,JAG1,KCNMA1,LEPR,LYVE1,MAST4,MDFIC,ME2,MECOM,MIA2,MLXIPL,MUC5AC,NCOA2,NUPR1,OVGP1,PAMR1,PDE4DIP,PLCL2,PROX1,PROZ,RAB23,RAPGEF3,RASAL2,RIOK3,S100A4,SEMA6A,SLC26A4,SLC38A2,SLC39A4,SRPX2,STAG2,STK4,TANK,TCF7L2,TFRC,TG,TGFB2,THBS1,TLK1,TPCN1,UBE3B,USH1C,UTRN,VDR,VLDLR,VPS13D,ZFR,ZNF277	77
Cancer,Endocrine System Disorders,Organismal Injury and Abnormalities	thyroid cancer	0.00000512			AMY1C (includes others),ANGPT2,CES2,CREB1,CXCR4,FLT1,MIA2,NEFH,PCM1,PDE4DIP,TAF13,TG,THBS1,UTRN,WNT5A	15
Cancer,Organismal Injury and Abnormalities	abdominal carcinoma	0.00000555			ABCC9,ABHD6,ABO,ADAM10,ADAMTS9,ALDH6A1,ALLC,ANGPT2,ATRX,C4A/C4B,CALCR,CALCRL,CES2,CHN2,CPE,CPNE3,CXCL12,CXCR4,CYP4F2,CYP4F3,DPT,EDIL3,EDNRA,EIF2S1,ELF1,ENOSF1,ENPEP,ENTPD1,FAM198B,FAM216A,FGL2,FHL1,FILIP1L,FLT1,GRB10,HNF1B,HNRNPL,IQGAP1,JAG1,KCNMA1,KIF13A,LEPR,LTBP1,MAGI1,MAP4K5,MAST4,MBNL1,MBNL2,MCAM,MECOM,MIA2,MLXIPL,MUC5AC,NCOA2,NEFH,NFASC,NR1D2,OVGP1,PCM1,PDE4DIP,PGGT1B,PRKACB,PROX1,PTGER2,RAPGEF3,RASAL2,RIOK3,SEMA4G,SEMA6A,SENP7,SLC13A2,SLC22A18,SLC26A4,SLC38A2,SLC39A4,SLCO2A1,SPTLC3,SRPX2,SSBP2,STAG2,SULT1C2,SYT1,TCF7L2,TES,TFRC,TG,THBS1,THSD7A,TM4SF1,TNC,TPCN1,TSPAN5,TUBAL3,UBE3B,USH1C,UTRN,VDR,VLDLR,VPS13D,WNT5A,ZFR,ZFX,ZFY,ZNF277,ZNF480,ZNF711	106

Cancer,Organismal Injury and Abnormalities	abdominal adenocarcinoma	0.0000057			ABCC9,ABHD6,ABO,ADAM10,ADAMTS9,ALDH6A1,ALLC,ANGPT2,ATRX,C4A/C4B,CALCR,CALCRL,CES2,CHN2,CPE,CPNE3,CXCL12,CXCR4,CYP4F2,CYP4F3,DPT,EDIL3,EDNRA,EIF2S1,ELF1,ENOSF1,ENPEP,ENTPD1,FAM198B,FAM216A,FGL2,FHL1,FILIP1L,FLT1,GRB10,HNF1B,HNRNPL,IQGAP1,JAG1,KCNMA1,KIF13A,LEPR,LTBP1,MAGI1,MAP4K5,MAST4,MBNL1,MBNL2,MCAM,MECOM,MIA2,MLXIPL,MUC5AC,NCOA2,NEFH,NFASC,NR1D2,OVGP1,PCM1,PDE4DIP,PGGT1B,PRKACB,PROX1,PTGER2,RAPGEF3,RASAL2,RIOK3,SEMA4G,SEMA6A,SENP7,SLC13A2,SLC22A18,SLC26A4,SLC38A2,SLC39A4,SPTLC3,SRPX2,SSBP2,STAG2,SULT1C2,SYT1,TCF7L2,TES,TFRC,TG,THBS1,THSD7A,TM4SF1,TNC,TPCN1,TSPAN5,TUBAL3,UBE3B,USH1C,UTRN,VDR,VLDLR,VPS13D,WNT5A,ZFR,ZFX,ZFY,ZNF277,ZNF480,ZNF711	105
Cancer,Organismal Injury and Abnormalities	tumorigenesis of carcinoma	0.00000592		-0.447	ABCC9,ABHD6,ADAM10,ADAMTS9,ALDH6A1,ALLC,ANGPT2,ATRX,C4A/C4B,CALCR,CALCRL,CEACAM1,CES2,CHN2,CPE,CPNE3,CXCL12,CYP4F2,CYP4F3,DLK1,EDIL3,EIF2S1,ELF1,ENPEP,FAM198B,FAM216A,FERMT1,FHL1,FILIP1L,FLT1,GPX2,GRB10,HNF1B,HNRNPL,IQGAP1,JAG1,KCNMA1,KIF13A,LEPR,LTBP1,MAGI1,MAP4K5,MAST4,MBNL1,MBNL2,MCAM,MECOM,MIA2,MLXIPL,MUC5AC,NCOA2,NEFH,NFASC,NR1D2,OVGP1,PCM1,PDE4DIP,PGGT1B,PROX1,PTGER2,RAPGEF3,RASAL2,RIOK3,SEMA4G,SEMA6A,SENP7,SLC13A2,SLC22A18,SLC26A4,SLC38A2,SLC39A4,SPTLC3,SRPX2,SSBP2,STAG2,SULT1C2,SYT1,TCF7L2,TES,TFRC,TG,THBS1,THSD7A,TM4SF1,TNC,TPCN1,TSPAN5,TUBAL3,UBE3B,USH1C,UTRN,VDR,VLDLR,VPS13D,WNT5A,ZFR,ZFX,ZFY,ZNF277,ZNF480,ZNF711	101
Cardiovascular System Development and Function,Organismal Development,Tissue Morphology	morphology of cardiovascular tissue	0.00000614			CLIC4,CXCR4,FLT1,SMAD5,STK4,TGFB2,THBS1	7
Cancer,Organismal Injury and Abnormalities	tumorigenesis of benign tumor	0.0000062			ANGPT2,ATRX,C4A/C4B,CES2,DPT,ENPEP,FLT1,ID4,LEPR,MCAM,MECOM,PTGER2,S100A4,SLC39A4,SSBP2,STAG2,TAF13,TG,THBS1,VDR	20
Cancer,Organismal Injury and Abnormalities,Reproductive System Disease	adenocarcinoma in endometrium	0.00000696			ABCC9,ABHD6,ANGPT2,ATRX,CES2,CHN2,CPNE3,CXCL12,CYP4F2,CYP4F3,EIF2S1,ELF1,FAM198B,FILIP1L,GRB10,HNRNPL,IQGAP1,JAG1,KCNMA1,KIF13A,MAP4K5,MAST4,MBNL1,MECOM,MUC5AC,NEFH,PDE4DIP,PROX1,RASAL2,RIOK3,SENP7,SLC13A2,SRPX2,STAG2,TES,TG,THBS1,TNC,TPCN1,UBE3B,USH1C,UTRN,VDR,VLDLR,ZFR,ZFX,ZNF277,ZNF480	48
Cancer,Neurological Disease,Organismal Injury and Abnormalities	central nervous system cancer	0.00000698			ANGPT2,ATRX,DPT,FLT1,KCNMA1,LHPP,MAP4K5,MECOM,NANOG,RSPH14,S100A4,STAG2,TAF13,TCF7L2,TGFB2,THBS1,TNC,USH1C,ZFX	19
Cell Death and Survival,Organismal Injury and Abnormalities	necrosis of epithelial tissue	0.0000071		-0.394	ANGPT2,ATRX,CALCR,CEACAM1,CLIC4,CXCL12,CXCR4,EDIL3,EMP1,FGL2,FLT1,MCAM,MECOM,NCOA2,SEMA6A,STK4,TFRC,THBS1,TMX1	19
Cancer,Gastrointestinal Disease,Organismal Injury and Abnormalities	upper gastrointestinal tract tumor	0.00000737			ABCC9,ADAM10,ADAMTS9,ANGPT2,CES2,CPE,CXCL12,CXCR4,CYP4F2,DPT,EDIL3,ENOSF1,ENPEP,FGL2,FILIP1L,FLT1,HNRNPL,JAG1,KCNMA1,LEPR,MAST4,MBNL2,ME2,MECOM,MUC5AC,NCOA2,NEFH,NFASC,OVGP1,PDE4DIP,PRKACB,PROX1,RASAL2,SEMA4G,SLC13A2,SLCO2A1,SPTLC3,SSBP2,TES,TG,THBS1,THSD7A,TLK1,TM4SF1,TNC,TPCN1,UBE3B,UTRN,VLDLR,WNT5A,ZFR,ZNF480	52
Cancer,Organismal Injury and Abnormalities,Reproductive System Disease	female genital tract serous carcinoma	0.00000772			ATRX,C4A/C4B,CXCL12,DPT,EDNRA,ENPEP,ENTPD1,FGL2,FILIP1L,KCNMA1,MCAM,MECOM,PTGER2,SLC39A4,STAG2	15
Cancer,Gastrointestinal Disease,Hepatic System Disease,Organismal Injury and Abnormalities	hepatocellular carcinoma	0.00000862			BAG2,CEACAM1,CES2,CREB1,CXCL12,DPT,FERMT1,FLT1,GPX2,IQGAP1,JAG1,LYVE1,NUPR1,PAMR1,PROX1,PROZ,RASAL2,S100A4,SLC26A4,TCF7L2,TG,VDR	22

Cancer,Gastrointestinal Disease,Organismal Injury and Abnormalities	gastrointestinal neoplasia	0.00000869		0.374	ABCC9,ADAM10,ADAMTS9,ALDH6A1,ALLC,ANGPT2,ATRX,CALCR,CALCRL,CALU,CDC40,CEACAM1,CES2,CPE,CREB1,CXCL12,CXCR4,CYP4F2,DLK1,DNAJB4,DPT,EDIL3,EFS,ELF1,EMP1,ENOSF1,ENPEP,FAM198B,FAM216A,FERMT1,FGL2,FHL1,FHL5,FILIP1L,FLT1,GGH,GPX2,GRB10,HNRNPL,IQGAP1,ITGB1BP1,JAG1,KCNMA1,KIF13A,LEPR,LHPP,LTBP1,MAGI1,MAP4K5,MAST4,MBNL1,MBNL2,MCAM,ME2,MECOM,MIA2,MLXIPL,MUC5AC,NCOA2,NEFH,NFASC,NR1D2,OVGP1,PAMR1,PCM1,PDCD4,PDE4DIP,PGGT1B,PLCL2,PRKACB,PROX1,RAPGEF3,RASAL2,RIOK3,S100A4,SEMA4G,SEMA6A,SENP7,SLC13A2,SLC26A4,SLC38A2,SLC39A4,SLCO2A1,SPAG5,SPTLC3,SRPX2,SSBP2,STAG2,STK4,SULT1C2,SYT1,TAF13,TCF7L2,TES,TFRC,TG,TGFB2,THBS1,THSD7A,TLK1,TM4SF1,TMX1,TNC,TPCN1,TSPAN5,TUBAL3,UBE3B,UTRN,VDR,VLDLR,VPS13D,WNT5A,ZFR,ZFX,ZFY,ZNF277,ZNF480,ZNF711	118
Cancer,Cellular Development,Cellular Growth and Proliferation,Organismal Injury and Abnormalities,Tumor Morphology	proliferation of cancer cells	0.00000876		-0.616	ADAM10,CEACAM1,CXCL12,CXCR4,DLK1,FHL1,JAG1,MCAM,PROX1,TCF7L2,TGFB2,THBS1,VDR,WNT5A	14
Cancer,Organismal Injury and Abnormalities	head and neck carcinoma	0.00000926			ABCC9,ABO,ADAM10,ALLC,AMY1C (includes others),ANGPT2,ATRX,CALCR,CES2,CREB1,CXCL12,CXCR4,DPT,EDIL3,ENPEP,FAM198B,FHL5,FLT1,MAGI1,MECOM,MIA2,MUC5AC,NEFH,NR1D2,OVGP1,PCM1,PDE4DIP,PRKACB,PROX1,S100A4,SEMA6A,STAG2,TG,THBS1,THSD7A,TLK1,TM4SF1,TNC,WNT5A	39
Cancer,Endocrine System Disorders,Organismal Injury and Abnormalities	endocrine gland tumor	0.00000974			ABCC9,ADAM10,AMY1C (includes others),ANGPT2,ATRX,CES2,CREB1,CXCR4,CYP4F2,FERMT1,FLT1,ID4,ITGB1BP1,KCNMA1,MIA2,NEFH,PCM1,PDCD6,PDE4DIP,RASAL2,TAF13,TG,THBS1,UTRN,VDR,WNT5A,ZFR,ZNF277	28
Cancer,Organismal Injury and Abnormalities	head and neck adenocarcinoma	0.00000979			ANGPT2,FLT1,PRKACB,TG,THBS1,TLK1,TNC	7
Nervous System Development and Function	development of central nervous system	0.0000103		0.113	ADAM10,ATRX,CREB1,CXCL12,CXCR4,HNF1B,ID4,MECOM,NEFH,PROX1,RAPGEF3,S100A4,SMAD5,STK4,TCF7L2,TG,THBS1,TNC,VLDLR,WNT5A	20
Cell Death and Survival	necrosis	0.0000103		-0.408	ABCC9,ADAM10,ANGPT2,ATRX,CALCR,CALCRL,CEACAM1,CLIC4,CREB1,CXCL12,CXCR4,DLK1,EDIL3,EDNRA,EIF2S1,ELF1,EMP1,ENTPD1,FGL2,FLT1,GPX2,GRB10,HNF1B,JAG1,KCNMA1,LEPR,LTBP1,MAGI1,MCAM,MECOM,NANOG,NCOA2,NEFH,NUPR1,PDCD4,PTGER2,RAPGEF3,RETREG1,S100A4,SEMA6A,SPAG5,STK4,TANK,TCF7L2,TFRC,TGFB2,THBS1,TMX1,TNC,VDR,WNT5A	51
Organ Morphology,Reproductive System Development and Function,Tissue Morphology	quantity of germ cells	0.0000114		-1.783	CXCL12,CXCR4,ENTPD1,FHL5,SMAD5,TGFB2,VDR,WNT5A,ZFX	9
Molecular Transport,Nucleic Acid Metabolism,Small Molecule Biochemistry	quantity of cyclic nucleotides	0.0000114		0.419	CALCRL,CXCL12,CXCR4,CYP4F2,ENTPD1,HNF1B,LEPR,PTGER2,THBS1,WNT5A	10
Cancer,Gastrointestinal Disease,Organismal Injury and Abnormalities	upper gastrointestinal tract cancer	0.0000123			ABCC9,ADAM10,ADAMTS9,ANGPT2,CES2,CPE,CXCL12,CXCR4,CYP4F2,DPT,EDIL3,ENPEP,FILIP1L,FLT1,HNRNPL,KCNMA1,MAST4,MBNL2,MUC5AC,NCOA2,NEFH,NFASC,OVGP1,PDE4DIP,PRKACB,SEMA4G,SLC13A2,SPTLC3,SSBP2,TG,THBS1,THSD7A,TLK1,TM4SF1,TNC,TPCN1,UBE3B,UTRN,VLDLR,WNT5A,ZFR,ZNF480	42
Cardiovascular System Development and Function,Tissue Morphology	permeability of vascular system	0.0000124		-0.58	ADAM10,ANGPT2,C4A/C4B,CREB1,CXCL12,FLT1,MCAM,RAPGEF3,THBS1	9
Cellular Movement,Reproductive System Development and Function	migration of primordial germ cells	0.0000126			CXCL12,CXCR4,WNT5A	3

Cancer,Endocrine System Disorders,Organismal Injury and Abnormalities	thyroid gland nonmedullary carcinoma	0.0000129			ANGPT2,CES2,CREB1,CXCR4,FLT1,PCM1,TG,THBS1,WNT5A	9
Cancer,Organismal Injury and Abnormalities,Reproductive System Disease	tumorigenesis of genital tumor	0.000013			ABCC9,ABHD6,ANGPT2,ATRX,C4A/C4B,CES2,CHN2,CPE,CPNE3,CXCL12,CYP4F2,CYP4F3,EIF2S1,ELF1,ENPEP,FAM198B,FILIP1L,FLT1,GRB10,HNRNPL,IQGAP1,JAG1,KCNMA1,KIF13A,MAP4K5,MAST4,MBNL1,MCAM,MECOM,MUC5AC,NEFH,PDE4DIP,PROX1,PTGER2,RASAL2,RIOK3,SENP7,SLC13A2,SLC22A18,SLC39A4,SRPX2,STAG2,TES,TG,THBS1,TM4SF1,TNC,TPCN1,TUBAL3,UBE3B,USH1C,UTRN,VDR,VLDLR,ZFR,ZFX,ZNF277,ZNF480	58
Cancer,Organismal Injury and Abnormalities,Reproductive System Disease	uterine carcinoma	0.0000131			ABCC9,ABHD6,ANGPT2,ATRX,CES2,CHN2,CPNE3,CXCL12,CYP4F2,CYP4F3,EIF2S1,ELF1,FAM198B,FILIP1L,GRB10,HNRNPL,IQGAP1,JAG1,KCNMA1,KIF13A,MAP4K5,MAST4,MBNL1,MECOM,MUC5AC,NEFH,PDE4DIP,PROX1,RASAL2,RIOK3,SENP7,SLC13A2,SLCO2A1,SRPX2,STAG2,TES,TG,THBS1,TNC,TPCN1,UBE3B,USH1C,UTRN,VDR,VLDLR,WNT5A,ZFR,ZFX,ZNF277,ZNF480	50
Cancer,Organismal Injury and Abnormalities	cancer of cells	0.0000132		-1.912	ABCC9,ABO,ADAM10,ADAMTS9,ALLC,ANGPT2,ATRX,CALCR,CES2,CHN2,CREB1,CXCL12,CXCR4,CYP4F3,DPT,EDIL3,ENOSF1,ENPEP,FAM198B,FERMT1,FGL2,FHL5,FILIP1L,GPX2,GRB10,JAG1,KCNMA1,LEPR,MAGI1,MAST4,MBNL1,MBNL2,MCAM,ME2,MECOM,MIA2,MUC5AC,NEFH,NR1D2,OVGP1,PCM1,PDCD4,PDE4DIP,PROX1,PTGER2,RASAL2,RETREG1,S100A4,SEMA4G,SEMA6A,SLCO2A1,SSBP2,STAG2,TCF7L2,TG,THBS1,THSD7A,TM4SF1,TNC,USH1C,WNT5A,ZNF711	62
Cardiovascular Disease,Cardiovascular System Development and Function,Organismal Injury and Abnormalities	abnormal morphology of vasculature	0.0000135			CALCRL,CLIC4,CREB1,CXCL12,CXCR4,DLK1,EDNRA,FLT1,LTBP1,SMAD5,STK4,TGFB2,THBS1	13
Post-Translational Modification	phosphorylation of protein	0.0000138		-1.579	ADAM10,CREB1,CXCR4,EDIL3,EDNRA,EIF2S1,ELF1,FLT1,GRB10,IQGAP1,MAP4K5,PRKACB,RAPGEF3,SMAD5,SRPX2,STK4,TGFB2,THBS1,TLK1,WNT5A	20
Cancer,Organismal Injury and Abnormalities,Reproductive System Disease	uterine endometrioid carcinoma	0.000014			ABCC9,ABHD6,ANGPT2,ATRX,CHN2,CPNE3,CXCL12,CYP4F2,CYP4F3,EIF2S1,ELF1,FAM198B,FILIP1L,GRB10,HNRNPL,IQGAP1,JAG1,KCNMA1,KIF13A,MAP4K5,MAST4,MBNL1,MECOM,MUC5AC,NEFH,PDE4DIP,PROX1,RASAL2,RIOK3,SENP7,SLC13A2,SRPX2,STAG2,TES,TG,THBS1,TNC,TPCN1,UBE3B,USH1C,UTRN,VDR,VLDLR,ZFR,ZFX,ZNF277,ZNF480	47
Connective Tissue Disorders,Inflammatory Disease,Organismal Injury and Abnormalities,Skeletal and Muscular Disorders	Rheumatic Disease	0.0000143		-0.627	ADAM10,ADAMTS9,C4A/C4B,CALCRL,CEACAM1,CPE,CREB1,CXCL12,CXCR4,CYP4F3,EDNRA,FGL2,FLT1,GRB10,JAG1,LEPR,MAGI1,MUC5AC,NCOA2,PCM1,PTGER2,TAF13,TCF7L2,TFRC,TGFB2,THBS1,TNC,VDR,WNT5A	29
Inflammatory Disease	chronic inflammatory disorder	0.0000144			ADAM10,C4A/C4B,CALCRL,CXCL12,CXCR4,CYP4F3,ENTPD1,FERMT1,FGL2,FLT1,GRB10,HSF2,LEPR,MAGI1,MCAM,MSMO1,NCOA2,PCM1,PTGER2,S100A4,TAF13,TCF7L2,TFRC,TGFB2,THBS1,TNC,VDR,WNT5A	28
Cancer,Endocrine System Disorders,Organismal Injury and Abnormalities	differentiated thyroid cancer	0.0000146			ANGPT2,CES2,CREB1,CXCR4,FLT1,PCM1,TG,THBS1,WNT5A	9
Molecular Transport	transport of molecule	0.0000148		0.627	ABCC9,ANGPT2,CALCR,CALCRL,CLIC4,CPE,CPNE3,CREB1,CXCL12,EDNRA,ENTPD1,FHL1,GRB10,IQGAP1,ITGB1BP1,KCNMA1,KIF13A,PDCD6,RAPGEF3,SLC13A2,SLC22A18,SLC26A4,SLC38A2,SLC38A3,SLC39A4,SLCO2A1,SYT1,TFRC,TG,TGFB2,THBS1,TLK1,VDR,VLDLR,WNT5A	35
Cancer,Neurological Disease,Organismal Injury and Abnormalities	glioma	0.0000152		-1	ALLC,ANGPT2,ATRX,CXCL12,CXCR4,DLK1,FLT1,JAG1,KCNMA1,LHPP,MECOM,MUC5AC,NEFH,PDE4DIP,S100A4,SMAD5,SPTLC3,STAG2,SULT1C2,TAF13,TCF7L2,TGFB2,TNC,USH1C,WNT5A,ZNF480	26

Cardiovascular System Development and Function,Cell-To-Cell Signaling and Interaction	adhesion of vascular endothelial cells	0.000016		-0.3	ADAM10,ANGPT2,CXCL12,CXCR4,FLT1,MCAM,THBS1	7
Cardiovascular System Development and Function,Cellular Movement	migration of endothelial cells	0.000016		-1.634	ADAM10,ANGPT2,CXCL12,EDIL3,FLT1,ITGB1BP1,MCAM,PDCD6,PROX1,TG,TGFB2,THBS1,WNT5A	13
Cancer,Organismal Injury and Abnormalities	benign connective or soft tissue neoplasm	0.0000163			ANGPT2,CALCRL,DPT,FLT1,IQGAP1,LTBP1,PTGER2,RAB23,RAPGEF3,SMAD5,TAF13,TNC,UTRN,VDR	14
Cancer,Neurological Disease,Organismal Injury and Abnormalities	astrocytoma	0.0000163			ALLC,ANGPT2,ATRX,CXCL12,CXCR4,FLT1,JAG1,KCNMA1,LHPP,MECOM,MUC5AC,NEFH,PDE4DIP,S100A4,SMAD5,SPTLC3,STAG2,SULT1C2,TAF13,TCF7L2,TGFB2,TNC,USH1C,ZNF480	24
Cancer,Organismal Injury and Abnormalities,Reproductive System Disease	metastasis of mammary tumor	0.0000183		-1.172	CXCR4,FLT1,PTGER2,S100A4,TM4SF1,WNT5A	6
Cancer,Gastrointestinal Disease,Organismal Injury and Abnormalities	colon tumor	0.0000184			ADAM10,ADAMTS9,ALDH6A1,ALLC,ANGPT2,ATRX,CALCR,CEACAM1,CES2,CYP4F2,EDIL3,ELF1,ENPEP,FAM216A,FERMT1,FHL1,FLT1,GGH,GPX2,HNRNPL,JAG1,KCNMA1,KIF13A,LEPR,LTBP1,MAGI1,MAP4K5,MAST4,MBNL1,MCAM,MECOM,MIA2,MLXIPL,MUC5AC,NCOA2,NEFH,NFASC,NR1D2,OVGP1,PCM1,PDCD4,PDE4DIP,PGGT1B,RAPGEF3,RASAL2,RIOK3,SEMA4G,SEMA6A,SENP7,SLC13A2,SLC38A2,SLC39A4,SSBP2,SULT1C2,SYT1,TCF7L2,TES,TFRC,TG,THBS1,THSD7A,TNC,TUBAL3,UTRN,VDR,VPS13D,WNT5A,ZFR,ZFX,ZFY,ZNF480,ZNF711	72
Cancer,Organismal Injury and Abnormalities,Reproductive System Disease	endometrioid endometrial adenocarcinoma	0.0000195			ABCC9,ABHD6,ANGPT2,CHN2,CPNE3,CXCL12,CYP4F2,CYP4F3,EIF2S1,ELF1,FAM198B,FILIP1L,GRB10,HNRNPL,IQGAP1,JAG1,KCNMA1,KIF13A,MAP4K5,MAST4,MBNL1,MECOM,MUC5AC,NEFH,PDE4DIP,PROX1,RASAL2,RIOK3,SENP7,SLC13A2,SRPX2,STAG2,TES,TG,THBS1,TNC,TPCN1,UBE3B,USH1C,UTRN,VDR,VLDLR,ZFR,ZFX,ZNF277,ZNF480	46
Cardiovascular System Development and Function,Organismal Development	patterning of blood vessel	0.0000197			CXCL12,CXCR4,EDNRA,FLT1,STK4	5
Cardiovascular System Development and Function	morphology of vessel component	0.0000198			ANGPT2,CALCRL,CXCL12,CXCR4,LTBP1,TGFB2	6
Cancer,Organismal Injury and Abnormalities,Respiratory Disease	lung tumor	0.0000199		1.061	ATRX,CEACAM1,CES2,CHN2,CXCR4,DLK1,DNAJB4,DPT,EIF2S1,FERMT1,FHL1,FLT1,GRB10,KCNMA1,LEPR,LTBP1,MAGI1,MAP4K5,MAST4,MECOM,NCOA2,NUPR1,PDCD4,PDCD6,PGGT1B,S100A4,SLC22A18,SSBP2,STAG2,TG,THBS1,THSD7A,TNC,UTRN,WNT5A	35
Cellular Movement	homing of stem cells	0.00002			CXCL12,CXCR4,S100A4	3
Organismal Functions	energy expenditure	0.0000215		-0.128	CEACAM1,CPE,CREB1,LEPR,PLCL2,RAPGEF3,TG,VDR	8
Cellular Movement	cell movement of melanoma cell lines	0.0000215		-1.979	CXCL12,CXCR4,MCAM,RAPGEF3,TGFB2,THBS1,TNC,WNT5A	8
Cell Death and Survival	cell viability	0.0000226		-1.144	ANGPT2,ATRX,CALCR,CDC40,CEACAM1,CREB1,CXCL12,CXCR4,DLK1,ELF1,FLT1,HNF1B,HSF2,ID4,IQGAP1,ITGB1BP1,JAG1,LEPR,MCAM,MECOM,NEFH,NUPR1,PDCD4,PRKACB,S100A4,SEMA6A,TANK,TGFB2,THBS1,VDR,WNT5A	31

Embryonic Development,Hematological System Development and Function,Lymphoid Tissue Structure and Development,Organ Development,Organismal Development,Tissue Development	formation of lymphoid tissue	0.0000229		-1.798	ADAM10,ANGPT2,C4A/C4B,CALCRL,CREB1,CXCL12,CXCR4,EDNRA,FLT1,GABPA,LYVE1,PROX1,STK4	13
Nervous System Development and Function,Neurological Disease	abnormal morphology of nervous system	0.0000235			CREB1,CXCL12,CXCR4,EDNRA,ENTPD1,JAG1,MBNL1,MBNL2,MECOM,NEFH,NFASC,PRKACB,RETREG1,SLC26A4,SMAD5,TCF7L2,TGFB2,USH1C,UTRN,VLDLR,WNT5A	21
Cancer,Cardiovascular Disease,Organismal Injury and Abnormalities	hemangioma	0.0000235			ANGPT2,CEACAM1,CXCL12,EDNRA,FLT1,JAG1,S100A4,THBS1	8
Cancer,Endocrine System Disorders,Organismal Injury and Abnormalities	malignant neoplasm of endocrine gland	0.0000235			AMY1C (includes others),ANGPT2,CES2,CREB1,CXCR4,FLT1,KCNMA1,MIA2,NEFH,PCM1,PDCD6,PDE4DIP,TAF13,TG,THBS1,UTRN,WNT5A	17
Cancer,Endocrine System Disorders,Organismal Injury and Abnormalities	thyroid carcinoma	0.0000239			AMY1C (includes others),ANGPT2,CES2,CREB1,CXCR4,FLT1,MIA2,NEFH,PCM1,PDE4DIP,TG,THBS1,WNT5A	13
Cancer,Organismal Injury and Abnormalities,Reproductive System Disease,Skeletal and Muscular Disorders	uterine leiomyoma	0.0000241			CALCRL,DPT,IQGAP1,LTBP1,RAB23,RAPGEF3,SMAD5,TAF13,TNC,UTRN,VDR	11
Cellular Movement,Skeletal and Muscular System Development and Function	migration of vascular smooth muscle cells	0.0000251		-1.14	ANGPT2,CALCRL,CLIC4,FHL1,FLT1,S100A4,THBS1	7
Cellular Movement	invasion of cells	0.0000253		-1.083	ADAM10,ANGPT2,CEACAM1,CXCL12,CXCR4,DNAJB4,FLT1,ID4,IQGAP1,KCNMA1,MAGI1,MCAM,NANOG,NCOA2,PDCD4,PROX1,PTGER2,S100A4,SMAD5,TGFB2,THBS1,TNC,VDR,WNT5A	24
Cancer,Gastrointestinal Disease,Hepatic System Disease,Organismal Injury and Abnormalities	hepatobiliary system cancer	0.0000254		0.64	ABCC9,ADAM10,ANGPT2,ATRX,BAG2,CALCRL,CDC40,CEACAM1,CES2,CHN2,CPNE3,CREB1,CXCL12,CXCR4,DPT,EDIL3,EDNRA,EFS,ENTPD1,FERMT1,FHL1,FLT1,GPX2,GRB10,ID4,IQGAP1,JAG1,KCNMA1,LEPR,LYVE1,MAP4K5,MAST4,MDFIC,ME2,MECOM,MIA2,MLXIPL,MUC5AC,NCOA2,NUPR1,OVGP1,PAMR1,PDE4DIP,PLCL2,PROX1,PROZ,RAB23,RAPGEF3,RASAL2,RIOK3,S100A4,SEMA6A,SLC26A4,SLC38A2,SLC39A4,SRPX2,STAG2,TCF7L2,TG,TGFB2,THBS1,TLK1,TM4SF1,TPCN1,UBE3B,USH1C,UTRN,VDR,VLDLR,VPS13D,ZFR,ZNF277	72
Connective Tissue Disorders,Organismal Injury and Abnormalities,Skeletal and Muscular Disorders	non-traumatic arthropathy	0.0000268			ADAM10,ADAMTS9,C4A/C4B,CALCRL,CXCL12,CXCR4,CYP4F3,FGL2,FLT1,GRB10,MAGI1,MUC5AC,NCOA2,PCM1,PTGER2,TAF13,TCF7L2,TFRC,TGFB2,THBS1,TNC,VDR,WNT5A	23
Reproductive System Development and Function	morphology of reproductive system	0.0000272			ATRX,CALCRL,DLK1,FHL5,FLT1,GRB10,MECOM,MLXIPL,NCOA2,NUPR1,PTGER2,RAPGEF3,SMAD5,STK4,TGFB2,THBS1,VDR,VLDLR,WNT5A	19

Cancer,Dermatological Diseases and Conditions,Organismal Injury and Abnormalities	skin tumor	0.0000277		-0.277	ABCC9,ABHD6,ABO,ADAM10,ADAMTS9,ALDH6A1,ALLC,ATRX,C4A/C4B,CALCR,CALCRL,CALU,CES2,CHN2,CLIC4,CPE,CPNE3,CXCL12,CYP4F2,CYP4F3,DLK1,EDIL3,EDNRA,EFS,ENPEP,ENTPD1,FAM198B,FAM216A,FERMT1,FGL2,FHL1,FHL5,FILIP1L,FLT1,GPX2,HNRNPL,HSF2,JAG1,KCNMA1,KIF13A,LEPR,LTBP1,MAGI1,MAP4K5,MAST4,MBNL1,MCAM,MECOM,MIA2,MLXIPL,MUC5AC,NCOA2,NEFH,NFASC,OVGP1,PAMR1,PDE4DIP,PLCL2,PRKACB,PROX1,PTGER2,RAPGEF3,RASAL2,RIOK3,S100A4,SEMA4G,SENP7,SLC13A2,SLC22A18,SLC39A4,SLCO2A1,SPAG5,SPTLC3,SULT1C2,SYT1,TCF7L2,TES,TFRC,TG,THBS1,THSD7A,TM4SF1,TNC,TSPAN5,TUBAL3,USH1C,UTRN,VDR,VLDLR,VPS13D,WNT5A,ZFR,ZFX,ZNF711	94
Cell Signaling,Molecular Transport,Vitamin and Mineral Metabolism	quantity of Ca2+	0.0000285		-0.985	ANGPT2,CALCR,CXCL12,CXCR4,EDNRA,EIF2S1,ENTPD1,LTBP1,MCAM,PTGER2,RAPGEF3,S100A4,VDR,WNT5A	14
Cancer,Organismal Injury and Abnormalities,Respiratory Disease	respiratory system tumor	0.0000287		1.061	ABCC9,ATRX,CALCR,CEACAM1,CES2,CHN2,CXCR4,DLK1,DNAJB4,DPT,EDIL3,EIF2S1,FERMT1,FHL1,FHL5,FLT1,GRB10,KCNMA1,LEPR,LTBP1,MAGI1,MAP4K5,MAST4,MECOM,NCOA2,NUPR1,PCM1,PDCD4,PDCD6,PGGT1B,PROX1,S100A4,SLC22A18,SSBP2,STAG2,TG,THBS1,THSD7A,TNC,UTRN,WNT5A	41
Cardiovascular System Development and Function,Tissue Morphology	morphology of blood vessel	0.0000298			ANGPT2,CALCRL,CXCL12,CXCR4,EDNRA,FLT1,ITGB1BP1,LTBP1,SMAD5,STK4,TGFB2,VDR	12
Cell Morphology,Cellular Function and Maintenance	permeability of cells	0.0000311		-0.505	ADAM10,ANGPT2,CREB1,CXCL12,ENTPD1,MCAM,RAPGEF3	7
Cardiovascular Disease,Organismal Injury and Abnormalities	abnormality of heart ventricle	0.0000314			ABCC9,ANGPT2,CXCR4,EDNRA,FLT1,MBNL1,MBNL2,PDCD4,SLCO2A1,STK4,TGFB2,THBS1,VDR	13
Cellular Movement	cell movement of muscle cells	0.0000345		-0.89	ANGPT2,CALCRL,CLIC4,CREB1,FHL1,FLT1,ITGB1BP1,S100A4,THBS1	9
Connective Tissue Disorders,Immunological Disease,Inflammatory Disease,Inflammatory Response,Organismal Injury and Abnormalities,Skeletal and Muscular Disorders	rheumatoid arthritis	0.0000349			ADAM10,C4A/C4B,CALCRL,CXCL12,CXCR4,CYP4F3,FGL2,FLT1,GRB10,MAGI1,NCOA2,PCM1,PTGER2,TAF13,TCF7L2,TFRC,TGFB2,THBS1,TNC,VDR,WNT5A	21
Cancer,Gastrointestinal Disease,Organismal Injury and Abnormalities	gastrointestinal tract cancer	0.0000352			ABCC9,ADAM10,ADAMTS9,ALDH6A1,ALLC,ANGPT2,ATRX,CALCR,CALCRL,CALU,CDC40,CEACAM1,CES2,CPE,CREB1,CXCL12,CXCR4,CYP4F2,DLK1,DNAJB4,DPT,EDIL3,EFS,ELF1,EMP1,ENOSF1,ENPEP,FAM198B,FAM216A,FERMT1,FHL1,FHL5,FILIP1L,FLT1,GGH,GPX2,GRB10,HNRNPL,IQGAP1,JAG1,KCNMA1,KIF13A,LEPR,LHPP,LTBP1,MAGI1,MAP4K5,MAST4,MBNL1,MBNL2,MCAM,MECOM,MIA2,MLXIPL,MUC5AC,NCOA2,NEFH,NFASC,NR1D2,OVGP1,PAMR1,PCM1,PDCD4,PDE4DIP,PGGT1B,PLCL2,PRKACB,PROX1,RAPGEF3,RASAL2,RIOK3,S100A4,SEMA4G,SEMA6A,SENP7,SLC13A2,SLC26A4,SLC38A2,SLC39A4,SLCO2A1,SPAG5,SPTLC3,SRPX2,SSBP2,STAG2,STK4,SULT1C2,SYT1,TAF13,TCF7L2,TES,TFRC,TG,TGFB2,THBS1,THSD7A,TLK1,TM4SF1,TMX1,TNC,TPCN1,TSPAN5,TUBAL3,UBE3B,UTRN,VDR,VLDLR,VPS13D,WNT5A,ZFR,ZFX,ZFY,ZNF480,ZNF711	114
Cellular Movement	migration of connective tissue cells	0.0000358		-1.003	CALCRL,CXCL12,CXCR4,DPT,ITGB1BP1,KCNMA1,PGGT1B,RAPGEF3,THBS1	9
Cell Signaling,Molecular Transport,Nucleic Acid Metabolism,Small Molecule Biochemistry	concentration of cyclic AMP	0.0000358		0.395	CALCRL,CXCL12,CXCR4,CYP4F2,ENTPD1,HNF1B,LEPR,PTGER2,THBS1	9

Organismal Development	abnormal morphology of body cavity	0.0000364			ADAM10,ADAMTS9,C4A/C4B,CALCRL,CEACAM1,CREB1,CXCR4,DLK1,EDNRA,FERMT1,IQGAP1,JAG1,LTBP1,MBNL1,MBNL2,MECOM,MUC5AC,NUPR1,PTGER2,RAPGEF3,SLCO2A1,SSBP2,TCF7L2,TGFB2,THBS1,USH1C,VDR,WNT5A	28
Skeletal and Muscular System Development and Function	relaxation of muscle	0.0000372		-0.816	KCNMA1,MBNL1,MBNL2,PTGER2,THBS1,VDR	6
Embryonic Development,Organ Development,Organismal Development,Reproductive System Development and Function,Tissue Development	development of genital organ	0.0000376			ATRX,CREB1,CXCL12,CXCR4,FHL5,HNF1B,HSF2,ID4,LEPR,NCOA2,NUPR1,PTGER2,SMAD5,TGFB2,VDR,WNT5A,ZFX	17
Cellular Movement	migration of breast cancer cell lines	0.0000384		-1.845	ANGPT2,CHN2,CPNE3,CXCL12,CXCR4,IQGAP1,MCAM,PDCD4,S100A4,SMAD5,WNT5A	11
Cardiovascular System Development and Function,Cell-To-Cell Signaling and Interaction	adhesion of endothelial cells	0.0000391		-0.643	ADAM10,ANGPT2,CXCL12,CXCR4,EDIL3,FLT1,MCAM,THBS1	8
Cell Morphology,Tissue Development	tubulation of cells	0.0000391		-0.766	CLIC4,CXCL12,CXCR4,FLT1,KCNMA1,PATJ,PROX1,TGFB2	8
Cancer,Neurological Disease,Organismal Injury and Abnormalities	growth of glioma	0.0000396		-1.342	CXCL12,CXCR4,DLK1,JAG1,WNT5A	5
Cardiovascular System Development and Function,Embryonic Development,Organ Development,Organismal Development,Tissue Development	formation of ventricular septum	0.0000397			CXCL12,CXCR4,LTBP1,PROX1,TGFB2,WNT5A	6
Cellular Development	differentiation of nervous system	0.0000402		-0.282	CREB1,CXCL12,CXCR4,ID4,JAG1,KCNMA1,NEFH,PROX1,RAPGEF3,S100A4,TCF7L2,TG,TGFB2,TNC,USH1C,WNT5A	16
Cancer,Dermatological Diseases and Conditions,Organismal Injury and Abnormalities	skin cancer	0.0000409		-0.762	ABCC9,ABHD6,ABO,ADAM10,ADAMTS9,ALDH6A1,ALLC,ATRX,C4A/C4B,CALCR,CALCRL,CALU,CES2,CHN2,CPE,CPNE3,CXCL12,CYP4F2,CYP4F3,DLK1,EDIL3,EDNRA,EFS,ENPEP,ENTPD1,FAM198B,FAM216A,FERMT1,FGL2,FHL1,FHL5,FILIP1L,FLT1,GPX2,HNRNPL,HSF2,JAG1,KCNMA1,KIF13A,LEPR,LTBP1,MAGI1,MAP4K5,MAST4,MBNL1,MCAM,MECOM,MIA2,MLXIPL,MUC5AC,NCOA2,NEFH,NFASC,OVGP1,PAMR1,PDE4DIP,PLCL2,PRKACB,PROX1,PTGER2,RAPGEF3,RASAL2,RIOK3,S100A4,SEMA4G,SENP7,SLC13A2,SLC22A18,SLC39A4,SLCO2A1,SPAG5,SPTLC3,SULT1C2,SYT1,TCF7L2,TES,TFRC,TG,THBS1,THSD7A,TM4SF1,TNC,TSPAN5,TUBAL3,USH1C,UTRN,VDR,VLDLR,VPS13D,WNT5A,ZFR,ZFX,ZNF711	93
Cell Morphology	sprouting	0.0000419		-1.466	ADAM10,ANGPT2,ATRX,CREB1,CXCL12,ITGB1BP1,NEFH,PROX1,SEMA6A,SRPX2,STK4,THBS1,TNC,WNT5A	14
Cell-To-Cell Signaling and Interaction,Cellular Assembly and Organization,Hematological System Development and Function,Immune Cell Trafficking,Infectious Diseases	fusion of mononuclear leukocytes	0.0000425			CXCL12,CXCR4,ENTPD1	3
Endocrine System Development and Function	glucose tolerance	0.0000427		0.05	ABCC9,CEACAM1,CPE,DLK1,GRB10,HNF1B,LEPR,PLCL2,RAPGEF3,TCF7L2,VDR	11
Cancer,Cardiovascular Disease,Organismal Injury and Abnormalities	vascular tumor	0.000044			ANGPT2,CEACAM1,CXCL12,EDNRA,FLT1,JAG1,S100A4,THBS1,VDR	9

Immunological Disease	systemic autoimmune syndrome	0.000044			ADAM10,C4A/C4B,CALCR,CALCRL,CEACAM1,CREB1,CXCL12,CXCR4,CYP4F3,FGL2,FLT1,GRB10,JAG1,MAGI1,NCOA2,PCM1,PDCD4,PTGER2,S100A4,TAF13,TCF7L2,TFRC,TGFB2,THBS1,TNC,VDR,WNT5A	27
Cancer,Endocrine System Disorders,Organismal Injury and Abnormalities	papillary thyroid carcinoma	0.0000442			CES2,CREB1,CXCR4,FLT1,PCM1,TG,THBS1,WNT5A	8
Cardiovascular System Development and Function,Cellular Development,Tissue Development	differentiation of endothelial cells	0.0000451			ANGPT2,CEACAM1,CXCR4,FLT1,JAG1,PROX1	6
Organismal Functions	energy homeostasis	0.0000455		-0.113	CEACAM1,CPE,CREB1,LEPR,NCOA2,PLCL2,RAPGEF3,TG,VDR	9
Cancer,Dermatological Diseases and Conditions,Organismal Injury and Abnormalities	cutaneous melanoma	0.0000455			ABCC9,ABHD6,ABO,ADAM10,ADAMTS9,ALDH6A1,ALLC,ATRX,C4A/C4B,CALCR,CALCRL,CALU,CES2,CHN2,CPE,CPNE3,CYP4F2,CYP4F3,DLK1,EDIL3,EDNRA,EFS,ENPEP,ENTPD1,FAM198B,FAM216A,FERMT1,FGL2,FHL1,FHL5,FILIP1L,FLT1,HNRNPL,HSF2,JAG1,KCNMA1,KIF13A,LEPR,LTBP1,MAGI1,MAP4K5,MAST4,MBNL1,MECOM,MIA2,MLXIPL,MUC5AC,NCOA2,NEFH,NFASC,OVGP1,PAMR1,PDE4DIP,PLCL2,PRKACB,PROX1,PTGER2,RAPGEF3,RASAL2,RIOK3,S100A4,SENP7,SLC13A2,SLC22A18,SLC39A4,SLCO2A1,SPAG5,SPTLC3,SULT1C2,SYT1,TCF7L2,TES,TFRC,TG,THBS1,THSD7A,TM4SF1,TNC,TSPAN5,TUBAL3,USH1C,UTRN,VDR,VLDLR,VPS13D,WNT5A,ZFR,ZFX,ZNF711	89
Cancer,Cellular Development,Cellular Growth and Proliferation,Organismal Injury and Abnormalities,Tumor Morphology	proliferation of tumor cells	0.0000455		-0.549	ADAM10,CEACAM1,CXCL12,CXCR4,DLK1,FHL1,JAG1,MCAM,PROX1,TCF7L2,TG,TGFB2,THBS1,VDR,WNT5A	15
Metabolic Disease	glucose metabolism disorder	0.0000456		-0.305	ABCC9,ADAMTS9,C4A/C4B,CALCR,CEACAM1,CHN2,CPE,CREB1,CXCL12,CXCR4,CYP4F2,DLK1,EDNRA,ENPEP,FLT1,HNF1B,LEPR,MLXIPL,PDCD4,PLCL2,PROX1,PTGER2,RAPGEF3,S100A4,TAF13,TCF7L2,THBS1,TNC,TSPAN5,VDR	30
Organismal Development	formation of vessel	0.000047		-1.91	ANGPT2,CREB1,CXCL12,CXCR4,EMP1,FLT1,JAG1,LTBP1,TGFB2	9
Cardiovascular System Development and Function,Organismal Development,Tissue Morphology	morphology of endothelial tissue	0.0000512			CLIC4,CXCR4,FLT1,SMAD5,STK4,TGFB2	6
Lipid Metabolism,Small Molecule Biochemistry	omega-hydroxylation of very long chain fatty acid	0.0000516			CYP4F2,CYP4F3	2
Cellular Movement,Immune Cell Trafficking	invasion of B cell hybridoma cells	0.0000516			CXCL12,CXCR4	2
Cell Morphology,Cellular Development,Tissue Development	tubulation of brain cancer cell lines	0.0000516			CXCL12,CXCR4	2

Cellular Movement	extravasation of colorectal cancer cell lines	0.0000516			CXCL12,CXCR4	2
Cellular Movement	homing of progenitor cells	0.0000516			CXCL12,CXCR4	2
Cell-To-Cell Signaling and Interaction,Cellular Assembly and Organization,Hematological System Development and Function,Immune Cell Trafficking,Infectious Diseases	fusion of thymocytes	0.0000516			CXCL12,CXCR4	2
Cellular Movement,Embryonic Development	migration of dental pulp stem cells	0.0000516			CXCL12,CXCR4	2
Cell-mediated Immune Response,Cellular Movement,Hematological System Development and Function,Hematopoiesis,Immune Cell Trafficking	migration of single positive thymocytes	0.0000516			CXCL12,HNRNPL	2
Cellular Movement	extravasation of hepatoma cell lines	0.0000516			CXCL12,CXCR4	2
Amino Acid Metabolism,Molecular Transport,Small Molecule Biochemistry	transport of asparagine	0.0000516			SLC38A2,SLC38A3	2
Cell-mediated Immune Response,Cellular Movement,Hematological System Development and Function,Hematopoiesis,Immune Cell Trafficking,Inflammatory Response,Lymphoid Tissue Structure and Development	chemorepulsion of thymocytes	0.0000516			CXCL12,CXCR4	2
Cancer,Gastrointestinal Disease,Organismal Injury and Abnormalities	colon cancer	0.0000523			ADAM10,ADAMTS9,ALDH6A1,ALLC,ANGPT2,ATRX,CALCR,CEACAM1,CES2,CYP4F2,EDIL3,ELF1,ENPEP,FAM216A,FERMT1,FHL1,FLT1,GGH,GPX2,HNRNPL,JAG1,KCNMA1,KIF13A,LEPR,LTBP1,MAGI1,MAP4K5,MAST4,MBNL1,MCAM,MECOM,MIA2,MLXIPL,MUC5AC,NCOA2,NEFH,NFASC,NR1D2,OVGP1,PCM1,PDCD4,PDE4DIP,PGGT1B,RAPGEF3,RASAL2,RIOK3,SEMA4G,SEMA6A,SENP7,SLC13A2,SLC38A2,SLC39A4,SSBP2,SULT1C2,SYT1,TCF7L2,TES,TFRC,TG,THBS1,TNC,TUBAL3,UTRN,VPS13D,WNT5A,ZFR,ZFX,ZFY,ZNF480,ZNF711	70
Tissue Development	branching of epithelial tissue	0.0000565		-1.284	ANGPT2,CLIC4,HNF1B,ITGB1BP1,PROX1,SRPX2,TNC	7
Cardiovascular System Development and Function	vascularization	0.0000577		-0.543	CEACAM1,CLIC4,CXCL12,CXCR4,FLT1,S100A4,SMAD5,STK4,THBS1,VLDLR	10
Cellular Assembly and Organization,Tissue Development	fibrogenesis	0.0000583		0.637	CREB1,CXCL12,EDNRA,FHL1,ITGB1BP1,JAG1,MAGI1,NEFH,PDCD4,PROX1,RAPGEF3,STK4,TNC,TPM4	14

Cellular Development,Connective Tissue Development and Function,Skeletal and Muscular System Development and Function,Tissue Development	differentiation of osteoclasts	0.0000592		-1.195	CALCR,CEACAM1,CREB1,JAG1,PTGER2,RAPGEF3,TFRC,TNC,WNT5A	9
Skeletal and Muscular System Development and Function	muscle contraction	0.0000594		0.816	ANGPT2,C4A/C4B,CALCRL,CXCR4,EDNRA,ENTPD1,KCNMA1,SMAD5,UTRN,VDR	10
Cancer,Organismal Injury and Abnormalities	endometrioid carcinoma	0.0000599			ABCC9,ABHD6,ANGPT2,ATRX,CES2,CHN2,CPE,CPNE3,CXCL12,CYP4F2,CYP4F3,EIF2S1,ELF1,FAM198B,FILIP1L,FLT1,GRB10,HNRNPL,IQGAP1,JAG1,KCNMA1,KIF13A,MAP4K5,MAST4,MBNL1,MECOM,MUC5AC,NEFH,PDE4DIP,PROX1,RASAL2,RIOK3,SENP7,SLC13A2,SLC22A18,SRPX2,STAG2,TES,TG,THBS1,TM4SF1,TNC,TPCN1,TUBAL3,UBE3B,USH1C,UTRN,VDR,VLDLR,ZFR,ZFX,ZNF277,ZNF480	53
Cancer,Organismal Injury and Abnormalities	adenoma formation	0.0000607			ATRX,C4A/C4B,CES2,DPT,ENPEP,ID4,LEPR,MCAM,MECOM,PTGER2,SLC39A4,SSBP2,STAG2,TAF13,TG,THBS1,VDR	17
Cancer,Organismal Injury and Abnormalities	serous neoplasm	0.000061			ABCC9,ATRX,C4A/C4B,CXCL12,DPT,EDNRA,ENPEP,ENTPD1,FGL2,FILIP1L,KCNMA1,MCAM,MECOM,MUC5AC,PTGER2,SLC39A4,STAG2,TG	18
Cardiovascular Disease,Cardiovascular System Development and Function,Organ Morphology,Organismal Development,Organismal Injury and Abnormalities	abnormal morphology of heart	0.0000611			ADAM10,ADAMTS9,CALCRL,CXCR4,EDNRA,JAG1,LTBP1,MBNL1,MBNL2,MECOM,SLCO2A1,TGFB2,THBS1	13
Organismal Injury and Abnormalities,Reproductive System Disease	disorder of pregnancy	0.0000616			C4A/C4B,CALCRL,CHN2,DLK1,FERMT1,FHL1,FHL5,FLT1,GRB10,MECOM,MSMO1,NCOA2,PTGER2,STK4,TMX1,TNC,VDR	17
Endocrine System Disorders,Metabolic Disease	insulin resistance	0.0000633		-0.24	CEACAM1,CPE,CREB1,DLK1,HNF1B,LEPR,MLXIPL,PLCL2,RAPGEF3,S100A4,THBS1,VDR	12
Cell Death and Survival	cell death	0.0000651		-0.561	ABCC9,ABO,ADAM10,ANGPT2,ATRX,C4A/C4B,CALCR,CALCRL,CEACAM1,CLIC4,CREB1,CXCL12,CXCR4,DLK1,EDIL3,EDNRA,EIF2S1,ELF1,EMP1,ENTPD1,FGL2,FLT1,GPX2,GRB10,HNF1B,HSF2,JAG1,KCNMA1,LEPR,LTBP1,MAGI1,MCAM,MECOM,NANOG,NCOA2,NEFH,NUPR1,PDCD4,PDCD6,PTGER2,RAPGEF3,RETREG1,S100A4,SEMA6A,SLC26A4,SMAD5,SPAG5,STK4,TANK,TCF7L2,TFRC,TGFB2,THBS1,TMX1,TNC,VDR,WNT5A,ZFR	58
Connective Tissue Disorders,Inflammatory Disease,Inflammatory Response,Organismal Injury and Abnormalities,Skeletal and Muscular Disorders	inflammation of joint	0.0000658		-0.627	ADAM10,ADAMTS9,C4A/C4B,CALCRL,CPE,CXCL12,CXCR4,CYP4F3,FGL2,FLT1,GRB10,LEPR,MAGI1,MUC5AC,NCOA2,PCM1,PTGER2,TAF13,TCF7L2,TFRC,TGFB2,THBS1,TNC,VDR,WNT5A	25
Cellular Development,Cellular Growth and Proliferation	proliferation of blood cells	0.0000684		0.982	CEACAM1,CREB1,CXCL12,CXCR4,EFS,ELF1,FGL2,HNRNPL,JAG1,LEPR,LTBP1,MECOM,PLCL2,SMAD5,STK4,TANK,TFRC,TG,TGFB2,THBS1,VDR,WNT5A	22
Organismal Injury and Abnormalities,Renal and Urological Disease	cystic kidney disease	0.0000692			ADGRL3,ANGPT2,C4A/C4B,CPE,FHL1,FILIP1L,GPX2,HNF1B,PDCD4,RAPGEF3,VLDLR	11
Cardiovascular System Development and Function,Cell-To-Cell Signaling and Interaction	binding of endothelial cells	0.0000695		-0.317	ADAM10,ANGPT2,CXCL12,CXCR4,EDIL3,FLT1,LEPR,MCAM,THBS1	9
Cell Signaling,Post-Translational Modification	activation of Protein kinase	0.0000702			CEACAM1,CXCR4,EDNRA,IQGAP1,ITGB1BP1,MAP4K5,MDFIC,THBS1,VLDLR,WNT5A	10

Cell-To-Cell Signaling and Interaction,Cellular Movement	recruitment of blood cells	0.0000739		-0.304	ADAM10,ANGPT2,C4A/C4B,CXCL12,CXCR4,EDIL3,ENTPD1,FLT1,S100A4,THBS1,TNC,VDR	12
Digestive System Development and Function,Organ Morphology,Organismal Development	morphology of intestine	0.0000741			C4A/C4B,CEACAM1,EFS,FERMT1,MUC5AC,TCF7L2,TGFB2,USH1C,VDR,WNT5A	10
Cardiovascular System Development and Function,Digestive System Development and Function,Organismal Development,Tissue Morphology	morphology of bile duct	0.000077			HNF1B,JAG1,THBS1	3
Skeletal and Muscular System Development and Function	function of smooth muscle	0.0000775		0	ABCC9,ENTPD1,KCNMA1,NCOA2,PTGER2,THBS1	6
Digestive System Development and Function	morphology of digestive system	0.0000785			C4A/C4B,CEACAM1,DLK1,EDNRA,EFS,FERMT1,HNF1B,IQGAP1,JAG1,LEPR,MLXIPL,MUC5AC,RAPGEF3,TCF7L2,TGFB2,THBS1,USH1C,VDR,WNT5A	19
Cancer,Gastrointestinal Disease,Organismal Injury and Abnormalities	stomach tumor	0.0000802			ABCC9,ADAM10,ADAMTS9,ANGPT2,CES2,CXCL12,CYP4F2,FILIP1L,FLT1,HNRNPL,KCNMA1,MAST4,MBNL2,MUC5AC,NCOA2,NFASC,PDE4DIP,SEMA4G,SLC13A2,SPTLC3,SSBP2,TES,TG,THBS1,THSD7A,TNC,UBE3B,UTRN,VLDLR,WNT5A,ZNF480	31
Cancer,Organismal Injury and Abnormalities	squamous-cell carcinoma	0.0000811		-0.218	ABCC9,ABO,ADAM10,ALLC,ATRX,CALCR,CES2,CHN2,CXCL12,CYP4F3,DPT,EDIL3,ENOSF1,ENPEP,FAM198B,FERMT1,FGL2,FHL5,FILIP1L,GPX2,GRB10,JAG1,KCNMA1,LEPR,MAGI1,MAST4,MBNL1,MBNL2,MCAM,ME2,MECOM,MUC5AC,NEFH,NR1D2,OVGP1,PCM1,PDCD4,PDE4DIP,PROX1,PTGER2,RASAL2,RETREG1,S100A4,SEMA4G,SEMA6A,SLCO2A1,SSBP2,STAG2,TG,THBS1,THSD7A,TM4SF1,TNC,USH1C,WNT5A	55
Cardiovascular System Development and Function	morphogenesis of cardiovascular system	0.0000826			ANGPT2,CLIC4,CXCR4,FLT1,MECOM,PROX1,SMAD5,TGFB2,THBS1,WNT5A	10
Developmental Disorder,Hereditary Disorder,Organismal Injury and Abnormalities,Renal and Urological Disease	Polycystic Kidney Disease	0.0000826			ADGRL3,ANGPT2,C4A/C4B,CPE,FHL1,FILIP1L,GPX2,HNF1B,RAPGEF3,VLDLR	10
Cell Cycle	cell cycle progression	0.000084		-1.14	CEACAM1,CHN2,CREB1,CXCL12,DNAJB4,EMP1,FLT1,GRB10,LTBP1,MECOM,MLXIPL,NANOG,NUPR1,PCM1,PGGT1B,PROX1,RAPGEF3,S100A4,SPAG5,SSBP2,TCF7L2,TGFB2,THBS1,TNC,ZFR	25
Cancer,Organismal Injury and Abnormalities,Reproductive System Disease	mammary tumor	0.0000855		-0.958	ABCC9,ABO,ADAMTS9,ANGPT2,ATRX,CES2,CPE,CPNE3,CXCL12,CXCR4,FAM198B,FLT1,JAG1,LEPR,LYVE1,MAGI1,MAST4,MCAM,MECOM,PDCD6,PROX1,PTGER2,S100A4,SENP7,SLC22A18,STAG2,TCF7L2,TES,TGFB2,THBS1,TM4SF1,TNC,UTRN,VDR,VLDLR,VPS13D,WNT5A	37
Cellular Movement	migration of leukemia cell lines	0.0000868		-1.399	ADAM10,CXCL12,CXCR4,IQGAP1,TNC,WNT5A	6
Cellular Assembly and Organization	formation of cytoskeleton	0.0000894		0	CREB1,CXCL12,EDNRA,ITGB1BP1,JAG1,MAGI1,NEFH,RAPGEF3,STK4,THBS1,TNC,TPM4,UTRN	13
Cellular Movement	release of cells	0.0000903			CXCL12,CXCR4,SYT1,TFRC	4

Cellular Movement,Skeletal and Muscular System Development and Function	cell movement of smooth muscle cells	0.0000905		-0.89	ANGPT2,CALCRL,CLIC4,CREB1,FHL1,FLT1,S100A4,THBS1	8
Cellular Movement,Skeletal and Muscular System Development and Function	migration of muscle cells	0.0000905		-1.169	ANGPT2,CALCRL,CLIC4,FHL1,FLT1,ITGB1BP1,S100A4,THBS1	8
Developmental Disorder,Organismal Injury and Abnormalities	congenital malformation of genitourinary system	0.0000911		0	ADGRL3,ANGPT2,ATRX,C4A/C4B,CPE,FHL1,FILIP1L,GPX2,HNF1B,RAPGEF3,TGFB2,VDR,VLDLR	13
Cardiovascular Disease,Cardiovascular System Development and Function,Organismal Development,Organismal Injury and Abnormalities,Tissue Morphology	abnormal morphology of endothelial tissue	0.0000915			CLIC4,FLT1,SMAD5,STK4,TGFB2	5
Cellular Development,Cellular Growth and Proliferation,Hematological System Development and Function,Lymphoid Tissue Structure and Development	cell proliferation of T lymphocytes	0.0000926		0.773	CEACAM1,CREB1,CXCL12,CXCR4,EFS,ELF1,FGL2,HNRNPL,JAG1,LEPR,LTBP1,STK4,TFRC,TG,TGFB2,THBS1,VDR	17
Cancer,Gastrointestinal Disease,Organismal Injury and Abnormalities	malignant neoplasm of large intestine	0.0000931			ABCC9,ADAM10,ADAMTS9,ALDH6A1,ALLC,ANGPT2,ATRX,CALCR,CALCRL,CALU,CDC40,CEACAM1,CES2,CREB1,CXCL12,CYP4F2,DLK1,DNAJB4,DPT,EDIL3,EFS,ELF1,EMP1,ENOSF1,ENPEP,FAM198B,FAM216A,FERMT1,FHL1,FHL5,FILIP1L,FLT1,GGH,GPX2,GRB10,HNRNPL,IQGAP1,JAG1,KCNMA1,KIF13A,LEPR,LHPP,LTBP1,MAGI1,MAP4K5,MAST4,MBNL1,MBNL2,MCAM,MECOM,MIA2,MLXIPL,MUC5AC,NCOA2,NEFH,NFASC,NR1D2,OVGP1,PAMR1,PCM1,PDCD4,PDE4DIP,PGGT1B,PLCL2,PROX1,RAPGEF3,RASAL2,RIOK3,S100A4,SEMA4G,SEMA6A,SENP7,SLC13A2,SLC26A4,SLC38A2,SLC39A4,SLCO2A1,SPAG5,SRPX2,SSBP2,STAG2,STK4,SULT1C2,SYT1,TAF13,TCF7L2,TES,TFRC,TG,TGFB2,THBS1,THSD7A,TLK1,TM4SF1,TMX1,TNC,TSPAN5,TUBAL3,UBE3B,UTRN,VDR,VLDLR,VPS13D,WNT5A,ZFR,ZFX,ZFY,ZNF480,ZNF711	109
Molecular Transport	quantity of metal	0.0000946		-1.305	ANGPT2,CALCR,CXCL12,CXCR4,EDNRA,EIF2S1,ENTPD1,LTBP1,MCAM,PTGER2,RAPGEF3,S100A4,TFRC,VDR,WNT5A	15
Connective Tissue Development and Function,Tissue Morphology	quantity of connective tissue	0.0000968		0.382	ANGPT2,CALCR,CEACAM1,CPE,DLK1,DPT,ID4,ITGB1BP1,LEPR,MLXIPL,NCOA2,PLCL2,RAPGEF3,SMAD5,TFRC,VDR,WNT5A	17
Organismal Development	morphology of head	0.0000976			ADAMTS9,ATRX,CLIC4,CXCL12,CXCR4,DLK1,DPT,EDNRA,ENTPD1,HSF2,ITGB1BP1,JAG1,LEPR,MECOM,MUC5AC,S100A4,SLC26A4,SMAD5,TCF7L2,TGFB2,THBS1,USH1C,VLDLR,WNT5A	24
Cancer,Gastrointestinal Disease,Hepatic System Disease,Organismal Injury and Abnormalities	liver cancer	0.0000984		1.342	ABCC9,ANGPT2,ATRX,BAG2,CALCRL,CDC40,CEACAM1,CES2,CHN2,CPNE3,CREB1,CXCL12,CXCR4,DPT,EDIL3,EDNRA,EFS,ENTPD1,FERMT1,FHL1,FLT1,GPX2,GRB10,ID4,IQGAP1,JAG1,KCNMA1,LEPR,LYVE1,MAST4,MDFIC,ME2,MECOM,MIA2,MLXIPL,MUC5AC,NCOA2,NUPR1,OVGP1,PAMR1,PDE4DIP,PLCL2,PROX1,PROZ,RAB23,RAPGEF3,RASAL2,RIOK3,S100A4,SEMA6A,SLC26A4,SLC38A2,SLC39A4,SRPX2,STAG2,TCF7L2,TG,TGFB2,THBS1,TLK1,TPCN1,UBE3B,USH1C,UTRN,VDR,VLDLR,VPS13D,ZFR,ZNF277	69
Cardiovascular System Development and Function,Cell Morphology,Organismal Development,Tissue Morphology	morphology of endothelial cells	0.0000987			CLIC4,CXCR4,FLT1,SMAD5,STK4	5

Cardiovascular System Development and Function,Cellular Movement	cell movement of endothelial progenitor cells	0.0000996			CXCL12,MCAM,PROX1	3
Cardiovascular Disease,Hematological Disease,Organismal Injury and Abnormalities	thrombus	0.0001		-1.573	ABO,ANGPT2,CEACAM1,EDNRA,ENTPD1,PROZ,PTGER2,THBS1,VDR	9
Cardiovascular System Development and Function,Organ Morphology,Organismal Development	morphology of heart ventricle	0.000101			ANGPT2,CXCL12,CXCR4,MBNL1,MBNL2,RAPGEF3,SLCO2A1,STK4,TGFB2,THBS1,VDR	11
Cardiovascular System Development and Function,Embryonic Development,Hematological System Development and Function,Lymphoid Tissue Structure and Development,Organ Development,Organismal Development,Tissue Development	development of lymph vessel	0.000102			ANGPT2,CXCL12,FLT1,PROX1	4
Cardiovascular Disease,Cardiovascular System Development and Function,Organ Morphology,Organismal Development,Organismal Injury and Abnormalities	enlargement of heart	0.000104			ABCC9,ANGPT2,CREB1,CXCL12,EDNRA,FLT1,LEPR,MBNL1,MBNL2,RAPGEF3,TG,VDR	12
Developmental Disorder,Hereditary Disorder,Organismal Injury and Abnormalities,Renal and Urological Disease	autosomal dominant polycystic kidney disease	0.000108			ADGRL3,ANGPT2,CPE,FHL1,FILIP1L,VLDLR	6
Cancer,Endocrine System Disorders,Organismal Injury and Abnormalities	thyroid gland tumor	0.00011			AMY1C (includes others),ANGPT2,CES2,CREB1,CXCR4,FLT1,MIA2,NEFH,PCM1,PDCD6,PDE4DIP,TAF13,TG,THBS1,UTRN,WNT5A	16
Cancer,Hematological Disease,Organismal Injury and Abnormalities	lymphocytic neoplasm	0.000112		1.457	ABCC9,ADAMTS9,ANGPT2,BAG2,CXCL12,CXCR4,DPT,FERMT1,FILIP1L,FLT1,HIST1H2BK,HNF1B,IQGAP1,LTBP1,MAGI1,MAP4K5,MCAM,MIA2,MLXIPL,MSMO1,NCOA2,PDCD4,RSPH14,S100A4,SMAD5,SSBP2,TAF13,TFRC,TGFB2,THBS1,TLK1,ZNF711	32
Cell-To-Cell Signaling and Interaction	activation of tumor cell lines	0.000114		-0.861	ANGPT2,CXCR4,ENPEP,IQGAP1,THBS1,WNT5A	6
Organismal Development	growth of organism	0.000115		-0.495	ATRX,CLIC4,CREB1,CXCL12,DLK1,FLT1,GRB10,HNF1B,ID4,JAG1,KCNMA1,LEPR,NANOG,S100A4,STK4,TCF7L2,TFRC,TNC,VDR,WNT5A,ZFX	21
Cardiovascular System Development and Function,Organismal Development	formation of blood vessel	0.000115		-1.941	CREB1,CXCL12,CXCR4,EMP1,FLT1,JAG1,LTBP1,TGFB2	8
Cardiovascular System Development and Function,Hematological System Development and Function	blood pressure	0.000116		-0.429	ABCC9,ANGPT2,CXCL12,CYP4F2,EDNRA,KCNMA1,LEPR,PTGER2,STK4,THBS1,VDR	11
Cancer,Gastrointestinal Disease,Organismal Injury and Abnormalities	oral cancer	0.000119			ADAM10,CXCL12,DPT,EDIL3,ENPEP,PRKACB,SSBP2,TLK1,TM4SF1,TNC	10

Tissue Morphology	morphology of connective tissue	0.00012			ADAM10,CEACAM1,DLK1,DPT,JAG1,LEPR,MECOM,MLXIPL,NCOA2,RAPGEF3,S100A4,TFRC,TG,TGFB2,THBS1,VDR,VLDLR,WNT5A	18
Cellular Growth and Proliferation,Tissue Development	proliferation of epithelial cells	0.000121		-0.235	CEACAM1,CXCL12,CXCR4,EDNRA,FERMT1,FLT1,HNF1B,NCOA2,PROX1,PTGER2,TCF7L2,TGFB2,THBS1,VDR,WNT5A	15
Cancer,Gastrointestinal Disease,Hepatic System Disease,Organismal Injury and Abnormalities	liver tumor	0.000123		1.342	ABCC9,ANGPT2,ATRX,BAG2,CALCRL,CALU,CDC40,CEACAM1,CES2,CHN2,CPNE3,CREB1,CXCL12,CXCR4,DPT,EDIL3,EDNRA,EFS,ENTPD1,FERMT1,FHL1,FLT1,GPX2,GRB10,ID4,IQGAP1,JAG1,KCNMA1,LEPR,LYVE1,MAST4,MDFIC,ME2,MECOM,MIA2,MLXIPL,MUC5AC,NCOA2,NUPR1,OVGP1,PAMR1,PDE4DIP,PLCL2,PROX1,PROZ,RAB23,RAPGEF3,RASAL2,RIOK3,S100A4,SEMA6A,SLC26A4,SLC38A2,SLC39A4,SRPX2,STAG2,TCF7L2,TG,TGFB2,THBS1,TLK1,TPCN1,UBE3B,USH1C,UTRN,VDR,VLDLR,VPS13D,ZFR,ZNF277	70
Embryonic Development,Hematological System Development and Function,Lymphoid Tissue Structure and Development,Organ Development,Organismal Development,Tissue Development	lymphangiogenesis	0.000123		-1.154	ANGPT2,CXCL12,FLT1,LYVE1,PROX1	5
Inflammatory Response,Organismal Injury and Abnormalities	inflammation of organ	0.000123		0.201	ABHD6,ANGPT2,C4A/C4B,CEACAM1,CREB1,CXCL12,CXCR4,EDNRA,EFS,ELF1,ENTPD1,FERMT1,FLT1,GPX2,HSF2,JAG1,LEPR,MCAM,PDCD4,PTGER2,RAPGEF3,S100A4,SLC39A4,TAF13,TANK,TFRC,THBS1,UTRN,VDR	29
Cancer,Organismal Injury and Abnormalities,Respiratory Disease	lung cancer	0.000124		1.114	ATRX,CEACAM1,CES2,CHN2,CXCR4,DLK1,DNAJB4,DPT,FERMT1,FHL1,FLT1,GRB10,KCNMA1,LEPR,LTBP1,MAGI1,MAP4K5,MAST4,MECOM,NCOA2,NUPR1,PDCD4,PDCD6,S100A4,SLC22A18,SSBP2,STAG2,TG,THBS1,THSD7A,UTRN,WNT5A	32
Cancer,Organismal Injury and Abnormalities	metastatic solid tumor	0.000126		-0.369	ANGPT2,C4A/C4B,CXCL12,CXCR4,EFS,EMP1,FLT1,JAG1,MAGI1,MAP4K5,S100A4,TGFB2,THBS1,TM4SF1,TNC,VDR,WNT5A	17
Cancer,Neurological Disease,Organismal Injury and Abnormalities	glioblastoma	0.000126			ANGPT2,ATRX,FLT1,KCNMA1,LHPP,MECOM,S100A4,STAG2,TAF13,TCF7L2,TGFB2,TNC,USH1C	13
Cancer,Cellular Movement,Organismal Injury and Abnormalities,Tumor Morphology	invasion of melanoma cells	0.000126			CEACAM1,CXCL12,MCAM	3
Cancer,Cellular Movement,Organismal Injury and Abnormalities,Tumor Morphology	invasion of tumor cells	0.000128		-1.142	CEACAM1,CXCL12,CXCR4,MCAM,PDCD4,PROX1,THBS1,WNT5A	8
Cardiovascular Disease,Cardiovascular System Development and Function,Organismal Injury and Abnormalities,Skeletal and Muscular Disorders,Skeletal and Muscular System Development and Function,Tissue Morphology	abnormal morphology of vascular smooth muscle	0.000128			CALCRL,CXCL12,CXCR4,LTBP1	4
Digestive System Development and Function,Gastrointestinal Disease,Organ Morphology,Organismal Development,Organismal Injury and Abnormalities	abnormal morphology of intestine	0.000131			C4A/C4B,CEACAM1,FERMT1,MUC5AC,TCF7L2,TGFB2,USH1C,VDR,WNT5A	9
Cellular Development,Connective Tissue Development and Function,Tissue Development	differentiation of adipocytes	0.000138		-0.146	ALDH6A1,CREB1,DLK1,ID4,JAG1,MECOM,STK4,TCF7L2,VDR,WNT5A	10

Developmental Disorder,Embryonic Development,Organismal Development,Tissue Morphology	abnormal morphology of embryonic tissue	0.00014			ADAM10,ADAMTS9,CXCR4,EDNRA,HNF1B,JAG1,LTBP1,MECOM,PRKACB,SLC26A4,SMAD5,STK4,TFRC,TGFB2,VLDLR,WNT5A	16
Cell Death and Survival	apoptosis	0.000141		0.437	ABO,ANGPT2,ATRX,CALCR,CALCRL,CEACAM1,CLIC4,CREB1,CXCL12,CXCR4,EDIL3,EDNRA,EIF2S1,ENTPD1,FGL2,FLT1,GPX2,GRB10,HNF1B,HSF2,JAG1,KCNMA1,LEPR,LTBP1,MCAM,MECOM,NANOG,NCOA2,NUPR1,PDCD4,PDCD6,PTGER2,RAPGEF3,RETREG1,S100A4,SMAD5,SPAG5,STK4,TCF7L2,TFRC,TGFB2,THBS1,TMX1,TNC,VDR,WNT5A,ZFR	47
Cancer,Cellular Development,Cellular Growth and Proliferation,Neurological Disease,Organismal Injury and Abnormalities,Tumor Morphology	proliferation of glioma cells	0.000142		-1	CXCR4,DLK1,JAG1,WNT5A	4
Cellular Growth and Proliferation	colony formation	0.000147		-0.126	CEACAM1,CXCL12,FLT1,ID4,IQGAP1,MCAM,MECOM,NANOG,NUPR1,S100A4,SMAD5,STK4,TCF7L2,TES,TGFB2,TNC	16
Cellular Development,Nervous System Development and Function	differentiation of oligodendrocytes	0.000147		0.808	CXCR4,ID4,RAPGEF3,TCF7L2,TG,TNC	6
Cancer	hyperplasia of secretory structure	0.000151		-0.342	CXCL12,PTGER2,RAPGEF3,TCF7L2,TG,TGFB2,THBS1,VDR	8
Cellular Movement	dissemination of cells	0.000151			ANGPT2,CXCL12,CXCR4,SENP7,WNT5A	5
Cancer,Gastrointestinal Disease,Organismal Injury and Abnormalities	gastric cancer	0.000152			ABCC9,ADAM10,ADAMTS9,ANGPT2,CES2,CXCL12,CYP4F2,FILIP1L,FLT1,HNRNPL,KCNMA1,MAST4,MBNL2,MUC5AC,NCOA2,NFASC,PDE4DIP,SEMA4G,SLC13A2,SPTLC3,SSBP2,TG,THBS1,THSD7A,TNC,UBE3B,UTRN,VLDLR,WNT5A,ZNF480	30
Cellular Development,Cellular Growth and Proliferation,Hematological System Development and Function,Lymphoid Tissue Structure and Development	proliferation of lymphocytes	0.000152		1.263	CEACAM1,CREB1,CXCL12,CXCR4,EFS,ELF1,FGL2,HNRNPL,JAG1,LEPR,LTBP1,PLCL2,STK4,TANK,TFRC,TG,TGFB2,THBS1,VDR	19
Cell-To-Cell Signaling and Interaction,Hematological System Development and Function	binding of CD4+ T-lymphocytes	0.000154			CXCL12,CXCR4	2
Small Molecule Biochemistry	abnormal activity of renin	0.000154			PTGER2,VDR	2
Cellular Function and Maintenance	retention of myeloid cells	0.000154			CXCL12,CXCR4	2
Cellular Function and Maintenance,Hematological System Development and Function,Hematopoiesis	retention of hematopoietic progenitor cells	0.000154			CXCL12,CXCR4	2

Cardiovascular System Development and Function,Cellular Movement,Immune Cell Trafficking,Lymphoid Tissue Structure and Development	chemotaxis of lymphatic endothelial cells	0.000154			CXCL12,PROX1	2
Cellular Movement	migration of gastrointestinal stromal tumor cell lines	0.000154			CXCL12,CXCR4	2
Cell Morphology	polarization of brain cancer cell lines	0.000154			CXCL12,CXCR4	2
Cellular Movement,Nervous System Development and Function	chemotaxis of cerebellar granule cell	0.000154			CXCL12,CXCR4	2
Cellular Assembly and Organization,Tissue Development	formation of filaments	0.000154		0.392	CREB1,CXCL12,EDNRA,FHL1,ITGB1BP1,JAG1,MAGI1,NEFH,PROX1,RAPGEF3,STK4,TNC,TPM4	13
Cellular Movement	chemorepulsion	0.000158			CXCL12,CXCR4,FLT1,WNT5A	4
Cell-mediated Immune Response,Cellular Movement,Hematological System Development and Function,Immune Cell Trafficking	T cell migration	0.00016		-1.328	ADAM10,CEACAM1,CXCL12,CXCR4,EFS,HNRNPL,MCAM,S100A4,THBS1,WNT5A	10
Cancer,Organismal Injury and Abnormalities	serous adenocarcinoma	0.000168			ATRX,C4A/C4B,CXCL12,DPT,EDNRA,ENPEP,ENTPD1,FGL2,FILIP1L,KCNMA1,MCAM,MECOM,MUC5AC,PTGER2,SLC39A4,STAG2,TG	17
Cardiovascular System Development and Function,Organ Morphology,Organismal Development	enlargement of heart ventricle	0.00017			ANGPT2,CXCL12,MBNL1,MBNL2,RAPGEF3,STK4,VDR	7
Cancer,Gastrointestinal Disease,Organismal Injury and Abnormalities	growth of digestive organ tumor	0.00017		-0.492	ADAM10,CREB1,CXCR4,LEPR,PROX1,THBS1	6
Cancer,Organismal Injury and Abnormalities	blue round small cell tumor	0.000175			C4A/C4B,CALCR,CXCR4,FERMT1,KCNMA1,MUC5AC,NCOA2,OVGP1,PDCD6,PDE4DIP,SLC22A18,SLC39A4,TG,THBS1,UTRN,WNT5A	16
Cardiovascular Disease,Cardiovascular System Development and Function,Cell Morphology,Organismal Development,Organismal Injury and Abnormalities,Tissue Morphology	abnormal morphology of vascular endothelial cells	0.000175			CLIC4,FLT1,SMAD5,STK4	4
Organismal Injury and Abnormalities,Tissue Morphology	abnormal morphology of epithelial tissue	0.000188			CLIC4,FLT1,MUC5AC,NCOA2,SLC26A4,SMAD5,STK4,TCF7L2,TGFB2,THBS1,USH1C,VDR,VLDLR	13

Cellular Movement	migration of eye cell lines	0.000192			FLT1,PDE4DIP,TGFB2	3
Infectious Diseases	viral entry by Retroviridae	0.000192			CXCL12,CXCR4,TFRC	3
Cellular Assembly and Organization	formation of plasma membrane	0.000194		-1.953	ADGRL3,CREB1,GABPA,HNF1B,IQGAP1,SRPX2,THBS1,TNC,UTRN,WNT5A	10
Cancer,Gastrointestinal Disease,Organismal Injury and Abnormalities	salivary gland tumor	0.000197			LEPR,PRKACB,SSBP2,TLK1,TNC	5
Cancer,Gastrointestinal Disease,Organismal Injury and Abnormalities	tumorigenesis of digestive organ tumor	0.000197			ABCC9,ADAM10,ADAMTS9,ALDH6A1,ALLC,ANGPT2,ATRX,CALCR,CEACAM1,CES2,CYP4F2,EDIL3,ELF1,ENPEP,FAM216A,FERMT1,FHL1,FILIP1L,FLT1,HNRNPL,JAG1,KCNMA1,KIF13A,LEPR,LTBP1,MAGI1,MAP4K5,MAST4,MBNL1,MBNL2,MECOM,MIA2,MLXIPL,MUC5AC,NCOA2,NEFH,NFASC,NR1D2,OVGP1,PCM1,PDE4DIP,PGGT1B,RAPGEF3,RASAL2,RIOK3,SEMA4G,SEMA6A,SENP7,SLC13A2,SLC38A2,SLC39A4,SPTLC3,SSBP2,STAG2,SYT1,TCF7L2,TES,TFRC,TG,THBS1,THSD7A,TNC,TUBAL3,UBE3B,UTRN,VDR,VLDLR,VPS13D,WNT5A,ZFR,ZFX,ZFY,ZNF480,ZNF711	74
Connective Tissue Development and Function,Tissue Morphology	quantity of adipose tissue	0.000199		0.736	CEACAM1,CPE,DLK1,DPT,ID4,LEPR,MLXIPL,NCOA2,RAPGEF3,VDR	10
Cardiovascular Disease	disorder of blood pressure	0.000205		0	ABCC9,ANGPT2,CHN2,CXCR4,EDNRA,FERMT1,FHL1,FHL5,FLT1,LEPR,MECOM,MSMO1,PTGER2,TAF13,TMX1,TNC,VDR,VLDLR	18
Cancer,Organismal Injury and Abnormalities,Reproductive System Disease	breast cancer	0.000206			ABCC9,ABO,ADAMTS9,ANGPT2,ATRX,CES2,CPE,CXCL12,CXCR4,FAM198B,FLT1,JAG1,LEPR,LYVE1,MAGI1,MAST4,MCAM,MECOM,PDCD6,PROX1,S100A4,SENP7,SLC22A18,STAG2,TCF7L2,TES,TGFB2,THBS1,TNC,UTRN,VDR,VLDLR,VPS13D,WNT5A	34
Cancer,Gastrointestinal Disease,Hepatic System Disease,Organismal Injury and Abnormalities	liver carcinoma	0.000206		1.732	ABCC9,ATRX,BAG2,CALCRL,CDC40,CEACAM1,CES2,CHN2,CPNE3,CREB1,CXCL12,CXCR4,DPT,EDIL3,EDNRA,EFS,FERMT1,FHL1,FLT1,GPX2,GRB10,ID4,IQGAP1,JAG1,KCNMA1,LEPR,LYVE1,MAST4,MDFIC,ME2,MECOM,MIA2,MLXIPL,MUC5AC,NCOA2,NUPR1,OVGP1,PAMR1,PDE4DIP,PLCL2,PROX1,PROZ,RAB23,RAPGEF3,RASAL2,RIOK3,S100A4,SEMA6A,SLC26A4,SLC38A2,SLC39A4,SRPX2,STAG2,TCF7L2,TG,TGFB2,THBS1,TLK1,TPCN1,UBE3B,USH1C,VDR,VLDLR,VPS13D,ZFR,ZNF277	66
Cancer,Gastrointestinal Disease,Organismal Injury and Abnormalities	oral cavity carcinoma	0.000208			ADAM10,CXCL12,DPT,EDIL3,ENPEP,PRKACB,TLK1,TM4SF1,TNC	9
Embryonic Development,Organ Development,Organismal Development,Reproductive System Development and Function,Tissue Development	gonadogenesis	0.000209			ATRX,CREB1,CXCL12,CXCR4,FHL5,HSF2,LEPR,NCOA2,NUPR1,PTGER2,SMAD5,TGFB2,VDR,WNT5A,ZFX	15
Cancer,Organismal Injury and Abnormalities	acinar-cell carcinoma	0.000214			ADAM10,PRKACB,TLK1,TNC	4
Cellular Development,Cellular Growth and Proliferation,Nervous System Development and Function,Tissue Development	development of neurons	0.000222		-0.751	ADAM10,ADGRL3,ATRX,CREB1,CXCL12,CXCR4,DLK1,EDNRA,GABPA,NEFH,PROX1,RAPGEF3,SEMA6A,SRPX2,TGFB2,THBS1,TNC,UTRN,VLDLR,WNT5A	20

Cancer,Gastrointestinal Disease,Organismal Injury and Abnormalities	pancreatobiliary tumor	0.000224			ABCC9,ABO,ADAM10,ATRX,CXCL12,CXCR4,CYP4F2,ENOSF1,FERMT1,FHL1,FLT1,GRB10,HNRNPL,IQGAP1,ITGB1BP1,LEPR,LTBP1,MAP4K5,MAST4,MCAM,MECOM,MIA2,MUC5AC,NEFH,NFASC,PCM1,PDE4DIP,PRKACB,RASAL2,SLC22A18,SPTLC3,STAG2,TCF7L2,TG,TGFB2,THBS1,THSD7A,TM4SF1,TNC,UBE3B,UTRN,WNT5A,ZFR,ZFX,ZNF277,ZNF480,ZNF711	47
Cell-To-Cell Signaling and Interaction,Cellular Movement,Hematological System Development and Function,Immune Cell Trafficking,Inflammatory Response	recruitment of macrophages	0.000226		-0.808	ANGPT2,CXCL12,FLT1,S100A4,THBS1,VDR	6
Cellular Development,Cellular Growth and Proliferation,Digestive System Development and Function,Hepatic System Development and Function,Organ Development	proliferation of liver cells	0.000226		-0.737	CEACAM1,CXCL12,CXCR4,EDNRA,FLT1,LEPR,PROX1,THBS1	8
Cellular Movement	release of blood cells	0.000232			CXCL12,CXCR4,TFRC	3
Organismal Injury and Abnormalities	Hypertrophy	0.000238			ANGPT2,CREB1,CXCL12,EDNRA,EMP1,FHL1,FLT1,LEPR,MBNL1,MBNL2,RAPGEF3,TG,VDR,WNT5A	14
Cell Morphology	abnormal morphology of cells	0.000238			CLIC4,CXCR4,DPT,FHL5,FLT1,MBNL1,MBNL2,MECOM,MUC5AC,NCOA2,NEFH,NFASC,PGGT1B,PTGER2,RAPGEF3,RETREG1,SLC26A4,SMAD5,STK4,TCF7L2,TFRC,TGFB2,THBS1,USH1C,UTRN,VDR,VLDLR,WNT5A	28
Cell-To-Cell Signaling and Interaction,Cellular Movement,Hematological System Development and Function,Immune Cell Trafficking	recruitment of leukocytes	0.000252		-0.191	ADAM10,ANGPT2,C4A/C4B,CXCL12,CXCR4,EDIL3,FLT1,S100A4,THBS1,TNC,VDR	11
Cardiovascular System Development and Function,Cell Morphology,Cellular Development,Organismal Development,Tissue Development	tubulation of endothelial cell lines	0.000257		-1.103	CLIC4,CXCL12,CXCR4,PATJ	4
Cancer,Organismal Injury and Abnormalities	neoplasia of tumor cells	0.000258		-1.067	CEACAM1,CREB1,CXCR4,MCAM,TM4SF1,WNT5A	6
Embryonic Development,Nervous System Development and Function,Organ Development,Organ Morphology,Organismal Development,Tissue Development,Visual System Development and Function	morphology of cornea	0.000267			DPT,JAG1,MUC5AC,TGFB2,THBS1	5
Cellular Movement	cell movement of stem cells	0.000267		-1.636	ANGPT2,CXCL12,CXCR4,FLT1,S100A4	5
Developmental Disorder,Embryonic Development,Organismal Development,Tissue Morphology	abnormal morphology of branchial arch	0.000267			ADAM10,EDNRA,LTBP1,MECOM,SMAD5	5
Cellular Assembly and Organization	development of cytoplasm	0.00027		0	CREB1,CXCL12,EDNRA,ITGB1BP1,JAG1,MAGI1,NEFH,RAB23,RAPGEF3,STK4,THBS1,TNC,TPM4,UTRN	14

Cardiovascular Disease,Organismal Injury and Abnormalities	intermediate disease stage peripheral arterial disease	0.000274			CXCL12,CXCR4,DNAJB4,MBNL2,PDE4DIP,TGFB2,THBS1	7
Gastrointestinal Disease,Hepatic System Disease,Organismal Injury and Abnormalities	formation of gallstone	0.000277			CPE,LEPR,MUC5AC	3
Protein Synthesis	expression of reporter protein	0.000277			CREB1,NCOA2,VDR	3
Endocrine System Disorders,Gastrointestinal Disease,Metabolic Disease,Organismal Injury and Abnormalities	non-insulin-dependent diabetes mellitus	0.000279			ADAMTS9,CALCR,CXCL12,EDNRA,FLT1,HNF1B,LEPR,PROX1,TAF13,TCF7L2,TNC,TSPAN5,VDR	13
Cardiovascular System Development and Function,Cell Morphology,Cellular Development,Organismal Development,Tissue Development	branching of endothelial cells	0.000284		-1.284	ANGPT2,ITGB1BP1,PROX1,SRPX2,TNC	5
Cell-mediated Immune Response,Cellular Movement,Hematological System Development and Function,Immune Cell Trafficking	cell movement of T lymphocytes	0.000291		-1.573	ADAM10,CXCL12,CXCR4,EFS,HNRNPL,MCAM,S100A4,THBS1,WNT5A	9
Cell-To-Cell Signaling and Interaction	activation of connective tissue cells	0.000293		-0.324	CXCL12,CXCR4,DLK1,LEPR,TANK,TGFB2,WNT5A	7
Developmental Disorder,Tissue Morphology	abnormal morphology of extraembryonic tissue	0.000295			ADAM10,ADAMTS9,HNF1B,JAG1,MECOM,SMAD5,STK4,ZFX	8
Cellular Movement,Immune Cell Trafficking	leukocyte migration	0.000301		-1.401	ADAM10,ANGPT2,C4A/C4B,CEACAM1,CXCL12,CXCR4,EDIL3,EDNRA,EFS,FLT1,HNRNPL,MCAM,PROX1,S100A4,STK4,TG,TGFB2,THBS1,TNC,VDR,WNT5A	21
Organismal Injury and Abnormalities	Bleeding	0.000303		0.072	ANGPT2,C4A/C4B,CXCR4,EDNRA,ENTPD1,FLT1,JAG1,MECOM,PROZ,PTGER2,SMAD5,THBS1,VDR	13
Cellular Movement	cell movement of leukemia cell lines	0.000304		-1.054	ADAM10,CXCL12,CXCR4,FLT1,IQGAP1,TNC,WNT5A	7
Organismal Injury and Abnormalities,Reproductive System Disease	disease of placenta	0.000304			CALCRL,DLK1,FLT1,GRB10,MECOM,NCOA2,STK4	7
Cardiovascular System Development and Function,Cell-To-Cell Signaling and Interaction	adhesion of endothelial progenitor cells	0.000307			CXCL12,MCAM	2

Cell-mediated Immune Response,Cellular Movement,Hematological System Development and Function,Hematopoiesis,Immune Cell Trafficking	emigration of thymocytes	0.000307			CXCL12,CXCR4	2
Amino Acid Metabolism,Molecular Transport,Small Molecule Biochemistry	transport of L-histidine	0.000307			SLC38A2,SLC38A3	2
Auditory and Vestibular System Development and Function,Auditory Disease,Organ Morphology,Organismal Development,Organismal Injury and Abnormalities	abnormal morphology of spiral limbus	0.000307			SLC26A4,TGFB2	2
Cellular Movement,Hematological System Development and Function	redistribution of megakaryocytes	0.000307			CXCR4,FLT1	2
Embryonic Development,Organismal Development,Tissue Development	formation of rostral migratory stream	0.000307			CXCR4,VLDLR	2
Cell Morphology,Hematological System Development and Function	polarization of B-lymphocyte derived cell lines	0.000307			CXCL12,STK4	2
Cellular Development,Cellular Growth and Proliferation,Nervous System Development and Function,Tissue Development	growth of thalamocortical axons	0.000307			CXCL12,CXCR4	2
Cellular Assembly and Organization,Cellular Function and Maintenance	microtubule dynamics	0.000308		-1.727	ADAM10,ANGPT2,ATRX,CALU,CREB1,CXCL12,CXCR4,EDNRA,FLT1,IQGAP1,NANOG,NEFH,PCM1,PDE4DIP,PRKACB,RAB23,RAPGEF3,S100A4,SEMA6A,SMAD5,SYT1,THBS1,TM4SF1,TNC,VLDLR,WNT5A	26
Cell-To-Cell Signaling and Interaction	binding of bone cancer cell lines	0.000327			CALCRL,CXCL12,TNC	3
Cell-mediated Immune Response,Cellular Movement,Hematological System Development and Function,Hematopoiesis,Immune Cell Trafficking	migration of thymocytes	0.000327			CXCL12,CXCR4,HNRNPL	3
Endocrine System Disorders,Gastrointestinal Disease,Organismal Injury and Abnormalities	pancreatic mass	0.000331			ABCC9,ABO,ADAM10,ATRX,CES2,CXCL12,CXCR4,CYP4F2,ENOSF1,FERMT1,FHL1,FLT1,GRB10,HNRNPL,IQGAP1,ITGB1BP1,LEPR,LTBP1,MAST4,MCAM,MECOM,MIA2,MUC5AC,NEFH,NFASC,PCM1,PDE4DIP,PRKACB,RASAL2,SLC22A18,SPTLC3,STAG2,TCF7L2,TG,TGFB2,THBS1,THSD7A,TNC,UTRN,WNT5A,ZFR,ZFX,ZNF277,ZNF480,ZNF711	45
Developmental Disorder	hypoplasia of organ	0.000334		1.689	ATRX,CALCRL,DLK1,EDNRA,HNF1B,ITGB1BP1,MECOM,NCOA2,SSBP2,TGFB2,THBS1,VDR,VLDLR	13

Cancer,Organismal Injury and Abnormalities	neoplasia of carcinoma cell lines	0.000337		-0.714	ADAMTS9,CEACAM1,CXCR4,DNAJB4,S100A4	5
Cancer,Organismal Injury and Abnormalities	cancer of exocrine gland	0.000337			ADAM10,PRKACB,SSBP2,TLK1,TNC	5
Cellular Development,Cellular Growth and Proliferation,Hair and Skin Development and Function	proliferation of epithelial cell lines	0.000354		0.923	CALCR,CREB1,ID4,NANOG,NCOA2,PTGER2,TFRC,TGFB2,WNT5A	9
Cancer,Endocrine System Disorders	hyperplasia of endocrine gland	0.000356		-0.555	RAPGEF3,TCF7L2,TG,THBS1,VDR	5
Cardiovascular Disease	Hypertension	0.000359		-0.391	ABCC9,ANGPT2,CHN2,CXCR4,EDNRA,FERMT1,FHL1,FHL5,FLT1,LEPR,MECOM,MSMO1,TAF13,TMX1,TNC,VDR,VLDLR	17
Cancer,Gastrointestinal Disease,Organismal Injury and Abnormalities	gastric carcinoma	0.000363			ABCC9,ADAM10,ADAMTS9,ANGPT2,CES2,CYP4F2,FILIP1L,HNRNPL,KCNMA1,MAST4,MBNL2,MUC5AC,NCOA2,NFASC,PDE4DIP,SEMA4G,SLC13A2,SPTLC3,SSBP2,TG,THBS1,THSD7A,TNC,UBE3B,UTRN,VLDLR,ZNF480	27
Cellular Movement	cell movement of mammary tumor cells	0.000364		-1.934	CXCL12,CXCR4,MCAM,WNT5A	4
Cancer,Gastrointestinal Disease,Organismal Injury and Abnormalities	gastroesophageal cancer	0.00037			ABCC9,ADAM10,ADAMTS9,ANGPT2,CES2,CPE,CXCL12,CYP4F2,FILIP1L,FLT1,HNRNPL,KCNMA1,MAST4,MBNL2,MUC5AC,NCOA2,NEFH,NFASC,OVGP1,PDE4DIP,SEMA4G,SLC13A2,SPTLC3,SSBP2,TG,THBS1,THSD7A,TNC,TPCN1,UBE3B,UTRN,VLDLR,WNT5A,ZNF480	34
Cellular Assembly and Organization,Cellular Function and Maintenance,Tissue Development	formation of actin stress fibers	0.000371		0.308	CREB1,CXCL12,EDNRA,ITGB1BP1,JAG1,MAGI1,RAPGEF3,TNC,TPM4	9
Cell Signaling	activation of enzyme	0.000373			CEACAM1,CXCR4,EDNRA,IQGAP1,ITGB1BP1,MAP4K5,MDFIC,PDCD6,THBS1,VLDLR,WNT5A	11
Hematological System Development and Function,Tissue Morphology	quantity of blood cells	0.000377		-1.54	ADAM10,C4A/C4B,CALCRL,CLIC4,CREB1,CXCL12,CXCR4,DLK1,ELF1,FLT1,LEPR,MECOM,PLCL2,PTGER2,SMAD5,SSBP2,STK4,TANK,TFRC,TG,THBS1,VDR,ZFX	23
Cellular Movement	invasion of lung cancer cell lines	0.000379		-0.647	ADAM10,CXCL12,CXCR4,DNAJB4,S100A4,TGFB2	6
Cell Morphology,Cellular Development	branching of cells	0.000379		-1.562	ADAM10,ANGPT2,ATRX,CREB1,CXCL12,ITGB1BP1,NEFH,PROX1,SEMA6A,SRPX2,TNC,WNT5A	12
Cell-To-Cell Signaling and Interaction,Hematological System Development and Function	binding of mononuclear leukocytes	0.000381		-1.558	ANGPT2,CXCL12,CXCR4,MCAM,STK4,TFRC,THBS1,TNC	8
Cardiovascular System Development and Function,Embryonic Development,Organismal Development,Tissue Development	vascularization of extraembryonic tissue	0.000383			CEACAM1,SMAD5,STK4	3

Cellular Development	differentiation of vasculature	0.000383			CXCL12,CXCR4,JAG1	3
Cardiovascular System Development and Function,Organismal Development	remodeling of vasculature	0.000383			CXCL12,CXCR4,FLT1	3
Cancer,Organismal Injury and Abnormalities,Reproductive System Disease	breast or ovarian cancer	0.000392			ABCC9,ABO,ADAM10,ADAMTS9,ANGPT2,ATRX,C4A/C4B,CES2,CLIC4,CPE,CXCL12,CXCR4,ENPEP,FAM198B,FLT1,JAG1,LEPR,LYVE1,MAGI1,MAST4,MCAM,MECOM,MUC5AC,NCOA2,OVGP1,PDCD6,PROX1,PTGER2,S100A4,SENP7,SLC22A18,SLC39A4,STAG2,TCF7L2,TES,TG,TGFB2,THBS1,TM4SF1,TNC,TPM4,TUBAL3,UTRN,VDR,VLDLR,VPS13D,WNT5A	47
Cardiovascular System Development and Function,Cellular Movement	cell movement of endothelial cell lines	0.000395		-0.447	ANGPT2,FLT1,LYVE1,PATJ,THBS1,TNC	6
Cell-To-Cell Signaling and Interaction	activation of cells	0.000398		-0.669	ADAM10,ANGPT2,C4A/C4B,CEACAM1,CPE,CXCL12,CXCR4,DLK1,ENPEP,ENTPD1,IQGAP1,KCNMA1,LEPR,LTBP1,PTGER2,RAPGEF3,TANK,TG,TGFB2,THBS1,TNC,WNT5A	22
Cardiovascular Disease,Cardiovascular System Development and Function,Organ Morphology,Organismal Development,Organismal Injury and Abnormalities	hypertrophy of heart	0.000402			ANGPT2,CREB1,CXCL12,EDNRA,FLT1,LEPR,MBNL1,MBNL2,RAPGEF3,TG,VDR	11
Cancer,Gastrointestinal Disease,Organismal Injury and Abnormalities	gastrointestinal adenocarcinoma	0.000405			ABCC9,ADAMTS9,ALDH6A1,ALLC,ANGPT2,ATRX,CALCR,CES2,CPE,CYP4F2,EDIL3,ELF1,ENPEP,FAM216A,FHL1,FILIP1L,FLT1,HNRNPL,JAG1,KCNMA1,KIF13A,LEPR,LTBP1,MAGI1,MAP4K5,MAST4,MBNL1,MBNL2,MECOM,MIA2,MLXIPL,MUC5AC,NCOA2,NEFH,NFASC,NR1D2,OVGP1,PCM1,PDE4DIP,PGGT1B,PRKACB,RAPGEF3,RASAL2,RIOK3,SEMA4G,SEMA6A,SENP7,SLC13A2,SLC38A2,SLC39A4,SPTLC3,SSBP2,SYT1,TCF7L2,TES,TFRC,TG,THBS1,THSD7A,TLK1,TNC,TPCN1,TUBAL3,UBE3B,UTRN,VDR,VPS13D,WNT5A,ZFR,ZFX,ZFY,ZNF480,ZNF711	73
Cancer,Gastrointestinal Disease,Organismal Injury and Abnormalities	gastro-esophageal carcinoma	0.000416			ABCC9,ADAM10,ADAMTS9,ANGPT2,CES2,CPE,CYP4F2,FILIP1L,FLT1,HNRNPL,KCNMA1,MAST4,MBNL2,MUC5AC,NCOA2,NEFH,NFASC,OVGP1,PDE4DIP,SEMA4G,SLC13A2,SPTLC3,SSBP2,TG,THBS1,THSD7A,TNC,TPCN1,UBE3B,UTRN,VLDLR,ZNF480	32
Cardiovascular System Development and Function,Tissue Morphology	permeability of vasculature	0.000428		-0.328	ANGPT2,C4A/C4B,CREB1,CXCL12,FLT1,THBS1	6
Cancer,Organismal Injury and Abnormalities	low grade acinar-cell carcinoma	0.000444			PRKACB,TLK1,TNC	3
Cellular Movement	migration of neuroblastoma cell lines	0.000444			ANGPT2,CXCL12,CXCR4	3
Organ Morphology,Reproductive System Development and Function	morphology of genital organ	0.000456			ATRX,FHL5,FLT1,MLXIPL,NCOA2,NUPR1,PTGER2,RAPGEF3,SMAD5,TGFB2,THBS1,VDR,VLDLR,WNT5A	14
Cell Death and Survival	apoptosis of breast cancer cell lines	0.000456		0.101	ANGPT2,CEACAM1,GPX2,NANOG,PDCD4,SPAG5,TFRC,TGFB2,THBS1,VDR	10

Cell Death and Survival,Organismal Injury and Abnormalities	cell death of endothelial cells	0.00046		-0.063	ANGPT2,CXCL12,CXCR4,EDIL3,FLT1,MCAM,SEMA6A,THBS1	8
Cardiovascular System Development and Function,Organismal Development	development of aorta	0.000462			CXCR4,FLT1,LTBP1,TGFB2	4
Cancer,Gastrointestinal Disease,Hepatic System Disease,Organismal Injury and Abnormalities	biliary tract adenocarcinoma	0.000465			ATRX,CXCR4,FLT1,LEPR,MAP4K5,TCF7L2,TM4SF1	7
Cell-To-Cell Signaling and Interaction,Cellular Growth and Proliferation	stimulation of cells	0.000466		-1.373	ANGPT2,CALCRL,CXCL12,HNF1B,PTGER2,S100A4,TG,TGFB2,THBS1,WNT5A	10
Cellular Movement,Hematological System Development and Function,Immune Cell Trafficking,Inflammatory Response	cell movement of phagocytes	0.000476		-1.887	ADAM10,ANGPT2,C4A/C4B,CEACAM1,CXCL12,CXCR4,EDIL3,EDNRA,FLT1,MCAM,S100A4,STK4,TGFB2,THBS1,WNT5A	15
Organismal Injury and Abnormalities,Renal and Urological Disease	urination disorder	0.000485		-0.6	ANGPT2,FERMT1,FLT1,KCNMA1,LEPR,SLC13A2,SLC26A4,TGFB2,VDR,VLDLR	10
Cellular Function and Maintenance,Cellular Movement,Hematological System Development and Function,Infectious Diseases	release of neutrophils	0.000509			CXCL12,CXCR4	2
Cellular Movement,Embryonic Development	homing of mesenchymal stem cells	0.000509			CXCR4,S100A4	2
Embryonic Development,Endocrine System Development and Function,Organ Development,Organismal Development,Tissue Development	formation of thyroid follicle	0.000509			CREB1,THBS1	2
Cancer,Gastrointestinal Disease,Organismal Injury and Abnormalities	gastrointestinal carcinoma	0.00051			ABCC9,ADAM10,ADAMTS9,ALDH6A1,ALLC,ANGPT2,ATRX,CALCR,CALCRL,CALU,CDC40,CEACAM1,CES2,CPE,CREB1,CXCL12,CXCR4,CYP4F2,DLK1,DNAJB4,DPT,EDIL3,ELF1,ENOSF1,ENPEP,FAM216A,FERMT1,FHL1,FHL5,FILIP1L,FLT1,GGH,GRB10,HNRNPL,IQGAP1,JAG1,KCNMA1,KIF13A,LEPR,LHPP,LTBP1,MAGI1,MAP4K5,MAST4,MBNL1,MBNL2,MCAM,MECOM,MIA2,MLXIPL,MUC5AC,NCOA2,NEFH,NFASC,NR1D2,OVGP1,PAMR1,PCM1,PDE4DIP,PGGT1B,PLCL2,PRKACB,PROX1,RAPGEF3,RASAL2,RIOK3,SEMA4G,SEMA6A,SENP7,SLC13A2,SLC26A4,SLC38A2,SLC39A4,SLCO2A1,SPAG5,SPTLC3,SRPX2,SSBP2,STAG2,SYT1,TAF13,TCF7L2,TES,TFRC,TG,TGFB2,THBS1,THSD7A,TLK1,TM4SF1,TMX1,TNC,TPCN1,TSPAN5,TUBAL3,UBE3B,UTRN,VDR,VLDLR,VPS13D,WNT5A,ZFR,ZFX,ZFY,ZNF480,ZNF711	106
Cellular Growth and Proliferation	colony formation of cells	0.000511		0.107	CEACAM1,CXCL12,FLT1,ID4,IQGAP1,MCAM,MECOM,NANOG,NUPR1,SMAD5,STK4,TCF7L2,TES,TGFB2	14
Cancer,Organismal Injury and Abnormalities	growth of secondary tumor	0.000511			CXCR4,TGFB2,TNC	3

Table G: Gene Functions Data Shown in Figure 6

Cardiovascular Disease,Cardiovascular System Development and Function,Organismal Injury and Abnormalities,Tissue Morphology	abnormal morphology of blood vessel	0.000526			CALCRL,CXCL12,CXCR4,EDNRA,FLT1,LTBP1,SMAD5,STK4,TGFB2	9
Organismal Development,Reproductive System Development and Function	development of reproductive system	0.000545			ATRX,CREB1,CXCL12,CXCR4,FHL5,HNF1B,HSF2,ID4,LEPR,NCOA2,NUPR1,PTGER2,SMAD5,STK4,TGFB2,VDR,WNT5A,ZFX	18
Cell Morphology,Cellular Assembly and Organization,Cellular Function and Maintenance	formation of cellular protrusions	0.000561		-1.365	ADAM10,ATRX,CALU,CREB1,CXCL12,CXCR4,EDNRA,IQGAP1,NANOG,NEFH,PCM1,PRKACB,RAB23,RAPGEF3,S100A4,SEMA6A,THBS1,TM4SF1,TNC,VLDLR,WNT5A	21
Hereditary Disorder,Organismal Injury and Abnormalities	autosomal dominant disease	0.000568			ABCC9,ADAM10,ADGRL3,ANGPT2,CPE,CXCR4,EDNRA,FHL1,FILIP1L,HNF1B,JAG1,MBNL1,MECOM,MLXIPL,NEFH,TGFB2,TNC,TPM4,VLDLR,WNT5A	20
Skeletal and Muscular Disorders	damage of muscle	0.000569		0.447	ANGPT2,FHL1,MBNL1,MBNL2,PDCD4	5
Cell-To-Cell Signaling and Interaction,Hematological System Development and Function	binding of lymphocytes	0.000577		-1.259	CXCL12,CXCR4,MCAM,STK4,TFRC,THBS1,TNC	7
Cell-To-Cell Signaling and Interaction,Connective Tissue Development and Function,Hepatic System Development and Function	activation of hepatic stellate cells	0.000578			CXCL12,DLK1,LEPR,WNT5A	4
Nutritional Disease	obesity	0.00058		0.356	ANGPT2,CEACAM1,CPE,CREB1,CXCR4,DLK1,ENTPD1,FLT1,LEPR,NCOA2,PLCL2,PROX1,RAPGEF3,VDR	14
Cellular Development,Nervous System Development and Function,Tissue Development	differentiation of hair cells	0.000585			JAG1,KCNMA1,USH1C	3
Cellular Movement	migration of neuronal progenitor cells	0.000585			ANGPT2,CXCL12,CXCR4	3
Skeletal and Muscular System Development and Function	morphology of muscle	0.000588			ANGPT2,CALCRL,CXCL12,CXCR4,EDNRA,FHL1,GRB10,LTBP1,MBNL1,RAPGEF3,TGFB2,THBS1,UTRN,VDR	14
Organismal Injury and Abnormalities	Fibrosis	0.000589		0.092	ANGPT2,CREB1,CXCL12,DLK1,EDNRA,ENTPD1,FLT1,MBNL1,MBNL2,S100A4,STK4,TGFB2,THBS1,TM4SF1,VDR	15
Cardiovascular System Development and Function	neovascularization	0.000595		-0.058	CLIC4,CXCL12,CXCR4,FLT1,S100A4,THBS1,VLDLR	7
Digestive System Development and Function,Gastrointestinal Disease	abnormal morphology of digestive system	0.000596			C4A/C4B,CEACAM1,DLK1,EDNRA,FERMT1,IQGAP1,JAG1,MUC5AC,RAPGEF3,TCF7L2,TGFB2,THBS1,USH1C,VDR,WNT5A	15
Gastrointestinal Disease,Hepatic System Disease,Organismal Injury and Abnormalities	cholelithiasis	0.00062			CPE,LEPR,MUC5AC,TNC	4
Cancer,Organismal Injury and Abnormalities,Tumor Morphology	progression of tumor	0.000628		-0.563	CXCL12,CXCR4,FLT1,MCAM,PDCD4,S100A4,THBS1,TNC,WNT5A	9

Cancer,Endocrine System Disorders,Gastrointestinal Disease,Organismal Injury and Abnormalities	pancreatic tumor	0.000634			ABCC9,ABO,ADAM10,ATRX,CXCL12,CXCR4,CYP4F2,ENOSF1,FERMT1,FHL1,FLT1,GRB10,HNRNPL,IQGAP1,ITGB1BP1,LEPR,LTBP1,MAST4,MCAM,MECOM,MIA2,MUC5AC,NEFH,NFASC,PCM1,PDE4DIP,PRKACB,RASAL2,SLC22A18,SPTLC3,STAG2,TCF7L2,TG,TGFB2,THBS1,THSD7A,TNC,UTRN,WNT5A,ZFR,ZFX,ZNF277,ZNF480,ZNF711	44
Cancer,Organismal Injury and Abnormalities	connective tissue tumor	0.000635		1.121	ANGPT2,ATRX,CALCR,CREB1,CXCL12,FERMT1,FLT1,LEPR,MUC5AC,NCOA2,OVGP1,PDE4DIP,S100A4,SLC22A18,SLC39A4,SSBP2,STAG2,TG,THBS1,VDR	20
Cellular Assembly and Organization,Cellular Function and Maintenance,Tissue Development	formation of actin filaments	0.000665		-0.095	CREB1,CXCL12,EDNRA,ITGB1BP1,JAG1,MAGI1,RAPGEF3,STK4,TNC,TPM4	10
Organismal Development,Organismal Injury and Abnormalities	abnormal morphology of thoracic cavity	0.00067			ADAM10,ADAMTS9,CALCRL,CREB1,CXCR4,EDNRA,JAG1,LTBP1,MBNL1,MBNL2,MECOM,SLCO2A1,TGFB2,THBS1,WNT5A	15
Cancer,Gastrointestinal Disease,Organismal Injury and Abnormalities	secondary neoplasm of digestive system	0.000678			CXCL12,CXCR4,EFS,EMP1,FLT1,JAG1,S100A4,TGFB2,THBS1,TM4SF1	10
Cancer,Gastrointestinal Disease,Organismal Injury and Abnormalities	colorectal carcinoma	0.000682			ADAMTS9,ALDH6A1,ALLC,ANGPT2,ATRX,CALCR,CEACAM1,CES2,CYP4F2,EDIL3,ELF1,ENPEP,FAM216A,FERMT1,FHL1,FLT1,HNRNPL,JAG1,KCNMA1,KIF13A,LEPR,LTBP1,MAGI1,MAP4K5,MAST4,MBNL1,MECOM,MIA2,MLXIPL,MUC5AC,NCOA2,NEFH,NFASC,NR1D2,OVGP1,PCM1,PDE4DIP,PGGT1B,RAPGEF3,RASAL2,RIOK3,SEMA4G,SEMA6A,SENP7,SLC13A2,SLC38A2,SLC39A4,SSBP2,STAG2,SYT1,TCF7L2,TES,TFRC,TG,THBS1,TNC,TUBAL3,UTRN,VDR,VPS13D,WNT5A,ZFR,ZFX,ZFY,ZNF480,ZNF711	66
Cellular Development,Nervous System Development and Function,Tissue Development	differentiation of neurons	0.000699		-1.342	CREB1,CXCL12,ID4,JAG1,KCNMA1,NEFH,PROX1,S100A4,TGFB2,TNC,USH1C,WNT5A	12
Nervous System Development and Function,Tissue Morphology	quantity of neurons	0.00071		-1.73	ADAM10,CREB1,CXCL12,CXCR4,HSF2,JAG1,NEFH,NFASC,PTGER2,TGFB2,UTRN,WNT5A	12
Organ Morphology,Organismal Development,Reproductive System Development and Function,Tissue Morphology	quantity of sperm	0.000712		-1	ENTPD1,FHL5,VDR,ZFX	4
Cancer,Organismal Injury and Abnormalities	neoplasia of cancer cells	0.000712			CEACAM1,CXCR4,MCAM,WNT5A	4
Cellular Function and Maintenance	function of blood cells	0.000713			CEACAM1,CREB1,CXCL12,EFS,ENTPD1,FLT1,MECOM,PTGER2,S100A4,STK4,TANK,TGFB2,TPCN1,VDR	14
Cellular Movement	cell movement of lung cancer cell lines	0.000723		-0.239	ADAM10,CXCL12,DNAJB4,S100A4,TCF7L2,TGFB2	6
Organismal Injury and Abnormalities,Renal and Urological Disease	proteinuria	0.000731		-0.277	ANGPT2,FERMT1,FLT1,LEPR,TGFB2,VDR,VLDLR	7
Cancer,Organismal Injury and Abnormalities,Reproductive System Disease	uterine serous papillary cancer	0.000745			CXCL12,DPT,EDNRA,ENPEP,ENTPD1,FGL2,FILIP1L,KCNMA1	8

Organismal Injury and Abnormalities,Renal and Urological Disease	renal lesion	0.000746			ADAMTS9,ATRX,CALCRL,CES2,CYP4F3,ENPEP,ENTPD1,FLT1,HNF1B,KCNMA1,MAGI1,MAST4,MECOM,MLXIPL,MUC5AC,PDE4DIP,PROX1,SLC26A4,STAG2,SULT1C2,THBS1,THSD7A,TSPAN5,UTRN	24
Cellular Movement	migration of glioma cells	0.000752			CXCL12,TGFB2,THBS1	3
Cardiovascular Disease,Cardiovascular System Development and Function,Organ Morphology,Organismal Development,Organismal Injury and Abnormalities	abnormal morphology of dilated atrium	0.000752			EDNRA,MBNL1,MBNL2	3
Organ Morphology,Reproductive System Development and Function	quantity of ovarian follicle	0.000756		-1.732	FLT1,HSF2,LEPR,PTGER2,ZFX	5
Cellular Movement	release of hematopoietic progenitor cells	0.00076			CXCL12,CXCR4	2
Nervous System Development and Function,Neurological Disease,Organ Morphology,Organismal Development,Organismal Injury and Abnormalities	abnormal morphology of external granule cell layer	0.00076			CXCL12,CXCR4	2
Cancer,Dermatological Diseases and Conditions,Organismal Injury and Abnormalities	neoplasia of melanoma cells	0.00076			MCAM,WNT5A	2
Amino Acid Metabolism,Molecular Transport,Small Molecule Biochemistry	efflux of glutamine family amino acid	0.00076			SLC38A2,SLC38A3	2
Cellular Movement,Hematological System Development and Function,Hematopoiesis,Immune Cell Trafficking,Inflammatory Response	migration of plasmacytoid precursor dendritic cells	0.00076			CXCL12,CXCR4	2
Amino Acid Metabolism,Molecular Transport,Small Molecule Biochemistry	transport of L-alanine	0.00076			SLC38A2,SLC38A3	2
Cellular Movement	release of blood platelets	0.00076			CXCL12,TFRC	2
Developmental Disorder,Ophthalmic Disease,Organismal Injury and Abnormalities	blepharophimosis	0.00076			DLK1,UBE3B	2
Lipid Metabolism,Small Molecule Biochemistry	fatty acid metabolism	0.00076		0.066	CEACAM1,CXCL12,CYP4F2,CYP4F3,EDNRA,ENTPD1,LEPR,ME2,MLXIPL,MSMO1,PTGER2,SLC38A2,SLCO2A1,VLDLR,WNT5A	15

Cell-To-Cell Signaling and Interaction,Cellular Assembly and Organization,Cellular Development,Cellular Function and Maintenance,Cellular Growth and Proliferation,Nervous System Development and Function,Tissue Development	developmental process of synapse	0.000763		-1.71	ADGRL3,CREB1,GABPA,SRPX2,THBS1,TNC,UTRN,WNT5A	8
Cellular Development,Connective Tissue Development and Function	differentiation of fibroblast cell lines	0.000773		-1.095	CREB1,DLK1,ID4,JAG1,TGFB2,VDR,WNT5A	7
Cancer,Organismal Injury and Abnormalities	embryonal tumor	0.000774			ATRX,C4A/C4B,CXCR4,FLT1,MUC5AC,NANOG,STAG2,UTRN,WNT5A,ZFX	10
Cellular Assembly and Organization,Cellular Function and Maintenance	organization of cytoskeleton	0.000778		-1.803	ADAM10,ANGPT2,ATRX,CALU,CREB1,CXCL12,CXCR4,EDNRA,FLT1,IQGAP1,NANOG,NEFH,PCM1,PDE4DIP,PRKACB,RAB23,RAPGEF3,S100A4,SEMA6A,SMAD5,SPAG5,SYT1,TGFB2,THBS1,TM4SF1,TNC,VLDLR,WNT5A	28
Organismal Injury and Abnormalities	Edema	0.000791		1.415	ANGPT2,CALCRL,CREB1,CXCR4,EDNRA,ENTPD1,FLT1,GPX2,MECOM,SMAD5,TFRC	11
Cancer,Organismal Injury and Abnormalities,Renal and Urological Disease	Renal Cancer and Tumors	0.000795			ADAMTS9,ATRX,CALCRL,CES2,CYP4F3,ENPEP,FLT1,HNF1B,KCNMA1,MAGI1,MAST4,MECOM,MLXIPL,MUC5AC,PDE4DIP,PROX1,SLC26A4,STAG2,SULT1C2,THBS1,THSD7A,TSPAN5,UTRN	23
Cellular Movement,Hematological System Development and Function,Immune Cell Trafficking	cell movement of mononuclear leukocytes	0.000795		-1.502	ADAM10,ANGPT2,CEACAM1,CXCL12,CXCR4,EFS,FLT1,HNRNPL,MCAM,S100A4,TG,THBS1,WNT5A	13
Cellular Compromise	dysfunction of cells	0.000814		-0.254	ANGPT2,CXCL12,CXCR4,NEFH	4
Cancer,Organismal Injury and Abnormalities,Renal and Urological Disease	kidney adenocarcinoma formation	0.000814			ATRX,CALCRL,CES2,CYP4F3,ENPEP,FLT1,HNF1B,KCNMA1,MAGI1,MAST4,MECOM,MLXIPL,MUC5AC,PDE4DIP,PROX1,SLC26A4,STAG2,SULT1C2,THBS1,THSD7A,TSPAN5	21
Cellular Development,Cellular Growth and Proliferation	cell proliferation of breast cancer cell lines	0.000818		-1.15	CEACAM1,CHN2,CREB1,CXCL12,CXCR4,NANOG,PTGER2,S100A4,TES,TNC,UTRN,VDR,WNT5A	13
Gastrointestinal Disease,Hepatic System Disease,Inflammatory Disease,Organismal Injury and Abnormalities	primary biliary cirrhosis	0.000828			CXCR4,DLK1,JAG1,TGFB2,WNT5A	5
Cell-To-Cell Signaling and Interaction,Hematological System Development and Function	binding of B-lymphocyte derived cell lines	0.000845			CXCL12,STK4,TNC	3

Cellular Development,Tissue Development	epithelial-mesenchymal transition of epithelial cells	0.000845			CLIC4,PDCD4,S100A4	3
Cellular Development,Tissue Development	differentiation of epithelial tissue	0.000845		1	ADAMTS9,ANGPT2,CEACAM1,CLIC4,CXCR4,FLT1,HNF1B,JAG1,PROX1,STK4,VDR	11
Cardiovascular Disease,Organismal Injury and Abnormalities	peripheral arterial disease	0.00088			CXCL12,CXCR4,DNAJB4,MBNL2,PDE4DIP,PTGER2,TGFB2,THBS1	8
Developmental Disorder	dysgenesis	0.000882		1.792	ATRX,CALCRL,DLK1,EDNRA,HNF1B,ITGB1BP1,MECOM,NCOA2,SSBP2,TGFB2,THBS1,VDR,VLDLR,WNT5A	14
Cell-To-Cell Signaling and Interaction,Hematological System Development and Function	binding of T lymphocytes	0.000889		-1.607	CXCL12,CXCR4,MCAM,STK4,THBS1,TNC	6
Cardiovascular Disease,Cardiovascular System Development and Function,Organ Morphology,Organismal Development,Organismal Injury and Abnormalities	ventricular hypertrophy	0.000889			ANGPT2,CXCL12,MBNL1,MBNL2,RAPGEF3,VDR	6
Cellular Growth and Proliferation	outgrowth of cells	0.000895		-1.692	ADAM10,CREB1,CXCL12,FLT1,IQGAP1,NEFH,S100A4,SEMA6A,THBS1,TNC,VLDLR,WNT5A	12
Cellular Movement,Immune Cell Trafficking	cell movement of lymphatic system cells	0.000908		-1.443	ADAM10,CEACAM1,CXCL12,CXCR4,EFS,HNRNPL,MCAM,PROX1,S100A4,TG,THBS1,WNT5A	12
Cancer,Organismal Injury and Abnormalities	metastasis of cells	0.000917		-1.803	ANGPT2,CREB1,CXCR4,MCAM,S100A4,TCF7L2,THBS1,TM4SF1,WNT5A	9
Cardiovascular System Development and Function,Organ Development	heart rate	0.000935		-0.239	ANGPT2,CXCR4,EDNRA,FLT1,SMAD5,TG,TGFB2,THBS1,VDR	9
Cellular Movement,Embryonic Development	movement of mesenchymal stem cells	0.000946			CXCL12,CXCR4,S100A4	3
Cellular Movement	dissemination of tumor cell lines	0.000946			CXCL12,CXCR4,SENP7	3
Tissue Morphology	quantity of cervical cancer cell lines	0.000946			KIF13A,MLXIPL,PDCD6	3

Cancer,Gastrointestinal Disease,Organismal Injury and Abnormalities	high grade salivary duct carcinoma	0.000946			PRKACB,TLK1,TNC	3
Cardiovascular System Development and Function,Organismal Development,Tissue Morphology	density of microvessel	0.000946			FLT1,JAG1,PTGER2	3
Cancer,Gastrointestinal Disease,Organismal Injury and Abnormalities	colon carcinoma	0.00095			ADAMTS9,ALDH6A1,ALLC,ANGPT2,ATRX,CALCR,CES2,CYP4F2,EDIL3,ELF1,ENPEP,FAM216A,FERMT1,FHL1,FLT1,HNRNPL,JAG1,KCNMA1,KIF13A,LEPR,LTBP1,MAGI1,MAP4K5,MAST4,MBNL1,MECOM,MIA2,MLXIPL,MUC5AC,NCOA2,NEFH,NFASC,NR1D2,OVGP1,PCM1,PDE4DIP,PGGT1B,RAPGEF3,RASAL2,RIOK3,SEMA4G,SEMA6A,SENP7,SLC13A2,SLC38A2,SLC39A4,SSBP2,SYT1,TCF7L2,TES,TFRC,TG,THBS1,TNC,TUBAL3,UTRN,VPS13D,WNT5A,ZFR,ZFX,ZFY,ZNF480,ZNF711	63
Organismal Development	development of endocrine gland	0.000951			CREB1,HNF1B,TCF7L2,THBS1,VDR,WNT5A	6
Cancer,Organismal Injury and Abnormalities	malignant neoplasm of aerodigestive tract	0.000962			ABCC9,ADAM10,CALCR,CES2,CPE,CXCL12,CXCR4,DPT,EDIL3,ENPEP,FHL5,FLT1,NEFH,OVGP1,PCM1,PRKACB,PROX1,SSBP2,TG,THSD7A,TLK1,TM4SF1,TNC,TPCN1,UTRN	25
Cardiovascular System Development and Function,Organismal Development	development of artery	0.000983			CREB1,CXCR4,EMP1,FLT1,LTBP1,TGFB2	6
Connective Tissue Development and Function,Organ Morphology,Organismal Development,Reproductive System Development and Function,Tissue Morphology	mass of epididymal fat	0.000984			MLXIPL,RAPGEF3,VDR,VLDLR	4
Cancer,Hematological Disease,Immunological Disease,Organismal Injury and Abnormalities	lymphoma	0.000985		1.457	ABCC9,ADAMTS9,ANGPT2,BAG2,CXCL12,CXCR4,DPT,FERMT1,FILIP1L,HIST1H2BK,HNF1B,IQGAP1,MAP4K5,MCAM,MIA2,MLXIPL,NCOA2,PDCD4,RSPH14,S100A4,SSBP2,TGFB2,THBS1	23
Cardiovascular System Development and Function,Organ Morphology,Organismal Development	enlargement of left ventricle	0.000986			ANGPT2,MBNL1,MBNL2,STK4,VDR	5
Cellular Development,Connective Tissue Development and Function,Tissue Development	differentiation of osteoblastic-lineage cells	0.000994		-1.455	DLK1,EDNRA,ID4,JAG1,PLCL2,SMAD5,TGFB2,TNC,TPM4	9
Inflammatory Response	inflammation of absolute anatomical region	0.000998		0.051	ABHD6,ANGPT2,C4A/C4B,CEACAM1,CREB1,CXCL12,EDNRA,EFS,ELF1,ENTPD1,FERMT1,FLT1,GPX2,HSF2,JAG1,PDCD4,PTGER2,RAPGEF3,S100A4,TAF13,TANK,TFRC,THBS1,VDR	24
Cell-To-Cell Signaling and Interaction,Cellular Movement	recruitment of myeloid cells	0.00101		-1.179	ADAM10,ANGPT2,CXCL12,CXCR4,EDIL3,FLT1,S100A4,THBS1,VDR	9

Cell-To-Cell Signaling and Interaction,Cellular Assembly and Organization	formation of intercellular junctions	0.00101		-1.697	ADGRL3,CREB1,GABPA,NFASC,SRPX2,THBS1,TNC,UTRN,WNT5A	9
Cell Death and Survival	cell viability of connective tissue cells	0.00102		0.314	CALCR,CXCL12,FLT1,LEPR,PDCD4,TANK	6
Hematological System Development and Function,Organismal Functions	coagulation of blood	0.00102			C4A/C4B,CALU,CXCL12,CYP4F2,ENTPD1,PROZ,THBS1	7
Cancer,Gastrointestinal Disease,Organismal Injury and Abnormalities	salivary gland cancer	0.00105			PRKACB,SSBP2,TLK1,TNC	4
Cancer,Hematological Disease,Organismal Injury and Abnormalities	B-cell malignant tumor	0.00105			ABCC9,CXCL12,CXCR4,DPT,FERMT1,FILIP1L,HIST1H2BK,HNF1B,IQGAP1,MAP4K5,MLXIPL,NCOA2,PDCD4,SSBP2,TFRC,TGFB2	16
Carbohydrate Metabolism,Energy Production	metabolism of pyruvic acid	0.00105			ME2,MLXIPL,VLDLR	3

Table H: Upstream Regulators shown in Figure 7

Upstream Regulator	Expr Fold Change	Molecule Type	Predicted Activation State	Activation z-score	p-value of overlap	Target molecules in dataset
CG		complex	Inhibited	-3.283	0.000059	CXCR4,DPT,EMP1,FAM198B,FGL2,FHL1,LEPR,MCAM,PTGER2,TGFB2,TM4SF1
OSM		cytokine	Inhibited	-2.855	0.000465	ANGPT2,CXCL12,CYP4F3,JAG1,PDCD4,PGGT1B,SMAD5,STK4,TM4SF1,TNC,VDR,WNT5A
EDN1		cytokine	Inhibited	-2.595	1.66E-06	BAG2,CALCRL,CXCR4,EDNRA,LEPR,MCAM,PTGER2,THBS1,TNC,TPM4
Vegf		group	Inhibited	-2.395	7.02E-07	ADAM10,ANGPT2,BAG2,CALCRL,CXCL12,CXCR4,EMP1,ENTPD1,FAM198B,FLT1,GRB10,LYVE1,MAGI1,NCOA2,SMAD5,TNC
TGFB1		growth factor	Inhibited	-2.325	2.02E-08	ADAM10,CALCR,CEACAM1,CLIC4,CXCL12,CXCR4,DNAJB4,EDNRA,FERMT1,FHL1,FILIP1L,FLT1,ID4,JAG1,LTBP1,LYVE1,MBNL2,MIA2,MLXIPL,MSMO1,MUC5AC,NUPR1,PDCD4,PTGER2,RAPGEF3,S100A4,SLC22A18,TFRC,TG,TGFB2,THBS1,TNC,USH1C,VDR,WNT5A
ESR1		ligand-dependent nuclear receptor	Inhibited	-2.166	5.34E-07	CPE,CREB1,CXCL12,FHL1,IQGAP1,ITGB1BP1,KCNMA1,KIF13A,LEPR,LTBP1,MAGI1,MAP4K5,MUC5AC,NR1D2,PCM1,PDCD4,PRKACB,SLC38A2,SLC39A4,SMAD5,STAG2,STK4,SULT1C2,TCF7L2,TFRC,TGFB2,TM4SF1
E. coli B5 lipopolysaccharide		chemical - endogenous non-mammalian	Inhibited	-2	0.118	CXCR4,MLXIPL,TGFB2,THBS1
mir-223		microRNA	Inhibited	-2	0.0141	EMP1,FAM198B,FGL2,MSMO1
NFKBIA		transcription regulator	Inhibited	-2	0.0948	CXCL12,CXCR4,MUC5AC,TFRC,TGFB2,TNC
TGFBR1		kinase		-1.982	0.00241	ID4,JAG1,LTBP1,NANOG
Akt		group		-1.976	0.000744	CXCL12,CXCR4,MCAM,PTGER2,SYT1,THBS1,TNC,UTRN
IL6		cytokine		-1.919	0.0369	CALCR,CEACAM1,CES2,CXCR4,MIA2,TFRC,THBS1,TNC,VLDLR,WNT5A
STAT3		transcription regulator		-1.912	0.00218	ANGPT2,CEACAM1,CREB1,CXCL12,CXCR4,FGL2,FLT1,JAG1,LEPR,THBS1,WNT5A
bleomycin		chemical drug		-1.783	4.13E-05	ANGPT2,CXCR4,LTBP1,PTGER2,S100A4,TGFB2,THBS1,WNT5A
AR		ligand-dependent nuclear receptor		-1.664	0.0048	C4A/C4B,CALU,CXCL12,EDNRA,MSMO1,NUPR1,OVGP1,TGFB2,THBS1
prostaglandin E2		chemical - endogenous mammalian		-1.661	0.000352	ANGPT2,CALCR,CXCL12,CXCR4,ENTPD1,FLT1,MUC5AC,PTGER2,THBS1
tamoxifen		chemical drug		-1.446	0.00336	CPE,CXCL12,CXCR4,DLK1,MUC5AC,PTGER2,VDR
metribolone		chemical reagent		-1.446	0.0176	CXCR4,NCOA2,PRKACB,THBS1,TM4SF1
HGF		growth factor		-1.405	5.38E-07	ANGPT2,BAG2,CALCR,CALCRL,CXCR4,EMP1,ENTPD1,FLT1,GRB10,LYVE1,NANOG,NCOA2,SMAD5,TGFB2,THBS1,VDR
MYC		transcription regulator		-1.343	0.00216	ANGPT2,CLIC4,EIF2S1,EMP1,GGH,ME2,MUC5AC,NANOG,PDCD4,PRKACB,TES,TFRC,TGFB2,THBS1,TNC,VLDLR,WNT5A
Salmonella enterica serotype abortus equi lipopolysaccharide		chemical toxicant		-1.342	0.00773	DNAJB4,FLT1,MBNL1,THBS1,WNT5A
mibolerone		chemical drug		-1.342	0.00303	C4A/C4B,CALU,CEACAM1,EIF2S1,FHL1
ERK		group		-1.34	0.0279	ADAM10,JAG1,MCAM,MUC5AC,THBS1
decitabine		chemical drug		-1.301	0.00391	ABO,CXCL12,CXCR4,FILIP1L,JAG1,LEPR,TFRC,TGFB2,THBS1,WNT5A
SB203580		chemical - kinase inhibitor		-1.234	0.00029	ADAMTS9,C4A/C4B,CEACAM1,CREB1,FLT1,MUC5AC,NEFH,TGFB2,THBS1,VDR
Insulin		group		-1.21	0.0184	ADAM10,GRB10,LEPR,MLXIPL,SLC38A2,SLC38A3,VLDLR
PTEN		phosphatase		-1.2	0.0182	ANGPT2,CXCR4,FLT1,ID4,JAG1,PDCD4,TAF13,THBS1

PTGS2		enzyme		-1.157	0.000179	ANGPT2,CXCL12,CXCR4,DLK1,EDNRA,FLT1,PTGER2
F2R		G-protein coupled receptor		-1.131	0.0011	ANGPT2,CXCL12,FLT1,THBS1
ERK1/2		group		-1.118	0.00137	ANGPT2,CXCL12,CXCR4,FLT1,LEPR,MUC5AC,NANOG,PTGER2
fluticasone propionate		chemical drug		-1.109	0.0083	EMP1,MDFIC,MUC5AC,PTGER2,THBS1
PI3K (complex)		complex		-1.109	0.0188	CREB1,CXCR4,MCAM,NANOG,PDCD4,THBS1
SNAI2		transcription regulator		-1.109	5.08E-05	CXCL12,CXCR4,ID4,NANOG,VDR
methylprednisolone		chemical drug		-1.081	0.0153	ABCC9,CYP4F3,EMP1,FGL2,LYVE1,MDFIC,MLXIPL,SLCO2A1,TFRC
D-glucose		chemical - endogenous mammalian		-1.071	0.0457	ANGPT2,CXCR4,JAG1,MLXIPL,PDCD4,S100A4,TFRC,THBS1
TP53		transcription regulator		-1.019	0.0013	CALU,CES2,CLIC4,CXCL12,EDIL3,FHL1,GABPA,KCNMA1,LTBP1,MAP4K5,MBNL2,MCAM,ME2,NANOG,NUPR1,S100A4,SEMA6A,TANK,TCF7L2,TGFB2,THBS1,VDR
8-bromo-cAMP		chemical reagent		-1.015	0.039	CREB1,ENTPD1,TGFB2,VLDLR
isobutylmethylxanthine		chemical toxicant		-1	0.0212	CALCR,CREB1,CXCR4,NCOA2
MMP9		peptidase		-1	0.0023	CALCR,CXCL12,CXCR4,JAG1
CDKN1A		kinase		-1	0.0207	ATRX,EDNRA,JAG1,MBNL2,SPAG5
ciprofibrate		chemical drug		-1	0.0212	CEACAM1,CXCL12,LHPP,SLCO2A1
SOX4		transcription regulator		-0.97	0.0021	EMP1,ENPEP,FHL1,MAGI1,MAP4K5,TES
HIF1A		transcription regulator		-0.843	0.000621	ADAM10,CXCL12,CXCR4,CYP4F3,FHL1,FLT1,HIST1H2BK,TFRC,TGFB2,THBS1
KLF4		transcription regulator		-0.826	0.0361	FLT1,NANOG,TGFB2,TNC,WNT5A
poly rI:rC-RNA		biologic drug		-0.823	0.00126	ADAM10,CEACAM1,CXCR4,CYP4F2,NANOG,SLC26A4,TANK,TG,THBS1,WNT5A
bucladesine		chemical toxicant		-0.78	0.00191	CALCR,CREB1,CXCR4,ID4,MCAM,TGFB2,UTRN,VDR
DICER1		enzyme		-0.762	0.000899	ANGPT2,CXCR4,ENTPD1,MCAM,NEFH,PDCD4,THBS1,TM4SF1
WNT3A		cytokine		-0.755	0.00229	ADAM10,CXCL12,NANOG,TGFB2,THBS1,TNC,WNT5A
beta-estradiol		chemical - endogenous mammalian		-0.718	3.71E-06	C4A/C4B,CALCR,CREB1,CXCL12,CXCR4,EIF2S1,ELF1,EMP1,FHL1,GRB10,HNF1B,ID4,IQGAP1,JAG1,LEPR,LTBP1,MSMO1,NCOA2,NUPR1,PDCD4,PTGER2,SLC38A2,SSBP2,SULT1C2,TANK,TGFB2,THBS1,TM4SF1,TSPAN5,VDR,WNT5A
TGFB2	-1.8	growth factor		-0.715	0.000889	ID4,JAG1,TGFB2,THBS1,VDR
trichostatin A		chemical drug		-0.705	0.00416	CXCL12,CXCR4,ELF1,FLT1,LEPR,MBNL1,NEFH,SSBP2,THBS1,UTRN,WNT5A
IL2		cytokine		-0.696	0.00706	CEACAM1,CREB1,CXCL12,CXCR4,ELF1,EMP1,ENTPD1,FGL2,PDCD4,S100A4
ACVRL1		kinase		-0.686	0.0001	ANGPT2,CXCL12,CXCR4,LYVE1
FGF2		growth factor		-0.666	0.00029	ANGPT2,CXCL12,CXCR4,FLT1,LYVE1,MCAM,MUC5AC,S100A4,TGFB2,THBS1
tretinoin		chemical - endogenous mammalian		-0.66	4.43E-08	ADAM10,CALCR,CEACAM1,CHN2,CPE,CXCR4,CYP4F2,CYP4F3,EFS,FERMT1,FLT1,GPX2,GRB10,HNF1B,LEPR,MECOM,MUC5AC,NCOA2,NEFH,PDCD4,RASAL2,SLC38A2,SLCO2A1,SMAD5,TFRC,TGFB2,THBS1,TNC,TPCN1,TPM4,VDR,WNT5A
HMOX1		enzyme		-0.651	0.00124	ANGPT2,CXCL12,FLT1,TGFB2,THBS1
PGR		ligand-dependent nuclear receptor		-0.647	0.0153	DNAJB4,EDNRA,GRB10,TM4SF1,TNC,VDR
ATF4		transcription regulator		-0.64	0.000842	GRB10,JAG1,NUPR1,SLC38A2,SLC38A3,SSBP2
TNF		cytokine		-0.619	0.000696	ANGPT2,CLIC4,CREB1,CXCL12,CXCR4,EMP1,FAM198B,FERMT1,FLT1,JAG1,LYVE1,MAGI1,MCAM,MECOM,MUC5AC,NCOA2,NEFH,TFRC,TG,TGFB2,THBS1,TM4SF1,TNC,VDR,WNT5A
resveratrol		chemical drug		-0.61	0.00533	CYP4F2,JAG1,MUC5AC,PDCD4,TFRC,TGFB2,VDR
dexamethasone		chemical drug		-0.584	0.000207	ADAM10,ALDH6A1,CALCR,CEACAM1,CES2,CLIC4,CPE,CXCL12,CXCR4,DLK1,EDNRA,FILIP1L,IQGAP1,JAG1,LYVE1,MUC5AC,PDCD4,PLCL2,SLCO2A1,SMAD5,TFRC,TGFB2,THBS1,TNC,VDR,WNT5A

progesterone		chemical - endogenous mammalian		-0.571	0.00396	CXCL12,CXCR4,DNAJB4,EDNRA,ENPEP,FLT1,GRB10,LEPR,PTGER2,TGFB2,VDR
CD40LG		cytokine		-0.555	0.00718	CXCR4,EIF2S1,FLT1,NEFH,STK4,TANK,TFRC,WNT5A
TGM2		enzyme		-0.555	0.0238	CEACAM1,CYP4F3,FERMT1,THBS1,TPCN1
spironolactone		chemical drug		-0.555	0.00253	CXCR4,KCNMA1,MLXIPL,SLCO2A1
EGF		growth factor		-0.512	0.0107	CXCR4,FLT1,MUC5AC,NCOA2,PDCD4,S100A4,THBS1,TNC,VDR
EPO		cytokine		-0.487	0.000249	ANGPT2,CXCL12,CXCR4,MIA2,NUPR1,PDCD4,PLCL2,TFRC,ZNF711
EGFR		kinase		-0.456	0.00729	CXCL12,CXCR4,JAG1,MUC5AC,PTGER2,THBS1,TNC,VDR
VCAN		other		-0.447	0.00258	ADAMTS9,C4A/C4B,CPE,CXCL12,TCF7L2
ADCYAP1		other		-0.447	0.0275	BAG2,CREB1,EDIL3,MCAM,TGFB2
CEBPB		transcription regulator		-0.415	0.00942	CXCR4,FHL1,LEPR,NUPR1,SLC38A2,TM4SF1,VDR,VLDLR
cyclosporin A		biologic drug		-0.415	1.86E-05	C4A/C4B,CREB1,CXCR4,EDIL3,EDNRA,FLT1,GPX2,LYVE1,PDCD4,PRKACB,TANK,UTRN,WNT5A
lipopolysaccharide		chemical drug		-0.367	0.0025	ANGPT2,CALCR,CEACAM1,CLIC4,CXCL12,CXCR4,CYP4F2,FLT1,JAG1,MUC5AC,NUPR1,PDCD4,PTGER2,SLCO2A1,TANK,TCF7L2,TFRC,TGFB2,THBS1,TNC,UTRN,VDR,VLDLR
PPIF		enzyme		-0.277	0.00491	CXCL12,CXCR4,DLK1,WNT5A
P38 MAPK		group		-0.256	0.00487	CXCL12,CXCR4,CYP4F3,MUC5AC,THBS1,VDR,VLDLR,WNT5A
AGT		growth factor		-0.25	0.00149	ANGPT2,CREB1,EDNRA,FLT1,JAG1,LEPR,MSMO1,SEMA6A,TFRC,TGFB2
cyclic AMP		chemical - endogenous mammalian		-0.218	0.0393	CREB1,FLT1,PTGER2,RAPGEF3,TG
LY294002		chemical - kinase inhibitor		-0.207	7.19E-05	ENTPD1,FLT1,GRB10,HNF1B,NANOG,NEFH,PTGER2,SLC38A3,SPAG5,SULT1C2,TG,THBS1,WNT5A
F2		peptidase		-0.184	0.000116	ADAMTS9,ANGPT2,CXCR4,ENTPD1,FHL1,FLT1,KIF13A,TGFB2,THBS1
IKBKB		kinase		-0.152	0.0128	CALCR,CXCL12,CXCR4,MUC5AC,S100A4,TNC
rosiglitazone		chemical drug		-0.128	0.0326	ABCC9,C4A/C4B,CREB1,EDNRA,THBS1,TNC,VLDLR
GATA6		transcription regulator		-0.055	0.000267	C4A/C4B,DLK1,HNF1B,LTBP1,LYVE1,NANOG,TGFB2
VEGFA		growth factor		-0.011	3.19E-05	ANGPT2,CEACAM1,CLIC4,CXCL12,CXCR4,FLT1,GRB10,ME2,THBS1,TNC
Hdac		group		0	0.0295	ADGRL3,CXCR4,EDNRA,HNF1B
Nr1h		group		0	0.0221	EDNRA,FLT1,THBS1,VLDLR
Nfat (family)		group		0	0.00421	CALCR,FHL1,KIF13A,TGFB2
OGA		enzyme		0	0.000337	CALCRL,CEACAM1,CPE,ELF1,HSF2,MCAM,MSMO1,THBS1,TNC,TSPAN5
IRF4		transcription regulator		0	0.0216	CXCR4,ENTPD1,FLT1,PDCD6
CD44		other		0	0.0157	LTBP1,NANOG,PDCD4,THBS1,WNT5A
ANGPT2	-1.8	growth factor		0	0.00261	DNAJB4,LEPR,MAP4K5,PDCD4,SPTLC3,THBS1
INSIG1		other		0	0.0063	CXCR4,CYP4F2,DLK1,MLXIPL
ERG		transcription regulator		0	0.00289	CXCR4,FHL1,FLT1,MAGI1,THBS1,UTRN
SMARCB1		transcription regulator		0	0.0337	C4A/C4B,CEACAM1,CXCR4,SMAD5
phorbol myristate acetate		chemical drug		0.049	0.00517	ANGPT2,CALCR,CPE,CREB1,CXCR4,FHL5,FLT1,LEPR,LYVE1,MAST4,MUC5AC,PDCD4,PTGER2,THBS1,VDR,VLDLR
BMP7		growth factor		0.068	0.0123	DLK1,GRB10,S100A4,SMAD5
medroxyprogesterone acetate		chemical drug		0.068	0.0134	ANGPT2,CXCR4,TNC,WNT5A
LEP		growth factor		0.069	0.000328	ANGPT2,DLK1,FLT1,LEPR,MLXIPL,MSMO1,MUC5AC,SLC13A2,TG,THBS1,WNT5A
FGFR2		kinase		0.185	0.00241	CPE,ENPEP,FAM198B,GRB10,NANOG
FSH		complex		0.2	6.26E-06	CPE,CXCR4,FILIP1L,HSF2,ID4,MSMO1,PTGER2,RASAL2,SMAD5,TFRC,TGFB2,THBS1,TLK1

TNFSF11		cytokine		0.205	0.0377	CALCR,GABPA,GRB10,PTGER2,TSPAN5
tetrachlorodibenzodioxin		chemical toxicant		0.254	0.00536	ENPEP,JAG1,MUC5AC,S100A4,SLC38A3,TGFB2,TNC,VLDLR
ERBB3		kinase		0.29	0.000434	CXCL12,CXCR4,STAG2,THBS1,TNC
IFNG		cytokine		0.303	0.0002	ADAMTS9,C4A/C4B,CEACAM1,CLIC4,CXCL12,CXCR4,EDNRA,FGL2,FLT1,JAG1,KCNMA1,NUPR1,RSPH14,TCF7L2,TFRC,TG,TGFB2,THBS1,VDR,VLDLR,WNT5A,ZFX
CREB1	-1.7	transcription regulator		0.308	0.000231	CREB1,CXCR4,EDIL3,ENTPD1,FGL2,FLT1,MCAM,MSMO1,MUC5AC,SSBP2,TAF13,TFRC,TG
fulvestrant		chemical drug		0.363	0.000642	CXCL12,FLT1,GABPA,LEPR,PDCD4,TFRC,TGFB2,ZFX
PKD1		ion channel		0.391	0.00997	CALCR,EDIL3,LTBP1,SLC13A2,ZNF711
calcitriol		chemical drug		0.4	0.000212	ANGPT2,CALCR,ENPEP,LTBP1,NUPR1,PDCD4,PTGER2,SPAG5,SULT1C2,TGFB2,THBS1,TLK1,VDR
IL13		cytokine		0.403	0.000126	CALCR,CHN2,CXCR4,JAG1,LTBP1,MSMO1,MUC5AC,PCM1,SLC26A4,THBS1,WNT5A
IL15		cytokine		0.403	0.0348	CEACAM1,CXCL12,CXCR4,ID4,PDCD4,TFRC,VDR
SP1		transcription regulator		0.415	0.0186	CEACAM1,CXCL12,CXCR4,FLT1,ID4,MUC5AC,TGFB2,TNC,VDR
TP73		transcription regulator		0.43	0.0338	ADAM10,JAG1,LTBP1,NUPR1,THBS1,VDR
CEBPA		transcription regulator		0.437	0.000061	CXCR4,FHL1,FLT1,GABPA,GGH,HNF1B,LEPR,TANK,TGFB2,THBS1,VDR,VLDLR
NOTCH1		transcription regulator		0.449	7.65E-05	DLK1,FLT1,JAG1,LYVE1,MCAM,MUC5AC,PROX1,TGFB2,WNT5A
forskolin		chemical toxicant		0.483	0.00913	CALCR,CREB1,CXCR4,MSMO1,NCOA2,RAPGEF3,RASAL2,THBS1,TLK1,TM4SF1,VDR
CSF3		cytokine		0.508	0.035	CXCL12,CXCR4,ENTPD1,TFRC
Lh		complex		0.577	0.00046	CREB1,CXCR4,MSMO1,RAPGEF3,RASAL2,TGFB2,THBS1,TLK1
raloxifene		chemical drug		0.6	0.000241	CALCR,CXCL12,GABPA,TFRC,TGFB2,TM4SF1,ZFX
indomethacin		chemical drug		0.6	0.00178	ANGPT2,C4A/C4B,CXCR4,FLT1,GPX2,TES,VDR
testosterone		chemical - endogenous mammalian		0.6	0.0329	ABCC9,EIF2S1,TG,TGFB2,THBS1,WNT5A
NS-398		chemical reagent		0.651	3.64E-05	ANGPT2,CXCR4,MAGI1,MUC5AC,PDCD4,TFRC
L-dopa		chemical - endogenous mammalian		0.671	0.000103	C4A/C4B,CXCL12,DLK1,GRB10,HNRNPL,IQGAP1,ITGB1BP1,LTBP1,MBNL2,MSMO1,PDCD4,PDE4DIP,SLC22A18,SLC38A2,VLDLR
IGF1		growth factor		0.711	0.00267	ANGPT2,C4A/C4B,CREB1,LEPR,MUC5AC,NEFH,NUPR1,TG,THBS1
valproic acid		chemical drug		0.728	0.000443	BAG2,CXCR4,EIF2S1,ELF1,MSMO1,S100A4,SSBP2,TES,THBS1,UTRN,WNT5A
docosahexaenoic acid		chemical drug		0.762	0.0295	CXCR4,MLXIPL,TGFB2,THBS1
triamcinolone acetonide		chemical drug		0.816	0.00415	EDNRA,EFS,SEMA6A,TCF7L2,TGFB2,VLDLR
estrogen receptor		group		0.816	0.00769	CXCL12,CXCR4,JAG1,TGFB2,TNC,WNT5A
RBPJ		transcription regulator		0.849	0.00353	CXCL12,JAG1,TGFB2,TNC,WNT5A
ERBB2		kinase		0.861	0.000338	BAG2,CDC40,CXCL12,DPT,EMP1,FLT1,JAG1,PDCD4,RETREG1,S100A4,SPAG5,THBS1,TNC,VDR,WNT5A
KLF2		transcription regulator		0.865	1.51E-05	ANGPT2,CXCR4,FLT1,LEPR,SLCO2A1,TFRC,TGFB2,THBS1
TP63		transcription regulator		0.954	2.12E-05	CEACAM1,CXCR4,EMP1,GPX2,JAG1,NANOG,S100A4,TGFB2,THBS1,TNC,VDR,WNT5A
HNF4A		transcription regulator		0.971	0.000694	ABHD6,C4A/C4B,CEACAM1,CES2,CYP4F2,CYP4F3,DNAJB4,FAM216A,FHL1,GABPA,GPX2,HNF1B,MLXIPL,NXT2,PATJ,PDCD4,PDE4DIP,PGGT1B,PROZ,RIOK3,SLC22A18,SULT1C2,TCF7L2,TFRC,TNC,TPCN1,USH1C,ZNF277
SOX2		transcription regulator		1	0.00769	CXCR4,ELF1,FLT1,JAG1,NANOG,TGFB2,WNT5A
trans-hydroxytamoxifen		chemical drug		1	0.0101	CALCR,CXCL12,TGFB2,TM4SF1
topotecan		chemical drug		1	0.0431	C4A/C4B,KCNMA1,MBNL2,TANK
ethionine		chemical toxicant		1	0.000173	C4A/C4B,GPX2,NANOG,TES
PD98059		chemical - kinase inhibitor		1.05	0.0064	ANGPT2,CREB1,CXCR4,EMP1,ENTPD1,MUC5AC,S100A4,SLC38A2,THBS1,VDR

PRKCD		kinase		1.067	0.0403	CXCL12,MAST4,MUC5AC,PDCD4
FOXA1		transcription regulator		1.086	0.000787	ALDH6A1,HNF1B,JAG1,MLXIPL,MUC5AC,NANOG
JUN		transcription regulator		1.091	0.016	LTBP1,NANOG,NEFH,SLC38A2,THBS1,TNC,VDR,WNT5A
HNF1A		transcription regulator		1.134	9.03E-05	ABCC9,ENPEP,GPX2,HNF1B,KIF13A,MIA2,MLXIPL,PAMR1,PATJ,SULT1C2,TFRC,VLDLR
GDF2		growth factor		1.179	0.000434	CXCL12,CXCR4,DLK1,LYVE1,PROX1
mir-21		microRNA		1.188	0.0377	FGL2,JAG1,LEPR,PDCD4,SMAD5
sirolimus		chemical drug		1.195	0.0296	CXCR4,EIF2S1,LEPR,MAP4K5,NANOG,PDCD4,S100A4,SLC38A3
deferoxamine		chemical drug		1.253	0.0143	ANGPT2,FLT1,TFRC,TGFB2,VDR
hydrogen peroxide		chemical - endogenous mammalian		1.328	0.0249	CXCR4,MCAM,MSMO1,MUC5AC,PDCD4,RIOK3,TFRC,VDR
IL17A		cytokine		1.331	0.00114	CXCL12,EDIL3,ENTPD1,JAG1,MLXIPL,MUC5AC,TNC
FEV		transcription regulator		1.342	0.00039	CALCR,CXCR4,PROX1,SYT1,TCF7L2
MAPK1		kinase		1.387	0.0377	EMP1,MUC5AC,NANOG,NUPR1,PTGER2,VDR
nitric oxide		chemical - endogenous mammalian		1.445	0.0278	CXCL12,FLT1,TFRC,THBS1
ERBB4		kinase		1.606	0.00344	CXCL12,STAG2,THBS1,TNC
APP		other		1.954	1	C4A/C4B,CXCL12,CXCR4,MAGI1,PDCD4
miR-1-3p (and other miRNAs w/seed GGAAUGU)		mature microRNA		1.977	0.0515	PDCD4,THBS1,TPM4,UTRN
Immunoglobulin		complex		1.982	0.0971	CHN2,ENTPD1,TCF7L2,TFRC
mir-1		microRNA	Activated	2	0.00115	CREB1,NXT2,PDCD4,TMX1,UTRN
miR-30c-5p (and other miRNAs w/seed GUAAACA)		mature microRNA	Activated	2	0.00794	ATRX,MBNL1,SLC38A2,WNT5A
INSR		kinase	Activated	2.19	0.00218	ALDH6A1,CXCR4,DLK1,FHL1,GRB10,IQGAP1,LEPR,MSMO1,PDCD4
DGCR8		enzyme	Activated	2.213	3.22E-05	ID4,JAG1,PDCD4,TCF7L2,TNC
miR-124-3p (and other miRNAs w/seed AAGGCAC)		mature microRNA	Activated	2.213	0.0266	CPNE3,IQGAP1,JAG1,MDFIC,SMAD5
CSF2		cytokine	Activated	2.391	0.000111	C4A/C4B,CEACAM1,CREB1,CXCR4,FLT1,GABPA,PROZ,PTGER2,SPAG5,THBS1,TPM4,VDR
platelet activating factor		chemical - endogenous mammalian			0.00654	ANGPT2,MUC5AC,THBS1
16,16-dimethylprostaglandin E2		chemical - endogenous mammalian			0.0492	PTGER2
12(R)-hydroxyeicosatetraenoic acid		chemical - endogenous mammalian			0.00718	MUC5AC
propylthiouracil		chemical drug			0.00721	DLK1,ENPEP,TG,VLDLR
PP1		chemical - kinase inhibitor			0.0304	ANGPT2,CXCR4
dextran sulfate		chemical drug			0.0297	C4A/C4B,PROZ,SPAG5,TGFB2,THBS1

xanthine		chemical - endogenous mammalian			0.0044	CXCR4,MUC5AC
L-threonine		chemical - endogenous mammalian			0.00222	EIF2S1,NANOG
hyaluronic acid		chemical - endogenous mammalian			0.048	CXCR4,PDCD4,TGFB2
allopurinol		chemical drug			0.0226	C4A/C4B,GPX2,TES
peptidoglycan		chemical - endogenous non-mammalian			0.0449	CXCR4,MUC5AC,VDR
nitroglycerin		chemical drug			0.0027	ADAM10,FLT1
dexamethasone phosphate		chemical drug			0.000658	CXCR4,LYVE1,THBS1
nitrate		chemical - endogenous mammalian			0.0354	TG
chloride		chemical - endogenous mammalian			0.0214	SLC26A4
fludrocortisone		chemical drug			0.0354	CXCR4
ATP		chemical - endogenous mammalian			0.0363	ADAMTS9,CXCR4,THBS1
triamcinolone		chemical drug			0.0423	CALCR
deflazacort		chemical drug			0.00718	EDNRA
CD300LF		other			0.0214	CALCR
bevacizumab		biologic drug			2.95E-05	ANGPT2,CXCL12,CXCR4
Cobra Venom Factor		chemical - endogenous non-mammalian			0.0423	TGFB2
zibotentan		chemical drug			0.0284	EDNRA
hydroxamic acid		chemical - other			0.00718	CXCR4
STAT5a/b		group			0.0279	EMP1,LEPR,PROX1
iodide		chemical - endogenous mammalian			0.0423	SLC26A4
mineralocorticoid		chemical drug			0.0214	SLC26A4
deoxycorticosterone acetate		chemical drug			0.0127	SLC26A4,TNC
Cyclin D		group			0.0492	NANOG
TGFBR		group			0.0423	WNT5A
Rar		group			0.0234	ADAM10,CYP4F2
Ctnna		group			0.0354	CXCR4
Ap1		complex			0.00513	CXCL12,MUC5AC,S100A4,UTRN,VDR

Rxr		group			0.0063	ADAM10,CYP4F2,DLK1,HNF1B
TD114-2		chemical - kinase inhibitor			0.00718	NANOG
conjugated linoleic acid		chemical drug			0.0304	CALCR,CXCR4
CYP2R1		enzyme			0.00718	VDR
HOXD8		transcription regulator			0.000754	ANGPT2,PROX1
GLDN		other			0.0214	NFASC
VIPAS39		other			0.0423	THBS1
DAND5		other			0.0284	NANOG
FOXN4		transcription regulator			0.0354	PROX1
indican		chemical - endogenous mammalian			0.00104	ANGPT2,CXCR4,THBS1
ZNF652		other			0.0492	TGFB2
GPR17		G-protein coupled receptor			0.0214	RAPGEF3
elocalcitol		chemical drug			0.0354	TGFB2
MARCH7		other			0.00718	NANOG
Meg3		other			0.000304	FLT1,IQGAP1
CHI3L1		enzyme			0.0149	EMP1,TNC
CLEC1B		transmembrane receptor			0.00322	CXCL12,TNC
SV2B		transporter			0.00718	SYT1
BTNL2		transmembrane receptor			0.0168	ENTPD1,FGL2,S100A4
RNF31		enzyme			0.0108	CXCR4,KCNMA1
MIR17HG		other			0.0127	CREB1,HNF1B
NQO2		enzyme			0.00222	CXCL12,CXCR4
MARCH2		enzyme			0.0143	TFRC
TRPS1		transcription regulator			0.000578	KCNMA1,SEMA6A,VDR
AGO1		translation regulator			0.0492	CXCR4
TACSTD2		other			0.0284	CREB1
Collagen(s)		complex			0.00413	C4A/C4B,THBS1,TNC
MYLIP		enzyme			0.0214	VLDLR
IGF1R		transmembrane receptor			0.0398	CALU,DLK1,GRB10,NANOG,S100A4
NLN		peptidase			0.0284	CREB1
DUOX1		enzyme			0.0143	MUC5AC
SLC25A33		transporter			0.0143	GABPA
Laminin (complex)		complex			0.0366	TGFB2,WNT5A
SRC (family)		group			0.0483	FLT1,S100A4
Pde3		group			0.0143	ENTPD1
COL1A1		other			0.00379	CLIC4,CXCR4
SPDEF		transcription regulator			0.0129	MUC5AC,TNC,WNT5A
SRRT		other			0.0143	PDCD4
BMPR2		kinase			0.0117	CLIC4,CXCL12
ZG16B		other			0.00718	CXCR4

SFRP5		transmembrane receptor			0.0492	CXCR4
IL33		cytokine			0.0295	FLT1,JAG1,LEPR
KDM4B		enzyme			0.0214	NANOG
PRPF19		enzyme			0.0354	NANOG
PPP1R14B		phosphatase			0.0423	FLT1
ZMYND10		other			0.0423	THBS1
SULT2B1		enzyme			0.0354	VLDLR
KDM4A		transcription regulator			0.00649	FHL1,NANOG
NSD2		enzyme			0.0304	CXCR4,RAB23
SV2A		transporter			0.0143	SYT1
FOXI1		transcription regulator			0.0354	SLC26A4
AGO3		translation regulator			0.0143	CXCR4
TRIM65		other			0.0284	PDCD4
SOX2-OCT4		complex			0.0492	NANOG
PDS5B		other			0.0492	NANOG
Oxi-4503		chemical drug			0.00718	CXCL12
RO4929097		chemical drug			0.0423	JAG1
AMH		growth factor			0.0149	CEACAM1,SMAD5
LDL-cholesterol		complex			0.0423	CXCR4
botulinum toxin type A		biologic drug			0.0143	S100A4
strontium ranelate		chemical drug			0.0492	WNT5A
firtecan pegol		chemical drug			0.0284	CXCR4
SRA1		transcription regulator			0.00983	TGFB2,THBS1
GLRX3		enzyme			0.0284	TFRC
RRM2		enzyme			0.0284	THBS1
NR0B1		ligand-dependent nuclear receptor			0.0334	MLXIPL,NANOG
CCND1		transcription regulator			0.0338	CALU,CPNE3,NANOG,PDCD4,UTRN,VPS13D
XDH		enzyme			0.0275	CXCR4,MUC5AC
DLL4		other			0.0149	FLT1,LYVE1
ASCL2		transcription regulator			0.0492	CXCR4
PDGFD		growth factor			0.0423	CXCR4
PPP2R2C		phosphatase			0.0284	NEFH
SRF		transcription regulator			0.035	CXCR4,FHL1,FILIP1L,FLT1,LEPR,TNC
hexachlorobenzene		chemical toxicant			0.008	C4A/C4B,GPX2,TES
PGF		growth factor			0.0289	CXCR4,FLT1
LYL1		transcription regulator			0.00727	ANGPT2,ID4
IL3RA		transmembrane receptor			0.0143	PROX1
PI3K (family)		group			0.0381	CXCL12,CXCR4,TNC
VHL		transcription regulator			0.0141	CXCL12,CXCR4,FILIP1L,TFRC
TFPI		other			0.0423	VLDLR
SMAD2		transcription regulator			0.0234	FLT1,NANOG,THBS1
SELL		transmembrane receptor			0.0492	CXCR4
CFB		peptidase			0.00575	C4A/C4B,TGFB2

GMNN		transcription regulator			0.0206	NANOG,TGFB2,WNT5A
SETD2		enzyme			0.0247	FLT1,WNT5A
TSHR		G-protein coupled receptor			0.0492	TG
TTR		transporter			0.0284	TG
ZNF281		transcription regulator			0.0284	NANOG
RPS11		other			0.0284	PDCD4
RAD23B		other			0.0143	NANOG
A2M		transporter			0.0466	CREB1,WNT5A
IBSP		other			0.0143	THBS1
ADRA2B		G-protein coupled receptor			0.0284	FLT1
PDIA4		enzyme			0.00718	TG
INHBB		growth factor			0.00575	THBS1,TNC
ACVR1		kinase			0.0431	EDIL3,SRPX2
PLEC		other			0.0214	CXCR4
CELA2A		peptidase			0.0284	MUC5AC
CDK9		kinase			0.00654	CXCR4,MAGI1,WNT5A
CAT		enzyme			0.00587	MUC5AC,THBS1,TNC
STC1		kinase			0.0423	TGFB2
MSX2		transcription regulator			0.0261	PROX1,TGFB2
ID1		transcription regulator			0.0115	CEACAM1,NANOG,TG
C3AR1		G-protein coupled receptor			0.0466	LTBP1,TGFB2
ZFP36L2		transcription regulator			0.0423	NEFH
ADAM10	-1.7	peptidase			0.00344	ATRX,CALCRL,CXCL12,SYT1
NRCAM		other			0.0214	NFASC
DMD		other			0.0383	KCNMA1,PAMR1,PROX1,UTRN
ZBTB46		transcription regulator			0.0354	CREB1
GNAS		enzyme			0.0398	MUC5AC,TG
Pzp		other			0.0423	VLDLR
EGLN1		enzyme			0.00921	ANGPT2,CXCR4,TGFB2
KRT19		other			0.0423	EIF2S1
CD24		other			0.00769	MBNL1,MCAM,THBS1,VDR
RASSF1		other			0.0271	CPE,CXCR4,EMP1
MMP14		peptidase			0.0221	JAG1,SLCO2A1
EIF3E		other			0.0334	THBS1,TNC
CCL18		cytokine			0.0492	S100A4
FOXP3		transcription regulator			0.0363	ENTPD1,FGL2,PDCD4
GFI1		transcription regulator			0.0345	CXCR4,ENTPD1,VDR
SUMO2		enzyme			0.0289	CEACAM1,TNC
PROC		peptidase			0.0431	ANGPT2,THBS1
SERPINE1		other			0.0261	DLK1,FLT1
EAF2		transcription regulator			0.0284	THBS1
SMC3		other			0.00506	CXCL12,THBS1
MIR124		group			0.0195	IQGAP1,NANOG

miR-291a-3p (and other miRNAs w/seed AAGUGCU)		mature microRNA			0.0256	MBNL2,PRKACB,STK4
miR-516a-3p (and other miRNAs w/seed GCUUCCU)		mature microRNA			0.0284	WNT5A
miR-2682-5p (and other miRNAs w/seed AGGCAGU)		mature microRNA			0.0214	CREB1
miR-18a-5p (and other miRNAs w/seed AAGGUGC)		mature microRNA			0.00506	HSF2,THBS1
mir-27		microRNA			0.0062	BAG2,FLT1,SEMA6A
miR-183-5p (miRNAs w/seed AUGGCAC)		mature microRNA			0.0354	PDCD4
miR-141-3p (and other miRNAs w/seed AACACUG)		mature microRNA			0.00725	JAG1,NANOG,TGFB2
mir-181		microRNA			0.0415	CREB1,NANOG
miR-137-3p (miRNAs w/seed UAUUGCU)		mature microRNA			0.0214	NCOA2
mir-128		microRNA			0.0284	BAG2
mir-122		microRNA			0.0256	CLIC4,CREB1,IQGAP1
mir-320		microRNA			0.0423	TFRC
miR-21-5p (and other miRNAs w/seed AGCUUAU)		mature microRNA			0.00879	JAG1,PDCD4,TGFB2
mir-8		microRNA			0.0226	LEPR,SMAD5,TGFB2
mir-135		microRNA			0.016	CXCL12,SMAD5
miR-23a-3p (and other miRNAs w/seed UCACAUU)		mature microRNA			0.00894	CXCL12,SMAD5
miR-802-5p (miRNAs w/seed CAGUAAC)		mature microRNA			0.00718	HNF1B
mir-663		microRNA			0.0143	CXCR4
mir-652		microRNA			0.0143	JAG1
miR-3118 (and other miRNAs w/seed GUGACUG)		mature microRNA			0.000754	CREB1,NANOG
AKT1		kinase			0.0449	CXCR4,HNRNPL,MSMO1,TGFB2,THBS1
NEUROG1		transcription regulator			0.0115	FAM198B,S100A4,THBS1
AGER		transmembrane receptor			0.0151	CALCR,FLT1,TNC
SOX18		transcription regulator			0.0354	PROX1
STAT3-Brachyury		complex			0.00718	NANOG
NUBP1		other			0.0143	TFRC

PTGER2	-1.9	G-protein coupled receptor			0.04	CXCR4,SPAG5,THBS1
NQO1		enzyme			0.0289	CXCL12,CXCR4
DNM1L		enzyme			0.0354	NANOG
RCE1		peptidase			0.00317	CXCL12,TGFB2,ZFR
NKX2-1		transcription regulator			0.041	EDIL3,TG,TGFB2,THBS1
ATN1		transcription regulator			0.016	MCAM,NEFH,PRKACB,TM4SF1
EPHB4		kinase			0.00587	CXCR4,TGFB2,WNT5A
ANKH		transporter			0.0284	WNT5A
SUMO3		other			0.0304	CEACAM1,TNC
ANGPT1		growth factor			0.0319	ANGPT2,JAG1
CYP4F2	4.2	enzyme			0.0492	FLT1
PTGES3		enzyme			0.00178	NUPR1,TM4SF1
RNF4		transcription regulator			0.0354	GABPA
SRSF3		other			0.0423	PDCD4
SDC4		other			0.0423	ANGPT2
SSPN		other			0.0423	UTRN
GLI2		transcription regulator			0.0354	JAG1,NANOG,VDR
LNX2		other			0.0027	JAG1,TCF7L2
LIMA1		other			0.0027	CREB1,VDR
NR5A1		ligand-dependent nuclear receptor			0.0124	ANGPT2,C4A/C4B,LEPR
MBP		other			0.0117	LEPR,NEFH
GUCA2B		other			0.00718	SLC26A4
IGFBP3		other			0.0261	LYVE1,TNC
COL7A1		other			5.12E-05	THBS1,WNT5A
GPS2		transcription regulator			0.0466	LHPP,MBNL1
NRP2		kinase			0.0214	S100A4
FTMT		enzyme			0.0214	TFRC
ETF1		translation regulator			0.00718	UTRN
SP3		transcription regulator			0.0196	ANGPT2,CEACAM1,FLT1,MUC5AC,TNC
FOXA2		transcription regulator			0.00274	CXCR4,DLK1,HNF1B,MLXIPL,MUC5AC,WNT5A
SMAD4		transcription regulator			0.025	ANGPT2,JAG1,TGFB2,THBS1,TNC
ELP1		other			0.0423	FLT1
IQGAP2		other			0.0284	IQGAP1
S100A4	-2	other			0.0304	CLIC4,THBS1
ATF1		transcription regulator			0.0366	CREB1,THBS1
IGFBP4		other			0.0492	MCAM
PTH		other			0.0251	CXCL12,CXCR4,ID4,VDR
TRHR		G-protein coupled receptor			0.0284	LEPR
GABPB1		transcription regulator			0.00808	TNC,UTRN
RPL11		other			0.0423	PDCD4
FOLR1		transporter			0.00472	PDCD4,RIOK3,S100A4,SLC13A2
EP300		transcription regulator			0.0363	CXCR4,DNAJB4,NANOG,RAPGEF3,TGFB2,TNC,UTRN
RARB		ligand-dependent nuclear receptor			0.0256	ADAM10,CXCL12,HNF1B

FANCC		other			0.0135	CXCL12,HNF1B,JAG1
F7		peptidase			0.0221	JAG1,PTGER2
HOXA9		transcription regulator			0.0085	CALCRL,CREB1,EDNRA,MAP4K5,MBNL1
CYP11B2		enzyme			0.0284	KCNMA1
CEACAM1	1.52	transporter			0.0304	ANGPT2,CXCR4
GIPC1		other			0.0284	PROX1
VEGFC		growth factor			0.0027	CXCR4,PROX1
PTGS1		enzyme			0.0261	ANGPT2,PTGER2
IL25		cytokine			0.0449	FLT1,JAG1
HNRNPA2B1		other			9.99E-07	ATRX,ENTPD1,FHL1,LEPR,MAST4,SYT1,TGFB2,USH1C,UTRN
HTR4		G-protein coupled receptor			0.0354	CXCL12
GCH1		enzyme			0.0143	TNC
SND1		enzyme			0.00116	EMP1,JAG1,LTBP1
NLRP12		other			0.0261	CXCL12,CXCR4
CTBP1		enzyme			0.0195	THBS1,VDR
PPP1R3A		phosphatase			0.00718	THBS1
FOXD3		transcription regulator			0.00322	CXCR4,NANOG
FAS		transmembrane receptor			0.0015	CREB1,FGL2,FLT1,PLCL2,PTGER2,TFRC,TLK1,TNC,TPM4
FBXL5		enzyme			0.0284	TFRC
RUVBL1		transcription regulator			0.0334	CYP4F3,HIST1H2BK
KLK6		peptidase			0.0284	ADAM10
FOXE1		transcription regulator			0.000304	TG,WNT5A
HNRNPL	1.56	other			0.0143	CEACAM1
MYRF		transcription regulator			0.0354	CEACAM1
TIMP3		other			0.00575	ADAM10,MUC5AC
LASP1		transporter			0.0284	S100A4
MEF2A		transcription regulator			0.0195	SYT1,TGFB2
SMAD3		transcription regulator			0.00696	JAG1,NANOG,SMAD5,TGFB2,THBS1,TNC
LAMC1		other			0.0044	TGFB2,WNT5A
MAD2L2		enzyme			0.0492	NANOG
GGT1		enzyme			0.0284	MUC5AC
ENG		transmembrane receptor			0.0319	CXCR4,THBS1
FOXC2		transcription regulator			0.0466	CXCR4,WNT5A
MEP1B		peptidase			0.0171	GRB10,TFRC
NEURL1		enzyme			0.0214	JAG1
PTCH2		transmembrane receptor			0.000304	CXCL12,JAG1
AGO4		translation regulator			0.0143	CXCR4
RPS5		other			0.0284	PDCD4
THBS1	-1.5	other			0.0171	TGFB2,THBS1
Klf7		transcription regulator			0.00718	TG
2-bromoethylamine		chemical reagent			0.0483	C4A/C4B,GPX2
IND S7		chemical - kinase inhibitor			0.0171	CALU,HNRNPL

GNL3		other			0.0117	EDNRA,NANOG
MEL S3		chemical - kinase inhibitor			0.0183	CALU,HNRNPL
ACKR3		G-protein coupled receptor			0.0138	CXCL12,CXCR4
NR2F2		ligand-dependent nuclear receptor			0.0304	HNF1B,PROX1
LMNB1		other			0.000491	CXCL12,MSMO1,TGFB2,ZFR
ATXN3		peptidase			0.00139	CREB1,CXCL12
WNT11		other			0.0195	NANOG,TGFB2
TG	1.53	other			0.00141	ANGPT2,SLC26A4,TG
A130040M12Rik		other			0.00718	RAB23
LRP1		transmembrane receptor			0.0261	CREB1,WNT5A
YY1		transcription regulator			0.00983	CXCL12,CXCR4,DNAJB4,ID4,PROX1,TGFB2
PGK1		kinase			0.0492	CXCR4
KDM5B		transcription regulator			0.0168	FHL1,ITGB1BP1,MCAM,TGFB2
CDK8		kinase			0.00649	CXCR4,MAGI1
GABPA	-1.5	transcription regulator			0.035	TNC,UTRN
SFPQ		transcription regulator			0.00983	RAB23,SEMA6A
HIVEP3		transcription regulator			0.0423	S100A4
GATA1		transcription regulator			0.00642	ANGPT2,FHL1,LEPR,MECOM,NEFH,TFRC
FOXL1		transcription regulator			0.0143	WNT5A
JAM2		other			0.00718	CXCL12
SRSF6		other			0.0354	TNC
DRP2		other			0.0143	UTRN
ABCB7		transporter			0.0354	TFRC
RAP1A		enzyme			0.0354	TG
FOXO3		transcription regulator			0.00153	ANGPT2,CXCR4,JAG1,MBNL1,NUPR1,SPAG5,STK4,TGFB2
VSNL1		other			0.0284	CREB1
RPS18		other			0.0284	PDCD4
TARBP2		other			0.0492	THBS1
PAX3		transcription regulator			9.45E-05	CXCR4,EIF2S1,FHL1,FILIP1L,ID4,MCAM,TGFB2,TNC
STAMBP		enzyme			0.0143	CXCR4
ANXA1		enzyme			0.0195	CXCR4,ITGB1BP1
ITIH4		other			0.00718	VDR
HRAS		enzyme			0.0261	CALU,CXCL12,FHL1,MCAM,MSMO1,S100A4,TG,TGFB2,THBS1
AQP5		transporter			0.0423	MUC5AC
MATK		kinase			0.0214	CXCR4
TNFSF10		cytokine			0.0345	ANGPT2,CXCR4,TFRC
cetuximab/radiotherapy		biologic drug			0.0214	FLT1
CSNK1A1		kinase			0.0284	CXCR4
CD164		other			0.00718	CXCR4
EXT1		enzyme			0.0423	NANOG
BMP2		growth factor			0.0151	CLIC4,CXCL12,ID4,TG,VDR
THBS4		other			0.016	TGFB2,TNC
SFTPC		other			0.000754	MUC5AC,S100A4

NRXN1		transporter			0.00155	CXCL12,THBS1,TNC
DIO2		enzyme			0.0247	NEFH,TG
17beta-estradiol dendrimer conjugate		chemical reagent			0.0143	CALCR
PTPN3		phosphatase			0.0214	VDR
IGKC		other			0.0423	VDR
NAB2		transcription regulator			0.00808	FLT1,ID4
FZD2		G-protein coupled receptor			0.00718	WNT5A
EHMT1		transcription regulator			0.0423	NANOG
TFAP2C		transcription regulator			0.00964	JAG1,NANOG,THBS1
tetramethylpyrazine		chemical - endogenous non-mammalian			0.00718	CXCR4
ERF		transcription regulator			0.0423	UTRN
ELK1		transcription regulator			0.0062	MECOM,NUPR1,THBS1
CLCA1		ion channel			0.0143	MUC5AC
CTNNB1		transcription regulator			0.0355	CEACAM1,CXCL12,EDIL3,GPX2,ID4,MECOM,NANOG,S100A4,TCF7L2,TNC
RPL35A		other			0.0284	PDCD4
YY2		transcription regulator			0.0354	CXCR4
EEF1A1		translation regulator			0.0214	S100A4
GZMB		peptidase			0.0284	ENTPD1
CALCA		other			0.0286	CALCR,CXCR4,FLT1
RPLP2		other			0.0143	PDCD4
Gm21596/Hmgb1		transcription regulator			0.00808	CXCL12,CXCR4
Irgm1		other			0.0382	NUPR1,SPAG5
Hrg		other			0.0423	ANGPT2
Foxe3		transcription regulator			0.0354	PROX1
Muc1		transmembrane receptor			0.0354	MUC5AC
Naip1 (includes others)		other			0.0143	MUC5AC
Nedd4		enzyme			0.00718	THBS1
RPL12		other			0.0284	PDCD4
OBP2B		transporter			0.0423	MLXIPL
CLEC2D		transmembrane receptor			0.0143	CXCR4
PRR13		other			0.00718	THBS1
SLC19A1		transporter			0.0492	CALCR
SRSF2		transcription regulator			0.00467	ABCC9,HNRNPL,MLXIPL
SPINT1		other			0.00506	PROX1,WNT5A
ATF2		transcription regulator			0.0279	ENTPD1,NUPR1,TGFB2
WT1		transcription regulator			0.000314	CLIC4,GRB10,HNRNPL,ITGB1BP1,THBS1,TM4SF1,TSPAN5,VDR
HOXA7		transcription regulator			0.0183	CALCRL,EDNRA
PROX1	-2.2	transcription regulator			0.0149	ANGPT2,LYVE1
RAD21		transcription regulator			0.0221	CXCL12,THBS1
ECM1		transporter			0.00506	NANOG,S100A4
PMX 205		chemical reagent			0.0214	THBS1

FTL		enzyme			0.0214	TFRC
RPS12		other			0.0354	PDCD4
CHCHD2		other			0.0354	NANOG
RPS15		other			0.0354	PDCD4
N-butyryl arginine		chemical reagent			0.0214	UTRN
3-hydroxybutyrate arginate		chemical reagent			0.0214	UTRN
1-(2-hydroxy-5-methylphenyl)-3-phenyl-1,3-propanedione		chemical reagent			0.0284	CREB1
gentamicin C		chemical drug			0.0105	C4A/C4B,GPX2,TES
4-[(4'-chloro-2'-fluoro)phenylamino]-6,7-dimethoxyquinazoline		chemical - kinase inhibitor			0.0143	ANGPT2
chloropromazine		chemical drug			0.0366	CYP4F2,MSMO1
halofuginone		chemical drug			0.00303	C4A/C4B,EMP1,LTBP1,MLXIPL,WNT5A
pyridoxal phosphate-6-azophenyl-2',4'-disulfonic acid		chemical reagent			0.0354	THBS1
cyclophosphamide		chemical drug			0.0162	C4A/C4B,GPX2,THBS1
acrolein		chemical toxicant			0.00894	ADAM10,MUC5AC
zoledronic acid		chemical drug			0.00184	CXCR4,FLT1,WNT5A
LG100268		chemical reagent			0.0334	CYP4F2,CYP4F3
4-(2-aminoethyl)benzenesulfonylfluoride		chemical - protease inhibitor			0.0195	SENP7,TNC
tacrolimus		chemical drug			0.0134	CHN2,CXCR4,PDCD4,PRKACB,TNC
BMS-275183		chemical drug			0.00718	THBS1
phosphorylethanolamine		chemical - endogenous mammalian			0.0143	FLT1
enalapril		biologic drug			0.0062	ANGPT2,C4A/C4B,GPX2
methimazole		chemical drug			0.0415	TG,VLDLR
GW501516		chemical drug			0.0372	FLT1,THBS1,VLDLR
acetazolamide		chemical drug			0.0423	SLC26A4
cilomilast		chemical drug			0.0214	CXCR4
rolipram		chemical drug			0.0195	CXCR4,MUC5AC
omeprazole		chemical drug			0.0466	CXCL12,CXCR4
N-ethylmaleimide		chemical reagent			0.0214	LEPR
plerixafor		chemical drug			0.00894	CXCL12,CXCR4
cibacron blue F 3GA		chemical reagent			0.0214	THBS1
2-acetylaminofluorene		chemical toxicant			0.00575	DLK1,JAG1
fenamic acid		chemical reagent			0.0135	C4A/C4B,GPX2,TES
liarozole		chemical drug			0.0284	VDR
dinophysistoxin 1		chemical toxicant			0.0143	CEACAM1
BIBX1522		chemical - kinase inhibitor			0.0143	MUC5AC

thalidomide		chemical drug			0.0261	ANGPT2,KCNMA1
captopril		chemical drug			0.0174	C4A/C4B,GPX2,TGFB2
dimethyl sulfoxide		chemical drug			0.0137	DNAJB4,NUPR1,TFRC,VLDLR
GW3965		chemical reagent			0.0162	EDNRA,MLXIPL,VLDLR
thioacetamide		chemical toxicant			0.000425	C4A/C4B,EMP1,GPX2,LTBP1,LYVE1,MLXIPL,TES,WNT5A
hexaarginine-neomycin B conjugate		chemical reagent			0.0143	CXCR4
histone deacetylase		complex			0.0319	CXCR4,THBS1
minodronate		chemical drug			0.0214	CXCR4
retinoid		chemical drug			0.0382	CEACAM1,MUC5AC
triamterene		chemical drug			0.0119	C4A/C4B,GPX2,TES
phenacetin		chemical drug			0.0366	GPX2,TES
SB-431542		chemical reagent			0.0319	CLIC4,NANOG,TGFB2
NSC 651016		chemical reagent			0.0143	CXCR4
nabumetone		chemical drug			0.00718	UTRN
etodolac		chemical drug			0.0214	THBS1
semaxinib		chemical drug			0.0234	FHL1,THBS1
trichosanthin		chemical drug			0.0143	JAG1
H-7		chemical - kinase inhibitor			0.035	ANGPT2,VDR
nisoldipine		chemical drug			0.0143	CREB1
4-tert-octylphenol		chemical toxicant			0.0208	EMP1,TANK
propyl gallate		chemical toxicant			0.0423	MUC5AC
tert-butyl-hydroquinone		chemical reagent			0.0241	GPX2,NEFH,WNT5A
nitroprusside		chemical drug			0.0483	CREB1,FLT1
cobalt chloride		chemical reagent			0.0168	CXCR4,FLT1,TFRC
potassium perchlorate		chemical reagent			0.0354	TG
retinol		chemical drug			0.00727	NANOG,TGFB2
tamibarotene		chemical drug			0.00983	CEACAM1,LEPR
[Ac-His1,D-Phe2,Lys15,Arg16,Leu27] VIP-(3-7)-GRF-(8-27)		chemical reagent			0.0423	CREB1
vancomycin		biologic drug			0.0241	C4A/C4B,GPX2,TES
microcystin		chemical toxicant			0.0143	CREB1
Phe-Pro-Arg-chloromethyl ketone		chemical - protease inhibitor			0.0214	FLT1
N-Ac-leucyl-leucyl-methioninal		chemical - protease inhibitor			0.0492	CREB1
TAPI-1		chemical - protease inhibitor			0.00222	LEPR,MUC5AC
androgen		chemical drug			0.0331	ALDH6A1,C4A/C4B,CXCR4,WNT5A
ursolic acid		chemical drug			0.000912	CXCR4,MUC5AC,PDCD4,SLC38A2
monocrotaline		chemical toxicant			0.0334	FHL1,SMAD5
camptothecin		chemical drug			0.012	CREB1,FGL2,FLT1,GRB10,MBNL2,PLCL2,PTGER2,TFRC,TLK1,TPM4
tiotropium		chemical drug			0.0284	MUC5AC
clofibrate		chemical drug			0.0345	CEACAM1,PDCD4,SLCO2A1

epinephrine		chemical - endogenous mammalian			0.0174	CXCR4,DLK1,TFRC
tyrphostin AG 1478		chemical - kinase inhibitor			0.014	CXCR4,MUC5AC,PTGER2
prostanoid		chemical drug			0.00718	ENTPD1
P1,P4-Di(adenosine-5') tetraphosphate		chemical - endogenous mammalian			0.00139	TGFB2,THBS1
zalcitabine		chemical drug			0.0284	CXCL12
gallium nitrate		chemical drug			0.0423	CALCR
honokiol		chemical - endogenous non-mammalian			0.0247	FLT1,IQGAP1
melphalan		chemical drug			0.00727	ADAM10,FLT1
arginine butyrate		chemical drug			0.0214	UTRN
galactose		chemical - endogenous mammalian			0.0044	THBS1,VDR
D-fructose		chemical - endogenous mammalian			0.0366	MLXIPL,THBS1
lactose		chemical - endogenous mammalian			0.0354	VDR
phorbol 12,13-didecanoate		chemical toxicant			0.0171	CALCR,HSF2
25-hydroxycholesterol		chemical reagent			0.011	CYP4F2,EIF2S1,VLDLR
steroid hormone		chemical - other			0.0284	CXCR4
dihydrotestosterone		chemical - endogenous mammalian			0.0407	C4A/C4B,JAG1,LTBP1,MCAM,PDCD4,TGFB2,THBS1,VLDLR
finasteride		chemical drug			0.00894	FLT1,TGFB2
arsenite		chemical toxicant			0.0429	SLC38A2,TGFB2,THBS1

Table J: Time dependent changes in gene expression in TAC + EVR Group shown in Figure 8

© 2000-2019 QIAGEN. All rights reserved.

Expr Fold Change	Expr p-value	Expr False Discovery Rate (q-value)	ID	Flags	Symbol	Entrez Gene Name	Location	Type(s)	Drug(s)
-1.51	0.0215	0.921	202852_s_at		AAGAB	alpha and gamma adaptin binding protein	Cytoplasm	other	
2.06	0.0205	0.921	217504_at		ABCA6	ATP binding cassette subfamily A member 6	Plasma Membrane	transporter	
1.64	0.0265	0.921	204719_at		ABCA8	ATP binding cassette subfamily A member 8	Plasma Membrane	transporter	
1.66	0.0479	0.921	218322_s_at		ACSL5	acyl-CoA synthetase long chain family member 5	Cytoplasm	enzyme	
-1.58	0.0434	0.921	200727_s_at		ACTR2	ARP2 actin related protein 2 homolog	Plasma Membrane	other	
-1.7	0.0381	0.921	208223_s_at		ACVR1B	activin A receptor type 1B	Plasma Membrane	kinase	
1.51	0.0179	0.921	209765_at		ADAM19	ADAM metallopeptidase domain 19	Plasma Membrane	peptidase	
2.63	0.0462	0.921	203741_s_at		ADCY7	adenylate cyclase 7	Plasma Membrane	enzyme	
2.33	0.0123	0.921	212980_at		AHSA2P	activator of HSP90 ATPase homolog 2, pseudogene	Other	other	
1.6	0.0188	0.921	205583_s_at		ALG13	ALG13, UDP-N-acetylglucosaminyltransferase subunit	Cytoplasm	enzyme	
1.87	0.0264	0.921	208498_s_at		AMY1C (includes others)	amylase, alpha 1C (salivary)	Extracellular Space	enzyme	
1.5	0.0132	0.921	214723_x_at		ANKRD36	ankyrin repeat domain 36	Other	other	
1.54	0.0225	0.921	204672_s_at		ANKRD6	ankyrin repeat domain 6	Nucleus	transcription regulator	
1.77	0.04	0.921	201012_at		ANXA1	annexin A1	Plasma Membrane	enzyme	hydrocortisone, hydrocortisone/prednisone, hydrocortisone/mitoxantrone
-1.61	0.0452	0.921	222269_at		APOOL	apolipoprotein O like	Extracellular Space	other	
1.54	0.0491	0.921	218067_s_at		ARGLU1	arginine and glutamate rich 1	Other	other	
-1.64	0.00605	0.921	211611_s_at		ATF6B	activating transcription factor 6 beta	Nucleus	transcription regulator	
2.15	0.0436	0.921	210538_s_at		BIRC3	baculoviral IAP repeat containing 3	Cytoplasm	enzyme	birinapant
1.61	0.02	0.921	217179_x_at		BMS1P20	BMS1, ribosome biogenesis factor pseudogene 20	Other	other	
1.6	0.00567	0.921	209770_at		BTN3A1	butyrophilin subfamily 3 member A1	Extracellular Space	other	

Table J: Time dependent changes in gene expression in TAC + EVR Group shown in Figure 8

1.7	0.0419	0.921	212613_at		BTN3A2	butyrophilin subfamily 3 member A2	Plasma Membrane	other	
1.75	0.0112	0.921	204820_s_at		BTN3A3	butyrophilin subfamily 3 member A3	Plasma Membrane	other	
1.55	0.0336	0.921	212077_at		CALD1	caldesmon 1	Cytoplasm	other	
-1.75	0.0415	0.921	202966_at		CAPN6	calpain 6	Cytoplasm	peptidase	
1.96	0.0362	0.921	211368_s_at	D	CASP1	caspase 1	Cytoplasm	peptidase	caspase 1 inhibitor
1.66	0.0394	0.921	211366_x_at	D	CASP1	caspase 1	Cytoplasm	peptidase	caspase 1 inhibitor
4.19	0.00111	0.921	210072_at		CCL19	C-C motif chemokine ligand 19	Extracellular Space	cytokine	
1.6	0.0244	0.921	207794_at		CCR2	C-C motif chemokine receptor 2	Plasma Membrane	G-protein coupled receptor	PF-4136309, MLN1202, BMS-813160
1.86	0.0483	0.921	206991_s_at		CCR5	C-C motif chemokine receptor 5 (gene/pseudogene)	Plasma Membrane	G-protein coupled receptor	maraviroc, vicriviroc, ancriviroc, BMS-813160
1.56	0.0451	0.921	210031_at		CD247	CD247 molecule	Plasma Membrane	transmembrane receptor	blinatumomab
1.58	0.046	0.921	209933_s_at		CD300A	CD300a molecule	Plasma Membrane	transmembrane receptor	
2.44	0.0465	0.921	204118_at		CD48	CD48 molecule	Plasma Membrane	other	
-1.79	0.0238	0.921	208727_s_at		CDC42	cell division cycle 42	Cytoplasm	enzyme	
2.91	0.0457	0.921	202158_s_at		CELF2	CUGBP Elav-like family member 2	Nucleus	other	
1.53	0.0273	0.921	219644_at		CEP83	centrosomal protein 83	Extracellular Space	other	
2.74	0.0274	0.921	217148_x_at		CKAP2	cytoskeleton associated protein 2	Cytoplasm	other	
1.76	0.0398	0.921	213415_at		CLIC2	chloride intracellular channel 2	Cytoplasm	enzyme	
1.97	0.00953	0.921	205518_s_at		CMAHP	cytidine monophospho-N-acetylneuraminic acid hydroxylase, pseudogene	Cytoplasm	other	
1.73	0.0105	0.921	211038_s_at		CROCCP2	ciliary rootlet coiled-coil, rootletin pseudogene 2	Other	other	
1.9	0.0419	0.921	210140_at		CST7	cystatin F	Extracellular Space	other	
1.9	0.0264	0.921	209975_at		CYP2E1	cytochrome P450 family 2 subfamily E member 1	Cytoplasm	enzyme	
-1.55	0.0411	0.921	215058_at		DENND5B	DENN domain containing 5B	Cytoplasm	other	
1.57	0.0228	0.921	201697_s_at		DNMT1	DNA methyltransferase 1	Nucleus	enzyme	5-azacytidine/decitabine, 5-azacytidine/cytarabine/decitabine, 5-azacytidine/sorafenib, decitabine/sorafenib, cladribine/cytarabine/decitabine, 4'-thio-2'-deoxycytidine, 5-azacytidine/lenalidomide, fluorocyclopentenylcytosine, decitabine/imatinib, 5-azacytidine, decitabine
2.59	0.00651	0.921	213160_at		DOCK2	dedicator of cytokinesis 2	Cytoplasm	other	

Table J: Time dependent changes in gene expression in TAC + EVR Group shown in Figure 8

1.7	0.0339	0.921	205003_at		DOCK4	dedicator of cytokinesis 4	Plasma Membrane	other	
1.51	0.0466	0.921	212538_at		DOCK9	dedicator of cytokinesis 9	Cytoplasm	other	
1.67	0.00757	0.921	201843_s_at		EFEMP1	EGF containing fibulin extracellular matrix protein 1	Extracellular Space	enzyme	
1.58	0.00187	0.921	220349_s_at		ENGASE	endo-beta-N-acetylglucosaminidase	Cytoplasm	enzyme	
1.53	0.0463	0.921	201719_s_at		EPB41L2	erythrocyte membrane protein band 4.1 like 2	Plasma Membrane	other	
1.85	0.0365	0.921	211742_s_at		EVI2B	ecotropic viral integration site 2B	Plasma Membrane	other	
3.57	0.0045	0.921	210889_s_at		FCGR2B	Fc fragment of IgG receptor IIb	Plasma Membrane	transmembrane receptor	IgG, BI-1206, XmAb5871
1.56	0.0119	0.921	221601_s_at		FCMR	Fc fragment of IgM receptor	Plasma Membrane	other	
1.54	0.0223	0.921	205782_at		FGF7	fibroblast growth factor 7	Extracellular Space	growth factor	
1.64	0.00312	0.921	212288_at		FNBP1	formin binding protein 1	Nucleus	enzyme	
1.74	0.00749	0.921	212232_at		FNBP4	formin binding protein 4	Nucleus	other	
2.12	0.042	0.921	211795_s_at		FYB1	FYN binding protein 1	Nucleus	other	
1.56	0.0217	0.921	203146_s_at	D	GABBR1	gamma-aminobutyric acid type B receptor subunit 1	Plasma Membrane	G-protein coupled receptor	arbaclofen placarbil, arbaclofen, baclofen, vigabatrin
2.71	0.0258	0.921	205890_s_at	D	GABBR1	gamma-aminobutyric acid type B receptor subunit 1	Plasma Membrane	G-protein coupled receptor	arbaclofen placarbil, arbaclofen, baclofen, vigabatrin
2.57	0.0331	0.921	204222_s_at		GLIPR1	GLI pathogenesis related 1	Extracellular Space	other	
2.65	0.00692	0.921	208798_x_at	D	GOLGA8A/GOLGA8B	golgin A8 family member A	Cytoplasm	other	
1.7	0.00935	0.921	210424_s_at	D	GOLGA8A/GOLGA8B	golgin A8 family member A	Cytoplasm	other	
1.77	0.0232	0.921	210425_x_at	D	GOLGA8A/GOLGA8B	golgin A8 family member A	Cytoplasm	other	
1.51	0.0077	0.921	204793_at		GPRASP1	G protein-coupled receptor associated sorting protein 1	Cytoplasm	transporter	
3.9	0.0347	0.921	205488_at		GZMA	granzyme A	Cytoplasm	peptidase	
3.65	0.0201	0.921	206666_at		GZMK	granzyme K	Cytoplasm	peptidase	
2.3	0.00698	0.921	217317_s_at		HERC2P2	hect domain and RLD 2 pseudogene 2	Other	other	
-1.51	0.00456	0.921	217427_s_at		HIRA	histone cell cycle regulator	Nucleus	transcription regulator	
-1.6	0.0332	0.921	201055_s_at		HNRNPA0	heterogeneous nuclear ribonucleoprotein A0	Nucleus	other	
1.51	0.0279	0.921	222040_at		HNRNPA1	heterogeneous nuclear ribonucleoprotein A1	Nucleus	enzyme	
2.16	0.00814	0.921	213359_at		HNRNPD	heterogeneous nuclear ribonucleoprotein D	Nucleus	transcription regulator	

Table J: Time dependent changes in gene expression in TAC + EVR Group shown in Figure 8

1.61	0.0374	0.921	212454_x_at		HNRNPDL	heterogeneous nuclear ribonucleoprotein D like	Nucleus	other	
1.8	0.0166	0.921	221860_at		HNRNPL	heterogeneous nuclear ribonucleoprotein L	Nucleus	other	
1.79	0.0125	0.921	213418_at		HSPA6	heat shock protein family A (Hsp70) member 6	Nucleus	enzyme	
1.77	0.0201	0.921	210029_at		IDO1	indoleamine 2,3-dioxygenase 1	Cytoplasm	enzyme	1-methyl-D-tryptophan, epacadostat, navaximod, KHK2455, PF-06840003, BMS-986205, LY3381916
2.06	0.024	0.921	208965_s_at		IFI16	interferon gamma inducible protein 16	Nucleus	transcription regulator	
3.02	0.015	0.921	214059_at	D	IFI44	interferon induced protein 44	Cytoplasm	other	
2.44	0.0167	0.921	214453_s_at	D	IFI44	interferon induced protein 44	Cytoplasm	other	
-1.65	0.0163	0.921	203628_at		IGF1R	insulin like growth factor 1 receptor	Plasma Membrane	transmembrane receptor	picropodophyllin, linsitinib, cixutumumab, ganitumab, AVE1642, BMS-754807, XL228, BIIB022, IGF1, dalotuzumab, ceritinib, brigatinib, [I-124]-CPD-1028, MM-141, IGF-methotrexate conjugate, PL225B, KW-2450, IGF-1R inhibitor
11.4	0.0456	0.921	217022_s_at		IGHA1	immunoglobulin heavy constant alpha 1	Extracellular Space	other	
3.15	0.0276	0.921	209374_s_at		IGHM	immunoglobulin heavy constant mu	Plasma Membrane	transmembrane receptor	
2.96	0.029	0.921	221671_x_at	D	IGKC	immunoglobulin kappa constant	Extracellular Space	other	
2.9	0.0308	0.921	221651_x_at	D	IGKC	immunoglobulin kappa constant	Extracellular Space	other	
1.57	0.0326	0.921	211881_x_at	D	IGLJ3	immunoglobulin lambda joining 3	Other	other	
7.38	0.0399	0.921	209138_x_at	D	IGLJ3	immunoglobulin lambda joining 3	Other	other	
1.58	0.0121	0.921	209827_s_at		IL16	interleukin 16	Extracellular Space	cytokine	
1.66	0.00664	0.921	222062_at		IL27RA	interleukin 27 receptor subunit alpha	Plasma Membrane	transmembrane receptor	
2.01	0.0323	0.921	209821_at		IL33	interleukin 33	Extracellular Space	cytokine	
2.26	0.0301	0.921	213817_at		IRAK3	interleukin 1 receptor associated kinase 3	Cytoplasm	kinase	
1.57	0.0455	0.921	204057_at		IRF8	interferon regulatory factor 8	Nucleus	transcription regulator	
1.63	0.0263	0.921	204698_at		ISG20	interferon stimulated exonuclease gene 20	Nucleus	enzyme	
1.92	0.0443	0.921	205884_at		ITGA4	integrin subunit alpha 4	Plasma Membrane	transmembrane receptor	natalizumab, vedolizumab
2.02	0.0446	0.921	211339_s_at		ITK	IL2 inducible T cell kinase	Cytoplasm	kinase	pazopanib, crizotinib/pazopanib
4.02	0.00822	0.921	212592_at		JCHAIN	joining chain of multimeric IgA and IgM	Extracellular Space	other	

Table J: Time dependent changes in gene expression in TAC + EVR Group shown in Figure 8

1.53	0.00554	0.921	221889_at		KCTD13	potassium channel tetramerization domain containing 13	Nucleus	ion channel	
1.62	0.0428	0.921	210644_s_at		LAIR1	leukocyte associated immunoglobulin like receptor 1	Plasma Membrane	transmembrane receptor	
1.69	0.0193	0.921	208885_at		LCP1	lymphocyte cytosolic protein 1	Cytoplasm	other	
2.13	0.00402	0.921	213703_at		LINC00342	long intergenic non-protein coding RNA 342	Other	other	
1.81	0.0177	0.921	221973_at		LOC100506076	uncharacterized LOC100506076	Other	other	
1.65	0.0406	0.921	209360_s_at		LOC100506403	uncharacterized LOC100506403	Other	other	
1.71	0.0269	0.921	214181_x_at		LST1	leukocyte specific transcript 1	Plasma Membrane	other	
-1.94	0.0321	0.921	210596_at		MAGT1	magnesium transporter 1	Plasma Membrane	enzyme	
1.57	0.0195	0.921	214786_at		MAP3K1	mitogen-activated protein kinase kinase kinase 1	Cytoplasm	kinase	E 6201
1.84	0.0172	0.921	218181_s_at		MAP4K4	mitogen-activated protein kinase kinase kinase kinase 4	Cytoplasm	kinase	
2.86	0.0194	0.921	219574_at		MARCH1	membrane associated ring-CH-type finger 1	Cytoplasm	enzyme	
-2.55	0.0317	0.921	219730_at		MED18	mediator complex subunit 18	Nucleus	other	
1.56	0.0284	0.921	210794_s_at		MEG3	maternally expressed 3	Other	other	
1.53	0.00551	0.921	212472_at		MICAL2	microtubule associated monooxygenase, calponin and LIM domain containing 2	Cytoplasm	enzyme	
1.51	0.0335	0.921	205408_at		MLLT10	MLLT10, histone lysine methyltransferase DOT1L cofactor	Nucleus	transcription regulator	
3.34	0.0352	0.921	204959_at		MNDA	myeloid cell nuclear differentiation antigen	Nucleus	other	
1.59	0.0249	0.921	221208_s_at		MSANTD2	Myb/SANT DNA binding domain containing 2	Other	other	
-1.79	0.0256	0.921	214765_s_at		NAAA	N-acylethanolamine acid amidase	Cytoplasm	enzyme	
1.64	0.02	0.921	218896_s_at		NCBP3	nuclear cap binding subunit 3	Nucleus	other	
1.56	0.0283	0.921	207677_s_at		NCF4	neutrophil cytosolic factor 4	Cytoplasm	enzyme	
1.69	0.0469	0.921	209734_at		NCKAP1L	NCK associated protein 1 like	Plasma Membrane	other	

Table J: Time dependent changes in gene expression in TAC + EVR Group shown in Figure 8

1.66	0.0436	0.921	214241_at		NDUFB8	NADH:ubiquinone oxidoreductase subunit B8	Cytoplasm	enzyme	
1.73	0.0347	0.921	213915_at		NKG7	natural killer cell granule protein 7	Plasma Membrane	other	
3.09	0.0348	0.921	202237_at		NNMT	nicotinamide N-methyltransferase	Cytoplasm	enzyme	atorvastatin/niacin, nicotinic acid/pioglitazone, nicotinic acid, lovastatin/niacin
2.04	0.00372	0.921	212307_s_at		OGT	O-linked N-acetylglucosamine (GlcNAc) transferase	Cytoplasm	enzyme	
1.75	0.00649	0.921	213125_at		OLFML2B	olfactomedin like 2B	Extracellular Space	other	
1.74	0.0264	0.921	209627_s_at		OSBPL3	oxysterol binding protein like 3	Cytoplasm	transporter	
1.84	0.028	0.921	220005_at		P2RY13	purinergic receptor P2Y13	Plasma Membrane	G-protein coupled receptor	
1.59	0.0324	0.921	218543_s_at		PARP12	poly(ADP-ribose) polymerase family member 12	Nucleus	other	
-1.62	0.00493	0.921	204213_at		PIGR	polymeric immunoglobulin receptor	Plasma Membrane	transporter	
1.5	0.0298	0.921	203895_at	D	PLCB4	phospholipase C beta 4	Cytoplasm	enzyme	
1.74	0.0336	0.921	203896_s_at	D	PLCB4	phospholipase C beta 4	Cytoplasm	enzyme	
1.8	0.0159	0.921	213241_at		PLXNC1	plexin C1	Plasma Membrane	transmembrane receptor	
1.72	0.0032	0.921	212176_at	D	PNISR	PNN interacting serine and arginine rich protein	Nucleus	other	
1.76	0.0189	0.921	212179_at	D	PNISR	PNN interacting serine and arginine rich protein	Nucleus	other	
1.83	0.0253	0.921	209598_at		PNMA2	PNMA family member 2	Nucleus	transporter	
-1.7	0.0308	0.921	209739_s_at		PNPLA4	patatin like phospholipase domain containing 4	Cytoplasm	enzyme	
1.5	0.0138	0.921	214617_at		PRF1	perforin 1	Cytoplasm	transporter	
1.68	0.0385	0.921	216069_at		PRMT2	protein arginine methyltransferase 2	Nucleus	enzyme	
1.51	0.0156	0.921	202127_at		PRPF4B	pre-mRNA processing factor 4B	Nucleus	kinase	
-1.63	0.00922	0.921	205171_at		PTPN4	protein tyrosine phosphatase, non-receptor type 4	Cytoplasm	phosphatase	
2.61	0.0225	0.921	207238_s_at	D	PTPRC	protein tyrosine phosphatase, receptor type C	Plasma Membrane	phosphatase	111 In-BC8
2.45	0.0239	0.921	212588_at	D	PTPRC	protein tyrosine phosphatase, receptor type C	Plasma Membrane	phosphatase	111 In-BC8

Table J: Time dependent changes in gene expression in TAC + EVR Group shown in Figure 8

3.1	0.035	0.921	212587_s_at	D	PTPRC	protein tyrosine phosphatase, receptor type C	Plasma Membrane	phosphatase	111 In-BC8
2.03	0.0423	0.921	213603_s_at		RAC2	Rac family small GTPase 2	Cytoplasm	enzyme	
1.51	0.0373	0.921	205647_at		RAD52	RAD52 homolog, DNA repair protein	Nucleus	other	
1.57	0.00385	0.921	212033_at		RBM25	RNA binding motif protein 25	Nucleus	other	
-1.91	0.0357	0.921	213663_s_at		RETREG1	reticulophagy regulator 1	Cytoplasm	other	
1.75	0.0137	0.921	209007_s_at		RSRP1	arginine and serine rich protein 1	Other	other	
1.79	0.019	0.921	218793_s_at		SCML1	Scm polycomb group protein like 1	Nucleus	transcription regulator	
-1.51	0.00258	0.921	215088_s_at		SDHC	succinate dehydrogenase complex subunit C	Cytoplasm	enzyme	
-1.54	0.0301	0.921	205405_at		SEMA5A	semaphorin 5A	Plasma Membrane	transmembrane receptor	
-1.71	0.00387	0.921	213048_s_at		SET	SET nuclear proto-oncogene	Nucleus	phosphatase	
2.24	0.0193	0.921	221768_at	D	SFPQ	splicing factor proline and glutamine rich	Nucleus	transcription regulator	
1.87	0.0258	0.921	214016_s_at	D	SFPQ	splicing factor proline and glutamine rich	Nucleus	transcription regulator	
1.55	0.00783	0.921	44673_at		SIGLEC1	sialic acid binding Ig like lectin 1	Plasma Membrane	other	
1.76	0.0468	0.921	219159_s_at		SLAMF7	SLAM family member 7	Plasma Membrane	other	elotuzumab
1.56	0.0378	0.921	212810_s_at		SLC1A4	solute carrier family 1 member 4	Plasma Membrane	transporter	riluzole
-1.77	0.0155	0.921	207429_at		SLC22A2	solute carrier family 22 member 2	Plasma Membrane	transporter	
2.08	0.0432	0.921	205421_at		SLC22A3	solute carrier family 22 member 3	Plasma Membrane	transporter	
1.88	0.0366	0.921	221024_s_at		SLC2A10	solute carrier family 2 member 10	Plasma Membrane	transporter	
-1.89	0.0443	0.921	202667_s_at		SLC39A7	solute carrier family 39 member 7	Plasma Membrane	transporter	
1.94	0.0164	0.921	200795_at		SPARCL1	SPARC like 1	Extracellular Space	other	
-1.52	0.0331	0.921	203128_at		SPTLC2	serine palmitoyltransferase long chain base subunit 2	Cytoplasm	enzyme	
-1.54	0.0262	0.921	208991_at		STAT3	signal transducer and activator of transcription 3	Nucleus	transcription regulator	golotimod, OPB-31121, OPB-51602, STAT3 inhibitor XIII, danvatirsen, TTI-101
1.73	0.0151	0.921	206118_at		STAT4	signal transducer and activator of transcription 4	Nucleus	transcription regulator	
-1.51	0.048	0.921	209622_at		STK16	serine/threonine kinase 16	Cytoplasm	kinase	
1.86	0.024	0.921	212344_at	D	SULF1	sulfatase 1	Cytoplasm	enzyme	

1.53	0.0298	0.921	212354_at	D	SULF1	sulfatase 1	Cytoplasm	enzyme	
1.73	0.0458	0.921	212353_at	D	SULF1	sulfatase 1	Cytoplasm	enzyme	
1.65	0.0323	0.921	219310_at		SYNDIG1	synapse differentiation inducing 1	Plasma Membrane	other	
2.18	0.0107	0.921	219563_at		SYNE3	spectrin repeat containing nuclear envelope family member 3	Nucleus	other	
1.86	0.0188	0.921	209403_at		TBC1D3 (includes others)	TBC1 domain family member 3F	Extracellular Space	other	
1.54	0.00781	0.921	201108_s_at		THBS1	thrombospondin 1	Extracellular Space	other	
1.7	0.0342	0.921	207571_x_at		THEMIS2	thymocyte selection associated family member 2	Other	other	
1.72	0.00785	0.921	201448_at		TIA1	TIA1 cytotoxic granule associated RNA binding protein	Nucleus	other	
1.6	0.023	0.921	210176_at		TLR1	toll like receptor 1	Plasma Membrane	transmembrane receptor	
1.53	0.0106	0.921	202643_s_at		TNFAIP3	TNF alpha induced protein 3	Nucleus	enzyme	
1.81	0.04	0.921	203508_at		TNFRSF1B	TNF receptor superfamily member 1B	Plasma Membrane	transmembrane receptor	
1.6	0.0359	0.921	219423_x_at		TNFRSF25	TNF receptor superfamily member 25	Plasma Membrane	transmembrane receptor	
1.51	0.0486	0.921	208609_s_at		TNXB	tenascin XB	Extracellular Space	other	
2.13	0.0189	0.921	213193_x_at	D	TRBC1	T cell receptor beta constant 1	Plasma Membrane	other	
2.69	0.0268	0.921	210915_x_at	D	TRBC1	T cell receptor beta constant 1	Plasma Membrane	other	
1.87	0.0116	0.921	213293_s_at		TRIM22	tripartite motif containing 22	Cytoplasm	transcription regulator	
-1.62	0.0479	0.921	208589_at		TRPC7	transient receptor potential cation channel subfamily C member 7	Plasma Membrane	ion channel	
1.64	0.00168	0.921	213510_x_at		USP32P2	ubiquitin specific peptidase 32 pseudogene 2	Other	other	
1.52	0.0471	0.921	221213_s_at		ZNF280D	zinc finger protein 280D	Other	other	
1.83	0.0115	0.921	215985_at		ZNRD1ASP	zinc ribbon domain containing 1 antisense, pseudogene	Other	other	

Table K: Time dependent changes in gene expression in TAC + MMF Group shown in Figure 8

© 2000-2019 QIAGEN. All rights reserved.

Expr Fold Change	Expr p-value	Expr False Discovery Rate (q-value)	ID	Flags	Symbol	Entrez Gene Name	Location	Type(s)	Drug(s)
1.53	0.0218	0.199	208561_at		ABCC9	ATP binding cassette subfamily C member 9	Plasma Membrane	ion channel	
-1.69	0.0417	0.244	45288_at		ABHD6	abhydrolase domain containing 6	Cytoplasm	enzyme	
-2.12	0.0396	0.24	205730_s_at		ABLIM3	actin binding LIM protein family member 3	Cytoplasm	other	
-1.59	0.0433	0.249	207071_s_at		ACO1	aconitase 1	Cytoplasm	enzyme	
-3.36	0.0188	0.191	204638_at		ACP5	acid phosphatase 5, tartrate resistant	Cytoplasm	phosphatase	
2.32	0.00368	0.133	214895_s_at	D	ADAM10	ADAM metallopeptidase domain 10	Plasma Membrane	peptidase	
1.87	0.0111	0.166	202604_x_at	D	ADAM10	ADAM metallopeptidase domain 10	Plasma Membrane	peptidase	
1.51	0.0127	0.171	213974_at		ADAMTSL3	ADAMTS like 3	Other	other	
1.69	0.0044	0.137	203865_s_at		ADARB1	adenosine deaminase, RNA specific B1	Nucleus	enzyme	
2.16	0.0244	0.206	209867_s_at		ADGRL3	adhesion G protein-coupled receptor L3	Plasma Membrane	G-protein coupled receptor	
1.78	0.0315	0.223	203571_s_at		ADIRF	adipogenesis regulatory factor	Nucleus	other	
1.66	0.0123	0.17	209869_at		ADRA2A	adrenoceptor alpha 2A	Plasma Membrane	G-protein coupled receptor	paliperidone, risperidone, antazoline/naphazoline, acetaminophen/clemastine/pseudoephedrine, articaine/epinephrine, bupivacaine/epinephrine, caffeine/ergotamine, acetaminophen/dexbrompheniramine/pseudoephedrine, dapiprazole, dexbrompheniramine/pseudoephedrine, chlorpheniramine/ibuprofen/pseudoephedrine, dipivefrin, cetirizine/pseudoephedrine, asenapine, epinephrine/prilocaine, epinephrine/lidocaine, myogane, V2006, lurasidone, lofexidine, dextromethorphan/quinidine, hydrocodone/pseudoephedrine, paliperidone palmitate, xylometazoline, brimonidine/brinzolamide, epinephrine/methotrexate, fexofenadine/pseudoephedrine, loratadine/pseudoephedrine, oxymetazoline, prazosin, methamphetamine, phenylpropanolamine, ephedrine, tolazoline, guanfacine, guanabenz, guanethidine, phenoxybenzamine, dexmedetomidine, ibuprofen/pseudoephedrine, brimonidine, clonidine, cirazoline, dexefaroxan, quinidine, polythiazide/prazosin, chlorothiazide/methyldopa, chlorthalidone/clonidine, propafenone, guanadrel, hydrochlorothiazide/methyldopa, naproxen/pseudoephedrine, periciazine, metaraminol, tizanidine, quetiapine, D-pseudoephedrine, apraclonidine, benzphetamine, mirtazapine, venlafaxine, phentolamine, labetalol, mephentermine, propylhexedrine, yohimbine, dihydroergotamine, ergotamine, norepinephrine, alpha-methyl dopa, epinephrine, droxidopa, dopamine, chlorpheniramine/phenylpropanolamine, desloratadine/pseudoephedrine, acrivastine/pseudoephedrine, carbinoxamine/pseudoephedrine, brompheniramine/codeine/phenylpropanolamine, pseudoephedrine/triprolidine, codeine/pseudoephedrine/triprolidine,

1.73	0.00211	0.121	209535_s_at		AKAP13	A-kinase anchoring protein 13	Cytoplasm	other	
-1.59	0.0206	0.197	214259_s_at		AKR7A2	aldo-keto reductase family 7 member A2	Cytoplasm	enzyme	
-2.28	0.0474	0.258	211552_s_at		ALDH4A1	aldehyde dehydrogenase 4 family member A1	Cytoplasm	enzyme	
-1.51	0.036	0.232	208950_s_at		ALDH7A1	aldehyde dehydrogenase 7 family member A1	Cytoplasm	enzyme	
-1.72	0.00548	0.143	220365_at		ALLC	allantoicase	Other	enzyme	
-1.52	0.0172	0.187	214707_x_at		ALMS1	ALMS1, centrosome and basal body associated protein	Cytoplasm	other	
1.56	0.00162	0.119	219868_s_at		ANKFY1	ankyrin repeat and FYVE domain containing 1	Cytoplasm	transcription regulator	
1.91	0.0275	0.213	204671_s_at	D	ANKRD6	ankyrin repeat domain 6	Nucleus	transcription regulator	
1.72	0.0321	0.224	204672_s_at	D	ANKRD6	ankyrin repeat domain 6	Nucleus	transcription regulator	
1.64	0.0121	0.169	208103_s_at		ANP32E	acidic nuclear phosphoprotein 32 family member E	Nucleus	other	
1.53	0.043	0.248	210278_s_at		AP4S1	adaptor related protein complex 4 subunit sigma 1	Cytoplasm	other	
1.56	0.0155	0.181	214995_s_at		APOBEC3G	apolipoprotein B mRNA editing enzyme catalytic subunit 3G	Nucleus	enzyme	
-1.85	0.022	0.2	213553_x_at		APOC1	apolipoprotein C1	Extracellular Space	transporter	
-2.26	0.0359	0.232	212884_x_at	D	APOE	apolipoprotein E	Extracellular Space	transporter	
-2.49	0.0442	0.251	212883_at	D	APOE	apolipoprotein E	Extracellular Space	transporter	
1.51	0.0388	0.238	209546_s_at		APOL1	apolipoprotein L1	Extracellular Space	transporter	
2.05	0.000499	0.103	213618_at		ARAP2	ArfGAP with RhoGAP domain, ankyrin repeat and PH domain 2	Cytoplasm	other	
1.86	0.0277	0.213	205414_s_at		ARHGAP44	Rho GTPase activating protein 44	Cytoplasm	other	
1.58	0.0143	0.175	210971_s_at		ARNTL	aryl hydrocarbon receptor nuclear translocator like	Nucleus	transcription regulator	
1.58	0.0131	0.172	205808_at		ASPH	aspartate beta-hydroxylase	Cytoplasm	enzyme	
1.99	0.000431	0.103	205446_s_at		ATF2	activating transcription factor 2	Nucleus	transcription regulator	
-1.52	0.000552	0.103	217550_at		ATF6	activating transcription factor 6	Cytoplasm	transcription regulator	

-1.58	0.000026	0.0655	214149_s_at		ATP6V0E1	ATPase H+ transporting V0 subunit e1	Cytoplasm	transporter	
-1.55	0.0141	0.175	214762_at		ATP6V1G2	ATPase H+ transporting V1 subunit G2	Cytoplasm	transporter	
1.54	0.00628	0.148	220416_at		ATP8B4	ATPase phospholipid transporting 8B4 (putative)	Plasma Membrane	transporter	
2.4	0.0141	0.175	213745_at		ATRNL1	attractin like 1	Other	other	
1.73	0.000072	0.0723	211022_s_at		ATRX	ATRX, chromatin remodeler	Nucleus	transcription regulator	
2.01	0.00498	0.14	211812_s_at	D	B3GALNT1	beta-1,3-N-acetylgalactosaminyltransferase 1 (globoside blood group)	Cytoplasm	enzyme	
1.56	0.0456	0.254	211379_x_at	D	B3GALNT1	beta-1,3-N-acetylgalactosaminyltransferase 1 (globoside blood group)	Cytoplasm	enzyme	
1.76	0.0104	0.162	219326_s_at		B3GNT2	UDP-GlcNAc:betaGal beta-1,3-N-acetylglucosaminyltransferase 2	Cytoplasm	enzyme	
1.85	0.0392	0.239	206233_at		B4GALT6	beta-1,4-galactosyltransferase 6	Cytoplasm	enzyme	
2.3	0.00843	0.157	219528_s_at		BCL11B	B cell CLL/lymphoma 11B	Nucleus	transcription regulator	
1.61	0.031	0.221	203140_at		BCL6	B cell CLL/lymphoma 6	Nucleus	transcription regulator	
1.56	0.0182	0.189	213208_at		BICRAL	BRD4 interacting chromatin remodeling complex associated protein like	Nucleus	other	
1.67	0.0251	0.208	210538_s_at		BIRC3	baculoviral IAP repeat containing 3	Cytoplasm	enzyme	birinapant
1.61	0.0378	0.236	207326_at		BTC	betacellulin	Extracellular Space	growth factor	
-1.6	0.0288	0.215	214116_at		BTD	biotinidase	Extracellular Space	enzyme	
1.52	0.00814	0.155	200921_s_at		BTG1	BTG anti-proliferation factor 1	Nucleus	transcription regulator	
1.57	0.00486	0.139	218983_at		C1RL	complement C1r subcomponent like	Extracellular Space	peptidase	
1.63	0.00456	0.137	213875_x_at		C6orf62	chromosome 6 open reading frame 62	Other	other	
2	0.000569	0.103	210815_s_at		CALCRL	calcitonin receptor like receptor	Plasma Membrane	G-protein coupled receptor	
-1.61	0.00295	0.124	214316_x_at		CALR	calreticulin	Cytoplasm	transcription regulator	
1.5	0.00083	0.106	208853_s_at		CANX	calnexin	Cytoplasm	other	

1.56	0.0192	0.192	203357_s_at		CAPN7	calpain 7	Cytoplasm	peptidase	
1.94	0.00953	0.16	207620_s_at		CASK	calcium/calmodulin dependent serine protein kinase	Plasma Membrane	kinase	
1.65	0.000857	0.106	218711_s_at		CAVIN2	caveolae associated protein 2	Plasma Membrane	other	
1.52	0.00842	0.156	206724_at		CBX4	chromobox 4	Nucleus	transcription regulator	
1.54	0.0109	0.165	212886_at		CCDC69	coiled-coil domain containing 69	Other	other	
3.08	0.0206	0.197	204655_at	D	CCL5	C-C motif chemokine ligand 5	Extracellular Space	cytokine	
3.3	0.0324	0.225	1405_i_at	D	CCL5	C-C motif chemokine ligand 5	Extracellular Space	cytokine	
1.73	0.00135	0.114	211559_s_at		CCNG2	cyclin G2	Nucleus	other	
2.43	0.0103	0.162	206978_at		CCR2	C-C motif chemokine receptor 2	Plasma Membrane	G-protein coupled receptor	PF-4136309, MLN1202, BMS-813160
2.21	0.0119	0.168	205987_at		CD1C	CD1c molecule	Plasma Membrane	other	
2.64	0.01	0.161	215784_at		CD1E	CD1e molecule	Cytoplasm	other	
2.08	0.032	0.224	205831_at		CD2	CD2 molecule	Plasma Membrane	transmembrane receptor	alefacept, siplizumab
1.86	0.0354	0.231	210031_at		CD247	CD247 molecule	Plasma Membrane	transmembrane receptor	blinatumomab
1.87	0.0333	0.227	206150_at		CD27	CD27 molecule	Plasma Membrane	transmembrane receptor	CDX-1127
1.6	0.0488	0.261	206804_at		CD3G	CD3g molecule	Plasma Membrane	transmembrane receptor	blinatumomab
2.91	0.0225	0.202	209795_at		CD69	CD69 molecule	Plasma Membrane	transmembrane receptor	
-1.5	0.0178	0.188	215296_at		CDC42BPA	CDC42 binding protein kinase alpha	Cytoplasm	kinase	
1.53	0.0394	0.239	204677_at		CDH5	cadherin 5	Plasma Membrane	other	
1.51	0.00117	0.109	206474_at		CDK17	cyclin dependent kinase 17	Cytoplasm	kinase	
-10.92	0.0136	0.174	209395_at	D	CHI3L1	chitinase 3 like 1	Extracellular Space	enzyme	
-9.13	0.0185	0.19	209396_s_at	D	CHI3L1	chitinase 3 like 1	Extracellular Space	enzyme	
-5.16	0.0264	0.211	208168_s_at		CHIT1	chitinase 1	Extracellular Space	enzyme	
1.5	0.00741	0.15	207099_s_at		CHM	CHM, Rab escort protein 1	Cytoplasm	enzyme	
1.95	0.0207	0.197	211419_s_at		CHN2	chimerin 2	Cytoplasm	other	
-1.76	0.0258	0.21	207993_s_at		CHP1	calcineurin like EF-hand protein 1	Cytoplasm	transporter	
1.64	0.0326	0.225	212801_at		CIT	citron rho-interacting serine/threonine kinase	Cytoplasm	kinase	
1.92	0.00256	0.122	207980_s_at		CITED2	Cbp/p300 interacting transactivator with Glu/Asp rich carboxy-terminal domain 2	Nucleus	transcription regulator	
1.51	0.0325	0.225	204482_at		CLDN5	claudin 5	Plasma Membrane	other	

Table K: Time dependent changes in gene expression in TAC + MMF Group shown in Figure 8

1.76	0.00736	0.15	201559_s_at	D	CLIC4	chloride intracellular channel 4	Plasma Membrane	ion channel	
1.51	0.0204	0.196	221881_s_at	D	CLIC4	chloride intracellular channel 4	Plasma Membrane	ion channel	
1.69	0.00288	0.124	211981_at		COL4A1	collagen type IV alpha 1 chain	Extracellular Space	other	collagenase clostridium histolyticum
-1.54	0.00562	0.143	205509_at		CPB1	carboxypeptidase B1	Extracellular Space	peptidase	
1.7	0.0213	0.198	201942_s_at		CPD	carboxypeptidase D	Extracellular Space	peptidase	
-1.64	0.0159	0.183	215700_x_at		CPNE6	copine 6	Plasma Membrane	transporter	
1.79	0.0224	0.201	202978_s_at		CREBZF	CREB/ATF bZIP transcription factor	Nucleus	transcription regulator	
-1.66	0.0131	0.172	213275_x_at		CTSB	cathepsin B	Cytoplasm	peptidase	
1.55	0.00512	0.141	210257_x_at	D	CUL4B	cullin 4B	Nucleus	other	
1.51	0.0102	0.162	215997_s_at	D	CUL4B	cullin 4B	Nucleus	other	
2.21	0.00268	0.122	209687_at		CXCL12	C-X-C motif chemokine ligand 12	Extracellular Space	cytokine	NOX-A12
2.04	0.0349	0.229	211919_s_at	D	CXCR4	C-X-C motif chemokine receptor 4	Plasma Membrane	G-protein coupled receptor	AMD 11070, ulocuplumab, cladribine/cytarabine/filgrastim/idarubicin/plerixafor, POL6326, BL-8040, LY-2510924, burixafor, USL311, PF-06747143, plerixafor, filgrastim/plerixafor
1.96	0.043	0.249	209201_x_at	D	CXCR4	C-X-C motif chemokine receptor 4	Plasma Membrane	G-protein coupled receptor	AMD 11070, ulocuplumab, cladribine/cytarabine/filgrastim/idarubicin/plerixafor, POL6326, BL-8040, LY-2510924, burixafor, USL311, PF-06747143, plerixafor, filgrastim/plerixafor
1.93	0.00108	0.106	202435_s_at	D	CYP1B1	cytochrome P450 family 1 subfamily B member 1	Cytoplasm	enzyme	
1.65	0.00154	0.119	202437_s_at	D	CYP1B1	cytochrome P450 family 1 subfamily B member 1	Cytoplasm	enzyme	
1.65	0.00532	0.142	202436_s_at	D	CYP1B1	cytochrome P450 family 1 subfamily B member 1	Cytoplasm	enzyme	
-1.57	0.0265	0.211	203979_at		CYP27A1	cytochrome P450 family 27 subfamily A member 1	Cytoplasm	enzyme	
-3.98	0.0384	0.238	205676_at		CYP27B1	cytochrome P450 family 27 subfamily B member 1	Cytoplasm	enzyme	
-1.78	0.038	0.237	205939_at		CYP3A7	cytochrome P450 family 3 subfamily A member 7	Cytoplasm	enzyme	cobicistat, cobicistat/elvitegravir/emtricitabine/tenofovir disoproxil, atazanavir/cobicistat, cobicistat/darunavir
-1.7	0.0316	0.223	220331_at		CYP46A1	cytochrome P450 family 46 subfamily A member 1	Cytoplasm	enzyme	
-1.71	0.0152	0.18	206153_at		CYP4F11	cytochrome P450 family 4 subfamily F member 11	Cytoplasm	enzyme	

-8.6	0.0283	0.214	210452_x_at		CYP4F2	cytochrome P450 family 4 subfamily F member 2	Cytoplasm	enzyme	
-5.53	0.0251	0.208	206514_s_at		CYP4F3	cytochrome P450 family 4 subfamily F member 3	Cytoplasm	enzyme	
1.7	0.00324	0.128	212853_at	D	DCUN1D4	defective in cullin neddylation 1 domain containing 4	Nucleus	other	
1.52	0.0176	0.188	212851_at	D	DCUN1D4	defective in cullin neddylation 1 domain containing 4	Nucleus	other	
-1.54	0.0434	0.249	213998_s_at		DDX17	DEAD-box helicase 17	Nucleus	enzyme	
1.91	0.0271	0.212	53991_at		DENND2A	DENN domain containing 2A	Cytoplasm	other	
1.65	0.0286	0.215	221081_s_at		DENND2D	DENN domain containing 2D	Cytoplasm	other	
-1.78	0.00444	0.137	215058_at		DENND5B	DENN domain containing 5B	Cytoplasm	other	
-1.5	0.0402	0.241	212504_at		DIP2C	disco interacting protein 2 homolog C	Other	other	
1.73	0.00708	0.149	202516_s_at		DLG1	discs large MAGUK scaffold protein 1	Plasma Membrane	kinase	
1.58	0.00296	0.124	205554_s_at		DNASE1L3	deoxyribonuclease 1 like 3	Nucleus	enzyme	
1.54	0.00884	0.158	205003_at		DOCK4	dedicator of cytokinesis 4	Plasma Membrane	other	
1.87	0.0308	0.221	204751_x_at		DSC2	desmocollin 2	Plasma Membrane	other	
-1.51	0.00117	0.109	203590_at		DYNC1LI2	dynein cytoplasmic 1 light intermediate chain 2	Cytoplasm	other	
-1.55	0.0117	0.168	202735_at		EBP	EBP, cholestenol delta-isomerase	Cytoplasm	enzyme	SR 31747
1.56	0.0187	0.191	204463_s_at		EDNRA	endothelin receptor type A	Plasma Membrane	transmembrane receptor	bosentan, avosentan, clazosentan, ambrisentan, sitaxsentan, zibotentan, SB 234551, TBC 3214, BSF 302146, YM598, macitentan, ambrisentan/tadalafil, fandosentan, atrasentan
2.07	0.00914	0.158	204841_s_at		EEA1	early endosome antigen 1	Cytoplasm	other	
1.51	0.0403	0.242	31845_at		ELF4	E74 like ETS transcription factor 4	Nucleus	transcription regulator	
1.72	0.0234	0.204	207691_x_at	D	ENTPD1	ectonucleoside triphosphate diphosphohydrolase 1	Plasma Membrane	enzyme	
1.69	0.0261	0.21	209474_s_at	D	ENTPD1	ectonucleoside triphosphate diphosphohydrolase 1	Plasma Membrane	enzyme	
-2.1	0.025	0.207	205757_at		ENTPD5	ectonucleoside triphosphate diphosphohydrolase 5	Cytoplasm	enzyme	

Table K: Time dependent changes in gene expression in TAC + MMF Group shown in Figure 8

1.98	0.00981	0.16	203499_at		EPHA2	EPH receptor A2	Plasma Membrane	kinase	dasatinib, MEDI-547, regorafenib, ephA2-targeting DOPC-encapsulated siRNA, DS-8895a, dasatinib/dexamethasone, dasatinib/nilotinib, cytarabine/dasatinib/dexamethasone/methotrexate, dasatinib/erlotinib
1.69	0.0102	0.162	206852_at		EPHA7	EPH receptor A7	Plasma Membrane	kinase	
1.66	0.00086	0.106	210870_s_at		EPM2A	EPM2A, laforin glucan phosphatase	Cytoplasm	phosphatase	
-1.55	0.00171	0.12	396_f_at		EPOR	erythropoietin receptor	Plasma Membrane	transmembrane receptor	darbepoetin alfa, continuous erythropoietin receptor activator, peginesatide, epoetin beta, EPO, EPO/sargramostim, darbepoetin alfa/filgrastim, EPO/filgrastim, erythropoietin/lenalidomide
1.51	0.024	0.205	221542_s_at		ERLIN2	ER lipid raft associated 2	Plasma Membrane	kinase	
-2.42	0.004	0.135	208394_x_at		ESM1	endothelial cell specific molecule 1	Extracellular Space	growth factor	
1.85	0.00862	0.157	218748_s_at		EXOC5	exocyst complex component 5	Cytoplasm	other	
-5.65	0.0445	0.252	205892_s_at		FABP1	fatty acid binding protein 1	Cytoplasm	transporter	
-1.82	0.0093	0.16	215600_x_at		FBXW12	F-box and WD repeat domain containing 12	Cytoplasm	enzyme	
2.5	0.00352	0.131	211734_s_at		FCER1A	Fc fragment of IgE receptor Ia	Plasma Membrane	transmembrane receptor	
1.57	0.00583	0.144	207501_s_at		FGF12	fibroblast growth factor 12	Extracellular Space	other	
1.93	0.00541	0.143	204421_s_at	D	FGF2	fibroblast growth factor 2	Extracellular Space	growth factor	pentosan polysulfate, suradista, CP-547632, sucralfate
2.16	0.0303	0.219	204422_s_at	D	FGF2	fibroblast growth factor 2	Extracellular Space	growth factor	pentosan polysulfate, suradista, CP-547632, sucralfate
2.1	0.0262	0.21	211401_s_at		FGFR2	fibroblast growth factor receptor 2	Plasma Membrane	kinase	pazopanib, lenvatinib, nintedanib, regorafenib, dexamethasone/thalidomide, bortezomib/dexamethasone/thalidomide, rituximab/thalidomide, bortezomib/thalidomide, prednisone/thalidomide, BAY1179470, lucitanib, debio 1347, ARQ 087, FPA144, BAY1187982, pemigatinib, PRN1371, docetaxel/nintedanib, everolimus/lenvatinib, thalidomide, palifermin
1.85	0.00593	0.145	204834_at		FGL2	fibrinogen like 2	Extracellular Space	peptidase	
2.18	0.00326	0.128	220170_at		FHL5	four and a half LIM domains 5	Nucleus	transcription regulator	
1.79	0.0101	0.162	210186_s_at		FKBP1A	FK506 binding protein 1A	Cytoplasm	enzyme	methotrexate/sirolimus/tacrolimus, everolimus/exemestane, lenalidomide/temsirolimus, imatinib/sirolimus, cyclophosphamide/sirolimus, cyclosporine A/tacrolimus, cyclosporine A/sirolimus/tacrolimus, everolimus/panobinostat, everolimus/pasireotide, ABI-009, everolimus/sorafenib, everolimus/gefitinib, everolimus/lenvatinib, sirolimus, temsirolimus, tacrolimus, everolimus/tamoxifen, everolimus, everolimus/fulvestrant, everolimus/letrozole, prednisone/tacrolimus, methylprednisolone/tacrolimus, everolimus/vandetanib, everolimus/ribociclib
2.08	0.0102	0.162	210786_s_at		FLI1	Fli-1 proto-oncogene, ETS transcription factor	Nucleus	transcription regulator	
-1.51	0.00149	0.117	220352_x_at		FLJ42627	uncharacterized LOC645644	Other	other	

Table K: Time dependent changes in gene expression in TAC + MMF Group shown in Figure 8

1.62	0.0105	0.163	212288_at		FNBP1	formin binding protein 1	Nucleus	enzyme	
2.12	0.000056	0.0687	215910_s_at		FNDC3A	fibronectin type III domain containing 3A	Cytoplasm	other	
1.95	0.0134	0.173	206307_s_at		FOXD1	forkhead box D1	Nucleus	transcription regulator	
1.71	0.00121	0.109	201564_s_at		FSCN1	fascin actin-bundling protein 1	Cytoplasm	other	
2	0.00232	0.122	210188_at		GABPA	GA binding protein transcription factor subunit alpha	Nucleus	transcription regulator	
1.51	0.0203	0.196	211810_s_at		GALC	galactosylceramidase	Cytoplasm	enzyme	
1.59	0.0481	0.259	210002_at		GATA6	GATA binding protein 6	Nucleus	transcription regulator	
1.71	0.00943	0.16	212241_at		GCOM1	GRINL1A complex locus 1	Other	other	
1.55	0.0169	0.186	218458_at		GMCL1	germ cell-less, spermatogenesis associated 1	Nucleus	other	
1.63	0.000342	0.0977	211426_x_at		GNAQ	G protein subunit alpha q	Plasma Membrane	enzyme	
1.5	0.00103	0.106	214548_x_at		GNAS	GNAS complex locus	Plasma Membrane	enzyme	
-1.97	0.0235	0.204	202382_s_at		GNPDA1	glucosamine-6-phosphate deaminase 1	Cytoplasm	enzyme	
-1.59	0.00982	0.16	204324_s_at		GOLIM4	golgi integral membrane protein 4	Cytoplasm	other	
1.77	0.0164	0.184	210007_s_at		GPD2	glycerol-3-phosphate dehydrogenase 2	Cytoplasm	enzyme	
6.14	0.0277	0.213	209469_at	D	GPM6A	glycoprotein M6A	Plasma Membrane	ion channel	
7.24	0.0327	0.225	209470_s_at	D	GPM6A	glycoprotein M6A	Plasma Membrane	ion channel	
-2	0.0373	0.235	204137_at		GPR137B	G protein-coupled receptor 137B	Plasma Membrane	other	
2.3	0.0242	0.206	207651_at		GPR171	G protein-coupled receptor 171	Plasma Membrane	G-protein coupled receptor	
1.97	0.0411	0.243	210279_at		GPR18	G protein-coupled receptor 18	Plasma Membrane	G-protein coupled receptor	
-1.54	0.0429	0.248	222140_s_at		GPR89A/GPF	G protein-coupled receptor 89B	Cytoplasm	ion channel	
1.58	0.00567	0.143	212856_at		GRAMD4	GRAM domain containing 4	Cytoplasm	other	
1.54	0.0467	0.256	204396_s_at		GRK5	G protein-coupled receptor kinase 5	Plasma Membrane	kinase	
-1.81	0.000593	0.103	222104_x_at		GTF2H3	general transcription factor IIH subunit 3	Nucleus	transcription regulator	
2.87	0.0282	0.214	205488_at		GZMA	granzyme A	Cytoplasm	peptidase	
2.32	0.0385	0.238	206666_at		GZMK	granzyme K	Cytoplasm	peptidase	
-1.63	0.00414	0.135	220071_x_at		HAUS2	HAUS augmin like complex subunit 2	Cytoplasm	other	
1.65	0.0367	0.233	206082_at		HCP5	HLA complex P5	Other	other	

2.43	0.002	0.12	210148_at		HIPK3	homeodomain interacting protein kinase 3	Nucleus	kinase	
1.68	0.0134	0.173	200697_at		HK1	hexokinase 1	Cytoplasm	kinase	
1.74	0.0464	0.256	221491_x_at		HLA-DRB1	major histocompatibility complex, class II, DR beta 1	Plasma Membrane	transmembrane receptor	apolizumab
-1.51	0.0466	0.256	208429_x_at		HNF4A	hepatocyte nuclear factor 4 alpha	Nucleus	transcription regulator	
-1.52	0.0403	0.242	222264_at		HNRNPUL2	heterogeneous nuclear ribonucleoprotein U like 2	Nucleus	other	
1.59	0.000285	0.0977	213823_at		HOXA11	homeobox A11	Nucleus	transcription regulator	
1.69	0.00722	0.15	205466_s_at		HS3ST1	heparan sulfate-glucosamine 3-sulfotransferase 1	Cytoplasm	enzyme	
1.76	0.0358	0.232	211220_s_at		HSF2	heat shock transcription factor 2	Nucleus	transcription regulator	
1.68	0.00184	0.12	202558_s_at		HSPA13	heat shock protein family A (Hsp70) member 13	Cytoplasm	other	
1.7	0.00473	0.138	202601_s_at		HTATSF1	HIV-1 Tat specific factor 1	Nucleus	transcription regulator	
-1.5	0.00163	0.119	206944_at		HTR6	5-hydroxytryptamine receptor 6	Plasma Membrane	G-protein coupled receptor	iloperidone, asenapine, WAY-181187, olanzapine, sertindole
1.64	0.0165	0.184	206855_s_at		HYAL2	hyaluronoglucosaminidase 2	Cytoplasm	enzyme	hyaluronic acid, hylan, diclofenac/hyaluronic acid
1.62	0.0369	0.234	207826_s_at		ID3	inhibitor of DNA binding 3, HLH protein	Nucleus	transcription regulator	
2.28	0.0406	0.242	209291_at	D	ID4	inhibitor of DNA binding 4, HLH protein	Nucleus	transcription regulator	
1.87	0.0485	0.26	209293_x_at	D	ID4	inhibitor of DNA binding 4, HLH protein	Nucleus	transcription regulator	
-1.88	0.0169	0.186	201193_at		IDH1	isocitrate dehydrogenase (NADP(+)) 1, cytosolic	Cytoplasm	enzyme	ivosidenib, IDH305, olutasidenib, BAY1436032, IDH1 inhibitor, DS-1001
1.51	0.00623	0.147	217502_at		IFIT2	interferon induced protein with tetratricopeptide repeats 2	Cytoplasm	other	
3.02	0.0451	0.254	217022_s_at		IGHA1	immunoglobulin heavy constant alpha 1	Extracellular Space	other	
2.56	0.0136	0.174	209374_s_at	D	IGHM	immunoglobulin heavy constant mu	Plasma Membrane	transmembrane receptor	
1.66	0.015	0.179	216491_x_at	D	IGHM	immunoglobulin heavy constant mu	Plasma Membrane	transmembrane receptor	
1.62	0.0368	0.233	211634_x_at	D	IGHM	immunoglobulin heavy constant mu	Plasma Membrane	transmembrane receptor	

Table K: Time dependent changes in gene expression in TAC + MMF Group shown in Figure 8

2.33	0.0136	0.174	211644_x_at	D	IGKC	immunoglobulin kappa constant	Extracellular Space	other	
1.63	0.0299	0.218	211643_x_at	D	IGKC	immunoglobulin kappa constant	Extracellular Space	other	
2.89	0.0329	0.226	214669_x_at	D	IGKC	immunoglobulin kappa constant	Extracellular Space	other	
2.28	0.0428	0.248	221651_x_at	D	IGKC	immunoglobulin kappa constant	Extracellular Space	other	
2.22	0.0495	0.263	221671_x_at	D	IGKC	immunoglobulin kappa constant	Extracellular Space	other	
2.38	0.0409	0.243	211645_x_at		IGKV1-17	immunoglobulin kappa variable 1-17	Extracellular Space	other	
1.57	0.0457	0.254	217378_x_at		IGKV1OR2-1(	immunoglobulin kappa variable 1/OR2-108 (non-functional)	Other	other	
1.71	0.00213	0.121	205992_s_at		IL15	interleukin 15	Extracellular Space	cytokine	ALT-803
1.65	0.0436	0.249	209827_s_at		IL16	interleukin 16	Extracellular Space	cytokine	
1.98	0.00461	0.137	222062_at		IL27RA	interleukin 27 receptor subunit alpha	Plasma Membrane	transmembrane receptor	
2.13	0.0365	0.233	205798_at		IL7R	interleukin 7 receptor	Plasma Membrane	transmembrane receptor	recombinant human interleukin-7
1.51	0.00926	0.159	205376_at		INPP4B	inositol polyphosphate-4-phosphatase type II B	Cytoplasm	phosphatase	
-1.75	0.00328	0.128	219843_at		IPP	intracisternal A particle-promoted polypeptide	Cytoplasm	other	
1.71	0.0285	0.215	204057_at		IRF8	interferon regulatory factor 8	Nucleus	transcription regulator	
2.4	0.0394	0.239	204698_at		ISG20	interferon stimulated exonuclease gene 20	Nucleus	enzyme	
-1.51	0.0385	0.238	218893_at		ISOC2	isochorismatase domain containing 2	Cytoplasm	enzyme	
2.88	0.0483	0.26	205885_s_at		ITGA4	integrin subunit alpha 4	Plasma Membrane	transmembrane receptor	natalizumab, vedolizumab
1.57	0.00388	0.135	205816_at		ITGB8	integrin subunit beta 8	Plasma Membrane	other	
1.98	0.0363	0.233	211339_s_at		ITK	IL2 inducible T cell kinase	Cytoplasm	kinase	pazopanib, crizotinib/pazopanib
1.73	0.0107	0.164	211323_s_at		ITPR1	inositol 1,4,5-trisphosphate receptor type 1	Cytoplasm	ion channel	
1.69	0.00808	0.155	201188_s_at	D	ITPR3	inositol 1,4,5-trisphosphate receptor type 3	Cytoplasm	ion channel	
1.72	0.0135	0.174	201189_s_at	D	ITPR3	inositol 1,4,5-trisphosphate receptor type 3	Cytoplasm	ion channel	
2.95	0.0368	0.234	209098_s_at		JAG1	jagged 1	Extracellular Space	growth factor	
1.85	0.00515	0.141	205842_s_at	D	JAK2	Janus kinase 2	Cytoplasm	kinase	WP1066, tofacitinib, fedratinib, pacritinib, XL019, ruxolitinib, AZD1480, momelotinib, baricitinib, NS-018, SB-1317, gandotinib, BMS-911543, jak2 inhibitor, methotrexate/tofacitinib, baricitinib/methotrexate

Table K: Time dependent changes in gene expression in TAC + MMF Group shown in Figure 8

1.77	0.00662	0.148	205841_at	D	JAK2	Janus kinase 2	Cytoplasm	kinase	WP1066, tofacitinib, fedratinib, pacritinib, XL019, ruxolitinib, AZD1480, momelotinib, baricitinib, NS-018, SB-1317, gandotinib, BMS-911543, jak2 inhibitor, methotrexate/tofacitinib, baricitinib/methotrexate
1.54	0.00787	0.154	219095_at		JMJD7-PLA2	JMJD7-PLA2G4B readthrough	Cytoplasm	other	
1.53	0.0402	0.241	210078_s_at		KCNAB1	potassium voltage-gated channel subfamily A member regulatory beta subunit 1	Plasma Membrane	ion channel	
-1.78	0.0414	0.244	203402_at		KCNAB2	potassium voltage-gated channel subfamily A regulatory beta subunit 2	Plasma Membrane	ion channel	
1.55	0.0126	0.171	203990_s_at	D	KDM6A	lysine demethylase 6A	Nucleus	enzyme	
1.5	0.0172	0.187	203991_s_at	D	KDM6A	lysine demethylase 6A	Nucleus	enzyme	
1.61	0.0197	0.193	220266_s_at	D	KLF4	Kruppel like factor 4	Nucleus	transcription regulator	
1.62	0.0236	0.204	221841_s_at	D	KLF4	Kruppel like factor 4	Nucleus	transcription regulator	
1.52	0.0243	0.206	220238_s_at		KLHL7	kelch like family member 7	Nucleus	other	
3.5	0.00699	0.149	214470_at		KLRB1	killer cell lectin like receptor B1	Plasma Membrane	transmembrane receptor	
2.48	0.0143	0.175	220646_s_at		KLRF1	killer cell lectin like receptor F1	Plasma Membrane	transmembrane receptor	
2.4	0.0176	0.188	204891_s_at	D	LCK	LCK proto-oncogene, Src family tyrosine kinase	Cytoplasm	kinase	dasatinib, pazopanib, dacomitinib, nintedanib, JNJ-26483327, docetaxel/nintedanib, dasatinib/nilotinib, crizotinib/pazopanib, dasatinib/erlotinib
1.52	0.0326	0.225	204890_s_at	D	LCK	LCK proto-oncogene, Src family tyrosine kinase	Cytoplasm	kinase	dasatinib, pazopanib, dacomitinib, nintedanib, JNJ-26483327, docetaxel/nintedanib, dasatinib/nilotinib, crizotinib/pazopanib, dasatinib/erlotinib
2.02	0.0023	0.122	211354_s_at	D	LEPR	leptin receptor	Plasma Membrane	transmembrane receptor	recombinant-methionyl human leptin
2.03	0.00277	0.122	211355_x_at	D	LEPR	leptin receptor	Plasma Membrane	transmembrane receptor	recombinant-methionyl human leptin
2.09	0.00666	0.148	211356_x_at	D	LEPR	leptin receptor	Plasma Membrane	transmembrane receptor	recombinant-methionyl human leptin
1.57	0.00203	0.12	219884_at		LHX6	LIM homeobox 6	Nucleus	transcription regulator	
1.52	0.0221	0.2	219399_at		LIN7C	lin-7 homolog C, crumbs cell polarity complex component	Cytoplasm	other	
1.82	0.00204	0.12	203294_s_at		LMAN1	lectin, mannose binding 1	Cytoplasm	other	
1.58	0.00214	0.121	207904_s_at		LNPEP	leucyl and cystinyl aminopeptidase	Cytoplasm	peptidase	
-1.54	0.0175	0.187	216229_x_at		LOC1053796	uncharacterized LOC105379655	Other	other	

-1.55	0.00327	0.128	215778_x_at		LOC1079867	enolase-phosphatase E1	Other	other	
1.54	0.000283	0.0977	201818_at		LPCAT1	lysophosphatidylcholine acyltransferase 1	Cytoplasm	enzyme	
1.59	0.0362	0.233	204442_x_at		LTBP4	latent transforming growth factor beta binding protein 4	Extracellular Space	growth factor	
-1.8	0.0103	0.162	207106_s_at		LTK	leukocyte receptor tyrosine kinase	Plasma Membrane	kinase	lorlatinib
2	0.0019	0.12	205668_at		LY75	lymphocyte antigen 75	Plasma Membrane	transmembrane receptor	CDX 1401, MEN1309
1.83	0.036	0.232	36711_at		MAFF	MAF bZIP transcription factor F	Nucleus	transcription regulator	
-2.35	0.0246	0.207	222372_at		MAGI1	membrane associated guanylate kinase, WW and PDZ domain containing 1	Plasma Membrane	kinase	
2.05	0.0038	0.134	208116_s_at		MAN1A1	mannosidase alpha class 1A member 1	Cytoplasm	enzyme	
1.5	0.00806	0.155	217921_at		MAN1A2	mannosidase alpha class 1A member 2	Cytoplasm	enzyme	
1.8	0.000158	0.0892	219003_s_at		MANEA	mannosidase endo-alpha	Cytoplasm	enzyme	
-1.67	0.00179	0.12	216206_x_at		MAP2K7	mitogen-activated protein kinase kinase 7	Cytoplasm	kinase	
2.81	0.00545	0.143	214786_at		MAP3K1	mitogen-activated protein kinase kinase kinase 1	Cytoplasm	kinase	E 6201
1.59	0.000627	0.103	221695_s_at		MAP3K2	mitogen-activated protein kinase kinase kinase 2	Cytoplasm	kinase	
1.78	0.0436	0.249	203553_s_at		MAP4K5	mitogen-activated protein kinase kinase kinase kinase 5	Cytoplasm	kinase	vemurafenib, cobimetinib/vemurafenib, dabrafenib/trametinib/vemurafenib, cetuximab/vemurafenib, trametinib/vemurafenib, irinotecan/panitumumab/vemurafenib, cetuximab/irinotecan/vemurafenib, crizotinib/vemurafenib
1.85	0.0207	0.197	201669_s_at		MARCKS	myristoylated alanine rich protein kinase C substrate	Plasma Membrane	other	
-2.08	0.0226	0.202	222348_at		MAST4	microtubule associated serine/threonine kinase family member 4	Other	kinase	
1.73	0.00209	0.121	205017_s_at	D	MBNL2	muscleblind like splicing regulator 2	Nucleus	other	
1.96	0.00639	0.148	205018_s_at	D	MBNL2	muscleblind like splicing regulator 2	Nucleus	other	
1.85	0.0433	0.249	210869_s_at		MCAM	melanoma cell adhesion molecule	Plasma Membrane	other	

1.52	0.0136	0.174	214056_at		MCL1	MCL1, BCL2 family apoptosis regulator	Cytoplasm	transporter	MIK665, AMG 176, AMG 397
1.5	0.00759	0.152	210154_at		ME2	malic enzyme 2	Cytoplasm	enzyme	
1.59	0.000918	0.106	216071_x_at		MED12	mediator complex subunit 12	Nucleus	transcription regulator	
-2.22	0.000031	0.0655	219730_at	D	MED18	mediator complex subunit 18	Nucleus	other	
-1.9	0.00255	0.122	221650_s_at	D	MED18	mediator complex subunit 18	Nucleus	other	
1.52	0.0269	0.212	212830_at		MEGF9	multiple EGF like domains 9	Extracellular Space	other	
1.91	0.0105	0.163	211913_s_at		MERTK	MER proto-oncogene, tyrosine kinase	Plasma Membrane	kinase	cabozantinib, cabozantinib/erlotinib, sitravatinib, ONO-7475, INCB081776, cabozantinib/nivolumab
-1.58	0.0359	0.232	204027_s_at		METTL1	methyltransferase like 1	Nucleus	enzyme	
-1.54	0.00556	0.143	211424_x_at	D	METTL7A	methyltransferase like 7A	Cytoplasm	other	
-1.84	0.0138	0.174	209703_x_at	D	METTL7A	methyltransferase like 7A	Cytoplasm	other	
1.59	0.0253	0.209	91816_f_at		MEX3D	mex-3 RNA binding family member D	Nucleus	other	
1.53	0.0118	0.168	219797_at		MGAT4A	alpha-1,3-mannosyl-glycoprotein 4-beta-N-acetylglucosaminyltransferase A	Cytoplasm	enzyme	
-1.72	0.0356	0.231	212925_at		MISP	mitotic spindle positioning	Plasma Membrane	other	
1.65	0.0233	0.204	205408_at		MLLT10	MLLT10, histone lysine methyltransferase DOT1L cofactor	Nucleus	transcription regulator	
-4.29	0.032	0.224	204580_at		MMP12	matrix metallopeptidase 12	Extracellular Space	peptidase	marimastat
1.88	0.0384	0.238	219091_s_at		MMRN2	multimerin 2	Extracellular Space	other	
2.33	0.0162	0.183	209708_at		MOXD1	monooxygenase DBH like 1	Cytoplasm	enzyme	
1.56	0.0081	0.155	209421_at		MSH2	mutS homolog 2	Nucleus	enzyme	
1.58	0.0137	0.174	211363_s_at		MTAP	methylthioadenosine phosphorylase	Nucleus	enzyme	
-2.27	0.0315	0.223	205675_at		MTTP	microsomal triglyceride transfer protein	Cytoplasm	transporter	lomitapide, hesperetin
-1.94	0.0488	0.261	218687_s_at		MUC13	mucin 13, cell surface associated	Extracellular Space	other	
1.53	0.0014	0.114	214156_at		MYRIP	myosin VIIA and Rab interacting protein	Cytoplasm	other	
-1.54	0.0132	0.173	220720_x_at		MZT2B	mitotic spindle organizing protein 2B	Cytoplasm	other	
-1.63	0.0175	0.188	214753_at		N4BP2L2	NEDD4 binding protein 2 like 2	Nucleus	transcription regulator	

1.53	0.0165	0.184	222369_at		NAA40	N(alpha)-acetyltransferase 40, NatD catalytic subunit	Other	other	
1.81	0.000668	0.103	211139_s_at	D	NAB1	NGFI-A binding protein 1	Nucleus	transcription regulator	
2.34	0.000918	0.106	208047_s_at	D	NAB1	NGFI-A binding protein 1	Nucleus	transcription regulator	
1.68	0.0404	0.242	217739_s_at		NAMPT	nicotinamide phosphoribosyltransferase	Extracellular Space	cytokine	daporinad, KPT-9274
1.77	0.0282	0.214	217465_at		NCKAP1	NCK associated protein 1	Plasma Membrane	other	
1.52	0.00371	0.133	209105_at		NCOA1	nuclear receptor coactivator 1	Nucleus	transcription regulator	
2.4	0.0024	0.122	205732_s_at		NCOA2	nuclear receptor coactivator 2	Nucleus	transcription regulator	
1.59	0.00762	0.152	218073_s_at		NDC1	NDC1 transmembrane nucleoporin	Nucleus	other	
-1.63	0.000385	0.102	220983_s_at	D	NEAT1	nuclear paraspeckle assembly transcript 1	Other	other	
-2.14	0.0217	0.199	214657_s_at	D	NEAT1	nuclear paraspeckle assembly transcript 1	Other	other	
1.66	0.0247	0.207	207279_s_at		NEBL	nebulette	Plasma Membrane	other	
-1.75	0.0123	0.17	215005_at		NECAB2	N-terminal EF-hand calcium binding protein 2	Cytoplasm	other	
-1.62	0.0228	0.202	201829_at		NET1	neuroepithelial cell transforming 1	Nucleus	other	
-1.55	0.00804	0.154	222339_x_at		NFIX	nuclear factor I X	Nucleus	transcription regulator	
1.9	0.000671	0.103	204107_at		NFYA	nuclear transcription factor Y subunit alpha	Nucleus	transcription regulator	
1.82	0.00597	0.145	218129_s_at		NFYB	nuclear transcription factor Y subunit beta	Nucleus	transcription regulator	
1.56	0.00172	0.12	218036_x_at		NMD3	NMD3 ribosome export adaptor	Nucleus	other	
2.69	0.0451	0.254	207400_at		NPY5R	neuropeptide Y receptor Y5	Plasma Membrane	G-protein coupled receptor	
1.67	0.00523	0.141	215073_s_at	D	NR2F2	nuclear receptor subfamily 2 group F member 2	Nucleus	ligand-dependent nuclear receptor	
1.52	0.00914	0.158	209120_at	D	NR2F2	nuclear receptor subfamily 2 group F member 2	Nucleus	ligand-dependent nuclear receptor	
4.03	0.0143	0.175	221796_at		NTRK2	neurotrophic receptor tyrosine kinase 2	Plasma Membrane	kinase	cabozantinib, cabozantinib/erlotinib, TSR-011, entrectinib, lorlatinib, PLX7486, larotrectinib, DS-6051b, TPX-0005, LOXO-195, ONO-7579, cabozantinib/nivolumab
-1.87	0.0327	0.225	200646_s_at	D	NUCB1	nucleobindin 1	Cytoplasm	other	
-1.59	0.0346	0.229	200649_at	D	NUCB1	nucleobindin 1	Cytoplasm	other	
1.58	0.00568	0.143	213461_at		NUDT21	nudix hydrolase 21	Nucleus	other	

Table K: Time dependent changes in gene expression in TAC + MMF Group shown in Figure 8

-1.53	0.0295	0.218	214251_s_at		NUMA1	nuclear mitotic apparatus protein 1	Nucleus	other	
2.15	0.00391	0.135	37079_at		NUS1P3	NUS1 pseudogene 3	Other	other	
2.19	0.00194	0.12	209629_s_at		NXT2	nuclear transport factor 2 like export factor 2	Nucleus	transporter	
1.69	0.0142	0.175	207564_x_at		OGT	O-linked N-acetylglucosamine (GlcNAc) transferase	Cytoplasm	enzyme	
1.58	0.0346	0.229	203352_at		ORC4	origin recognition complex subunit 4	Nucleus	other	
1.7	0.0095	0.16	206637_at		P2RY14	purinergic receptor P2Y14	Plasma Membrane	G-protein coupled receptor	
-1.69	0.042	0.245	202733_at		P4HA2	prolyl 4-hydroxylase subunit alpha 2	Cytoplasm	transporter	
1.5	0.0299	0.218	211547_s_at		PAFAH1B1	platelet activating factor acetylhydrolase 1b regulatory subunit 1	Cytoplasm	enzyme	
1.68	0.0433	0.249	208875_s_at		PAK2	p21 (RAC1) activated kinase 2	Cytoplasm	kinase	
1.56	0.000141	0.0846	212720_at		PAPOLA	poly(A) polymerase alpha	Nucleus	enzyme	
1.57	0.0466	0.256	204005_s_at		PAWR	pro-apoptotic WT1 regulator	Nucleus	transcription regulator	
-2.01	0.00028	0.0977	213517_at		PCBP2	poly(rC) binding protein 2	Nucleus	other	
1.62	0.0462	0.255	210674_s_at		PCDHA12	protocadherin alpha 12	Other	other	
-1.52	0.00628	0.148	211713_x_at		PCLAF	PCNA clamp associated factor	Nucleus	other	
1.59	0.00595	0.145	209996_x_at		PCM1	pericentriolar material 1	Cytoplasm	other	
1.73	0.00424	0.136	202731_at	D	PDCD4	programmed cell death 4	Nucleus	other	
1.62	0.00649	0.148	202730_s_at	D	PDCD4	programmed cell death 4	Nucleus	other	
1.56	0.00596	0.145	204735_at		PDE4A	phosphodiesterase 4A	Cytoplasm	enzyme	enprofylline, dyphylline, caffeine/ergotamine, arofylline, tetomilast, L 869298, ibudilast, apremilast, aspirin/dipyridamole/telmisartan, drotaverin, anagrelide, cilomilast, milrinone, dipyridamole, L-826,141, aspirin/dipyridamole, amrinone, ketotifen, roflumilast, theophylline, pentoxifylline, caffeine
1.69	0.00172	0.12	211302_s_at		PDE4B	phosphodiesterase 4B	Cytoplasm	enzyme	enprofylline, dyphylline, nitroglycerin, caffeine/ergotamine, arofylline, tetomilast, L 869298, ibudilast, apremilast, anagrelide, cilomilast, milrinone, dipyridamole, L-826,141, ketotifen, roflumilast, tolbutamide, papaverine, theophylline, pentoxifylline, caffeine
-1.61	0.012	0.168	206792_x_at		PDE4C	phosphodiesterase 4C	Cytoplasm	enzyme	dyphylline, nitroglycerin, caffeine/ergotamine, arofylline, tetomilast, L 869298, ibudilast, anagrelide, cilomilast, milrinone, dipyridamole, L-826,141, ketotifen, roflumilast, tolbutamide, theophylline, pentoxifylline, caffeine

1.69	0.0175	0.187	205463_s_at		PDGFA	platelet derived growth factor subunit A	Extracellular Space	growth factor	
1.85	0.00629	0.148	205960_at		PDK4	pyruvate dehydrogenase kinase 4	Cytoplasm	kinase	
1.65	0.0041	0.135	206288_at		PGGT1B	protein geranylgeranyltransferase type I subunit beta	Cytoplasm	enzyme	
-1.67	0.0281	0.214	219891_at		PGPEP1	pyroglutamyl-peptidase I	Cytoplasm	peptidase	
1.79	0.00136	0.114	209803_s_at		PHLDA2	pleckstrin homology like domain family A member 2	Cytoplasm	other	
1.74	0.00004	0.0655	204484_at		PIK3C2B	phosphatidylinositol-4-phosphate 3-kinase catalytic subunit type 2 beta	Cytoplasm	kinase	
1.64	0.00887	0.158	216218_s_at		PLCL2	phospholipase C like 2	Cytoplasm	enzyme	
1.53	0.00203	0.12	217677_at		PLEKHA2	pleckstrin homology domain containing A2	Cytoplasm	other	
-1.52	0.0265	0.211	220952_s_at		PLEKHA5	pleckstrin homology domain containing A5	Cytoplasm	other	
-1.79	0.029	0.216	209122_at		PLIN2	perilipin 2	Plasma Membrane	other	
-1.76	0.0374	0.235	200827_at		PLOD1	procollagen-lysine,2-oxoglutarate 5-dioxygenase 1	Cytoplasm	enzyme	
-1.76	0.0333	0.227	214807_at		PLXDC2	plexin domain containing 2	Extracellular Space	other	
-1.54	0.00108	0.106	212705_x_at		PNPLA2	patatin like phospholipase domain containing 2	Cytoplasm	enzyme	
-2.26	0.0447	0.252	220675_s_at		PNPLA3	patatin like phospholipase domain containing 3	Cytoplasm	enzyme	
1.5	0.0054	0.143	214374_s_at	D	PPFIBP1	PPFIA binding protein 1	Plasma Membrane	other	
-1.56	0.0222	0.2	214375_at	D	PPFIBP1	PPFIA binding protein 1	Plasma Membrane	other	
2.24	0.0327	0.225	213849_s_at		PPP2R2B	protein phosphatase 2 regulatory subunit Bbeta	Cytoplasm	phosphatase	
1.57	0.00682	0.148	209799_at		PRKAA1	protein kinase AMP-activated catalytic subunit alpha 1	Cytoplasm	kinase	phenformin
1.89	0.000577	0.103	202742_s_at		PRKACB	protein kinase cAMP-activated catalytic subunit beta	Cytoplasm	kinase	
1.61	0.00472	0.138	206099_at	D	PRKCH	protein kinase C eta	Cytoplasm	kinase	ingenol mebutate
1.61	0.0156	0.182	218764_at	D	PRKCH	protein kinase C eta	Cytoplasm	kinase	ingenol mebutate
1.58	0.0141	0.175	209677_at		PRKCI	protein kinase C iota	Cytoplasm	kinase	ingenol mebutate

1.53	0.0166	0.184	202529_at		PRPSAP1	phosphoribosyl pyrophosphate synthetase associated protein 1	Extracellular Space	other	
-1.78	0.00398	0.135	219392_x_at		PRR11	proline rich 11	Other	other	
1.6	0.0233	0.203	219383_at		PRR5L	proline rich 5 like	Cytoplasm	other	
1.72	0.0133	0.173	206631_at		PTGER2	prostaglandin E receptor 2	Plasma Membrane	G-protein coupled receptor	misoprostol, prostaglandin E2, prostaglandin E1, CP 533536, diclofenac/misoprostol, EP2 receptor agonist
-2.22	0.0129	0.172	211909_x_at		PTGER3	prostaglandin E receptor 3	Plasma Membrane	G-protein coupled receptor	misoprostol, prostaglandin E2, prostaglandin E1, diclofenac/misoprostol, bimatoprost
1.89	0.000766	0.106	209896_s_at	D	PTPN11	protein tyrosine phosphatase, non-receptor type 11	Cytoplasm	phosphatase	TNO155, RMC-4630
1.65	0.00301	0.125	209895_at	D	PTPN11	protein tyrosine phosphatase, non-receptor type 11	Cytoplasm	phosphatase	TNO155, RMC-4630
1.52	0.00278	0.122	200927_s_at		RAB14	RAB14, member RAS oncogene family	Cytoplasm	enzyme	
1.87	0.0272	0.212	210951_x_at	D	RAB27A	RAB27A, member RAS oncogene family	Cytoplasm	enzyme	
1.64	0.0307	0.22	209514_s_at	D	RAB27A	RAB27A, member RAS oncogene family	Cytoplasm	enzyme	
1.71	0.00166	0.12	201048_x_at		RAB6A	RAB6A, member RAS oncogene family	Cytoplasm	enzyme	
1.53	0.041	0.243	214552_s_at		RABEP1	rabaptin, RAB GTPase binding effector protein 1	Cytoplasm	transporter	
1.5	0.00266	0.122	210255_at		RAD51B	RAD51 paralog B	Nucleus	enzyme	
1.6	0.0208	0.198	216125_s_at		RANBP9	RAN binding protein 9	Nucleus	other	
2.02	0.0466	0.256	213280_at		RAP1GAP2	RAP1 GTPase activating protein 2	Cytoplasm	other	
1.59	0.0381	0.237	204681_s_at		RAPGEF5	Rap guanine nucleotide exchange factor 5	Nucleus	other	
1.84	0.00197	0.12	206636_at		RASA2	RAS p21 protein activator 2	Cytoplasm	other	
1.53	0.0104	0.162	212743_at		RCHY1	ring finger and CHY zinc finger domain containing 1	Nucleus	enzyme	
1.65	0.0311	0.221	218344_s_at		RCOR3	REST corepressor 3	Nucleus	transcription regulator	
1.72	0.0209	0.198	204128_s_at		RFC3	replication factor C subunit 3	Nucleus	enzyme	
1.53	0.00101	0.106	218430_s_at		RFX7	regulatory factor X7	Other	other	
-2.02	0.0324	0.225	218723_s_at		RGCC	regulator of cell cycle	Cytoplasm	other	
-1.66	0.0111	0.166	215588_x_at	D	RIOK3	RIO kinase 3	Cytoplasm	kinase	
1.52	0.0135	0.174	202129_s_at	D	RIOK3	RIO kinase 3	Cytoplasm	kinase	
1.62	0.00512	0.141	218425_at		RNF216	ring finger protein 216	Cytoplasm	enzyme	
1.54	0.0138	0.174	220788_s_at		RNF31	ring finger protein 31	Cytoplasm	enzyme	
-1.55	0.000701	0.105	213642_at		RPL27	ribosomal protein L27	Cytoplasm	other	

1.56	0.0415	0.244	220738_s_at		RPS6KA6	ribosomal protein S6 kinase A6	Cytoplasm	kinase	
1.57	0.0266	0.211	219684_at		RTP4	receptor transporter protein 4	Plasma Membrane	other	
-2.02	0.00312	0.126	213939_s_at	D	RUFY3	RUN and FYVE domain containing 3	Plasma Membrane	other	
-1.7	0.00491	0.14	213430_at	D	RUFY3	RUN and FYVE domain containing 3	Plasma Membrane	other	
1.64	0.0393	0.239	204198_s_at		RUNX3	runt related transcription factor 3	Nucleus	transcription regulator	
1.54	0.001	0.106	215064_at		SC5D	sterol-C5-desaturase	Cytoplasm	enzyme	
1.66	0.00622	0.147	212311_at	D	SEL1L3	SEL1L family member 3	Nucleus	other	
1.74	0.0134	0.173	212314_at	D	SEL1L3	SEL1L family member 3	Nucleus	other	
2.33	0.00319	0.127	220735_s_at		SENP7	SUMO specific peptidase 7	Nucleus	peptidase	
2.1	0.00524	0.141	212415_at		SEPT6	septin 6	Cytoplasm	other	
1.54	0.00963	0.16	216899_s_at		SKAP2	src kinase associated phosphoprotein 2	Cytoplasm	other	
-2.43	0.021	0.198	219915_s_at		SLC16A10	solute carrier family 16 member 10	Plasma Membrane	transporter	
-1.95	0.00854	0.157	207444_at		SLC22A13	solute carrier family 22 member 13	Plasma Membrane	transporter	
1.66	0.002	0.12	205421_at		SLC22A3	solute carrier family 22 member 3	Plasma Membrane	transporter	
-2.5	0.0307	0.22	205896_at		SLC22A4	solute carrier family 22 member 4	Plasma Membrane	transporter	
-1.52	0.014	0.175	210686_x_at		SLC25A16	solute carrier family 25 member 16	Cytoplasm	transporter	
-1.52	0.0083	0.156	201917_s_at		SLC25A36	solute carrier family 25 member 36	Cytoplasm	transporter	
-1.55	0.0036	0.132	203971_at		SLC31A1	solute carrier family 31 member 1	Plasma Membrane	transporter	
-3.77	0.0457	0.254	217530_at		SLC34A1	solute carrier family 34 member 1	Plasma Membrane	transporter	
-1.61	0.00092	0.106	209711_at		SLC35D1	solute carrier family 35 member D1	Cytoplasm	transporter	
-1.77	0.0141	0.175	220796_x_at		SLC35E1	solute carrier family 35 member E1	Cytoplasm	other	
1.56	0.0354	0.231	216504_s_at		SLC39A8	solute carrier family 39 member 8	Extracellular Space	transporter	
1.65	0.0395	0.239	217859_s_at		SLC39A9	solute carrier family 39 member 9	Other	transporter	
-2.91	0.0254	0.209	210739_x_at		SLC4A4	solute carrier family 4 member 4	Plasma Membrane	transporter	
-1.75	0.0139	0.174	216603_at	D	SLC7A8	solute carrier family 7 member 8	Plasma Membrane	transporter	
-2.36	0.0209	0.198	217248_s_at	D	SLC7A8	solute carrier family 7 member 8	Plasma Membrane	transporter	

-2.53	0.0226	0.202	216604_s_at	D	SLC7A8	solute carrier family 7 member 8	Plasma Membrane	transporter	
1.93	0.0396	0.24	219229_at		SLCO3A1	solute carrier organic anion transporter family member 3A1	Plasma Membrane	transporter	
1.51	0.0468	0.256	203813_s_at		SLIT3	slit guidance ligand 3	Extracellular Space	other	
1.51	0.0104	0.163	205188_s_at		SMAD5	SMAD family member 5	Nucleus	transcription regulator	
1.6	0.00561	0.143	212257_s_at	D	SMARCA2	SWI/SNF related, matrix associated, actin dependent regulator of chromatin, subfamily a, member 2	Nucleus	transcription regulator	
1.67	0.0164	0.184	206544_x_at	D	SMARCA2	SWI/SNF related, matrix associated, actin dependent regulator of chromatin, subfamily a, member 2	Nucleus	transcription regulator	
1.57	0.0343	0.229	204240_s_at		SMC2	structural maintenance of chromosomes 2	Nucleus	transporter	
1.75	0.00978	0.16	212577_at	D	SMCHD1	structural maintenance of chromosomes flexible hinge domain containing 1	Nucleus	enzyme	
1.52	0.019	0.192	212569_at	D	SMCHD1	structural maintenance of chromosomes flexible hinge domain containing 1	Nucleus	enzyme	
1.57	0.00131	0.113	218032_at		SNN	stannin	Plasma Membrane	other	
-1.54	0.000904	0.106	217040_x_at		SOX15	SRY-box 15	Nucleus	transcription regulator	
1.93	0.037	0.234	219993_at		SOX17	SRY-box 17	Nucleus	transcription regulator	
1.75	0.0193	0.192	201417_at		SOX4	SRY-box 4	Nucleus	transcription regulator	
1.52	0.00889	0.158	207724_s_at		SPAST	spastin	Nucleus	enzyme	
1.51	0.00585	0.144	220299_at		SPATA6	spermatogenesis associated 6	Cytoplasm	other	
1.55	0.0116	0.168	218817_at		SPCS3	signal peptidase complex subunit 3	Cytoplasm	peptidase	
-7.45	0.0479	0.259	206239_s_at		SPINK1	serine peptidase inhibitor, Kazal type 1	Extracellular Space	other	
1.62	0.0323	0.224	216202_s_at		SPTLC2	serine palmitoyltransferase long chain base subunit 2	Cytoplasm	enzyme	
1.72	0.00636	0.148	201742_x_at		SRSF1	serine and arginine rich splicing factor 1	Nucleus	other	

-1.88	0.00149	0.117	213742_at		SRSF11	serine and arginine rich splicing factor 11	Nucleus	other	
1.51	0.0184	0.19	202816_s_at		SS18	SS18, nBAF chromatin remodeling complex subunit	Nucleus	transcription regulator	
-1.68	0.00518	0.141	214597_at		SSTR2	somatostatin receptor 2	Plasma Membrane	G-protein coupled receptor	pasireotide, BIM 23A760, edotreotide, octreotide/prednisone, everolimus/pasireotide, 177Lu-edotreotide, haloperidol/octreotide, XmAb18087, dexamethasone/metoclopramide/octreotide, methylprednisolone/octreotide, octreotide/prochlorperazine, lanreotide, octreotide
-1.61	0.00862	0.157	207871_s_at		ST7	suppression of tumorigenicity 7	Other	other	
1.87	0.0272	0.212	206118_at		STAT4	signal transducer and activator of transcription 4	Nucleus	transcription regulator	
1.73	0.00451	0.137	202695_s_at	D	STK17A	serine/threonine kinase 17a	Nucleus	kinase	
1.53	0.0288	0.216	202693_s_at	D	STK17A	serine/threonine kinase 17a	Nucleus	kinase	
1.5	0.012	0.168	212800_at		STX6	syntaxin 6	Cytoplasm	transporter	
2.4	0.0301	0.219	203999_at		SYT1	synaptotagmin 1	Cytoplasm	transporter	
1.8	0.00233	0.122	221618_s_at		TAF9B	TATA-box binding protein associated factor 9b	Nucleus	transcription regulator	
1.95	0.0061	0.146	210458_s_at		TANK	TRAF family member associated NFKB activator	Cytoplasm	other	
1.5	0.00243	0.122	221428_s_at		TBL1XR1	transducin beta like 1 X-linked receptor 1	Nucleus	transcription regulator	
1.53	0.0138	0.174	216511_s_at		TCF7L2	transcription factor 7 like 2	Nucleus	transcription regulator	
1.54	0.0455	0.254	214862_x_at		TET1	tet methylcytosine dioxygenase 1	Nucleus	enzyme	
1.69	0.000845	0.106	204147_s_at		TFDP1	transcription factor Dp-1	Nucleus	transcription regulator	
1.59	0.0286	0.215	209676_at		TFPI	tissue factor pathway inhibitor	Extracellular Space	other	dalteparin
3.19	0.0178	0.188	209278_s_at		TFPI2	tissue factor pathway inhibitor 2	Extracellular Space	other	
-1.88	0.0306	0.22	208691_at		TFRC	transferrin receptor	Plasma Membrane	transporter	CALAA-01
-1.6	0.0313	0.222	212040_at		TGOLN2	trans-golgi network protein 2	Cytoplasm	other	
1.56	0.0383	0.238	201109_s_at		THBS1	thrombospondin 1	Extracellular Space	other	
-1.95	0.00931	0.16	213869_x_at	D	THY1	Thy-1 cell surface antigen	Plasma Membrane	other	
-1.8	0.0153	0.181	208850_s_at	D	THY1	Thy-1 cell surface antigen	Plasma Membrane	other	
-1.89	0.016	0.183	208851_s_at	D	THY1	Thy-1 cell surface antigen	Plasma Membrane	other	

2.15	0.0365	0.233	204468_s_at		TIE1	tyrosine kinase with immunoglobulin like and EGF like domains 1	Plasma Membrane	kinase	
1.65	0.000981	0.106	214168_s_at		TJP1	tight junction protein 1	Plasma Membrane	other	
1.5	0.00699	0.149	203221_at	D	TLE1	transducin like enhancer of split 1	Nucleus	transcription regulator	
1.73	0.0122	0.169	203222_s_at	D	TLE1	transducin like enhancer of split 1	Nucleus	transcription regulator	
1.84	0.00178	0.12	210379_s_at	D	TLK1	tousled like kinase 1	Nucleus	kinase	
2.02	0.00253	0.122	211077_s_at	D	TLK1	tousled like kinase 1	Nucleus	kinase	
1.72	0.00304	0.125	209387_s_at	D	TM4SF1	transmembrane 4 L six family member 1	Plasma Membrane	other	
1.54	0.00733	0.15	209386_at	D	TM4SF1	transmembrane 4 L six family member 1	Plasma Membrane	other	
1.7	0.00968	0.16	215034_s_at	D	TM4SF1	transmembrane 4 L six family member 1	Plasma Membrane	other	
1.52	0.049	0.261	204427_s_at		TMED2	transmembrane p24 trafficking protein 2	Cytoplasm	transporter	
1.57	0.0138	0.174	209655_s_at		TMEM47	transmembrane protein 47	Plasma Membrane	other	
1.57	0.0277	0.213	65630_at		TMEM80	transmembrane protein 80	Other	other	
1.68	0.00131	0.113	209753_s_at	D	TMPO	thymopoietin	Nucleus	other	
2.14	0.00952	0.16	209754_s_at	D	TMPO	thymopoietin	Nucleus	other	
1.51	0.044	0.25	203432_at	D	TMPO	thymopoietin	Nucleus	other	
1.66	0.00192	0.12	208097_s_at		TMX1	thioredoxin related transmembrane protein 1	Cytoplasm	enzyme	
1.64	0.00614	0.146	210260_s_at	D	TNFAIP8	TNF alpha induced protein 8	Cytoplasm	other	
1.51	0.0198	0.194	208296_x_at	D	TNFAIP8	TNF alpha induced protein 8	Cytoplasm	other	
-1.86	0.00966	0.16	207730_x_at		TNFAIP8L1	TNF alpha induced protein 8 like 1	Cytoplasm	other	
1.56	0.00243	0.122	216100_s_at		TOR1AIP1	torsin 1A interacting protein 1	Nucleus	other	
1.56	0.00152	0.118	201746_at		TP53	tumor protein p53	Nucleus	transcription regulator	APR-246, cenersen, ALT-801, CGM097, kevetrin, azurin 50-77, COTI-2, BI 907828
-1.82	0.0247	0.207	217914_at		TPCN1	two pore segment channel 1	Plasma Membrane	ion channel	
1.55	0.0158	0.183	221218_s_at		TPK1	thiamin pyrophosphokinase 1	Cytoplasm	kinase	
2.08	0.0322	0.224	209670_at	D	TRAC	T cell receptor alpha constant	Plasma Membrane	other	
2.44	0.0386	0.238	210972_x_at	D	TRAC	T cell receptor alpha constant	Plasma Membrane	other	
1.69	0.0329	0.226	205804_s_at		TRAF3IP3	TRAF3 interacting protein 3	Other	other	

Table K: Time dependent changes in gene expression in TAC + MMF Group shown in Figure 8

2.92	0.00757	0.151	210915_x_at	D	TRBC1	T cell receptor beta constant 1	Plasma Membrane	other	
4.12	0.0165	0.184	211796_s_at	D	TRBC1	T cell receptor beta constant 1	Plasma Membrane	other	
2.23	0.0263	0.21	213193_x_at	D	TRBC1	T cell receptor beta constant 1	Plasma Membrane	other	
1.69	0.0274	0.213	206308_at		TRDMT1	tRNA aspartic acid methyltransferase 1	Nucleus	enzyme	
1.62	0.00691	0.149	202479_s_at		TRIB2	tribbles pseudokinase 2	Plasma Membrane	kinase	
1.61	0.00758	0.151	205150_s_at		TRIL	TLR4 interactor with leucine rich repeats	Other	other	
1.72	0.0248	0.207	214908_s_at		TRRAP	transformation/transcription domain associated protein	Nucleus	transcription regulator	
-1.55	0.0097	0.16	218245_at		TSKU	tsukushi, small leucine rich proteoglycan	Extracellular Space	other	
1.59	0.0038	0.134	214007_s_at		TWF1	twinfilin actin binding protein 1	Cytoplasm	kinase	
-1.51	0.0103	0.162	216609_at		TXN	thioredoxin	Cytoplasm	enzyme	PX-12
1.51	0.00811	0.155	208760_at		UBE2I	ubiquitin conjugating enzyme E2 I	Nucleus	enzyme	
1.5	0.00569	0.143	202316_x_at		UBE4B	ubiquitination factor E4B	Cytoplasm	enzyme	
1.51	0.0184	0.19	204062_s_at		ULK2	unc-51 like autophagy activating kinase 2	Cytoplasm	kinase	
-2.43	0.0233	0.204	221173_at		USH1C	USH1 protein network component harmonin	Plasma Membrane	other	
1.75	0.00122	0.11	215886_x_at		USP12	ubiquitin specific peptidase 12	Cytoplasm	peptidase	
1.57	0.0282	0.214	212381_at		USP24	ubiquitin specific peptidase 24	Nucleus	peptidase	
1.52	0.000174	0.0907	212065_s_at		USP34	ubiquitin specific peptidase 34	Extracellular Space	peptidase	
2.2	0.00242	0.122	206624_at		USP9Y	ubiquitin specific peptidase 9 Y-linked	Cytoplasm	peptidase	
1.79	0.00619	0.147	205139_s_at		UST	uronyl 2-sulfotransferase	Cytoplasm	enzyme	
2.33	0.000788	0.106	213023_at	D	UTRN	utrophin	Plasma Membrane	transmembrane receptor	
2.24	0.0015	0.117	213022_s_at	D	UTRN	utrophin	Plasma Membrane	transmembrane receptor	
1.51	0.00908	0.158	219330_at		VANGL1	VANGL planar cell polarity protein 1	Cytoplasm	other	
1.51	0.0494	0.263	209822_s_at		VLDLR	very low density lipoprotein receptor	Plasma Membrane	transporter	
1.86	0.0172	0.187	215711_s_at		WEE1	WEE1 G2 checkpoint kinase	Nucleus	kinase	adavosertib
-1.83	0.00188	0.12	206213_at		WNT10B	Wnt family member 10B	Extracellular Space	other	

Table K: Time dependent changes in gene expression in TAC + MMF Group shown in Figure 8

1.59	0.0218	0.199	205990_s_at	D	WNT5A	Wnt family member 5A	Extracellular Space	cytokine	
2.11	0.0421	0.246	213425_at	D	WNT5A	Wnt family member 5A	Extracellular Space	cytokine	
-1.57	0.0121	0.169	71933_at		WNT6	Wnt family member 6	Extracellular Space	other	
2.49	0.0349	0.229	206366_x_at		XCL1	X-C motif chemokine ligand 1	Extracellular Space	cytokine	
2.83	0.0276	0.213	214567_s_at		XCL2	X-C motif chemokine ligand 2	Extracellular Space	cytokine	
-1.58	0.00901	0.158	207598_x_at		XRCC2	X-ray repair cross complementing 2	Nucleus	enzyme	
1.64	0.000004	0.0515	214659_x_at		YLPM1	YLP motif containing 1	Nucleus	transcription regulator	
1.67	0.0464	0.256	211902_x_at		YME1L1	YME1 like 1 ATPase	Cytoplasm	peptidase	
-1.61	0.0299	0.218	222357_at		ZBTB20	zinc finger and BTB domain containing 20	Nucleus	transcription regulator	
-2.1	0.0151	0.18	206169_x_at		ZC3H7B	zinc finger CCCH-type containing 7B	Nucleus	other	
1.76	0.0182	0.189	219247_s_at		ZDHHC14	zinc finger DHHC-type containing 14	Cytoplasm	enzyme	
1.5	0.00266	0.122	217741_s_at		ZFAND5	zinc finger AN1-type containing 5	Nucleus	other	
1.55	0.0326	0.225	207068_at		ZFP37	ZFP37 zinc finger protein	Nucleus	transcription regulator	
1.73	0.022	0.2	202137_s_at		ZMYND11	zinc finger MYND-type containing 11	Nucleus	transcription regulator	
-1.69	0.0201	0.195	214715_x_at		ZNF160	zinc finger protein 160	Nucleus	transcription regulator	
1.6	0.0019	0.12	207304_at		ZNF45	zinc finger protein 45	Nucleus	transcription regulator	
1.99	0.000863	0.106	222283_at		ZNF480	zinc finger protein 480	Nucleus	transcription regulator	
2.76	0.0144	0.175	207781_s_at		ZNF711	zinc finger protein 711	Nucleus	transcription regulator	
